# Global prevalence and burden of depressive and anxiety disorders in 204 countries and territories in 2020 due to the COVID-19 pandemic

**DOI:** 10.1016/S0140-6736(21)02143-7

**Published:** 2021-11-06

**Authors:** COVID-19 Mental Disorders Collaborators, Damian F Santomauro, Ana M Mantilla Herrera, Jamileh Shadid, Peng Zheng, Charlie Ashbaugh, David M Pigott, Cristiana Abbafati, Christopher Adolph, Joanne O Amlag, Aleksandr Y Aravkin, Bree L Bang-Jensen, Gregory J Bertolacci, Sabina S Bloom, Rachel Castellano, Emma Castro, Suman Chakrabarti, Jhilik Chattopadhyay, Rebecca M Cogen, James K Collins, Xiaochen Dai, William James Dangel, Carolyn Dapper, Amanda Deen, Megan Erickson, Samuel B Ewald, Abraham D Flaxman, Joseph Jon Frostad, Nancy Fullman, John R Giles, Ababi Zergaw Giref, Gaorui Guo, Jiawei He, Monika Helak, Erin N Hulland, Bulat Idrisov, Akiaja Lindstrom, Emily Linebarger, Paulo A Lotufo, Rafael Lozano, Beatrice Magistro, Deborah Carvalho Malta, Johan C Månsson, Fatima Marinho, Ali H Mokdad, Lorenzo Monasta, Paulami Naik, Shuhei Nomura, James Kevin O'Halloran, Samuel M Ostroff, Maja Pasovic, Louise Penberthy, Robert C Reiner Jr, Grace Reinke, Antonio Luiz P Ribeiro, Aleksei Sholokhov, Reed J D Sorensen, Elena Varavikova, Anh Truc Vo, Rebecca Walcott, Stefanie Watson, Charles Shey Wiysonge, Bethany Zigler, Simon I Hay, Theo Vos, Christopher J L Murray, Harvey A Whiteford, Alize J Ferrari

## Abstract

**Background:**

Before 2020, mental disorders were leading causes of the global health-related burden, with depressive and anxiety disorders being leading contributors to this burden. The emergence of the COVID-19 pandemic has created an environment where many determinants of poor mental health are exacerbated. The need for up-to-date information on the mental health impacts of COVID-19 in a way that informs health system responses is imperative. In this study, we aimed to quantify the impact of the COVID-19 pandemic on the prevalence and burden of major depressive disorder and anxiety disorders globally in 2020.

**Methods:**

We conducted a systematic review of data reporting the prevalence of major depressive disorder and anxiety disorders during the COVID-19 pandemic and published between Jan 1, 2020, and Jan 29, 2021. We searched PubMed, Google Scholar, preprint servers, grey literature sources, and consulted experts. Eligible studies reported prevalence of depressive or anxiety disorders that were representative of the general population during the COVID-19 pandemic and had a pre-pandemic baseline. We used the assembled data in a meta-regression to estimate change in the prevalence of major depressive disorder and anxiety disorders between pre-pandemic and mid-pandemic (using periods as defined by each study) via COVID-19 impact indicators (human mobility, daily SARS-CoV-2 infection rate, and daily excess mortality rate). We then used this model to estimate the change from pre-pandemic prevalence (estimated using Disease Modelling Meta-Regression version 2.1 [known as DisMod-MR 2.1]) by age, sex, and location. We used final prevalence estimates and disability weights to estimate years lived with disability and disability-adjusted life-years (DALYs) for major depressive disorder and anxiety disorders.

**Findings:**

We identified 5683 unique data sources, of which 48 met inclusion criteria (46 studies met criteria for major depressive disorder and 27 for anxiety disorders). Two COVID-19 impact indicators, specifically daily SARS-CoV-2 infection rates and reductions in human mobility, were associated with increased prevalence of major depressive disorder (regression coefficient [*B*] 0·9 [95% uncertainty interval 0·1 to 1·8; p=0·029] for human mobility, 18·1 [7·9 to 28·3; p=0·0005] for daily SARS-CoV-2 infection) and anxiety disorders (0·9 [0·1 to 1·7; p=0·022] and 13·8 [10·7 to 17·0; p<0·0001]. Females were affected more by the pandemic than males (*B* 0·1 [0·1 to 0·2; p=0·0001] for major depressive disorder, 0·1 [0·1 to 0·2; p=0·0001] for anxiety disorders) and younger age groups were more affected than older age groups (−0·007 [–0·009 to −0·006; p=0·0001] for major depressive disorder, −0·003 [–0·005 to −0·002; p=0·0001] for anxiety disorders). We estimated that the locations hit hardest by the pandemic in 2020, as measured with decreased human mobility and daily SARS-CoV-2 infection rate, had the greatest increases in prevalence of major depressive disorder and anxiety disorders. We estimated an additional 53·2 million (44·8 to 62·9) cases of major depressive disorder globally (an increase of 27·6% [25·1 to 30·3]) due to the COVID-19 pandemic, such that the total prevalence was 3152·9 cases (2722·5 to 3654·5) per 100 000 population. We also estimated an additional 76·2 million (64·3 to 90·6) cases of anxiety disorders globally (an increase of 25·6% [23·2 to 28·0]), such that the total prevalence was 4802·4 cases (4108·2 to 5588·6) per 100 000 population. Altogether, major depressive disorder caused 49·4 million (33·6 to 68·7) DALYs and anxiety disorders caused 44·5 million (30·2 to 62·5) DALYs globally in 2020.

**Interpretation:**

This pandemic has created an increased urgency to strengthen mental health systems in most countries. Mitigation strategies could incorporate ways to promote mental wellbeing and target determinants of poor mental health and interventions to treat those with a mental disorder. Taking no action to address the burden of major depressive disorder and anxiety disorders should not be an option.

**Funding:**

Queensland Health, National Health and Medical Research Council, and the Bill and Melinda Gates Foundation.

**Research in context**

**Evidence before this study**

For our study, we did a systematic literature review of the peer-reviewed and grey literature for studies published between Jan 1, 2020, and Jan 29, 2021, investigating prevalence of depressive and anxiety disorders during the COVID-19 pandemic. Most of the identified work consisted of surveys done for a specific location, at a snapshot in time, and indicated an increase in prevalence of depressive and anxiety disorders during the COVID-19 pandemic. We also found several systematic reviews and meta-analyses to summarise these studies; however, these studies were either qualitative syntheses or simply presented pooled estimates across studies. We did not find any studies that attempted to synthesise the global prevalence and burden of depressive and anxiety disorders due to the COVID-19 pandemic for the year 2020.

**Added value of this study**

This study is the first to quantify the prevalence and burden of depressive and anxiety disorders by age, sex, and location globally. We compiled available evidence of the change in the prevalence of major depressive disorder and anxiety disorders during the COVID-19 pandemic. We then developed a model to estimate the prevalence of major depressive disorder and anxiety disorders by age, sex, and location on the basis of available indicators of the location-specific impact of the COVID-19 pandemic. We then used prevalence to estimate the burden of major depressive disorder and anxiety disorders due to the COVID-19 pandemic in 2020.

**Implications of all the available evidence**

We found that depressive and anxiety disorders increased during 2020 due to the COVID-19 pandemic. Even before the COVID-19 pandemic, depressive and anxiety disorders featured as leading causes of burden globally, despite the existence of intervention strategies that can reduce their effects. Meeting the added demand for mental health services due to COVID-19 will be difficult, but not impossible. Mitigation strategies should promote mental wellbeing and target determinants of poor mental health exacerbated by the pandemic, as well as interventions to treat those who develop a mental disorder.

## Introduction

Mental disorders are among the leading causes of the global health-related burden. The Global Burden of Diseases, Injuries, and Risk Factors Study (GBD) 2019 showed that the two most disabling mental disorders were depressive and anxiety disorders, both ranked among the top 25 leading causes of burden worldwide in 2019.^1^, ^2^ This burden was high across the entire lifespan, for both sexes, and across many locations.^2^ Perhaps more importantly, no reduction in the global prevalence or burden was detected for either disorder since 1990, despite compelling evidence of interventions that reduce their impact.^3^

The emergence of the COVID-19 pandemic in 2020 against this backdrop has raised many questions around the resulting effects on mental health via its direct psychological effects and long-term economic and social consequences.^4^ COVID-19 continues to spread across most of the world's populations^1^, ^2^ with significant health consequences and mortality among those who become infected.^5^ In addition to the direct effects of COVID-19, the pandemic has created an environment in which many determinants of mental health are also affected. Social restrictions, lockdowns, school and business closures, loss of livelihood, decreases in economic activity, and shifting priorities of governments in their attempt to control COVID-19 outbreaks all have the potential to substantially affect the mental health of the population. The need for up-to-date information on the global prevalence and burden of mental disorders incorporating the mental health impacts of COVID-19 in a way that informs health system responses has never been more urgent.

GBD 2020 is in the process of estimating the burden of 370 diseases and injuries 88 risk factors across 204 countries and territories. GBD 2020 is quantifying the burden using disability-adjusted life-years (DALYs), which represent the number of years of healthy life lost to either mortality or disability. Here, as part of GBD 2020, we present a method built on the mental disorder estimates presented in GBD 2019 by incorporating the effect of the COVID-19 pandemic. We quantify the impact of COVID-19 on the prevalence and burden of major depressive disorder and anxiety disorders by location, age, and sex in 2020.

## Methods

### Overview

First, we conducted a systematic literature review to assemble data from surveys measuring the effect of COVID-19 on the prevalence of depressive and anxiety disorders. Second, we used these data in a meta-analysis to (1) estimate the change in prevalence of major depressive disorder and anxiety disorders before versus during the COVID-19 pandemic, (2) predict (through the use of selected indicators of COVID-19 impact) the resulting change in prevalence of each disorder across all GBD locations, and (3) translate changes in prevalence to corresponding changes in burden estimates as years lived with disability (YLDs) and DALYs. A conceptual overview of this process is shown in the appendix (p 17). This study complies with the Guidelines for Accurate and Transparent Health Estimates Reporting (GATHER) recommendations (appendix pp 26–27)^6^ and all code used in the analyses can be found online.

### Case definitions

To ensure comparability in measurement, we used case definitions for major depressive disorder and anxiety disorders used within the GBD framework. These definitions adhere to criteria presented in the Diagnostic and Statistical Manual of Mental Disorder fourth edition text revision (DSM-IV-TR)^7^ and the tenth International Classification of Diseases and Related Health Problems (ICD-10).^8^ Detailed case definitions are in the appendix (p 6).

### Data sources

We conducted a systematic literature search to identify population surveys reporting on the prevalence of depressive or anxiety disorders, or both, during the COVID-19 pandemic. We used the Preferred Reporting Items for Systematic Reviews and Meta-Analyses (PRISMA) guidelines;^9^ our PRISMA checklist is in the appendix (pp 28–31), and the search protocol was registered with the International Prospective Register of Systematic Reviews (PROSPERO, CRD42021216590).

We searched for data sources published between Jan 1, 2020, and Jan 29, 2021. The search involved electronic searches of the peer-reviewed literature using PubMed, the grey literature (ie, the COVID-19: living map of the evidence by Eppi-centre, The DEPRESSD Project, Google Scholar, The Neurology and Neuropsychiatry of COVID-19 Blog, the WHO COVID 19 literature database, COVID-Minds, and the MedRxiv and PsyArXiv preprint servers), and expert consultation. Search strings for each search are in the appendix (p 5). No language restrictions were applied.

Eligible studies reported prevalence of depressive or anxiety disorders during the COVID-19 pandemic and had a pre-pandemic baseline. We used timelines for these periods as defined by each study. Prevalence surveys conducted during the pandemic could not be included without comparable pre-pandemic data (ie, using the same instrument, location, and age group) collected since 2013 to assess the change in prevalence. Longitudinal studies using samples representative of the general population were preferred, but cross-sectional studies were also included if comparable pre-pandemic prevalence data existed. Studies using random sampling were preferred; however, due to challenges imposed by the COVID-19 pandemic, there were few studies of this type. Studies using market research quota sampling were also included but were controlled for with a covariate in our analysis. Market research quota sampling is a non-probabilistic sampling strategy whereby participants are identified from a database to match the population of interest (a discussion of the use of these estimates is in the appendix [p 7]).^10^ Samples obtained via this method might produce results that differ from the general population.^11^, ^12^

Studies reporting probable cases of depressive or anxiety disorders using established screening measures were included due to the paucity of available survey data from during the COVID-19 pandemic using diagnostic instruments. In these instances, only prevalence estimates for cases that reached established thresholds for probable depressive or anxiety disorder were included. We assumed the predictive validity between probable versus diagnosed cases using these screening measures did not change during the COVID-19 pandemic compared with before the pandemic, and therefore the change in the prevalence of probable cases would be equivalent to the change in the prevalence of diagnosed cases (additional discussion of this assumption is in the appendix [pp 8–9]). We also included measures capturing symptoms of both depressive and anxiety disorders (eg, the K-6 Distress Scale), which were also controlled for with a covariate in analyses. For eligible studies, we extracted information on location, age, sex, prevalence, uncertainty, number of cases, sample size, recall period, disorder, diagnostic instrument, sampling strategy, and dates between which the survey was conducted. We extracted the most detailed data reported by age and sex.

We required indicators of the impact of the COVID-19 pandemic that had an association with prevalence of major depressive disorder and anxiety disorders, hereafter referred to as COVID-19 impact indicators. The risk factor of interest was the COVID-19 pandemic, with the COVID-19 impact indicators acting as proxies for the effect of COVID-19 in the population. A COVID-19 impact indicator had to capture an effect of COVID-19, be consistently measured across locations, and be consistently measured with sufficient granularity across time (preferably daily or weekly) over the course of the pandemic. Our process of selecting the COVID-19 impact indicators is detailed in the appendix (p 10). Ultimately, we considered three novel indicators of the COVID-19 pandemic: decreasing human mobility,^13^ estimated total (as opposed to reported) daily SARS-CoV-2 infection rate, and estimated daily excess mortality rate during the pandemic (including excess deaths occurring during the pandemic where COVID-19 was not reported as the underlying cause of death).^5^, ^13^

The estimation of human mobility, daily SARS-CoV-2 infection rate, and daily excess mortality rate was done by the Institute for Health Metrics and Evaluation (IHME) COVID-19 Forecasting Team and is described in detail elsewhere.^13^ In summary, human mobility was represented by a composite human mobility index representing daily change from pre-pandemic mobility. Data from mobile phone users provided by Facebook, Google, Descartes Labs, Safegraph, and Baidu, and data on physical distancing mandates informed a Gaussian process regression to estimate a time series for human mobility. Daily SARS-CoV-2 infection and excess mortality rates were estimated via a deterministic susceptible, exposed, infectious, and recovered (known as SEIR) compartmental framework and was informed by daily confirmed SARS-CoV-2 infections, COVID-19-related deaths, SARS-CoV-2 tests conducted, antibody seroprevalence, human mobility, physical distancing mandates, pneumonia seasonality, facemask use, and vaccine coverage.^5^, ^13^

GBD conducts routine systematic reviews of the epidemiology of major depressive disorder and anxiety disorders as part of the estimation of YLDs (and separately to the aforementioned systematic review of the change in prevalence due to COVID-19). The compiled epidemiological data for GBD 2020 includes studies done from 1980 to 2019 on the prevalence, incidence, remission, duration, and excess mortality of major depressive disorder and anxiety disorders. These data were identified through routine electronic searches of PubMed, PsycINFO, Embase, grey literature sources (including a review of the Global Health Data Exchange library), and consultation with experts. GBD 2020 uses Disease Modelling Meta-Regression version 2.1 (known as DisMod-MR 2.1), a Bayesian disease modelling meta-regression tool, to analyse these data and generate internally consistent estimates of prevalence, incidence, remission, and mortality by sex, location, year, and age group for each disorder.^14^ It also estimates prevalence for locations that are missing raw epidemiological data. GBD 2020 does this by estimating prevalence across a cascade down five levels of a geographical hierarchy: global, super-region, region, country or territory, and subnational locations. The prevalence from locations higher in the hierarchy act as priors for the prevalence for locations lower in the hierarchy. More detail on DisMod-MR 2.1 and the estimation of prevalence data for major depressive disorder and anxiety disorders has been published elsewhere.^1^, ^14^ Because no epidemiological data from the year 2020 informed the DisMod-MR 2.1 model for major depressive disorder and anxiety disorders, the prevalence produced for 2020 by age and sex and for each of the 204 countries and territories represented the prevalence of each disorder without the impact of the COVID-19 pandemic.

We made use of severity distributions and disability weights in GBD 2020 (appendix p 32). We apportioned the final disorder prevalence for the year 2020 into the categories of asymptomatic, mild, moderate, and severe with corresponding disability weights. Disability weights in GBD 2020 quantify health loss from a health state on a scale of 0 (no health loss) to 1 (equivalent to death). The process to estimate severity proportions and disability weights in GBD 2020 has been described elsewhere.^2^, ^15^

### Statistical analysis

We estimated the prevalence of major depressive disorder and anxiety disorders during the COVID-19 pandemic by first developing a model to predict adjustments to pre-pandemic prevalence of major depressive disorder and anxiety disorders based on COVID-19 impact indicators, and then modifying the pre-pandemic prevalence of major depressive disorder and anxiety disorders estimated from DisMod-MR 2.1. We conducted meta-regressions, using meta-regression—Bayesian, regularised, trimmed^16^ (MR-BRT), on the difference between logit disorder prevalence before the COVID-19 pandemic and during the COVID-19 pandemic. The process to build a final meta-regression model is described in detail in the appendix (pp 6–7). Briefly, potential bias covariates that were identified were cross-sectional comparisons informed by random samples, longitudinal comparisons informed by market research and quota samples, cross-sectional comparisons informed by market research and quota samples, and estimates representing combined symptoms of depressive and anxiety disorders (appendix p 32). Because only three studies used diagnostic instruments to measure prevalence, we did not have sufficient data to explore the effect of a bias covariate on data from screening scales identifying probable cases of depressive or anxiety disorders (appendix pp 8–9). Each unique sample was given a random intercept and random effects were placed on the COVID-19 impact indicators. We ran models separately for major depressive disorder and anxiety disorders. We also included age, sex, and most bias covariates as effect modifiers to ensure that the prevalence change remained zero when the COVID-19 impact was zero. The exception was the bias covariate for cross-sectional comparisons informed by market research and quota samples, which had random sample pre-pandemic baselines and therefore had a prevalence difference even when the COVID-19 impact indicator was zero. Sex was quantified by the proportion of female participants in the sample, and age was quantified as the mean age of the sample (or midpoint of the age range of the study sample when mean age was not reported).

Due to the strong collinearity between the COVID-19 impact indicators (inspected via Pearson correlation coefficients) and the need for age, sex, and bias covariates to be treated as effect modifiers on the impact indicators, we developed prevalence adjustment models via a two-step process. In step one, we used indicator models to develop a so-called index for the impact of COVID-19. Human mobility, daily SARS-CoV-2 infection rate, and daily excess mortality rate were included simultaneously in a meta-regression on the change in logit prevalence to quantify their independent effect on prevalence change. We used the coefficients from these models to calculate a single COVID-19 impact indicator for each disorder. In step two, we developed a final model for each disorder via backward elimination to regress the COVID-19 impact indicator, age, sex, and the bias covariates on the change in logit prevalence. The least significant covariate was iteratively removed until no improvement was seen in the Akaike information criterion. We assessed the generalisability of the model using a leave-one-country-out cross-validation analysis (appendix p 11).

We used the final disorder-specific models to predict the change in logit prevalence of major depressive disorder and anxiety disorders and adjust the DisMod-MR 2.1 prevalence estimates for the year 2020 via a Markov chain Monte Carlo simulation. We extracted 1000 samples from the probability distributions of the change in logit prevalence of major depressive disorder and anxiety disorders, and their logit prevalences from DisMod-MR 2.1, by age, sex, and location for every day of the year 2020, based on daily estimates of the significant COVID-19 impact indicators. We adjusted the logit prevalence by the change and inverse-logit transformed the result to estimate the adjusted daily prevalence. We estimated the average daily prevalence for the year 2020 by age, sex, and location as the point prevalence for the year (an applied example is shown in the appendix [p 18]). Once estimated, we divided the prevalences of major depressive disorder and anxiety disorders into sequela-specific prevalences using the GBD 2020 severity splits (appendix p 32). We report estimates with 95% uncertainty intervals (UIs), which represent the 25th and 975th ranked results across the 1000 samples and can be interpreted similarly to 95% CIs.

Consistent with GBD methods, we followed ICD rules for determining the underlying cause of death. DALYs for major depressive disorder and anxiety disorders were composed entirely of YLDs, which meant that, despite potential excess deaths, major depressive disorder and anxiety disorders were not considered underlying causes of death.^2^ We first estimated sequela-specific YLDs by multiplying sequela-specific prevalences by their respective disability weights. We then corrected the sequela-specific YLDs for comorbidities to adjust for the co-occurrence of causes of YLDs within GBD 2020; these methods have been described in detail elsewhere.^1^ For GBD 2020, burden is only estimated using the COVID-19-adjusted prevalence estimates. Therefore, we calculated baseline burden estimates by adjusting GBD 2020 burden estimates by the ratio between baseline prevalence and adjusted prevalence.

p values of less than 0.05 were considered to be significant. We did all analyses using DisMod MR-2.1, MR-BRT, and R (version 3.6.3).

### Role of the funding source

The funders of the study had no role in the study design, data collection, data analysis, data interpretation, or the writing of the report.

## Results

Of the 5683 unique data sources obtained from the systematic review, 1674 remained following title and abstract screening. In total, 46 studies met inclusion criteria for major depressive disorder and 27 for anxiety disorders (48 in total, one of which reported data across two regions; appendix p 19). A supplemental search for pre-pandemic baseline measures provided an additional 11 studies for major depressive disorder and seven studies for anxiety disorders. Study characteristics of included studies are in the appendix (pp 33–34). Most studies were from western Europe (n=22) and high-income North America (n=14), and the remaining studies were from Australasia (n=5), high-income Asia Pacific (n=5), east Asia (n=2), and central Europe (n=1; appendix p 20).

Human mobility and daily SARS-CoV-2 infection rate were significantly associated with the change in major depressive disorder and anxiety disorder prevalence (table 1). After controlling for human mobility and daily SARS-CoV-2 infection rate, daily excess mortality rate was not associated with the change in prevalence for either major depressive disorder or anxiety disorders. This was likely due to high collinearity between the daily excess mortality rate and the other two COVID-19 impact variables (*r* = 0·8 with daily SARS-CoV-2 infection rate and *r* = 0·8 with human mobility), which was less of an issue shared between the daily SARS-CoV-2 infection rate and human mobility (*r* = 0·5).

**Table 1:** Meta-regression coefficients from the indicator model on the change in major depressive disorder and anxiety disorders logit prevalences over the course of the COVID-19 pandemic in 2020

	**Major depressive disorder**		**Anxiety disorders**	
	*B* (95% UI)	p value	*B* (95% UI)	p value
Decreasing human mobility	0·9 (0·1 to 1·8)	0·029	0·9 (0·1 to 1·7)	0·022
Daily SARS-CoV-2 infection rate*	18·1 (7·9 to 28·3)	0·0005	13·8 (10·7 to 17·0)	<0·0001
Daily excess mortality rate*†	0·0 (−122·6 to 122·6)	>0·9999	0·0 (−112·5 to 112·5)	>0·9999

All indicators included in the model simultaneously and therefore control for each other. UI=uncertainty interval.

* Square-root transformed before analysis to correct for positive skew (appendix pp 6–7).

† Bayesian directional prior specified due to strong collinearity with other indicators.

Increases in the COVID-19 impact index (informed by the significant indicators of human mobility and daily SARS-CoV-2 infection rate) were associated with an increase in prevalence of major depressive disorder (0·4 [95% UI 0·1–0·6]) and anxiety disorders (0·4 [0·3–0·5]; table 2). For both disorders, females were affected more than males, and younger age groups were affected more than older age groups. Bias covariates for cross-sectional comparisons informed by random samples and longitudinal comparisons informed by market research and quota samples were not significant for either disorder (appendix p 67) and subsequently were dropped from the final models (table 2). Estimates representing combined depressive and anxiety disorder symptoms significantly overestimated the increase in prevalence for anxiety disorders 0·3 (0·1–0·4) but not for major depressive disorder (appendix p 67) and were not included in the major depressive disorder model. Cross-sectional market research and quota samples significantly overestimated the increase in prevalence for both major depressive disorder (0·9 [0·6–1·2]) and anxiety disorders (0·6 [0·2–1·0]; table 2). Results of our leave-one-country-out cross-validation analysis are in the appendix (p 11).

**Table 2:** Meta-regression coefficients on the change in major depressive disorder and anxiety disorders logit prevalences over the course of the COVID-19 pandemic in 2020

		**Major depressive disorder**		**Anxiety disorders**	
		*B* (95% UI)	p value	*B* (95% UI)	p value
COVID-19 impact index		0·4 (0·1 to 0·6)	0·0044	0·4 (0·3 to 0·5)	<0·0001
	Decreasing human mobility*	0·3 (0·03 to 0·8)	..	0·4 (0·1 to 0·8)	..
	Daily SARS-CoV-2 infection rate*†	6·6 (1·6 to 13·0)	..	6·0 (4·3 to 8·1)	..
Mean or midpoint age		−0·007 (−0·0009 to −0·0006)	0·0001	−0·003 (−0·005 to −0·0002)	0·0001
Proportion female		0·1 (0·1 to 0·2)	0·0001	0·1 (0·1 to 0·2)	0·0001
Combined depressive and anxiety disorder symptoms		..	..	0·3 (0·1 to 0·4)	0·0080
Cross-sectional market research and quota sample		0·9 (0·6 to 1·2)	<0·0001	0·6 (0·2 to 1·0)	0·0022

Longitudinal market research and quota sample was not significant, and so not included in the final model.

* Coefficients are estimated using the *B* of the COVID-19 impact index multiplied by the *B* of the COVID-19 impact indicators from the signal model and 95% UIs estimated via multiplying 1000 samples of the posterior distribution of these impact indicator covariates from the indicator model with 1000 samples of the posterior distribution of the indicator coefficient.

† Square-root transformed before analysis to correct for positive skew of the COVID-19 impact index coefficient (appendix pp 6–7). UI=uncertainty interval.

**Figure 1:**
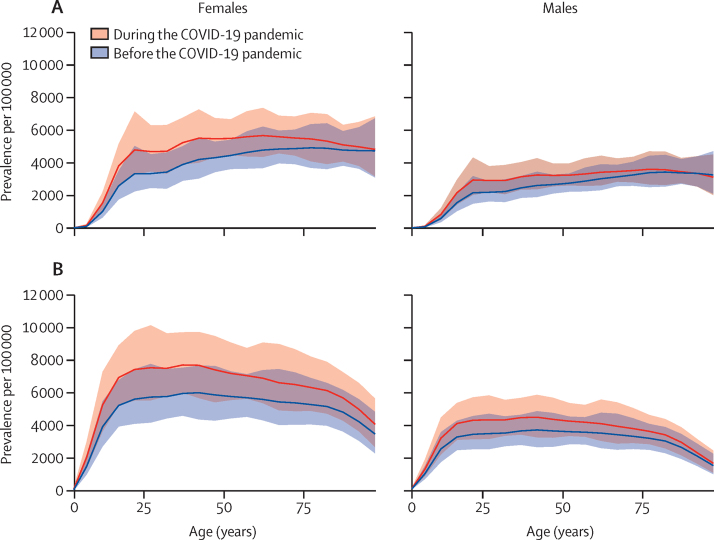
Global prevalence of major depressive disorder (A) and anxiety disorders (B) before and after adjustment for (ie, during) the COVID-19 pandemic, 2020, by age and sex

**Figure 2:**
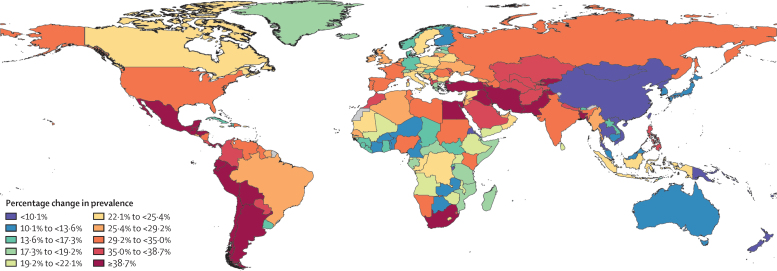
Change in the prevalence of major depressive disorder after adjustment for (ie, during) the COVID−19 pandemic, 2020

Before adjustment for the COVID-19 pandemic, the estimated global prevalence of major depressive disorder in 2020 was 2470·5 cases (95% UI 2143·5–2870·7) per 100 000 population, equivalent to 193 million (167–224) people (appendix pp 35–50). After adjustment for the COVID-19 pandemic, the estimated prevalence of major depressive disorder was 3152·9 cases (2722·5–3654·5) per 100 000 population, equivalent to 246 million (212–285) people. We estimated an additional 53·2 million (44·8–62·9) cases of major depressive disorder globally in 2020 due to the effects of COVID-19 (682·4 [574·1–807·2] new cases per 100 000 population, an increase of 27·6% [25·1–30·3]; table 3). Females had a greater increase in prevalence of major depressive disorder than males did, with 35·5 million (30·0–41·8) additional cases among females (equivalent to 912·5 [772·1–1075·2] per 100 000 females; a 29·8% [27·3–32·5] increase) compared with 17·7 million (14·7–21·3) additional cases in males (equivalent to 453·6 [376·3–545·0] per 100 000 males; a 24·0% [21·5–26·7] increase; data by sex by super-region and region are available on the Global Health Data Exchange). Global patterns of prevalence before and after adjustment for (ie, during) the COVID-19 pandemic, by age and sex, are presented in figure 1, and estimates of the change in prevalence of major depressive disorder are shown in figure 2 and in the appendix (p 21).

**Table 3:** Prevalence of major depressive disorder and anxiety disorders, by super-region, 2020

	**Major depressive disorder, per 100 000 population**				**Anxiety disorders, per 100 000 population**			
	Baseline (95% UI)	Additional (95% UI)	Final (95% UI)	Percentage change (95% UI)	Baseline (95% UI)	Additional (95% UI)	Final (95% UI)	Percentage change (95% UI)
Global	2470·5 (2143·5–2870·7)	682·4 (574·1–807·2)	3152·9 (2722·5–3654·5)	27·6 (25·1–30·3)	3824·9 (3283·3–4468·1)	977·5 (824·8–1161·6)	4802·4 (4108·2–5588·6)	25·6 (23·2–28·0)
Central Europe, eastern Europe, and central Asia	2519·7 (2185·0–2911·5)	741·6 (579·1–941·3)	3261·3 (2798·6–3804·8)	29·4 (23·9–35·8)	3274·3 (2801·2–3821·9)	981·0 (774·1–1214·4)	4255·3 (3593·1–4970·8)	30·0 (24·9–35·0)
High-income	3103·3 (2735·6–3526·4)	840·1 (671·7–1030·4)	3943·3 (3466·9–4516·1)	27·1 (22·6–31·5)	5356·8 (4609·1–6233·3)	1349·0 (1044·1–1678·8)	6705·7 (5773·4–7829·4)	25·2 (20·3–30·7)
Latin America and Caribbean	2626·8 (2291·4–3034·4)	914·2 (737·4–1127·5)	3541·0 (3063·3–4097·7)	34·8 (29·5–40·7)	5705·9 (4865·4–6732·9)	1804·1 (1425·8–2225·1)	7510·0 (6397·9–8786·6)	31·7 (25·8–37·7)
North Africa and Middle East	3321·4 (2752·3–4013·2)	1235·2 (896·1–1642·5)	4556·6 (3729·1–5578·3)	37·2 (29·5–46·0)	5148·9 (4210·4–6289·4)	1664·8 (1178·0–2251·6)	6813·6 (5557·9–8391·8)	32·4 (24·9–41·1)
South Asia	2664·2 (2313·9–3099·5)	962·6 (761·6–1187·1)	3626·8 (3122·5–4232·7)	36·1 (29·7–42·8)	3019·7 (2590·4–3531·6)	1058·3 (813·0–1318·7)	4077·9 (3459·3–4786·7)	35·1 (28·2–42·0)
Southeast Asia, east Asia, and Oceania	1707·8 (1492·4–1958·7)	195·8 (121·8–281·4)	1903·6 (1656·1–2194·3)	11·5 (7·2–16·0)	3367·2 (2903·3–3891·5)	466·0 (307·2–632·0)	3833·2 (3281·8–4478·2)	13·8 (9·3–18·3)
Sub-Saharan Africa	2429·0 (2048·0–2910·2)	559·0 (423·3–722·8)	2988·0 (2513·5–3583·4)	23·0 (18·3–27·9)	3001·9 (2465·1–3671·3)	644·0 (479·0–829·9)	3645·9 (2985·7–4475·5)	21·5 (17·1–25·7)

UI=uncertainty interval.

**Figure 3:**
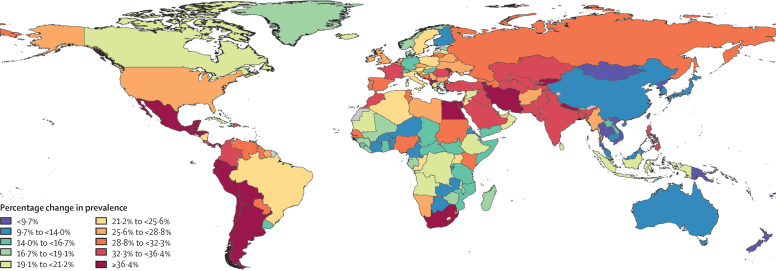
Change in the prevalence of anxiety disorders after adjustment for (ie, during) the COVID−19 pandemic, 2020

Before adjustment for the COVID-19 pandemic, the estimated global prevalence of anxiety disorders in 2020 was 3824·9 (95% UI 3283·3–4468·1) per 100 000 population, which is equivalent to 298 million (256–348) people (appendix pp 51–66). After adjustment for the COVID-19 pandemic, the global prevalence of anxiety disorders in 2020 was 4802·4 (4108·2–5588·6), equivalent to 374 million (320–436) people. We estimated an additional 76·2 million (64·3–90·6) cases of anxiety disorders in 2020 due to the COVID-19 pandemic (977·5 [824·8–1161·6] new cases per 100 000 population; an increase of 25·6% (23·2–28·0) globally (table 3). Females had a greater increase in prevalence than males did, with 51·8 million (43·8–61·1) additional cases among females (equivalent to 1332·1 [1126·1–1573·2] per 100 000 females; a 27·9% [25·6–30·4] increase) compared with 24·4 million (20·3–29·5) additional cases among males (equivalent to 625·0 [518·3–755·3] per 100 000 males; a 21·7% [19·3–24·1] increase; data by sex by super-region and region will be available on the Global Health Data Exchange after full release of GBD 2020). Global patterns of prevalence before and after adjustment for (ie, during) the COVID-19 pandemic, by age and sex, are presented in figure 1, and estimates of the change in prevalence of anxiety disorders are shown in figure 3 and in the appendix (p 22).

**Figure 4:**
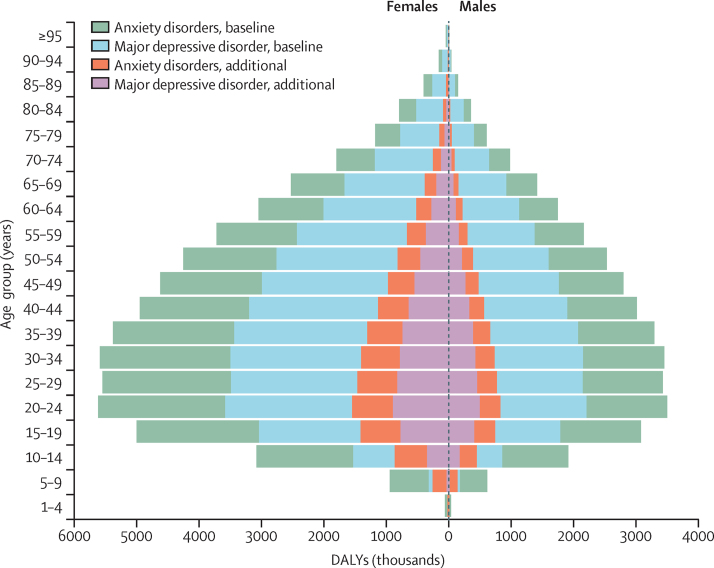
Global burden of major depressive disorder and anxiety disorders by age and sex, 2020 Baseline refers to pre-pandemic DALYs and additional refers to additional burden due to the COVID−19 pandemic. DALYs=disability-adjusted life-years.

Before adjustment for the COVID-19 pandemic, major depressive disorder was responsible for 38·7 million (95% UI 26·4–53·9) DALYs globally, equivalent to 497·0 DALYs (338·3–691·1) per 100 000 population. After adjustment for the COVID-19 pandemic, major depressive disorder was responsible for 49·4 million (33·6–68·7) DALYs, equivalent to 634·1 DALYs (431·3–881·0) per 100 000 population (appendix pp 35–50). We estimated that the COVID-19 pandemic led to an additional 10·7 million (7·21–14·9) DALYs for major depressive disorder globally, of which 7·07 million (4·80–9·80) were among females and 3·62 million (2·40–5·09) were among males. The global major depressive disorder additional DALY rate due to the COVID-19 pandemic was 137·1 DALYs (92·5–190·6) per 100 000 population, 182·0 (123·5–252·2) per 100 000 females, and 92·5 (61·5–130·3) per 100 000 males (DALY rates by sex by region will be available on the Global Health Data Exchange after full release of GBD 2020). The burden of DALYS due to major depressive disorder by age and sex is presented in figure 4.

Before adjustment for the COVID-19 pandemic, anxiety disorders were responsible for 35·5 million (95% UI 23·9–50·1) DALYs globally, equivalent to 454·8 DALYs (307·0–642·5) per 100 000 population (appendix pp 51–66). After adjustment for the COVID-19 pandemic, anxiety disorders were responsible for 44·5 million (30·2–62·5) DALYs globally, equivalent to 570·9 (387·3–802·2) per 100 000 population. Anxiety disorders were responsible for an additional 9·05 million (6·18–12·8) DALYs due to the pandemic, of which 6·11 million (4·21–8·56) DALYs were among females and 2·94 million (1·97–4·19) among males. The global anxiety disorder additional DALY rate due to the COVID-19 pandemic was 116·1 (79·3–163·8) per 100 000 population, 157·2 (108·3–220·3) per 100 000 females, and 75·3 (50·3–107·1) per 100 000 males. The burden of DALYS due to anxiety disorders by age and sex is presented in figure 4.

## Discussion

In this study, we estimated a substantial increase in the prevalence and burden of major depressive disorder and anxiety disorders as a result of the COVID-19 pandemic. This is, to our knowledge, the first study to systematically identify and analyse population mental health survey data and quantify the resulting impact of the COVID-19 pandemic on the prevalence of these two disorders by location, age, and sex in 2020.

Increases in the prevalence of major depressive disorder and anxiety disorders during 2020 were both associated with increasing SARS-CoV-2 infection rates and decreasing human mobility. These two COVID-19 impact indicators incorporated the combined effects of the spread of the virus, lockdowns, stay-at-home orders, decreased public transport, school and business closures, and decreased social interactions, among other factors. We estimated that countries hit hardest by the pandemic during 2020 had the greatest increases in prevalence of these disorders.

The two COVID-19 impact indicators used in our model should not be interpreted as risk factors for major depressive disorder and anxiety disorders. The risk factor of interest was the COVID-19 pandemic, with these two indicators acting as proxies for the effect of COVID-19 in the population. The COVID-19 pandemic is occurring against a complex backdrop of a range of social determinants of mental health, as well as well known inequalities within these determinants. The greater increase in disorder prevalence among females than among males, which resulted in an even greater sex difference in prevalence than before the pandemic, was anticipated because females are more likely to be affected by the social and economic consequences of the pandemic.^17^, ^18^, ^19^ Additional carer and household responsibilities due to school closures or family members becoming unwell are more likely to fall on women.^17^ Women are more likely to be financially disadvantaged during the pandemic due to lower salaries, less savings, and less secure employment than their male counterparts.^17^, ^18^, ^19^ They are also more likely to be victims of domestic violence, the prevalence of which increased during periods of lockdown and stay-at-home orders.^20^, ^21^ We also estimated greater change in the prevalence of major depressive disorder and anxiety disorders among younger age groups than among older age groups. UNESCO declared COVID-19 to be the most severe disruption to global education in history, estimating 1·6 billion learners in over 190 countries to be fully or partially out of school in 2020.^22^ With school closures and wider social restrictions in place, young people have been unable to come together in physical spaces, affecting their ability to learn and for peer interaction. Furthermore, young people are more likely to become unemployed during and following economic crises than older people.^23^

Our study is not the first to show how population shocks (ie, unexpected or unpredictable events that disrupt the environmental, health, economic, or social circumstances within a population) can increase the prevalence of depressive and anxiety disorders. In their review of mental health outcomes after the economic crisis in 2009, Frasquilho and colleagues^24^ identified several studies showing an increase in common mental disorders in the general population. After the 2009 financial crisis in Greece, point prevalence of major depressive episodes increased from 3·3% (95% UI 3·1–3·5) in 2008 to 6·8% (6·4–7·2) in 2009 and 8·2% (8·1–8·3) in 2011.^25^ Survey respondents reporting serious economic hardship were most at risk of developing a major depressive episode.^25^, ^26^ Similarly, after the 2008 financial crisis in Hong Kong, past-year prevalence of major depressive episodes increased from 8·2% (95% UI 7·2–9·2) in 2007 to 12·5% (11·0–13·9) in 2009.^27^ The extent of the increase in prevalence differed across studies, which might be due to study or population characteristics or different combinations of health and socioeconomic determinants of poor mental health, or a combination of these factors. Another point of difference is the time period over which the impact of the shock is measured. This period might be a single point in time, weeks or months, or, as in our analysis, an average over the course of a longer period (eg, a year) during which there would have been fluctuating effects from the pandemic (ie, the shock).

Both major depressive disorder and anxiety disorders increase the risk of other diseases and suicide.^28^, ^29^ In their time-series analysis of the impact of COVID-19 on suicide rates across 21 high-income and middle-income countries, Pirkis and colleagues^30^ found no significant increases in suicide rates between April and July, 2020. This finding raises the question of whether or not the increased prevalence of major depressive disorder and anxiety disorders we found was accompanied by a significant increase in suicide rates. We have insufficient data to draw any conclusions on this matter. Pirkis and colleagues^30^ relied on data in the first few months of the pandemic, which might have been too early in the pandemic to detect an association between a new diagnosis of major depressive disorder or anxiety disorders and suicide. Suicide trends might vary over extended periods, and as we progress through different phases of the pandemic the full scale of economic consequences and their effects will emerge, and their subsequent effects on suicide trends. For example, Tanaka and Okamota^31^ found that, although suicide rates in Japan decreased by 14% during the first 5 months of the pandemic (February to June, 2020), they then increased by 16% (between July and October, 2020), with a larger increase among females and younger populations than among males and older populations.

Even before the emergence of the COVID-19 pandemic, major depressive disorder and anxiety disorders (and mental disorders overall) were among the leading causes of health burden globally, with the mental health system in most countries being under-resourced and disorganised, despite evidence that effective prevention and intervention tools exist.^2^, ^3^, ^32^, ^33^ Meeting the added demand for mental health services due to COVID-19 will be difficult. Strategies to reduce the spread of SARS-CoV-2, such as physical distancing and restricted travel, have made it more difficult to acquire medication, attend treatment facilities, and receive in-person care. In some settings, outpatient and inpatient services have been interrupted or resources redirected to treat those with COVID-19.^4^, ^34^, ^35^, ^36^ In other settings, individuals have become less likely to seek care for their mental health issues than before the pandemic because of concerns about becoming infected with SARS-CoV-2 in the process.^4^, ^34^, ^35^, ^36^ The COVID-19 pandemic has created a greater urgency for governments and policy makers to strengthen their mental health systems, and now with the added priority of integrating a mental health response within their COVID-19 recovery plan.

In the wake of the impact of the COVID-19 pandemic on the prevalence and burden of major depressive disorder and anxiety disorders, taking no action cannot be an option. Resources exist to guide the development of mitigation strategies for reducing the mental health burden imposed by COVID-19. These resources include strategies that make efficient use of already limited resources, consider the local context and vulnerable populations, and prioritise key principles such as inclusivity, stigma reduction, and human rights.^4^, ^34^, ^35^, ^36^ Strategies should promote mental wellbeing and target determinants of poor mental health exacerbated by the pandemic and interventions to treat those who develop a mental disorder.^4^, ^34^, ^35^, ^36^ They should consider public health messaging about the mental health impacts of COVID-19, how individuals can best manage their mental health, and well defined pathways to assessment and service access. A mixture of digital, telehealth, and face-to-face services have been suggested that can be tailored to individual need.^4^, ^34^, ^35^, ^36^ There is already encouraging emerging evidence of the implementation of some of these strategies;^4^ however, the full effects of the COVID-19 pandemic are still unfolding in many countries, with most programmes implemented under a public health emergency, with little capacity to fully assess their performance.

Here, we estimated the change in disorder prevalence in 2020 using the best data at our disposal, but these data will continue to be updated throughout the different phases of the pandemic. This ongoing work will also explore ways to improve the following limitations in the analyses reported here. First, we were limited by the data available to consistently measure the effect of COVID-19 across all GBD locations using our choice of COVID-19 indicators. Human mobility and SARS-CoV-2 infection rates are unlikely to have captured the full impact of the pandemic on mental health across all countries. The precision in these two indicators can be improved, for example, in countries where infection rates are not consistently measured or reported. Reliance on mobile phone tracking technology to monitor human mobility will also not be accurate in locations where subgroups of the population (eg, people of low socioeconomic status) have little or no mobile phone use. Some subgroups might not be able to reduce their mobility because of their employment type and might be more prone to infection, and so reductions in human mobility might be exaggerated in these locations. As we progress through the pandemic and the full economic effects on some populations emerge, re-evaluation of available indicators will be important. For example, we made no distinction in prevalence between those with and without previous SARS-CoV-2 infection within the population. Emerging evidence suggests people with post-acute sequelae of COVID-19 (sometimes known as long COVID) might develop depressive and anxiety disorder symptoms,^37^, ^38^ and so prevalence of long COVID might be a potential indicator in future work. Second, we found very few surveys that met our inclusion criteria from low-income and middle-income countries, meaning that findings from our regression analysis might be less generalisable to these locations. For example, we estimated large increases in prevalence within Latin America and the Caribbean, north Africa and the Middle East, and south Asia, despite not finding any surveys from these super-regions that met our inclusion criteria. Given the absence of high quality data for most countries, and the wide UIs around our estimates, we emphasise caution against extrapolating direct rankings between countries and territories. Our leave-one-country-out cross-validation analysis showed that although our estimates are generally within the bounds of uncertainty for re-predicting data for missing locations, the relative rankings of locations were affected by data availability. Precise rankings between countries and territories would require substantially more high quality data and improved data coverage globally. Third, most surveys in our dataset used symptom scales that only estimate probable cases of depressive and anxiety disorders. Where these scales were used, we assumed the predictive validity of symptom scales in diagnosis via established thresholds remained constant between before and during the pandemic*.* However, this assumption has the potential to bias our estimates. For example, high scores on anxiety disorder symptom scales might reflect a natural psychological and physiological reaction to a perceived threat (ie, the COVID-19 pandemic) rather than a probable anxiety disorder. At the time of publication, not enough data were available to assess this assumption. We identified only three diagnostic mental health surveys that had been done since the beginning of the pandemic. Increases in prevalence were observed in two of the three diagnostic surveys.^39^, ^40^ The study that did not report an increased prevalence was conducted in one specific city in Norway (Trondheim) using very small cross-sectional samples.^41^ The authors of this study also reported that the shift from face-to-face to telephone survey administration occurred at the onset of the pandemic, which might have affected interviewers' ability to identify mental disorders, especially in the early stages of this shift when they were less experienced with telephone survey administration. The paucity of diagnostic surveys conducted during the pandemic also meant that we were unable to explore its effect on the severity distribution of major depressive disorder and anxiety disorders. We estimated prevalent cases of each disorder but could not assess how existing cases changed in their severity. Existing cases might have worsened during the COVID-19 pandemic, while novel cases might have had milder disorder severity. For the burden analysis presented, we had to assume that the severity distribution of both existing and new cases remained unchanged from before the COVID-19 pandemic. Fourth, many studies needed to be excluded from our systematic review because of reliance on convenience sampling strategies (eg, snowball sampling), case definitions that did not adhere to internationally accepted definitions for mental disorders, and use of survey instruments for which no comparable pre-pandemic estimate was available. Given the paucity of studies using random sampling of the general population, we took advantage of studies using market research and quota sampling during the COVID-19 pandemic. We found that cross-sectional (but not longitudinal) studies that used market research quota sampling significantly over-estimated the increase in disorder prevalence. Samples obtained via this method might be more prone to mental disorders than the random samples that informed their pre-pandemic baseline estimates. We also found instruments that captured symptoms of both depressive and anxiety disorders combined overestimated the increase in prevalence of anxiety disorders, but not major depressive disorder. This could be because they captured more new depressive disorder cases than anxiety disorder cases. We hope that the data standards set for this analysis will guide decisions in the field for future mental health surveys done as a response to COVID-19 or other population shocks. Fifth, prevalences of mental disorders other than major depressive disorder and anxiety disorders might have also been affected by COVID-19. For instance, emerging evidence suggests that other disorders such as eating disorders have been affected by the COVID-19 pandemic but these data have yet to be appropriately assessed.^42^ Most of the available scientific literature focuses on changes in symptoms of major depressive disorder and anxiety disorder as a result of COVID-19 because these are historically more sensitive to population shocks. As new mental health surveys are undertaken, the effect of COVID-19 on other disorders will need to be quantified. The methodological framework we have developed can be adapted to other mental disorders. It can also be adapted to measure other population shocks on prevalence and disease burden.

At the time of writing this Article, the COVID-19 pandemic is ongoing and its full impact on mental health outcomes is not known. We continue to observe shifts in SARS-CoV-2 infection rates and human mobility as lockdown and stay-at-home orders are re-implemented or eased and COVID-19 vaccination programmes are rolled out. Our work is ongoing and will continue to be updated over the course of the COVID-19 pandemic. To inform this ongoing work, high quality surveys conducted during the COVID-19 pandemic are needed within regions that are not represented by available data. Researchers planning surveys during the pandemic should strive to align their sample representation and measures of mental health with existing pre-pandemic baseline estimates to ensure appropriate comparable data from before and during the pandemic. Where feasible, researchers should consider including diagnostic measures of mental disorders, alongside widely used screening questionnaires (eg, Patient Health Questionnaire-9 or General Anxiety Disorder-7). With the addition of more estimates derived via diagnostic instruments, we could explore the robustness of (or correct for) our assumption that the predictive validity of screening questionnaires for diagnosis of probable cases is unchanged by the pandemic, and explore any shifts in the severity distribution among individuals diagnosed with major depressive disorder or anxiety disorders.

Unlike other population shocks, COVID-19 has become global, disrupting many aspects of life for most, if not all, of the world's populations. Our analysis suggests that the impacts on the prevalence and burden of major depressive disorder and anxiety disorders were substantial, particularly among females and younger populations. Ongoing and additional mental health surveys are necessary to quantify the duration and severity of this impact. Unfortunately, even before the emergence of the COVID-19 pandemic, major depressive disorder and anxiety disorders were leading causes of disease burden, with the mental health-care system in most countries being under-resourced and disorganised in their service delivery. Therefore, tackling this increased mental health burden will present immediate challenges in most nations, but it is also an opportunity for countries to broadly reconsider their mental health service response. Recommended mitigation strategies should incorporate ways to promote mental wellbeing and target determinants of poor mental health exacerbated by the pandemic, as well as interventions to treat those who develop a mental disorder. Taking no action in the face of the estimated impact of the COVID-19 pandemic on the prevalence and burden of major depressive disorder and anxiety disorders should not be an option.

## Data sharing

To download the code used in these analyses, please visit the GitHub page. Data sources are also listed by location and institution in the appendix (pp 33–34).

## Declaration of interests

C Adolph reports support from the Benifcus Foundation. A Flaxman holds stock in Agathos, and consults and advises Janssen, SwissRe, Sanofi, and Merck for Mothers on simulation modeling, outside of the submitted work. S Nomura reports support from the Ministry of Education, Culture, Sports, Science and Technology of Japan. All other authors declare no competing interests.
