# Supplementary material for: Global prevalence and burden of depressive and anxiety disorders in 204 countries and territories in 2020 due to the COVID-19 pandemic
Source: Lancet. 2021 Nov 6;398(10312):1700–12. doi: 10.1016/S0140-6736(21)02143-7 (PMC8500697; doi:10.1016/S0140-6736(21)02143-7)
Supplement: Supplementary appendix [file mmc1.pdf]

THE LANCET

Supplementary appendix

This appendix formed part of the original submission and has been peer reviewed. We post it as supplied by the authors.

Supplement to: COVID-19 Mental Disorders Collaborators. Global prevalence and burden of depressive and anxiety disorders in 204 countries and territories in 2020 due to the COVID-19 pandemic. *Lancet* 2021; published online Oct 8. http://dx.doi.org/10.1016/S0140-6736(21)02143-7.

Appendix: Supplementary methods and results to "Estimating the global prevalence and burden of depressive and anxiety disorders in 2020 due to the COVID-19 pandemic"

This appendix provides supplemental methods, figures and more detailed results for "Estimating the global prevalence and burden of depressive and anxiety disorders in 2020 due to the COVID-19 pandemic."

Table of Contents

List of figures and tables 3
Figures 3
Tables 3
Section 1. Statement of GATHER compliance 4
Section 2. Search strategy for data sources reporting the prevalence of depressive and anxiety disorders during the COVID-19 pandemic 5
Section 2.1. Pubmed 5
Section 2.2. MedRXiv 5
Section 2.3. PsyArXiv 5
Section 2.4. COVID-19 databases 5
Section 2.5. Google Scholar 5
Section 3. Supplementary methods for the development of the model to predict change in prevalence of depressive and anxiety disorders 6
Section 3.1. Case definition 6
Section 3.2. Data preparation and model specifications 6
Section 3.3. The inclusion and utility of market research / quota sampling studies 7
Section 4. Symptom scales vs diagnostic instruments 8
Section 5. Selection of COVID-19 impact indicators 10
Section 6. Leave-one-country-out cross-validation analysis 11
Section 7. References 12
Section 8. Figures and tables 17
Figure S1. Graphical summary of process to estimate prevalence of major depressive disorder and anxiety disorders due to the COVID-19 pandemic 17
Figure S2. Applied example (females aged 20-24 in France) of prevalence adjustment for every day of the year 2020. 18
Figure S3. PRISMA flowchart for systematic review of depressive and anxiety disorder prevalence estimates during the COVID-19 pandemic 19
Figure S4: Number of studies by country informing the meta-regression to estimate change in depressive and anxiety disorder prevalence during the COVID-19 pandemic in 2020 20
Figure S5: Prevalence of major depressive disorder per 100 000 persons due to the COVID-19 pandemic, 2020 21
Figure S6: Prevalence of anxiety disorders per 100 000 persons due to the COVID-19 pandemic, 2020 22
Figure S7. The distribution of untransformed estimated daily COVID-19 infection rates vs square root and logarithmic transformations 23

Figure S8. The distribution of untransformed estimated daily excess mortality rates vs square root and logarithmic transformations 23
Figure S9. Scatterplot of observed difference in logit prevalence vs predicted difference in logit prevalence from the leave-one-country-out cross-validation analysis for major depressive disorder 24
Figure S10. Scatterplot of observed difference in logit prevalence vs predicted difference in logit prevalence from the leave-one-country-out cross-validation analysis for anxiety disorders 25
Table S1: Guidelines for Accurate and Transparent Health Estimates Reporting (GATHER) checklist . 26
Table S2: PRISMA 2020 checklist 28
Table S3: Severity proportions and disability weights for major depressive disorder and anxiety disorders in GBD 2020 32
Table S4: Bias covariates considered in the meta-regression of change in prevalence 32
Table S5: Characteristics of studies and data included in the analysis 33
Table S6: Prevalence and DALYs of MDD per 100 000 persons, with 95% uncertainty intervals, by location, for the year 2020 35
Table S7: Prevalence and DALYs of MDD in 1000s, with 95% uncertainty intervals, by location, for the year 2020 43
Table S8: Prevalence and DALYs of Anxiety disorders per 100 000 persons, with 95% uncertainty intervals, by location, for the year 2020 51
Table S9: Prevalence and DALYs of anxiety disorders in 1000s, with 95% uncertainty intervals, by location, for the year 2020 59
Table S10: Meta-regression coefficients from initial and final prevalence-adjustment models for MDD and anxiety disorders 67
Table S11: Leave-one-country-out cross-validation analysis results 68
Section 9. Authors' contributions 80
Section 9.1. Managing the estimation or publications process 80
Section 9.2. Writing the first draft of the manuscript 80
Section 9.3. Primary responsibility for applying analytical methods to produce estimates 80
Section 9.4. Primary responsibility for seeking, cataloguing, extracting, or cleaning data; designing or coding figures and tables 80
Section 9.5. Providing data or critical feedback on data sources 80
Section 9.5. Developing methods or computational machinery 80
Section 9.6. Providing critical feedback on methods or results 80
Section 9.7. Drafting the work or revising is critically for important intellectual content 81
Section 9.7. Managing the overall research enterprise 81

List of figures and tables

Figures

Figure S1. Graphical summary of process to estimate prevalence of major depressive disorder and anxiety disorders due to the COVID-19 pandemic

Figure S2. Applied example of prevalence adjustment for every day of the year 2020. Example is females aged 20–24 in Spain

Figure S3. PRISMA flowchart for systematic review of depressive and anxiety disorder prevalence estimates during the COVID-19 pandemic

Figure S4: Number of studies by country informing the meta-regression to estimate change in depressive and anxiety disorder prevalence during the COVID-19 pandemic in 2020

Figure S5: Prevalence of major depressive disorder per 100 000 persons due to the COVID-19 pandemic, 2020

Figure S6: Prevalence of anxiety disorders per 100 000 persons due to the COVID-19 pandemic, 2020

Figure S7. The distribution of untransformed estimated daily COVID-19 infection rates vs square root and logarithmic transformations

Figure S8. The distribution of untransformed estimated daily excess mortality rates vs square root and logarithmic transformations

Figure S9. Scatterplot of observed difference in logit prevalence vs predicted difference in logit prevalence from the leave-one-country-out cross-validation analysis for major depressive disorder

Figure S10. Scatterplot of observed difference in logit prevalence vs predicted difference in logit prevalence from the leave-one-country-out cross-validation analysis for anxiety disorders

Tables

Table S1: Guidelines for Accurate and Transparent Health Estimates Reporting (GATHER) checklist

Table S2: PRISMA 2020 checklist

Table S3: Severity proportions and disability weights for major depressive disorder and anxiety disorders in GBD 2020

Table S4: Bias covariates considered in the meta-regression of change in prevalence

Table S5: Characteristics of studies and data included in the analysis

Table S6: Prevalence and DALYs of MDD per 100 000 persons, with 95% uncertainty intervals, by location, for the year 2020

Table S7: Prevalence and DALYs of MDD in 1000s, with 95% uncertainty intervals, by location, for the year 2020

Table S8: Prevalence and DALYs of Anxiety disorders per 100 000 persons, with 95% uncertainty intervals, by location, for the year 2020

Table S9: Prevalence and DALYs of anxiety disorders in 1000s, with 95% uncertainty intervals, by location, for the year 2020

Table S10: Meta-regression coefficients from initial and final prevalence-adjustment models for MDD and anxiety disorders

Table S11: Leave-one-country-out cross-validation analysis results

Section 1. Statement of GATHER compliance

This study complies with the Guidelines for Accurate and Transparent Health Estimates Reporting (GATHER) recommendations (Table S1, pp 26-27).

Section 2. Search strategy for data sources reporting the prevalence of depressive and anxiety disorders during the COVID-19 pandemic

Section 2.1. Pubmed

("Mental health"[Title/Abstract] OR "Mental disorders"[Title/Abstract] OR "Anxiety Disorders"[MeSH Terms] OR "Depressive Disorder"[MeSH Major Topic] OR "anxiety"[Title/Abstract] OR "depress*"[Title/Abstract] OR "Mental disorder"[Title/Abstract] OR "psycholog*"[Title/Abstract]) AND ("novel coronavirus"[Title/Abstract] OR "COVID"[Title/Abstract] OR "covid-19"[Title/Abstract] OR "covid-19"[Title/Abstract] OR "covid19"[Title/Abstract] OR "nCoV"[Title/Abstract] OR "novel CoV"[Title/Abstract] OR "2019nCoV"[Title/Abstract] OR "Coronavirus"[Title/Abstract] OR "coronavi*"[Title/Abstract] OR "SARS-COV-2"[Title/Abstract] OR "SARSCoV2"[Title/Abstract] OR "SARS CoV2"[Title/Abstract] OR "outbreak"[Title/Abstract] OR "epidemic"[Title/Abstract] OR "pandemic"[Title/Abstract] OR "Coronavirus"[MeSH Terms] OR "covid-19"[Supplementary Concept] OR "Pandemics"[MeSH Terms] OR "severe acute respiratory syndrome coronavirus 2"[Supplementary Concept] OR "covid-19"[Supplementary Concept]) AND (("prevalen*"[Title/Abstract] OR "Prevalence"[MeSH Terms] OR "impact"[Title/Abstract] OR "effect"[Title/Abstract] OR "outcome"[Title/Abstract] OR "percentage"[Title/Abstract]) Filters: Publication date from 2020/01/01

Section 2.2. MedRXiv

"mental health" "mental disorder" anxiety depression depressive psychological" (match whole any)
Full text or abstract or title in MedRxiv only posted between "01 Jan, 2020 and 31 Dec, 2020"

Section 2.3. PsyArXiv

("novel coronavirus" OR "COVID" OR "covid 19" OR covid-19 OR "covid19" OR "nCoV" OR "novel CoV" OR "2019nCoV" OR "Coronavirus" OR Coronavi* OR SARS-COV-2 OR "SARSCoV2" OR "outbreak" OR "epidemic" OR "pandemic") AND ("mental health" OR "mental disorder" OR anxiety OR depression OR depress* OR psychology*) AND (prevalen* OR impact OR effect OR outcome OR percentage)

Section 2.4. COVID-19 databases

A number of COVID-19 databases were searched on a regular basis using relevant filters. The databases and filters used include:

- COVID-19: living map of the evidence by Eppi-centre: Studies included in the database were filtered by "mental health impacts".
- The DEPRESSD Project**:** References identified from research question 2 "Factors Associated with Levels or Changes in Symptoms".
- The Neurology and Neuropsychiatry of COVID-19 Blog: Results in this database were filtered by the groups “DATA PAPERS: Psychiatric”, “REVIEWS, EDITORIALS AND POSITION PAPERS: Systematic reviews/meta-analyses”, and “REVIEWS, EDITORIALS AND POSITION PAPERS: Psychiatry”
- WHO-COVID-19: Results in this database were filtered by the topic "mental health”.
- COVID-minds: Lists studies currently in the field which assess the impact of COVID on mental health. Studies were reviewed on a regular basis to check if results are available.
- Researchgate: Studies within the COVID group were filtered by psychological impact.

Section 2.5. Google Scholar

Google Scholar was used to source grey literature and baseline data (i.e., pre-covid data). For grey literature, a combination of keywords were used in English and Spanish including : ("mental health" OR "anxiety" OR "depression") AND "covid-19" (i.e., Spanish equivalent (“salud mental” O “ansiedad” O “depression”) Y “covid-19”).

For baseline data, a combination of location, parameter, disorder, mental health instrument, and or sample type-specific keywords were used, restricting publication year from 2013 to 2020. For example, the search for baseline data on depression estimates measured with the Patient Health Questionnaire (PHQ-9) in Ireland included:

- Location-specific terms: (“Ireland”, OR “Irish”), AND
- Parameter-specific terms: “prevalence”, AND
- Disorder-specific terms: (“depression” OR “depressed” OR “anxiety”), AND
- Instrument-specific terms: “PHQ”, AND
- Sample type: (“representative” OR "survey")

Section 3. Supplementary methods for the development of the model to predict change in prevalence of depressive and anxiety disorders

Section 3.1. Case definition

To ensure comparability in measurement, we followed case definitions for major depressive disorder (MDD) and anxiety disorders used within the Global Burden of Diseases, Injuries, and Risk Factors Study (GBD). These definitions adhere to criteria presented in Diagnostic and Statistical Manual of Mental Disorders (DSM-IV-TR) and the International Classification of Diseases and Related Health Problems (ICD-10).[1,2] In summary, MDD involves the presence of at least one major depressive episode, which is the experience of either depressed mood or loss of interest/pleasure, for most of every day, for at least two weeks (DSM-IV-TR: 296.21–24, 296.31–34; ICD-10: F32.0–9, F33.0–9). Anxiety disorders involves experiences of intense fear and distress, typically in combination with other physiological symptoms. In GBD, anxiety disorders are modelled as a single cause for “any” anxiety disorder to avoid the double-counting of individuals meeting criteria for more than one anxiety disorder (DSM-IV-TR: 300.0-300.3, 208.3, 309.21, 309.81; ICD-10: F40-42, F43.0, F43.1, F93.0-93.2, F93.8)

Section 3.2. Data preparation and model specifications

We developed models to estimate the change in prevalence of major depressive disorder (MDD) and anxiety disorders with the intention to use this model to adjust the prevalences of MDD and anxiety disorder estimated by DisMod-MR 2.1 (which represent the pre-pandemic prevalences). Models were run separately for MDD and anxiety disorders. We conducted meta-regressions via meta-regression: Bayesian, regularised, trimmed[3] (MR-BRT) on the difference between the logit disorder prevalence during the COVID-19 pandemic and prior to the pandemic. The difference in logit prevalence was chosen to ensure any adjusted prevalence estimate would be restricted between 0 and 1.

Three COVID-19 impact indicators were tested as independent variables: Human mobility, daily COVID-19 infection rate, and daily excess mortality rate. For each COVID-19 impact indicator, the average daily estimate between the start date of the survey minus the recall period of the assessment tool and the end date of the survey was calculated and assigned to each estimate informing the model. For example, Peters and colleagues[4] used the PHQ-9 to assess prevalence in Germany between April 16, 2020, and May 29, 2020. The PHQ-9 has a recall of 14 days, and so the average daily estimate of the COVID-19 impact indicators in Germany between April 2, 2020, and May 29, 2020, was calculated and assigned to this estimate. COVID-19 daily infections and excess mortality were extremely positively skewed, and so we corrected this via a square-root transformation. This transformation was also important for the extrapolation of prevalence change along these impact indicators as a adjustments along a severely skewed index would have led to undesirable outliers post-estimation. We also tested a logarithmic transformation but this resulted in a substantial negative skew and so was not considered further (Figure S7 & Figure S8).

Potential bias covariates flagged cross-sectional comparisons informed by random samples, longitudinal comparisons informed by market research / quota samples, cross-sectional comparisons informed by market research / quota samples, and estimates representing combined symptoms of depressive and anxiety disorders (eg, K6[5]) (see Table S4 for reasons for potential bias). As only three studies used diagnostic instruments to measure prevalence, we did not have sufficient data to explore the impact of a bias covariate on data from screening scales identifying probable cases of depressive or anxiety disorders (Appendix, Section 4). Age, sex, and most bias covariates were also included as effect modifiers to ensure that the prevalence change remained zero when the COVID-19 impact was zero. The exception was the bias covariate for cross-sectional comparisons informed by market research / quota samples, which had random sample pre-pandemic baselines and therefore had a prevalence difference even when the impact indicator was zero.

Sex was quantified by the percentage female of the sample and was centered at 50% female. Age was quantified as the mean age of the sample (where available) or the midpoint of the age range of the study sample (when mean-age was not available) and was mean-centered at 49·1 years. Where reported, the most detailed data by age and sex were extracted and included in the model. For studies that reported age-specific and sex-specific prevalence, but not age-sex-specific prevalence,

we conducted a standard GBD data preparation process called "age and sex splitting". This process involves estimating the within-study sex ratio and adjusting the age-specific data accordingly to estimate age-sex-specific estimates from the study.

Each unique sample was given a random intercept and random effects were placed on the COVID-19 impact indicators. Models were run separately for MDD and anxiety disorders. Disorder-specific estimates were included in their respective models, and estimates representing combined depressive and anxiety disorder symptoms were included in both models, with their respective bias covariate. We specified 5% data trimming to robustify the models against outliers. Trimming within MR-BRT seeks to fit a specified majority of the most self-coherent data, giving an understanding of the overall relationship in the face of outlying observations. MR-BRT classifies observations into (majority) inliers and (minority) outliers, while simultaneously fitting the model with respect to which this classification is made.[3,6]

Due to the strong collinearity between the COVID-19 impact indicators, and the need for age, sex, and bias covariates to be treated as effect modifiers on the impact indicators, we developed prevalence adjustment models via a two-step process. First, we conducted indicator models to develop an index for the COVID-19 impact based on the COVID-19 impact indicators. Human mobility, daily COVID-19 infection rate, and daily excess mortality rate were included in a meta-regression on the change in logit prevalence to quantify their independent impact on the prevalence change. Following this, the coefficients from these models were used to calculate a single COVID-19 impact indicator for each disorder. Then a final model for each disorder was developed via backward elimination to regress this indicator, age, sex, and the bias covariates on the change in logit prevalence. The least significant covariate was iteratively removed until no improvement was seen in the Akaike information criterion. Initial and final model results are presented in Table S10. We also conducted a leave-one-country-out cross-validation analysis to explore the generalizability of the model to locations missing data detailed in Section 6.

Section 3.3. The inclusion and utility of market research / quota sampling studies

The inclusion of market research / quota samples was necessary in the development of our prevalence-adjustment models due to the very limited data available derived from random samples. In total, 66% of data sources informing the final MDD model (n = 27) and 52% of the data sources informing the final anxiety disorders model (n = 13) were from market research / quota samples and so the available data would be substantially limited if these estimates were excluded. These estimates also increased geographical coverage of the data, adding six additional countries for MDD (Japan, Czechia, Austria, Denmark, France, and Ireland), and four additional countries for anxiety disorders (USA, Japan, Czechia, and France). We did not observe a significant bias in the longitudinal market research / quota samples. Bias was only observed in cross-sectional comparisons between market research / quota samples and a pre-COVID-19 random sample (Table S10). This suggests a baseline bias in market research / quota samples and not a bias in prevalence change within these samples. We believe including a covariate for cross-sectional market research / quota samples was the best approach to accommodate this bias, and increase data richness and geographical coverage.

Section 4. Symptom scales vs diagnostic instruments

Our prevalence-adjustment regression model was primarily informed by studies measuring change in probable depressive or anxiety disorders using symptom scales. In an ideal scenario, we would have sourced enough estimates using diagnostic instruments to diagnose cases of MDD and anxiety disorders to adequately capture any systematic differences between change in prevalence of MDD and anxiety disorders vs change in prevalence of probable depressive or anxiety disorders via a covariate. Unfortunately, our systematic review only revealed three studies meeting our inclusion criteria that used diagnostic instruments to diagnose cases of MDD or anxiety disorders. This meant it was not feasible to include a covariate to differentiate between diagnostic instruments and symptom scales. This was especially challenging given the change in prevalence was also expected to vary by age, sex, bias covariates, and the impact of COVID-19.

In The GBD Study, symptom scales are used as a proxy for prevalence of MDD. For these symptom scale estimates, a bias correction is conducted to adjust these estimates to the level they would have been had the study used a diagnostic instrument. This bias correction represents the predictive validity of these symptom scales to MDD prevalence.

For the current analysis, we assumed that the assumption that the predictive validity (and in turn, the bias correction) of these symptom scales to MDD and anxiety disorders remained the same between pre-pandemic and mid-pandemic. If this assumption is true, then the change in probable depressive or anxiety disorder prevalence is equal to the change in MDD and anxiety disorder prevalence. For example, we can assume that disorder prevalence is a function of probable disorder prevalence from a symptom scale and the predictive validity of the symptom scale:

$$D = P \times V$$

where *D* represents the disorder prevalence, *P* represents the probable disorder prevalence from the symptom scale, and *V* represents the predictive validity of that symptom scale. If *V* is constant, then the change in prevalence is equal between probable disorder prevalence from the symptom scale and disorder prevalence from the diagnostic instrument:

$$Change = \frac{P_{pandemic} \times V}{P_{baseline} \times V} = \frac{P_{pandemic}}{P_{baseline}} = \frac{D_{pandemic}}{D_{baseline}}$$

However, it is possible that the assumption that *V* remains unchanged between pre-pandemic and mid-pandemic does not always hold true. For example, high scores on anxiety disorder symptom scales may reflect a natural psychological reaction to a perceived threat (i.e., the COVID-19 pandemic) rather than a probable anxiety disorder. At the time of publication, there was not enough data available to evaluate this.

Of the three studies using diagnostic measures, two showed substantial increases in disorder prevalence. Ayuso-Mateos et al[7] reported a mid-pandemic MDD point prevalence of 9.8% (*SE* = 1.2%) vs a pre-pandemic 1-year recall prevalence of 7.8% (*SE* = 0.8%) using the CIDI within a longitudinal random sample. After adjusting the pre-pandemic estimate to reflect point-prevalence using the MDD year-to-point recall adjustment of 2.0 (*SE* = 1.0) used in for GBD 2020, the estimated pre-pandemic point prevalence of MDD was 3.9% (*SE* = 2.0%, up 50.5%). Winkler and colleagues[8] reported a MDD prevalence of 11.8% (*SE* = 0.6%) during the COVID-19 pandemic vs a pre-pandemic estimate of 4.0% (*SE* = 0.3%, up 197.2%). For anxiety disorders, they reported a prevalence of 12.8% (*SE* = 0.6%) vs a pre-pandemic estimate of 7.8% (SE = 0.4%, up 64.8%). However these estimates were within a market research / quota sample and likely over-estimated the increase in prevalence.

The third study conducted by Knudsen and colleagues[9] reported prevalence of MDD and anxiety disorders using the CIDI on random samples in Norway across three time points, March to May 2020, June to July 2020, and August to September 2020. Compared to their pre-pandemic baseline MDD prevalence of 2.7% (*SE* = 0.7%), they reported MDD prevalences of 1.7% (*SE* = 0.5%, down 37.0%), 1.3% (*SE* = 0.5%, down 51.9%), and 4.6% (*SE* = 1.1%, up 70.3%) across the three time points respectively. Compared to their pre-pandemic baseline anxiety disorder prevalence of 10.4% (*SE* = 1.4%), they reported anxiety disorder prevalences of 6.1% (*SE* = 0.9%, down 41.3%), 8.2% (*SE* = 0.9%, down 21.2%), and 7.8% (*SE* = 1.4%, down 25.0%) across the three time points respectively. There are three limitations to this study that warrants mention. First, the samples at baseline and each time point are very small, ranging between 372 and 691. Second, the authors also reported that the shift from face-to-face to telephone survey administration occurred at the onset of the pandemic, which may have

impacted on interviewers' ability to identify mental disorders, especially in the early stages of this shift when they were less experienced with telephone survey administration. Third, the baseline estimates from this study may have been abnormally elevated. A recent analysis of GP visits in Norway by Hvide and Johnsen showed abnormally high GP visits related to mental health in January and February of 2020 compared to previous years.[10] Hvide and Johnsen suggest this may have been due to abnormally poor weather, with January being the wettest January ever recorded in Norway.[11] Additionally, a discussion of this finding with Norwegian GBD collaborators raised the fact that a prominent figure in the Norwegian community passed away end of December 2019 and the circumstances were related to mental health. National coverage of this death may have impacted the mental health of the community.[12] This has yet to be substantiated with epidemiological data.

Section 5. Selection of COVID-19 impact indicators

We required indicators of the impact of the COVID-19 pandemic that had an association with depressive and anxiety disorder prevalence, referred to hereafter as COVID-19 impact indicators. The risk factor of interest was the COVID-19 pandemic, with the COVID-19 impact indicators acting as proxies for the impact of COVID-19 in the population. A COVID-19 impact indicator had to: a) capture an impact of COVID-19, b) be consistently measured across countries, and c) be consistently measured with sufficient granularity across time (preferably daily or weekly), over the course of the pandemic. We considered two potential indicators relevant to past shocks (eg, epidemics, financial crises): change in unemployment status and real gross domestic product (GDP);[13-17] and three novel indicators unique to the COVID-19 pandemic but without established relationships with mental health: human mobility,[18] estimated total (as opposed to reported) COVID-19 daily infection rate,[19] and estimated daily excess mortality rate during the pandemic (including excess deaths not reported as due to COVID-19).[20]

Whilst there is clear evidence on the relationship between change in unemployment status or GDP and mental health, these two indicators may not best reflect the unprecedented scale and breadth of the COVID-19 response. They failed to capture the mitigating impact of government involvement and unrelated changes in individual behaviour due to the pandemic (eg, lockdown and social distancing). Furthermore, these indicators lacked consistency across countries and temporal granularity across the pandemic (eg, GDP often reported quarterly). The daily excess mortality rate and infection rate were considered direct measures of the course of the COVID-19 pandemic. These were chosen over reported infections and deaths to reported infections and deaths as better proxies of the overall impact of the pandemic. We did not intend for the change in prevalence of MDD and anxiety disorders to be reactive to reported numbers. The prevalence change was expected to be due to the overall impact of the pandemic on the population, with estimated infections and excess mortality as proxies to this impact. We suspect using reported estimates would likely underestimate the prevalence change for locations where official reporting of cases and deaths have not been adequate.

However, these indicators hide the impacts in countries that have lower case-fatality rates due to health systems in place, or implemented strong lockdown mandates stopping transmission and mortality but still impacting wellbeing in the community and the local economy. Human mobility potentially reflects impacts of the COVID-19 pandemic manifested through reductions in movement due to 1) lockdown mandates, 2) social withdrawal from fear or anxiety within communities, especially where community infection is rampant, and 3) economic factors such as unemployment and reduced business activity.

The estimation of human mobility, COVID-19 daily infection rate, and daily excess mortality rate was conducted by the IHME COVID-19 Forecasting Team and is described in detail elsewhere.[18] In summary, human mobility was represented by a composite human mobility index representing daily change from pre-pandemic mobility. Data from mobile phone users provided by Facebook, Google, Descartes Labs, Safegraph, and Baidu, and data on social distancing mandates informed a Gaussian process regression to estimate a time series for human mobility. COVID-19 daily infection and excess mortality rates were estimated via a deterministic SEIR (susceptible, exposed, infectious, and recovered) compartmental framework and was informed by daily confirmed COVID-19 cases, deaths, tests conducted, antibody seroprevalence, human mobility, social distancing mandates, pneumonia seasonality, mask use, and vaccine coverage.[19,20]

Section 6. Leave-one-country-out cross-validation analysis

We conducted a leave-one-country-out cross-validation (LOCOCV) analysis to evaluate the generalisability of our prevalence-adjustment models towards countries not informing our models. For each country, we re-ran our MDD and anxiety disorder prevalence-adjustment models without data for that country. We then used these models to predict the change in logit prevalence (the dependent variable of the models) for the missing data, and crosschecked whether the data fell within the bounds of prediction intervals of our models. We also calculated the country-level root mean squared error (RMSE) and compared this against a benchmark model without the COVID-19 impact indicator index or its effect modifiers. Instead, the benchmark model contained a non-zero intercept, linear time trend between baseline and mid-pandemic estimate, and bias covariates flagged as significant in the original prevalence-adjustment models. In the absence of the indicators in the benchmark models, bias covariates were included as dichotomous variables.

We also compared the mean country-level RMSE between the prevalence-adjustment model and the benchmark model. However, USA and UK contributed a substantial amount of data for both MDD and anxiety disorders prevalence-adjustment models. Removing the data from these countries resulted in a substantially reduced dataset and therefore these countries were a priori expected to perform poorly in the LOCOCV. For MDD, the USA contributed 22% of estimates and 29% of data sources, and the UK contributed 22% of estimates and 21% of data sources. Likewise for anxiety disorders, the USA contributed 23% of estimates and 27% of data sources, and the UK contributed 21% of estimates and 15% of data sources. We therefore also compared the country-level RMSE between the prevalence-adjustment model and the benchmark model after excluding the RMSE for USA and UK.

For MDD, only three of the 209 estimates informing the model were outside of the prediction intervals (Table S11). Two estimates were cross-sectional market research / quota sampling studies with small samples (n ~ 1000) from Denmark, and the third was a very small sample (n < 500) from UK. A scatter between observed estimates against estimates predicted by the LOCOCV analysis is presented in Figure S9. The RMSE of the MDD prevalence-adjustment model was superior to the benchmark model for all countries with the exception of Austria, Czechia, New Zealand, UK, and USA. USA and UK were again a priori expected to perform poorly. Twelve of the 14 estimates from Czechia (*M* = -1.17), Austria (*M* = -1.31), and New Zealand (*M* = -1.16) were derived from cross-sectional market research / quota samples and these estimates were very close to the mean for studies flagged on this bias covariate (*M* = -1.18), which is likely why a non-zero intercept model with binary bias covariates performed well for these locations. The mean country-level RMSE was 0.53 for the prevalence-adjustment model compared 0.55 for the benchmark model. However, the mean country-level RMSE was 0.50 for the prevalence-adjustment model and 0.54 for the benchmark model after excluding the RMSE for USA and UK.

All 141 estimates informing the anxiety disorders model fell within the prediction intervals generated as part of the LOCOCV analysis. A scatter between observed estimates against estimates predicted by the LOCOCV analysis is presented in Figure S10. The RMSE of the anxiety prevalence-adjustment model was superior to the benchmark model for all countries with the exception of Australia, New Zealand, UK, and USA. Again, USA and UK were a priori expected to perform poorly due to large reduction in estimates informing the prevalence-adjustment model when data from these countries were removed. The six estimates from Australia represented change in combined depressive and anxiety disorder symptoms derived from random samples, and the average of these estimates (*M* = -0.31) was very close to the average of estimates of this type (*M* = -0.33). For new Zealand, seven of the nine estimates represented change in combined depressive and anxiety disorder symptoms derived from cross-sectional market-research / quota samples and their average (*M* = -1.16) was very close to the average of these types of estimates (*M* = -1.21). This again was likely why a non-zero intercept model with binary bias covariates performed well for these locations. Again, the mean country-level RMSE for the prevalence-adjustment model was 0.54 compared to 0.61 for the benchmark model. After excluding the USA and the UK, the mean country-level RMSE was 0.48 for the prevalence-adjustment model and 0.62 for the benchmark model.

Section 7. References

References

1. American Psychiatric Association. Diagnostic and statistical manual of mental disorders : DSM-IV-TR. 4th ed., text revision. ed. Washington, DC: American Psychiatric Association; 2000.
2. World Health Organization. The ICD-10 classification of mental and behavioural disorders : clinical descriptions and diagnostic guidelines. Geneva: World Health Organization; 1992.
3. Zheng P, Barber R, Sorensen RJD, Murray CJL, Aravkin AY. Trimmed Constrained Mixed Effects Models: Formulations and Algorithms. *Journal of Computational and Graphical Statistics* 2021: 1-13.
4. Peters A, Rospleszcz S, Greiser KH, Dallavalle M, Berger K. The Impact of the COVID-19 Pandemic on Self-Reported Health. *Deutsches Arzteblatt international* 2020; **117**(50): 861-7.
5. Kessler RC, Barker PR, Colpe LJ, et al. Screening for Serious Mental Illness in the General Population. *Archives of General Psychiatry* 2003; **60**(2): 184-9.
6. Aravkin A, Davis D. Trimmed Statistical Estimation via Variance Reduction. *Mathematics of Operations Research* 2019; **45**(1): 292-322.
7. Ayuso-Mateos JL, Morillo D, Haro JM, Olaya B, Lara E, Miret M. Changes in depression and suicidal ideation under severe lockdown restrictions during the first wave of the COVID-19 pandemic in Spain: a longitudinal study in the general population. *Epidemiology and Psychiatric Sciences* 2021; **30**(e49).
8. Winkler P, Formanek T, Mlada K, et al. Increase in prevalence of current mental disorders in the context of COVID-19: analysis of repeated nationwide cross-sectional surveys. *Epidemiol Psychiatr Sci* 2020; **29**: e173.
9. Knudsen AKS, Stene-Larsen K, Gustavson K, et al. Prevalence of mental disorders, suicidal ideation and suicides in the general population before and during the COVID-19 pandemic in Norway: A population-based repeated cross-sectional analysis. *The Lancet Regional Health - Europe* 2021; **4**.
10. Hvide H, Johnsen JV. COVID-19 and mental health: A longitudinal population study through 2020. 2021. https://voxeu.org/article/covid-19-and-mental-health-evidence-norway (accessed 12th August 2021).
11. Meteorologisk institutt. Januar 2020 ble den våteste noen gang. 2020. https://kommunikasjon.ntb.no/pressemelding/januar-2020-ble-den-vateste-noen-gang?publisherId=17846853&releaseId=17878908 (accessed 12th August 2021).
12. Niederkrotenthaler T, Braun M, Pirkis J, et al. Association between suicide reporting in the media and suicide: systematic review and meta-analysis. *BMJ* 2020; **368**: m575.
13. Christodoulou NG, Christodoulou GN. Financial Crises: Impact on Mental Health and Suggested Responses. *Psychotherapy and Psychosomatics* 2013; **82**(5): 279-84.
14. Frasquilho D, Matos MG, Salonna F, et al. Mental health outcomes in times of economic recession: a systematic literature review. *BMC Public Health* 2016; **16**(1): 115.
15. Marazziti D, Avella MT, Mucci N, et al. Impact of economic crisis on mental health: a 10-year challenge. *CNS Spectr* 2021; **26**(1): 7-13.
16. World Health Organization. Impact of economic crises on mental health. Geneva: World Health Organization, 2011.
17. Jahoda M. Economic Recession and Mental Health: Some Conceptual Issues. *Journal of Social Issues* 1988; **44**(4): 13-23.

18. IHME COVID-19 Forecasting Team. Modeling COVID-19 scenarios for the United States. *Nat Med* 2021; **27**(1): 94-105.
19. [COVID-19 Cumulative Infection Collaborators]. Estimating global, regional and national daily and cumulative infections with COVID-19: a modelling study. *The Lancet* In prep.
20. [COVID-19 Excess Mortality Collaborators]. Estimating the total number of deaths due to SARS-CoV-2 infection: a systematic analysis of total COVID-19 mortality between January 2020 and August 2021. *The Lancet* In Prep.
21. Biddle N, Edwards B, Gray M, Sollis K. Hardship, distress, and resilience: The initial impacts of COVID-19 in Australia: Australian National University. Centre for Social Research and Methods, 2020.
22. Bulbulia JA, Piven SD, Barlow FK, et al. National Longitudinal Mediators of Psychological Distress During Stringent COVID-19 Lockdown. *medRxiv* 2020: 2020.09.15.20194829.
23. Daly M, Sutin A, Robinson E. Longitudinal changes in mental health and the COVID-19 pandemic: Evidence from the UK Household Longitudinal Study. *Psychological Medicine* 2020; **13**: 1-10.
24. Katz B, Yovel I. Mood Symptoms PredictCOVID-19 Pandemic Distress but not Vice Versa: An 18-Month Longitudinal Study. *PsyArXiv* 2020.
25. Kikuchi H, Machida M, Nakamura I, et al. Changes in Psychological Distress during the COVID-19 Pandemic in Japan: a Longitudinal Study. *J Epidemiol* 2020; **30(11)**: 522-8.
26. Kwong ASF, Pearson RM, Adams MJ, et al. Mental health before and during the COVID-19 pandemic in two longitudinal UK population cohorts. *Br J Psychiatry* 2020; **24**: 1-10.
27. Marroquin B, Vine V, Morgan R. Mental health during the COVID-19 pandemic: Effects of stay-at-home policies, social distancing behavior, and social resources. *Psychiatry Res* 2020; **293**: 113419.
28. van der Velden PG, Contino C, Das M, van Loon P, Bosmans MWG. Anxiety and depression symptoms, and lack of emotional support among the general population before and during the COVID-19 pandemic. A prospective national study on prevalence and risk factors. *Journal of Affective Disorders* 2020; **277**: 540-8.
29. Vizard T, Davis J, White E, Beynon B. Coronavirus and depression in adults, Great Britain: June 2020. 2020. https://www.ons.gov.uk/peoplepopulationandcommunity/wellbeing/articles/coronavirusanddepressioninadultsgreatbritain/june2020#measuring-the-data.
30. Wanberg CR, Csillag B, Douglass RP, Zhou L, Pollard MS. Socioeconomic status and well-being during COVID-19: A resource-based examination. *J Appl Psychol* 2020; **105**(12): 1382-96.
31. Widnall E, Winstone L, Mars B, Haworth C, Kidger J. Young People's Mental Health during the COVID-19 Pandemic: Initial findings from a secondary school survey study in South West England: NIHR School for Public Health Research, 2020.
32. Zhang L, Zhang D, Fang J, Wan Y, Tao F, Sun YJJNO. Assessment of Mental Health of Chinese Primary School Students Before and After School Closing and Opening During the COVID-19 Pandemic. *JAMA Netw Open* 2020; **3**(9): e2021482.
33. Australian Bureau of Statistics. Household Impacts of COVID-19 Survey, 2020.
34. Australian Bureau of Statistics. Australia National Health Survey 2017-2018.
35. Bryan C, Bryan AO, Baker JC. Associations among state-level physical distancing measures and suicidal thoughts and behaviors among U.S. adults during the early COVID-19 pandemic. *Suicide Life Threat Behav* 2020; **50**(6): 1223-9.

36. National Center for Health Statistics. Centers for Disease Control and Prevention. United States National Health and Nutrition Examination Survey 2017-2018.
37. Choi EPH, Hui BPH, Wan EYF. Depression and anxiety in Hong Kong during COVID-19. *International journal of environmental research public health* 2020; **17**(10): 3740.
38. Ni MY, Li TK, Pang H, et al. Direct participation in and indirect exposure to the Occupy Central Movement and depressive symptoms: a longitudinal study of Hong Kong adults. *American journal of epidemiology* 2016; **184**(9): 636-43.
39. Lau JTF, Kim Y, Wu AMS, Wang Z, Huang B, Mo PKH. The Occupy Central (Umbrella) movement and mental health distress in the Hong Kong general public: political movements and concerns as potential structural risk factors of population mental health. *Soc Psychiatry Psychiatr Epidemiol* 2017; **52**(5): 525-36.
40. Daly M, MacLachlan M, Maguire R, et al. Changes in PTSD, depression, and generalized anxiety before and during the COVID-19 pandemic in the Republic of Ireland. *Journal of Affective Disorders Reports* 2020; **5**(100184).
41. Central Statistics Office (Ireland). Ireland Health Survey 2015. Dublin, Ireland.
42. Ettman CK, Abdalla SM, Cohen GH, Sampson L, Vivier PM, Galea S. Prevalence of Depression Symptoms in US Adults Before and During the COVID-19 Pandemic. *JAMA Network Open* 2020; **3**(9): e2019686-e.
43. Every-Palmer S, Jenkins M, Gendall P, et al. Psychological distress, anxiety, family violence, suicidality, and wellbeing in New Zealand during the COVID-19 lockdown: A cross-sectional study. *PLOS ONE* 2020; **15**(11): e0241658.
44. Ministry of Health (New Zealand). New Zealand Health Survey 2019-2020.
45. Fukase Y, Ichikura K, Murase H, Tagaya H. Depression, risk factors, and coping strategies in the context of social dislocations resulting from the second wave of COVID-19 in Japan. *BMC psychiatry* 2021; **21**(1): 33.
46. Ito R SM, Sato K, Kato M, Fujisawa D, Naito A, Morita T, Miyashita M. The nature of QOL and its related factors measured by the question items of the medical treatment behavior survey for the general public in Japan. *Palliative Care Research* 2020; **15(2)**.
47. Groarke JM, Berry E, Graham-Wisener L, McKenna-Plumley PE, McGlinchey E, Armour C. Loneliness in the UK during the COVID-19 pandemic: Cross-sectional results from the COVID-19 Psychological Wellbeing Study. *PLoS One* 2020; **15**(9): e0239698.
48. Wilson JM, Lee J, Shook NJ. COVID-19 worries and mental health: the moderating effect of age. *Aging & mental health* 2020: 1-8.
49. Kantor B, Kantor J. Mental health outcomes and associations during the coronavirus disease 2019 pandemic: A cross-sectional survey of the US general population. *Front Psychiatry* 2020; **11**: 569083.
50. Terlizzi EP, MA V. Symptoms of Generalized Anxiety Disorder Among Adults: United States, 2019. NCHS Data Brief, no 378. Hyattsville, MD: National Center for Health Statistics. 2020, 2019.
51. Killgore WDS, Cloonan SA, Taylor EC, Miller MA, Dailey NS. Three months of loneliness during the COVID-19 lockdown. *Psychiatry Res* 2020; **293**: 113392.
52. Kiuchi K, Kishi K, Araki K. A Foundational Assessment of the Effects of the Spread of COVID-19 Virus Infection and Related Activity Restrictions on Mental and Physical Health, Psychological Distress, and Suicidal Ideation in Japan. *Asia Pac J Public Health* 2020; **32**(8): 463-6.
53. Ministry of Health, Labour and Welfare (Japan). Japan Comprehensive Survey of Living Conditions. In: Ministry of Health, Labour and Welfare (Japan), editors.; 2019.

54. McGinty EE, Presskreischer R, Han H, Barry CL. Psychological Distress and Loneliness Reported by US Adults in 2018 and April 2020. *Jama* 2020; **324**(1): 93-4.
55. McGinty EE, Presskreischer R, Anderson KE, Han H, Barry CL. Psychological Distress and COVID-19–Related Stressors Reported in a Longitudinal Cohort of US Adults in April and July 2020. *Jama* 2020.
56. Maxfield M, Pituch KA. COVID-19 worry mental health indicators and preparedness for future care needs across the adult lifespan. *Aging & mental health* 2020: 1-8.
57. O'Connor RC, Wetherall K, Cleare S, et al. Mental health and wellbeing during the COVID-19 pandemic: longitudinal analyses of adults in the UK COVID-19 Mental Health & Wellbeing study. *The British Journal of Psychiatry* 2020: 1-17.
58. Peretti-Watel P, Alleaume C, Leger D, Beck F, Verger P, Group C. Anxiety, depression and sleep problems: a second wave of COVID-19. *Gen Psychiatr* 2020; **33**(5): e100299.
59. Institute for Research and Documentation in Health Economics (IRDES). France Survey of Health and Welfare 2014, 2014.
60. Christoph P, Sanja B, Elke H, Thomas P. Comparing Mental Health during COVID-19 Lockdown and 6 Months Later in Austria: A Longitudinal Study. *Front Psychiatry* 2021; **12**: 625973.
61. Central Statistics Office. Austria Health Survey 2018-2019.
62. Pieh C, Budimir S, Probst T. The effect of age gender income work and physical activity on mental health during coronavirus disease (COVID-19) lockdown in Austria. *J Psychosom Res* 2020; **136**(110186).
63. Pieh C, Budimir S, Delgadillo J, Barkham M, Fontaine JRJ, Probst T. Mental health during COVID-19 lockdown in the United Kingdom. *Psychosomatic Medicine* 2021; **83**(4): 328-37.
64. Sønderskov KM, Dinesen PT, Santini ZI, Østergaard SD. Increased psychological well-being after the apex of the COVID-19 pandemic. *Acta Neuropsychiatrica* 2020; **32**(5): 277-9.
65. Sønderskov KM, Dinesen PT, Santini ZI, Østergaard SD. The depressive state of Denmark during the COVID-19 pandemic. *Acta Neuropsychiatr* 2020; **32**(4): 226-8.
66. Santé publique France. Covid-19: une enquête pour suivre l'évolution des comportements et de la santé mentale pendant l'épidémie. 22 juin 2020. 2020.
67. Shevlin M, McBride O, Murphy J, et al. Anxiety, depression, traumatic stress and COVID-19-related anxiety in the UK general population during the COVID-19 pandemic. *BJPsych open* 2020; **6**(6): e125.
68. Sibley CG, Greaves LM, Satherley N, et al. Effects of the COVID-19 pandemic and nationwide lockdown on trust, attitudes toward government, and well-being. *American Psychologist* 2020; **75**(5): 618-30.
69. Twenge JM, Joiner TE. Mental distress among U.S. adults during the COVID-19 pandemic. *J Clin Psychol* 2020; **76**(12): 2170-82.
70. Ravens-Sieberer U, Kaman A, Erhart M, Devine J, Schlack R, Christiane O. Impact of the COVID-19 pandemic on quality of life and mental health in children and adolescents in Germany. *Eur Child Adolesc Psychiatry* 2021.
71. Ueda M, Stickley A, Sueki H, Matsubayashi T. Mental Health Status of the General Population in Japan during the COVID-19 Pandemic. *Psychiatry and Clinical Neurosciences* 2020; **74**(9): 505-6.
72. Valiente C, Vázquez C, Peinado V, Contreras A, Trucharte A. Estudio nacional representativo de las respuestas de los ciudadanos de España ante la crisis de Covid-19: respuestas psicológicas. 2020.

73. Ministry of Health. Social Services and Equality. National Statistics Institute (Spain). Spain European Health Survey 2014. Madrid, Spain: National Statistics Institute
74. Vieira JB, Pierzchajlo S, Jangard S, Marsh A, Olsson A. Perceived threat and acute anxiety predict increased everyday altruism during the COVID-19 pandemic: PsyArXiv, 2020.
75. Scholten S, Velten J, Margraf J. Mental distress and perceived wealth, justice and freedom across eight countries: The invisible power of the macrosystem. *PloS one* 2018; **13**(5): e0194642.
76. Yamamoto T, Uchiumi C, Suzuki N, Yoshimoto J, Murillo-Rodriguez E. The Psychological Impact of 'Mild Lockdown' in Japan during the COVID-19 Pandemic: A Nationwide Survey under a Declared State of Emergency. *International journal of environmental research and public health* 2020; **17**(24).
77. Zhou Y, MacGeorge EL, Myrick JG. Mental Health and Its Predictors during the Early Months of the COVID-19 Pandemic Experience in the United States. *International journal of environmental research and public health* 2020; **17**(17): 6315.

Section 8. Figures and tables

Figure S1. Graphical summary of process to estimate prevalence of major depressive disorder and anxiety disorders due to the COVID-19 pandemic

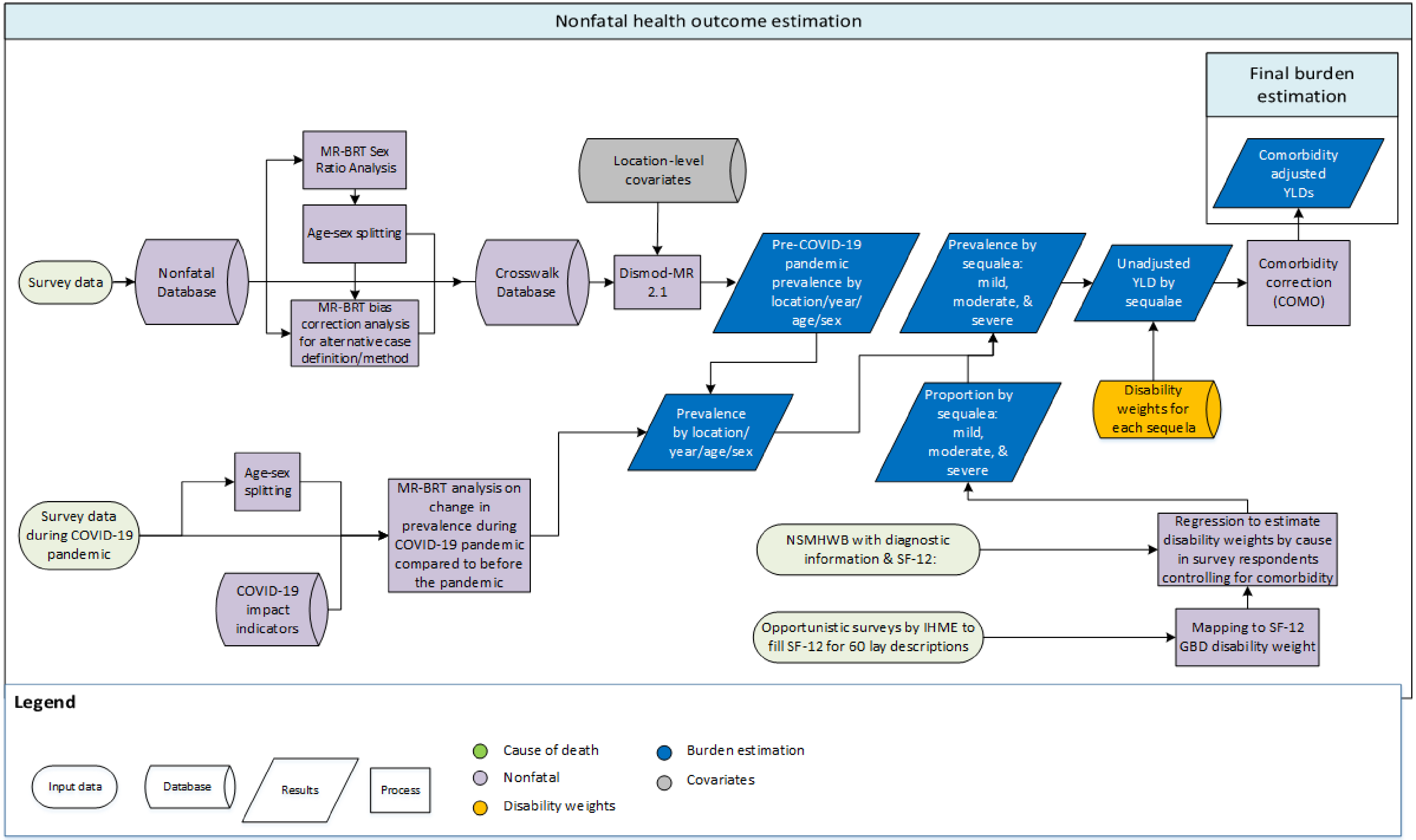

MR-BRT=meta-regression—Bayesian, regularised, trimmed. IHME=Institute for Health Metrics and Evaluation. NSMWHB=Australian National Survey of Mental Health and Wellbeing. GBD=Global Burden of Diseases, Injuries, and Risk Factors Study. YLDs=years lived with disability.

Figure S2. Applied example (females aged 20-24 in France) of prevalence adjustment for every day of the year 2020.

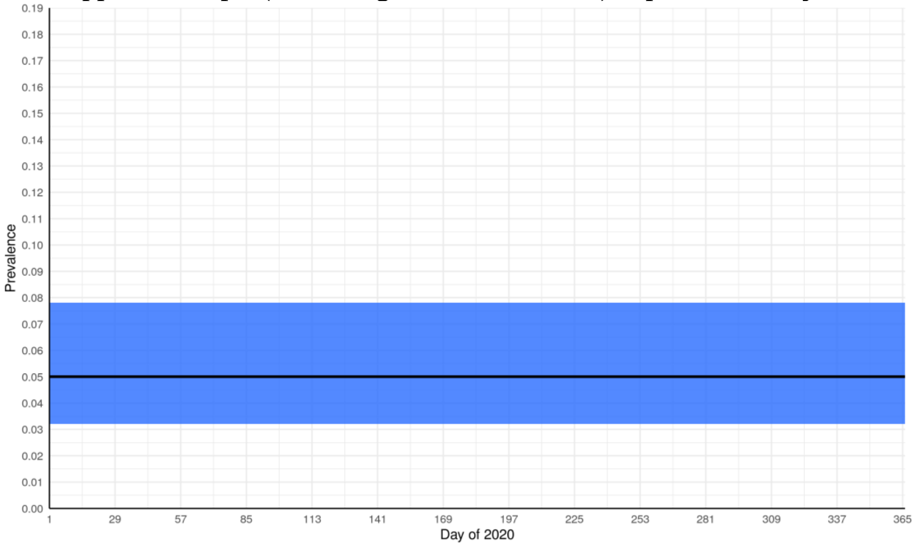

Step 1: Assign pre-COVID-19 prevalence from DisMod-MR 2.1 for every day of the year 2020

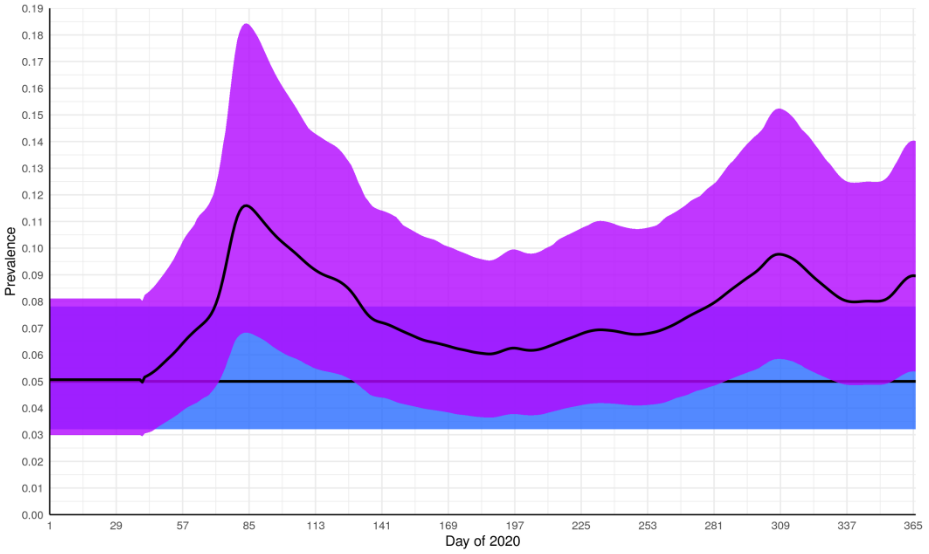

Step 2: Adjust daily prevalence by daily estimates of COVID-19 impact indicators

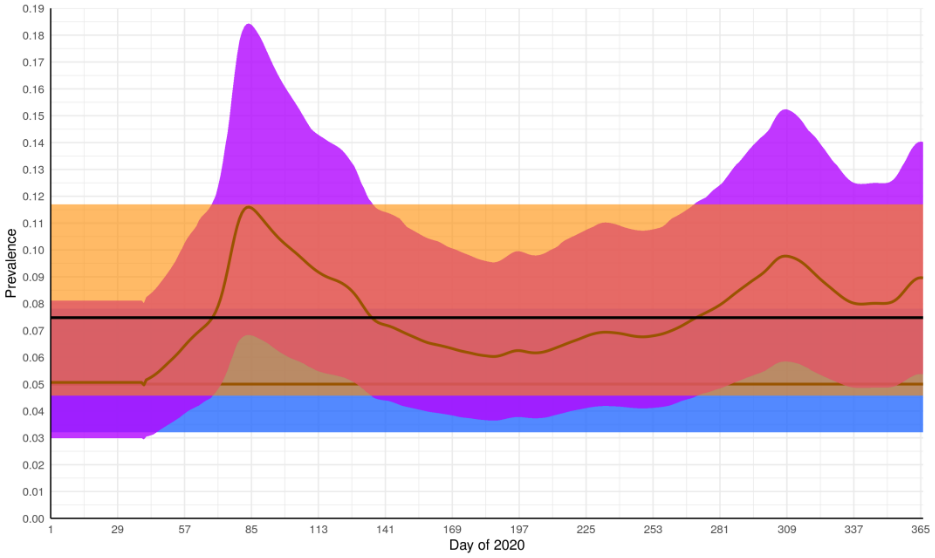

Step 3: Calculate average daily prevalence for the year 2020 as the point prevalence for the year

Solid lines represent prevalence estimates and colour fill represents 95% uncertainty intervals. Blue = Pre-COVID-19 prevalence. Purple = Daily adjusted prevalence. Orange = Prevalence adjusted for COVID-19 pandemic.

Figure S3. PRISMA flowchart for systematic review of depressive and anxiety disorder prevalence estimates during the COVID-19 pandemic

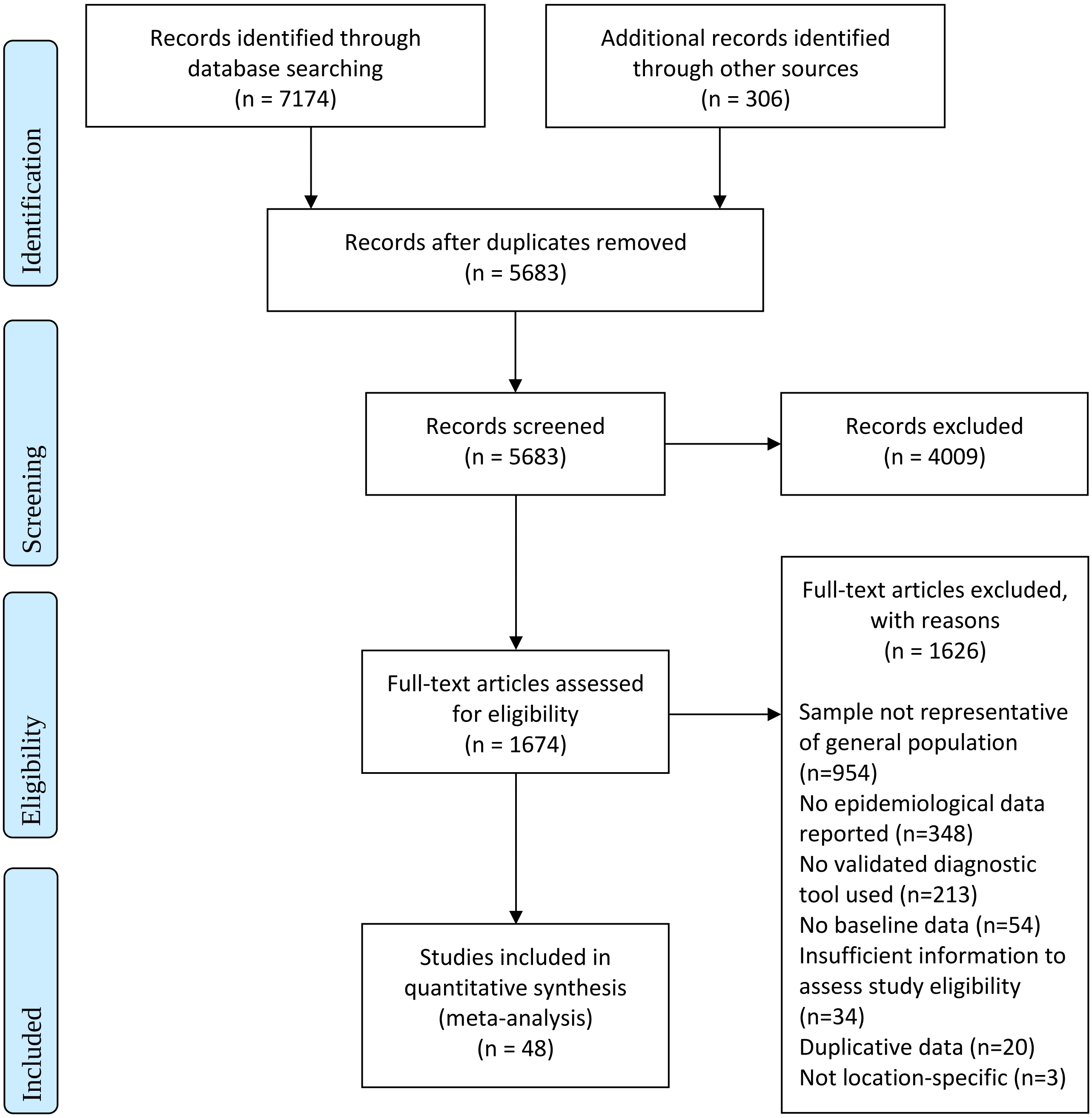

From: Moher D, Liberati A, Tetzlaff J, Altman DG, The PRISMA Group (2009). *P*referred *R*eporting *I*tems for *S*ystematic Reviews and *M*eta-*A*nalyses: The PRISMA Statement. PLoS Med 6(7): e1000097. doi:10.1371/journal.pmed1000097

For more information, visit www.prisma-statement.org.

Figure S4: Number of studies by country informing the meta-regression to estimate change in depressive and anxiety disorder prevalence during the COVID-19 pandemic in 2020

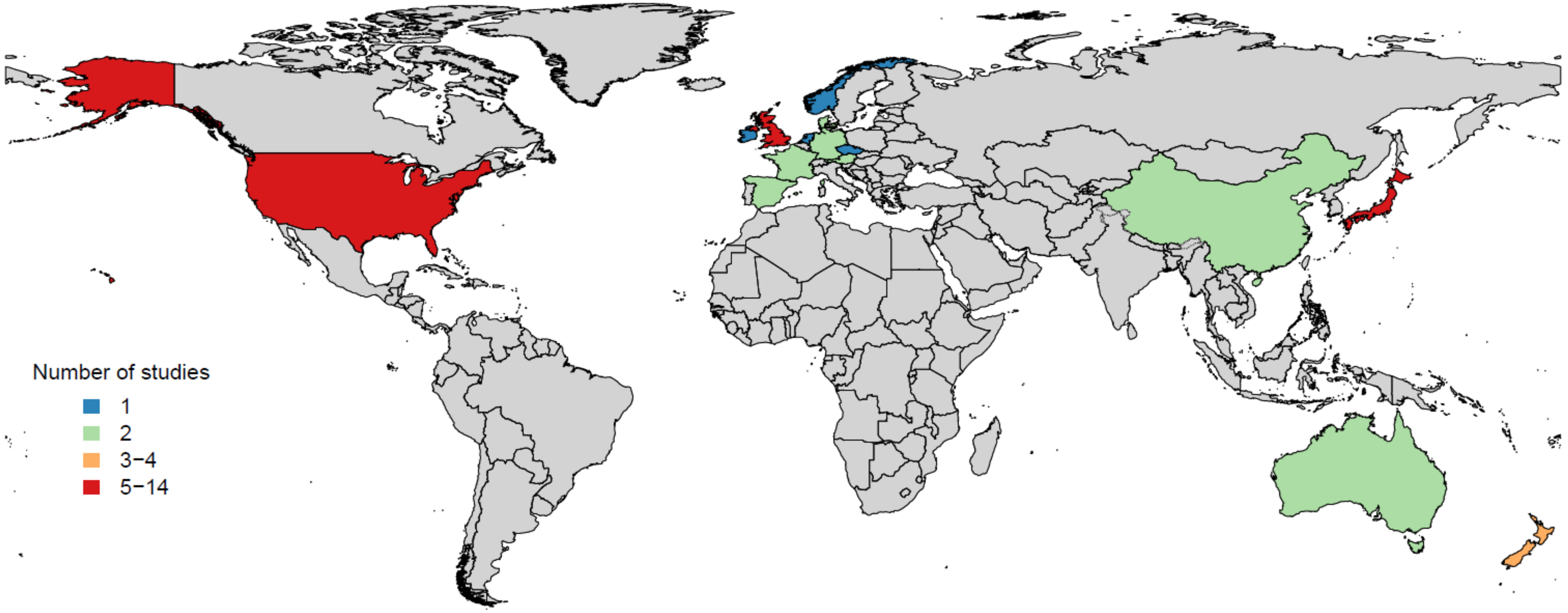

Figure S5: Prevalence of major depressive disorder per 100 000 persons due to the COVID-19 pandemic, 2020

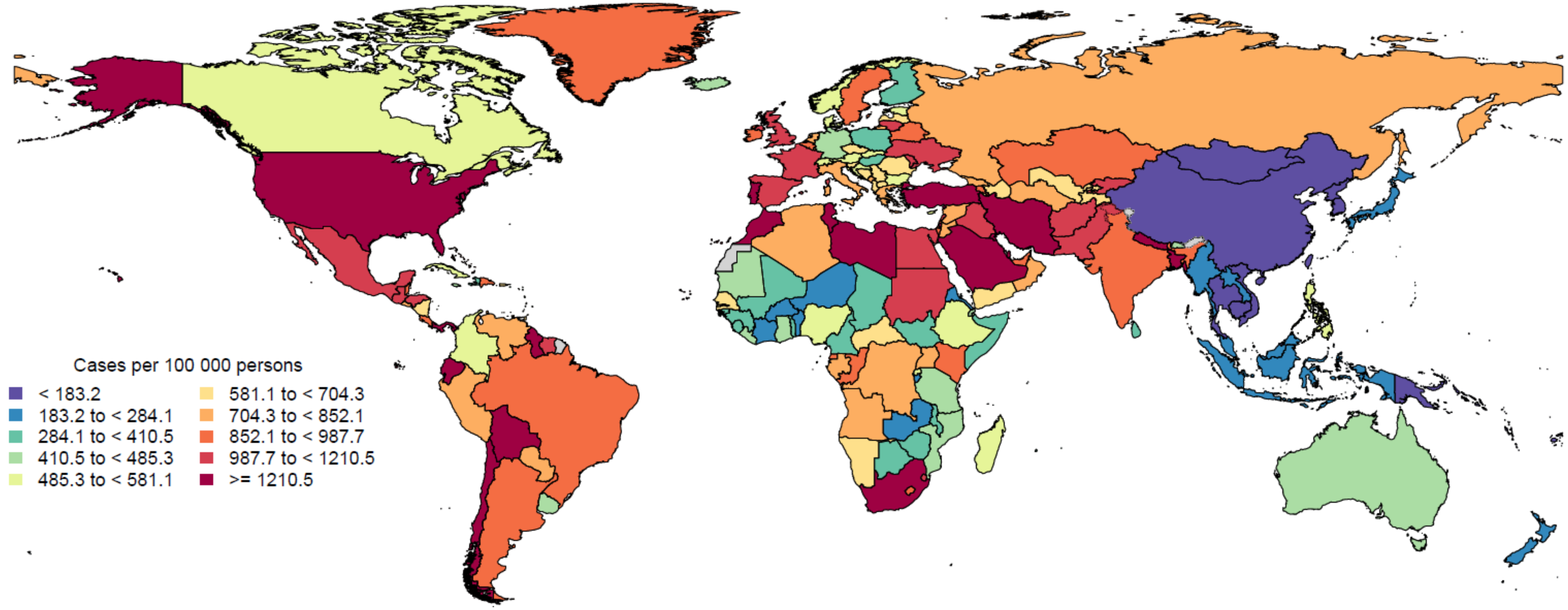

Figure S6: Prevalence of anxiety disorders per 100 000 persons due to the COVID-19 pandemic, 2020

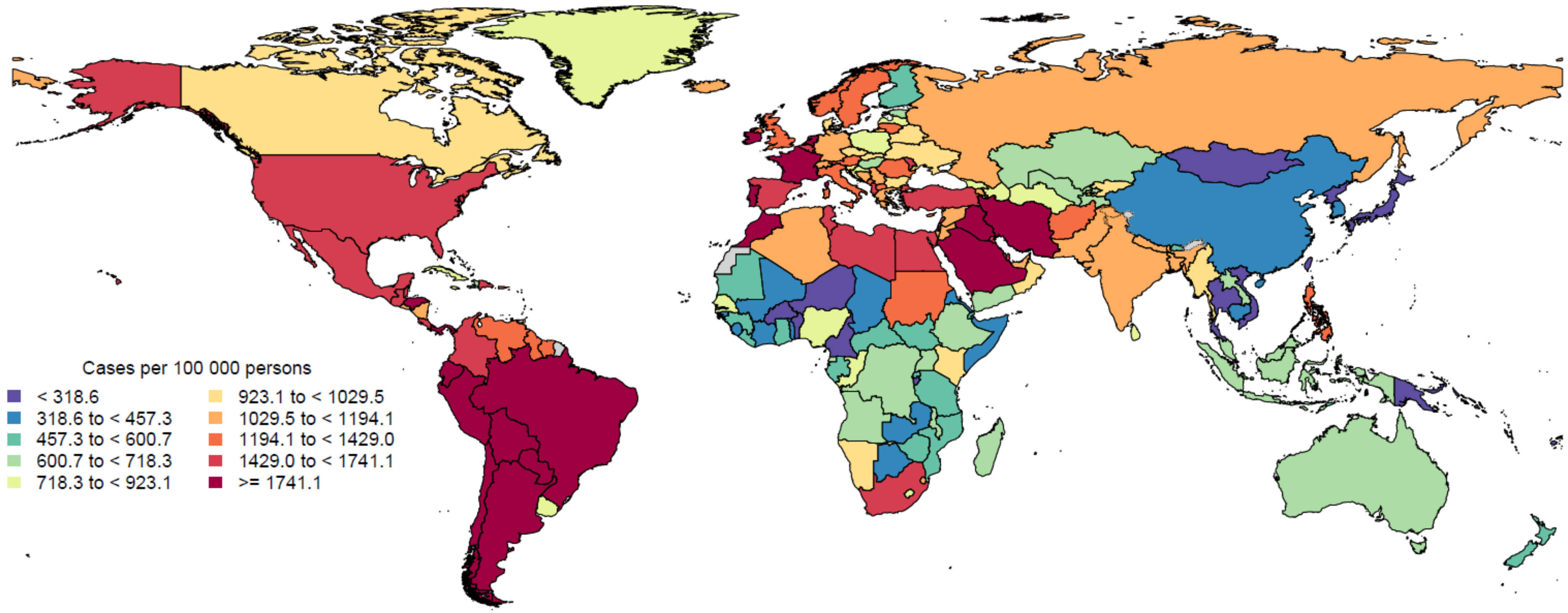

Figure S7. The distribution of untransformed estimated daily COVID-19 infection rates vs square root and logarithmic transformations

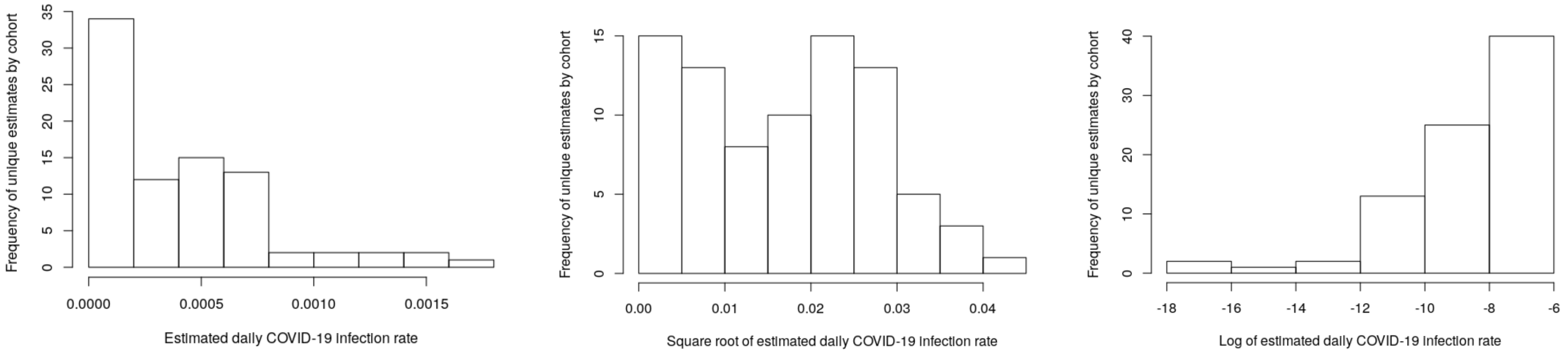

Figure S8. The distribution of untransformed estimated daily excess mortality rates vs square root and logarithmic transformations

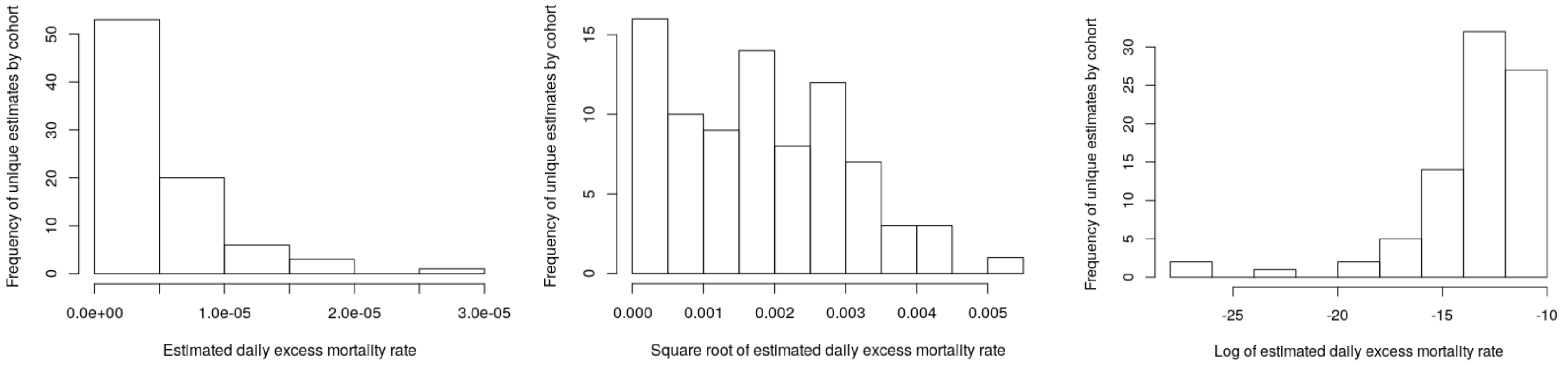

Figure S9. Scatterplot of observed difference in logit prevalence vs predicted difference in logit prevalence from the leave-one-country-out cross-validation analysis for major depressive disorder

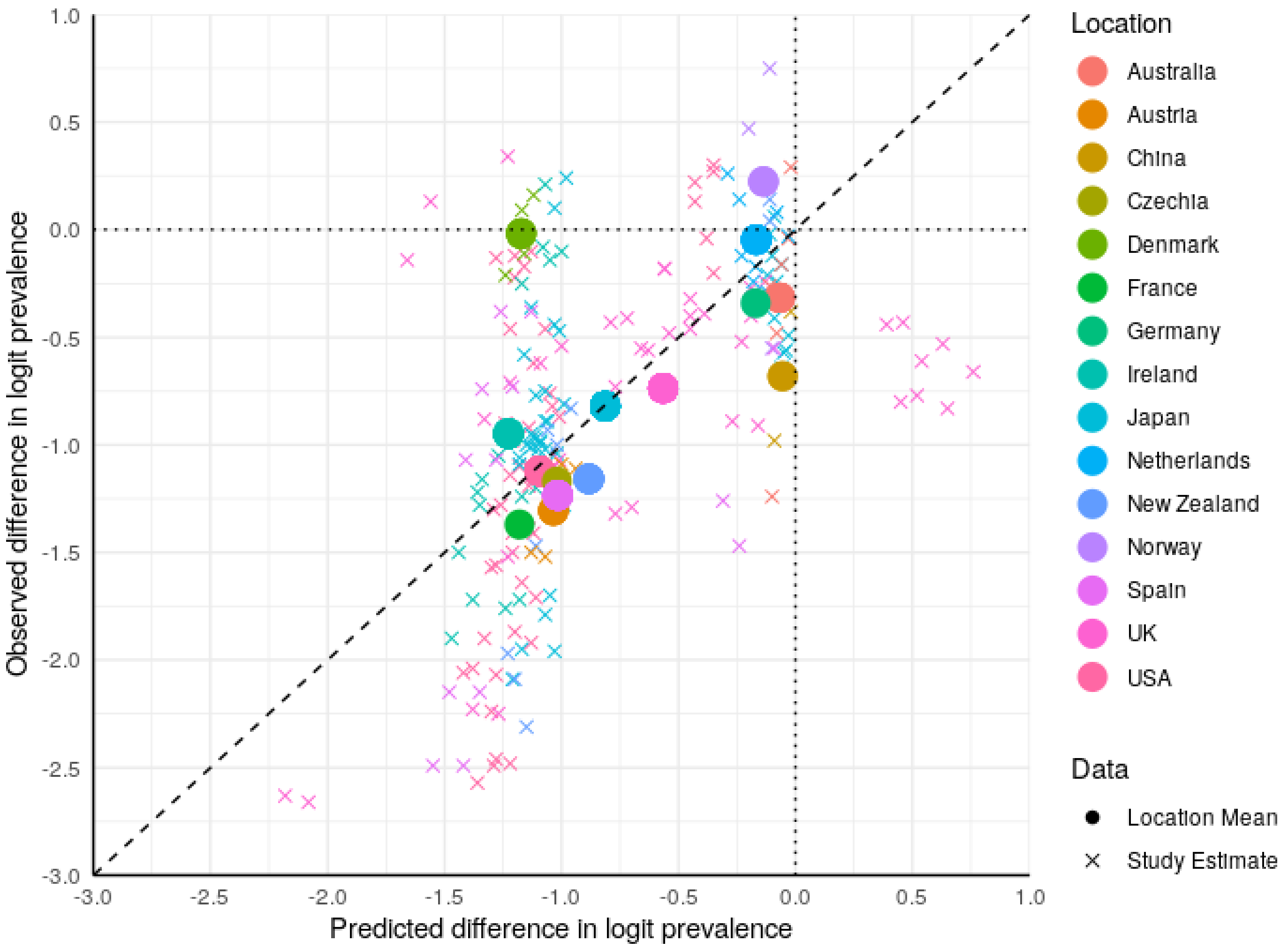

Figure S10. Scatterplot of observed difference in logit prevalence vs predicted difference in logit prevalence from the leave-one-country-out cross-validation analysis for anxiety disorders

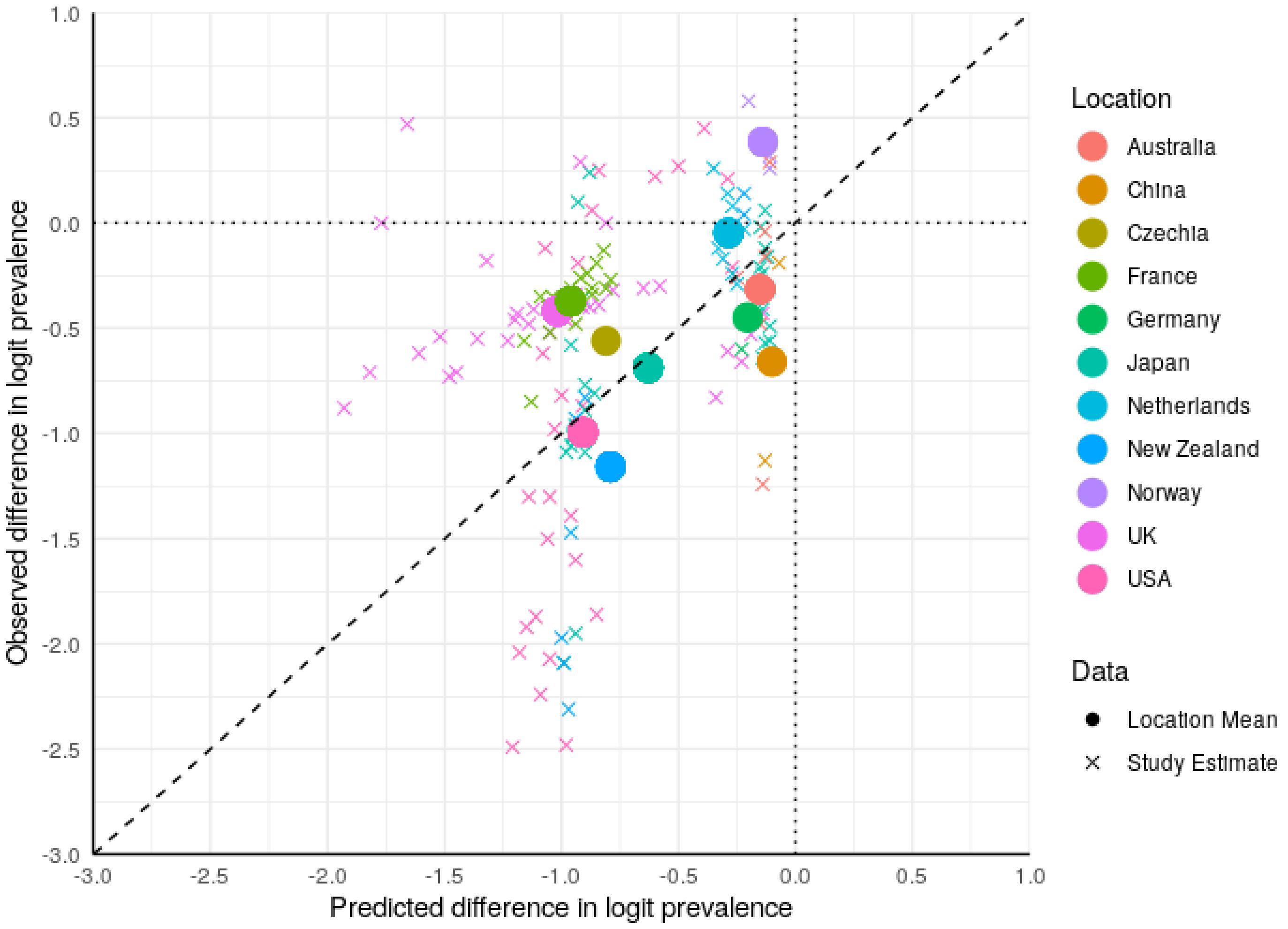

Table S1: Guidelines for Accurate and Transparent Health Estimates Reporting (GATHER) checklist

Item #	Checklist item	Location
**Objectives and funding**		
1	Define the indicator(s), populations (including age, sex, and geographic entities), and time period(s) for which estimates were made.	Introduction p 6
2	List the funding sources for the work.	Acknowledgments p 24
**Data Inputs**		
For all data inputs from multiple sources that are synthesized as part of the study:		
3	Describe how the data were identified and how the data were accessed.	Methods: Data sources pp 8-11
4	Specify the inclusion and exclusion criteria. Identify all ad-hoc exclusions.	Methods: Data sources pp 8-9
5	Provide information on all included data sources and their main characteristics. For each data source used, report reference information or contact name/institution, population represented, data collection method, year(s) of data collection, sex and age range, diagnostic criteria or measurement method, and sample size, as relevant.	Table S5 Appendix pp 33-34
6	Identify and describe any categories of input data that have potentially important biases (e.g., based on characteristics listed in item 5).	Methods: Data sources pp 8-9
For data inputs that contribute to the analysis but were not synthesized as part of the study:		
7	Describe and give sources for any other data inputs.	Methods: Data sources pp 9-11
For all data inputs:		
8	Provide all data inputs in a file format from which data can be efficiently extracted (e.g., a spreadsheet rather than a PDF), including all relevant meta-data listed in item 5. For any data inputs that cannot be shared because of ethical or legal reasons, such as third-party ownership, provide a contact name or the name of the institution that retains the right to the data.	*Datasets will be available for download via Global Health Data Exchange website (http://ghdx.healthdata.org/)*<br>*Data prepped for analysis is available at https://github.com/ihmeuw/mental_disorders/tree/COVID-19_depressive_anxiety_disorders_lancet*
**Data analysis**		
9	Provide a conceptual overview of the data analysis method. A diagram may be helpful.	Methods: *Overview* p 7<br>Figure S1 Appendix p 17
10	Provide a detailed description of all steps of the analysis, including mathematical formulae. This description should cover, as relevant, data cleaning, data pre-processing, data adjustments and weighting of data sources, and mathematical or statistical model(s).	Methods: Analysis pp 11-13<br>Appendix pp 6-7

11	Describe how candidate models were evaluated and how the final model(s) were selected.	Methods: Analysis pp 11-13<br>Appendix pp 6-7
12	Provide the results of an evaluation of model performance, if done, as well as the results of any relevant sensitivity analysis.	Appendix pp 6-7<br>Appendix p 11
13	Describe methods for calculating uncertainty of the estimates. State which sources of uncertainty were, and were not, accounted for in the uncertainty analysis.	Methods: Analysis pp 11-13<br>Appendix pp 6-7
14	State how analytic or statistical source code used to generate estimates can be accessed.	*R scripts available at https://github.com/ihmeuw/mental_disorders/tree/COVID-19_depressive_anxiety_disorders_lancet*
**Results and Discussion**		
15	Provide published estimates in a file format from which data can be efficiently extracted.	*Estimates by country available in Tables S6-S9 pp 32-63*
16	Report a quantitative measure of the uncertainty of the estimates (e.g. uncertainty intervals).	*All estimates are reported with 95% uncertainty intervals.*
17	Interpret results in light of existing evidence. If updating a previous set of estimates, describe the reasons for changes in estimates.	Discussion pp 17-19
18	Discuss limitations of the estimates. Include a discussion of any modelling assumptions or data limitations that affect interpretation of the estimates.	Discussion pp 20-23

Table S2: PRISMA 2020 checklist

Section and Topic	Item #	Checklist item	Location where item is reported
**TITLE**			
Title	1	Identify the report as a systematic review.	*The title instead reflects the focus of this study, which was to estimate the prevalence and burden of depressive and anxiety disorders due to the COVID-19 pandemic*
**ABSTRACT**			
Abstract	2	See the PRISMA 2020 for Abstracts checklist.	Abstract: p 4 – 5
**INTRODUCTION**			
Rationale	3	Describe the rationale for the review in the context of existing knowledge.	Research in context: *Evidence before this study:* p 2
Objectives	4	Provide an explicit statement of the objective(s) or question(s) the review addresses.	Background: pp 6-7
**METHODS**			
Eligibility criteria	5	Specify the inclusion and exclusion criteria for the review and how studies were grouped for the syntheses.	Methods: *Case definitions* p 7<br>Methods: *Data sources* pp 8 – 9
Information sources	6	Specify all databases, registers, websites, organisations, reference lists and other sources searched or consulted to identify studies. Specify the date when each source was last searched or consulted.	Methods: *Data sources* p 8
Search strategy	7	Present the full search strategies for all databases, registers and websites, including any filters and limits used.	Appendix: p 5
Selection process	8	Specify the methods used to decide whether a study met the inclusion criteria of the review, including how many reviewers screened each record and each report retrieved, whether they worked independently, and if applicable, details of automation tools used in the process.	Methods: *Data sources* pp 8 – 9<br>Author contributions: pp 23 – 24
Data collection process	9	Specify the methods used to collect data from reports, including how many reviewers collected data from each report, whether they worked independently, any processes for obtaining or confirming data from study investigators, and if applicable, details of automation tools used in the process.	Methods: *Data sources* pp 8 – 9<br>Author contributions: p 23
Data items	10a	List and define all outcomes for which data were sought. Specify whether all results that were compatible with each outcome domain in each study were sought (e.g. for all measures, time points, analyses), and if not, the methods used to decide which results to collect.	Methods: *Data sources* pp 8 – 9
	10b	List and define all other variables for which data were sought (e.g. participant and intervention characteristics, funding sources). Describe any assumptions made about any missing or unclear information.	Methods: *Data sources* pp 8 – 9
Study risk of bias assessment	11	Specify the methods used to assess risk of bias in the included studies, including details of the tool(s) used, how many reviewers assessed each study and whether they worked independently, and if applicable, details of automation tools used in the process.	Methods: *Analysis* pp 11 – 12
Effect measures	12	Specify for each outcome the effect measure(s) (e.g. risk ratio, mean difference) used in the synthesis or presentation of results.	Methods: *Analysis* pp 11 – 13
Synthesis methods	13a	Describe the processes used to decide which studies were eligible for each synthesis (e.g. tabulating the study intervention characteristics and comparing against the planned groups for each synthesis (item #5)).	Methods: *Data sources* pp 8 – 9
	13b	Describe any methods required to prepare the data for presentation or synthesis, such as handling of missing	Methods: *Analysis* pp 11 – 13

Section and Topic	Item #	Checklist item	Location where item is reported
		summary statistics, or data conversions.	Appendix : pp 5 – 6
	13c	Describe any methods used to tabulate or visually display results of individual studies and syntheses.	*Individual studies included in the analyses are summarised in Table S5 pp 33 – 34*
	13d	Describe any methods used to synthesize results and provide a rationale for the choice(s). If meta-analysis was performed, describe the model(s), method(s) to identify the presence and extent of statistical heterogeneity, and software package(s) used.	Methods: *Analysis* pp 11 – 12<br>Appendix : pp 9 – 11
	13e	Describe any methods used to explore possible causes of heterogeneity among study results (e.g. subgroup analysis, meta-regression).	Methods: *Analysis* pp 11 – 12<br>Appendix : pp 6 – 7
	13f	Describe any sensitivity analyses conducted to assess robustness of the synthesized results.	Appendix : pp 6 – 7<br>Appendix : p 11
Reporting bias assessment	14	Describe any methods used to assess risk of bias due to missing results in a synthesis (arising from reporting biases).	*The impact of COVID-19 was estimated for locations with no epidemiological data available through the use of COVID-19 impact indicators. Additional uncertainty due to missing data was incorporated into the final estimates as described below. Trimming was used to robustify final estimates (Appendix pp 6 – 7)*
Certainty assessment	15	Describe any methods used to assess certainty (or confidence) in the body of evidence for an outcome.	*Our uncertainty intervals for all results incorporate uncertainty across all input data, model results, prevalence estimation, severity estimation, and disability weights. Trimming was used to robustify final estimates (appendix pp 6 – 7)*
**RESULTS**			
Study selection	16a	Describe the results of the search and selection process, from the number of records identified in the search to the number of studies included in the review, ideally using a flow diagram.	Results: *Study characteristics* p11 – 12<br>Figure S3 p 19
	16b	Cite studies that might appear to meet the inclusion criteria, but which were excluded, and explain why they were excluded.	The number of studies excluded and reasons for exclusion are provided in Figure S3 p 19
Study characteristics	17	Cite each included study and present its characteristics.	Table S5 pp 30 – 31
Risk of bias in studies	18	Present assessments of risk of bias for each included study.	*Bias was accounted for via bias covariates in meta-regression*
Results of individual studies	19	For all outcomes, present, for each study: (a) summary statistics for each group (where appropriate) and (b) an effect estimate and its precision (e.g. confidence/credible interval), ideally using structured tables or plots.	*Prevalence change estimates varied by age, sex, measure, time and could not be appropriately summarised. Data informing the meta-regression is available at*<br>*https://github.com/ihmeuw/mental_disorders/tree/COVID-19_depressive_anxiety_disorders_lancet*

Section and Topic	Item #	Checklist item	Location where item is reported
Results of syntheses	20a	For each synthesis, briefly summarise the characteristics and risk of bias among contributing studies.	Results: *Impact of COVID-19 pandemic indicators* p 14 – 15
	20b	Present results of all statistical syntheses conducted. If meta-analysis was done, present for each the summary estimate and its precision (e.g. confidence/credible interval) and measures of statistical heterogeneity. If comparing groups, describe the direction of the effect.	Results: pp 14 – 17<br>Appendix :pp 6 – 7
	20c	Present results of all investigations of possible causes of heterogeneity among study results.	Appendix: pp 6 – 7
	20d	Present results of all sensitivity analyses conducted to assess the robustness of the synthesized results.	Appendix : p 6
Reporting biases	21	Present assessments of risk of bias due to missing results (arising from reporting biases) for each synthesis assessed.	*The impact of COVID-19 was estimated for locations with no epidemiological data available through the use of COVID-19 impact indicators. Additional uncertainty due to missing data was incorporated into the final estimates as described below. A leave-one-country-out cross validation was conducted to test the generalisability of the models to locations with missing data (Appendix: p 11)*
Certainty of evidence	22	Present assessments of certainty (or confidence) in the body of evidence for each outcome assessed.	*Our uncertainty intervals for all results incorporate uncertainty across all input data, model results, prevalence estimation, severity estimation, and disability weights. A leave-one-country-out cross validation was conducted to test the generalisability of the models to locations with missing data (Appendix: p 11)*
**DISCUSSION**			
Discussion	23a	Provide a general interpretation of the results in the context of other evidence.	Discussion pp 17 – 19
	23b	Discuss any limitations of the evidence included in the review.	Discussion pp 20 – 23
	23c	Discuss any limitations of the review processes used.	Discussion pp 20 – 23
	23d	Discuss implications of the results for practice, policy, and future research.	Discussion pp 19 – 20
**OTHER INFORMATION**			
Registration and protocol	24a	Provide registration information for the review, including register name and registration number, or state that the review was not registered.	Methods: *Data sources* p 8
	24b	Indicate where the review protocol can be accessed, or state that a protocol was not prepared.	Methods: *Data sources* p 8<br>*Protocol can be obtained by contacting the corresponding author.*
	24c	Describe and explain any amendments to information provided at registration or in the protocol.	*The start and end dates for the literature search were updated at the end of the project accordingly.*

Section and Topic	Item #	Checklist item	Location where item is reported
Support	25	Describe sources of financial or non-financial support for the review, and the role of the funders or sponsors in the review.	Methods: *Role of the funding source* p 13<br>Acknowledgements: p 24
Competing interests	26	Declare any competing interests of review authors.	Declarations of interest: p 24
Availability of data, code and other materials	27	Report which of the following are publicly available and where they can be found: template data collection forms; data extracted from included studies; data used for all analyses; analytic code; any other materials used in the review.	Data and code used for analyses are available in the upcoming GBD input data tool and at https://github.com/ihmeuw/mental_disorders/tree/COVID-19_depressive_anxiety_disorders_lancet

From: Page MJ, McKenzie JE, Bossuyt PM, Boutron I, Hoffmann TC, Mulrow CD, et al. The PRISMA 2020 statement: an updated guideline for reporting systematic reviews. BMJ 2021;372:n71. doi: 10.1136/bmj.n71

For more information, visit: http://www.prisma-statement.org/

Table S3: Severity proportions and disability weights for major depressive disorder and anxiety disorders in GBD 2020

Sequela	Severity proportions (95% UI)	Disability weights (95% UI)
**Major depressive disorder**		
Asymptomatic	13·0% (9·7–16·6)	0·000
Mild	59·4% (49·1–68·8)	0·145 (0·099–0·209)
Moderate	17·3% (12·7–22·1)	0·396 (0·267–0·531)
Severe	10·3% (3·2–19·8)	0·658 (0·477–0·807)
**Anxiety disorders**		
Asymptomatic	19·5% (16·1–23·1)	0·000
Mild	37·6% (28·4–46·6)	0·030 (0·018–0·046)
Moderate	26·7% (20·2–32·8)	0·133 (0·091–0·186)
Severe	16·2% (9·5–24·4)	0·523 (0·362–0·677)

UI=uncertainty interval.

Table S4: Bias covariates considered in the meta-regression of change in prevalence

Bias covariate	Reason for potential bias
Combined depressive and anxiety disorder symptoms	Estimates reflecting change in combined depressive and anxiety disorder symptoms will likely over-estimate the change compared to estimates of one disorder as the former will include more new cases than the latter.
Cross-sectional random sample	Comparisons between cross-sectional random samples are likely to be more heterogeneous than comparisons within a longitudinal sample. However, there is no clear prior to the direction of any systematic bias.
Cross-sectional market research / quota sample	Comparisons between cross-sectional random samples are likely to be more heterogeneous than comparisons within a longitudinal sample. Samples derived from market research / quota sampling are typically not randomly sampled from the population and there may be systematic biases in the sample selected and / or biases participant retention in o. These samples may have lower rates of employment, be more likely to come from a lower socioeconomic status, and have higher rates of mental disorders. Estimates of change in prevalence derived from these samples vs a baseline random sample may look larger than the estimates derived from the general population because their baseline prevalence prior to the pandemic was already higher than the general population and any increase in prevalence will exacerbate this difference.
Longitudinal market research / quota sample	Samples derived from market research quota sampling are not randomly sampled from the population and there may be systematic biases in the sample selected. These samples may have lower rates of employment, be more likely to come from a lower socioeconomic status, and have higher rates of mental disorders. Estimates of change in prevalence derived from these samples may look smaller than the estimates derived from the general population because their baseline prevalence prior to the pandemic was already higher than the general population.

Table S5: Characteristics of studies and data included in the analysis

Study name	Case name	Location	Ages	Sample size	Case definition	Baseline
**Longitudinal studies (14) ***						
Ayuso-Mateos et al[7]	Major depressive episode	Spain	18 to 99	1103	CIDI	
Biddle et al [21]	Depression and anxiety symptoms	Australia	18 to 99	3155	K6 (19+ score)	
Bulbulia et al[22] (1)	Depression and anxiety symptoms	New Zealand	18 to 99	940	K6 (13+ score)	
Daly et al (a)[23] (2)	Depression and anxiety symptoms	United Kingdom	18 to 99	14 393	GHQ-12 (3+score)	
Katz et al [24]	Probable depression and probable anxiety	United States of America	19 to 72	102	DASS-A (8+ score), DASS-D (11+ score)	
Katz et al [24]	Probable depression and probable anxiety	United Kingdom	21 to 75	100	DASS-A (8+ score), DASS-D (11+ score)	
Kikuchi et al[25]	Depression and anxiety symptoms	Tokyo, Japan	20 to 79	2078	K6 (13+ score)	
Kwong et al[26] (3)	Probable depression and probable anxiety	England	27 to 29	2872	SMFQ (11+score); GAD-7 (10+ score)	
Marroquin et al[27]	Probable depression and probable anxiety	United States of America	20 to 73	118	CES-D (16+ score), GAD-7 (10+ score)	
Peters et al[4] (4)	Probable depression and probable anxiety	Germany	20 to 74	113 928	PHQ-9 (10+ score) GAD-9 (10+ score)	
Van der Velden et al [28] (5)	Depression and anxiety symptoms	Netherlands	18 to 99	3980	MHI-5 (60+ score)	
Vizard et al[29] (6)	Probable depression	United Kingdom	18 to 99	3527	PHQ-8 (10+ score)	
Wanberg et al[30] (7)	Probable depression	United States of America	30 to 81	1143	PHQ-8 (10+ score)	
Widnall et al[31]	Probable depression and probable anxiety	England	13 to 14	1047	HADS-depression (7+score) HADS-Anxiety (9+ score)	
Zhang et al[32]	Probable depression and probable anxiety	Anhui, China	9 to 15	1241	MFQ (27+ score), HBQ(4 + score)	
**Cross-sectional studies (34)**						
ABS (2020)[33]	Depression and anxiety symptoms	Australia	18 to 99	16 370	K10 (22+ score)	ABS (2017/2018)[34]
Bryan et al[35]	Probable depression	United States of America	18 to 99	10 625	PHQ-9 (10+ score)	NHANES[36]
Choi et al[37]	Probable depression and probable anxiety	Hong Kong	18 to 99	500	PHQ-9 (10+ score), GAD-7 (10+ score)	Ni et al[38], Lau et al[39]
Daly et al (b)[40]	Probable depression	Ireland	18 to 99	1038	PHQ-9 (10+ score)	Ireland Health survey[41]
Ettman et al[42] (8)	Probable depression	United States of America	18 to 99	1441	PHQ-9 (10+ score)	NHANES[36]
Every-Palmer et al[43]	Depression and anxiety symptoms	New Zealand	18 to 90	2416	K6 (13+ score)	New Zealand Health Survey[44]
Fukase et al[45]	Probable depression	Japan	20 to 69	2708	PHQ-9 (10+ score)	Reiko et al[46]
Groarke et al[47]	Probable depression	United Kingdom	18 to 87	1402	PHQ-9 (10+ score)	Vizard et al[29]
Wilson et al[48]	Probable depression	United States of America	18 to 85	848	PHQ-9 (10+ score)	NHANES[36]
Kantor et al[49]	Probable depression and probable anxiety	United States of America	18 to 99	1005	PHQ-9 (10+ score), GAD-7 (10+ score)	NHANES[36], NCHS[50]
Killgore et al[51]	Probable depression	United States of America	18 to 84	1070	PHQ-9 (10+ score)	NHANES[36]

Study name	Case name	Location	Ages	Sample size	Case definition	Baseline
Kiuchi et al[52]	Depression and anxiety symptoms	Japan	18 to 99	1500	K6 (13+ score)	MHLW[53]
Knudsen et al[9]	Major depressive episode and anxiety disorder	Trondheim, Norway	20 to 65	566	CIDI	Knudsen et al[9]
McGinty et al (a)[54] (8)	Depression and anxiety symptoms	United States of America	18 to 99	1468	K6 (13+ score)	McGinty et al (a)[54] (8)
McGinty et al (b)[55] (8)	Depression and anxiety symptoms	United States of America	18 to 99	1337	K6 (13+ score)	McGinty et al (b)[55] (8)
Maxfield et al[56]	Probable depression	United States of America	18 to 82	485	PHQ-9 (10+ score)	NHANES[36]
O'Connor et al[57]	Probable depression	United Kingdom	18 to 99	3077	PHQ-9 (10+ score)	Vizard et al[29]
Peretti-Watel et al[58]	Probable depression	France	18 to 99	2003	PHQ-9 (10+ score)	IRDES 2014[59]
Pieh et al (a)[60]	Probable depression	Austria	18 to 99	437	PHQ-9 (10+ score)	Austria Health Survey[61]
Pieh et al (b)[62]	Probable depression	Austria	18 to 99	1005	PHQ-8 (10+ score)	Austria Health Survey[61]
Pieh et al (c)[63]	Probable depression	United Kingdom	18 to 99	436	PHQ-9 (10+ score)	Vizard et al[29]
Sønderskov et al (a)[64] (9)	Depression and anxiety symptoms	Denmark	18 to 99	3508	WHO-5 (50+ score)	Sønderskov et al[65]
Sønderskov et al (b)[65] (9)	Depression and anxiety symptoms	Denmark	18 to 99	3508	WHO-5 (50+ score)	Sønderskov et al[65]
Sante Publique France[66]	probable anxiety	France	18 to 99	2000	HAD (10+ score)	Sante Publique France[66]
Shevlin et al[67]	Probable depression	United Kingdom	18 to 83	2025	PHQ-9 (10+ score)	Vizard et al[29]
Sibley et al[68] (1)	Depression and anxiety symptoms	New Zealand	18 to 99	991	K6 (13+ score)	Sibley et al[68]
Twenge et al[69]	Depression and anxiety symptoms	United States of America	18 to 99	2032	K6 (13+ score)	NCHS[50]
Ravens-Sieberer et al[70] (10)	probable anxiety	Germany	11 to 17	1040	German version of SCARED (9+ score)	Ravens-Sieberer et al[70]
Ueda et al[71]	Probable depression	Japan	18 to 99	2000	PHQ-9 (10+ score)	Reiko et al[46]
Valiente et al[72]	Probable depression	Spain	18 to 75	2070	PHQ-9 (10+ score)	European Health survey[73]
Vieira et al[74]	Probable depression and probable anxiety	United States of America	17 to 74	887	DASS-A (8+ score)<br>DASS-D (11+ score)	Scholten et al[75]
Winkler et al[8]	Major depressive disorder and anxiety disorder	Czech Republic	18 to 99	3021	MINI	Winkler et al[8]
Yamamoto et al[76]	Probable depression, depression symptoms	Japan	18 to 89	11 333	PHQ-9 (10+ score), K6 (13+ score)	Reiko et al[46]
Zhou et al[77]	Probable depression and probable anxiety	United States of America	18 to 90	1025	DASS-A (8+ score)<br>DASS-D (11+ score)	Scholten et al[75]

CIDI: The Composite International Diagnostic Interview; PHQ-9: The Patient Health Questionnaire (PHQ)-9; GAD-7: General Anxiety Disorder-7; DASS: Depression Anxiety Stress Scales, K6 OR 10: Anxiety and depression checklist; GHQ-12: The General Health Questionnaire-12, MHI-5: The Mental Health Inventory; SCARED: Screen for Child Anxiety Related Disorders; HAD: Hospital Anxiety and Depression Scale; N/A: not available; * longitudinal studies have their own baseline. Some studies used the same cohort of people, cohorts available in more than one study were: (1): New Zealand Attitudes and Values Study (NZAVS); (2): UK Household Longitudinal Study (UKHLS or Understanding Society); (3): Avon Longitudinal Study of Parents and Children (ALSPAC); (4): German National Cohort (NAKO); (5): Longitudinal Internet studies for the Social Sciences (LISS) panel; (6): Opinions and Lifestyle Survey (OPN); (7): RAND American Life Panel; (8): AmeriSpeak standing panel; (9): COVID-19 Consequences Denmark Panel Survey 2020 (CCDPS 2020); (10): COPSY study

Table S6: Prevalence and DALYs of MDD per 100 000 persons, with 95% uncertainty intervals, by location, for the year 2020

Location	Baseline prevalence	Additional prevalence	Final prevalence	% change	Baseline DALYs	Additional DALYs	Final DALYs
**Global**	**2470·5 (2143·5–2870·7)**	**682·4 (574·1–807·2)**	**3152·9 (2722·5–3654·5)**	**27·6 (25·1–30·3)**	**497·0 (338·3–691·1)**	**137·1 (92·5–190·6)**	**634·1 (431·3–881·0)**
**Central Europe, eastern Europe, and central Asia**	**2519·7 (2185·0–2911·5)**	**741·6 (579·1–941·3)**	**3261·3 (2798·6–3804·8)**	**29·4 (23·9–35·8)**	**500·8 (343·0–704·9)**	**147·2 (97·2–211·5)**	**647·9 (441·3–907·3)**
**Central Asia**	**2096·6 (1774·9–2515·0)**	**728·4 (425·3–1082·9)**	**2825·0 (2301·8–3477·0)**	**34·7 (21·8–49·2)**	**425·5 (286·7–600·8)**	**147·6 (77·6–239·4)**	**573·1 (373·9–812·1)**
Armenia	2421·3 (2040·1–2886·1)	879·7 (153·6–1720·9)	3301·0 (2435·8–4324·7)	36·4 (6·3–70·4)	484·6 (322·8–687·8)	175·8 (31·0–363·4)	660·4 (408·0–980·4)
Azerbaijan	1859·9 (1546·6–2228·8)	646·4 (133·1–1276·4)	2506·3 (1872·9–3328·6)	34·8 (7·7–67·5)	377·1 (252·2–536·1)	130·9 (27·7–272·7)	508·0 (320·6–768·6)
Georgia	3086·1 (2595·2–3656·8)	848·6 (27·5–1847·3)	3934·7 (2927·9–5175·0)	27·5 (0·9–59·1)	611·5 (408·2–863·5)	167·9 (4·4–380·9)	779·4 (479·9–1151·2)
Kazakhstan	2460·7 (2094·0–2907·7)	861·8 (154·7–1662·7)	3322·5 (2463·9–4356·8)	35·0 (6·4–67·9)	496·2 (337·3–701·8)	173·4 (31·3–350·0)	669·7 (425·8–973·8)
Kyrgyzstan	2210·4 (1871·3–2645·7)	1129·7 (349·5–2123·9)	3340·1 (2453·3–4495·7)	51·2 (16·2–91·9)	450·7 (302·1–642·7)	230·8 (70·1–464·4)	681·5 (413·7–1048·0)
Mongolia	2906·3 (2451·9–3485·3)	62·4 (–613·4–877·4)	2968·8 (2177·7–3966·2)	2·2 (–20·1–30·1)	590·8 (391·9–836·5)	12·2 (–129·4–181·3)	603·0 (375·9–911·2)
Tajikistan	1546·1 (1276·2–1884·7)	597·1 (152·6–1154·1)	2143·2 (1580·3–2835·6)	38·6 (9·9–74·0)	316·1 (210·9–448·0)	121·9 (30·8–244·2)	438·0 (268·3–661·8)
Turkmenistan	1955·6 (1639·5–2368·5)	732·2 (155·9–1434·1)	2687·8 (1973·3–3614·1)	37·5 (8·1–73·1)	398·2 (261·8–572·6)	149·1 (30·2–325·8)	547·3 (336·9–826·0)
Uzbekistan	1919·7 (1591·0–2333·1)	682·8 (131·7–1356·9)	2602·5 (1910·9–3447·5)	35·5 (6·9–68·4)	391·9 (262·5–554·0)	139·0 (25·2–296·2)	530·9 (324·5–790·5)
**Central Europe**	**2080·1 (1791·2–2408·6)**	**517·6 (351·3–728·0)**	**2597·8 (2212·2–3041·6)**	**24·9 (17·1–34·6)**	**410·5 (278·5–569·9)**	**102·0 (59·6–156·7)**	**512·5 (347·5–707·6)**
Albania	1704·8 (1439·1–2027·8)	660·0 (125·1–1360·6)	2364·7 (1730·4–3164·1)	38·7 (7·2–76·9)	341·6 (229·9–485·7)	132·8 (25·1–288·4)	474·5 (284·0–712·7)
Bosnia and Herzegovina	2346·3 (1960·5–2820·9)	644·5 (43·1–1332·7)	2990·8 (2264·6–3875·4)	27·6 (1·9–56·8)	461·5 (305·7–651·0)	126·5 (10·8–287·0)	588·0 (375·7–876·3)
Bulgaria	2400·1 (1974·8–2852·8)	561·7 (–49·0–1311·8)	2961·8 (2237·5–3886·2)	23·5 (–2·0–53·0)	470·2 (314·6–658·9)	110·1 (–9·1–261·8)	580·3 (369·8–860·3)
Croatia	2771·3 (2316·7–3280·0)	549·7 (–86·1–1352·4)	3321·1 (2464·7–4342·6)	19·8 (–3·4–47·9)	542·8 (368·9–760·2)	108·1 (–19·2–284·1)	650·9 (411·0–991·3)
Czechia	2490·1 (2106·8–2945·4)	582·7 (–65·9–1297·3)	3072·8 (2308·3–3966·8)	23·5 (–2·5–52·9)	488·1 (330·3–685·7)	114·1 (–11·4–266·3)	602·1 (384·3–859·1)
Hungary	2573·9 (2159·9–3074·0)	368·4 (–231·3–1016·8)	2942·2 (2223·4–3832·4)	14·3 (–8·4–39·4)	503·2 (338·5–708·8)	72·3 (–44·3–217·0)	575·5 (351·8–846·8)
Montenegro	2225·3 (1878·1–2680·2)	716·3 (57·4–1440·6)	2941·6 (2184·6–3834·6)	32·2 (2·5–63·4)	442·1 (295·6–614·7)	142·0 (9·3–296·3)	584·0 (356·2–842·8)
North Macedonia	1913·4 (1594·4–2286·7)	698·0 (173·8–1384·2)	2611·4 (1939·8–3432·3)	36·5 (9·3–70·1)	379·9 (253·5–532·1)	138·3 (31·7–282·0)	518·3 (323·4–770·3)
Poland	1553·1 (1324·3–1813·5)	377·5 (250·7–530·0)	1930·6 (1626·1–2279·2)	24·3 (16·6–32·8)	308·7 (212·7–435·2)	74·8 (44·7–115·0)	383·5 (264·9–537·1)
Romania	2138·7 (1797·1–2524·4)	692·0 (100·9–1433·4)	2830·7 (2122·4–3731·4)	32·4 (4·7–67·2)	423·3 (282·3–588·6)	136·8 (20·0–311·7)	560·1 (350·6–823·7)
Serbia	2379·9 (1989·7–2835·7)	591·8 (–1·0–1315·7)	2971·7 (2245·3–3884·9)	24·8 (–0·0–55·3)	468·7 (315·3–653·3)	116·6 (–0·2–275·1)	585·3 (367·5–869·5)
Slovakia	2221·3 (1840·9–2636·6)	527·1 (–47·2–1199·0)	2748·4 (2032·5–3639·4)	23·8 (–2·3–52·9)	439·7 (287·3–618·9)	103·7 (–8·9–250·7)	543·4 (348·9–800·3)
Slovenia	2967·4 (2504·9–3525·3)	677·5 (–44·5–1554·1)	3644·9 (2749·2–4776·0)	22·8 (–1·6–51·0)	581·1 (393·3–820·3)	131·9 (–7·9–313·9)	713·0 (444·3–1053·7)
**Eastern Europe**	**2954·9 (2542·3–3412·6)**	**869·6 (641·2–1179·5)**	**3824·5 (3258·2–4497·5)**	**29·4 (22·1–38·3)**	**584·5 (399·5–816·9)**	**171·8 (109·2–261·0)**	**756·3 (518·1–1072·3)**
Belarus	3685·0 (3122·7–4320·9)	909·2 (–16·1–2013·9)	4594·2 (3420·3–5927·6)	24·7 (–0·5–54·0)	730·7 (490·1–1019·9)	179·8 (–2·6–419·7)	910·5 (575·4–1335·6)
Estonia	3723·5 (3086·1–4403·5)	539·8 (–309·7–1534·6)	4263·3 (3162·6–5540·8)	14·5 (–8·3–40·6)	731·2 (486·1–1029·5)	105·9 (–57·6–322·9)	837·1 (519·1–1218·7)
Latvia	3811·9 (3187·6–4496·8)	694·9 (–204·0–1841·5)	4506·8 (3346·7–6011·9)	18·2 (–5·4–47·1)	748·7 (501·4–1037·4)	136·7 (–34·5–362·0)	885·4 (545·6–1325·2)

Lithuania	4135·3 (3501·1–4863·0)	1079·1 (–28·2–2331·6)	5214·4 (3913·7–6746·4)	26·1 (–0·7–54·7)	816·0 (540·4–1143·8)	213·2 (–7·0–500·7)	1029·3 (642·0–1558·8)
Republic of Moldova	2923·1 (2449·7–3476·3)	1031·6 (214·7–2049·6)	3954·8 (2921·1–5201·4)	35·3 (7·1–69·4)	581·2 (386·2–834·5)	204·7 (39·6–417·8)	785·9 (494·1–1185·5)
Russia	2578·6 (2200·2–2998·8)	824·2 (671·0–1014·5)	3402·8 (2916·9–3983·6)	32·0 (27·2–37·1)	511·0 (347·8–717·9)	163·1 (108·3–233·0)	674·1 (461·9–947·9)
Ukraine	3918·9 (3346·4–4541·7)	1003·0 (105·1–2115·2)	4921·9 (3813·0–6341·2)	25·6 (2·6–52·4)	772·2 (528·9–1078·2)	196·9 (20·1–443·2)	969·1 (644·6–1460·1)
**High–income**	**3103·3 (2735·6–3526·4)**	**840·1 (671·7–1030·4)**	**3943·3 (3466·9–4516·1)**	**27·1 (22·6–31·5)**	**620·5 (427·4–854·2)**	**167·8 (107·9–239·3)**	**788·3 (544·7–1094·1)**
**Australasia**	**3534·3 (3033·8–4146·3)**	**387·3 (–332·6–1233·2)**	**3921·6 (2968·5–4977·1)**	**10·9 (–9·5–35·2)**	**711·1 (487·8–1006·6)**	**78·2 (–66·6–263·5)**	**789·3 (501·2–1179·8)**
Australia	3700·1 (3155·6–4368·0)	422·0 (–424·4–1439·0)	4122·1 (3038·4–5361·3)	11·4 (–11·8–39·4)	744·2 (511·1–1055·2)	85·2 (–85·3–305·3)	829·4 (514·5–1266·1)
New Zealand	2697·2 (2336·4–3159·1)	228·7 (–300·1–827·7)	2925·9 (2248·6–3732·3)	8·5 (–11·7–30·7)	543·8 (379·1–762·4)	46·3 (–62·8–180·9)	590·1 (373·7–865·2)
**High income Asia Pacific**	**1807·6 (1590·8–2042·8)**	**198·3 (52·1–367·0)**	**2005·9 (1736·2–2320·1)**	**11·0 (2·9–20·0)**	**364·7 (253·5–506·2)**	**40·0 (10·3–79·6)**	**404·6 (281·5–566·7)**
Brunei	1067·3 (866·6–1303·0)	130·2 (–132·5–449·0)	1197·5 (869·1–1632·8)	12·2 (–12·4–40·7)	220·1 (143·8–320·1)	26·8 (–26·7–92·5)	246·9 (150·6–377·6)
Japan	1839·0 (1630·6–2065·8)	201·6 (96·1–313·9)	2040·6 (1804·6–2315·4)	11·0 (5·2–16·8)	370·0 (258·2–511·6)	40·5 (17·3–67·7)	410·5 (286·9–574·3)
Singapore	1622·2 (1391·2–1883·3)	278·9 (–110·4–758·4)	1901·1 (1460·7–2510·8)	17·2 (–6·1–45·8)	332·2 (226·3–465·9)	57·3 (–21·6–160·9)	389·6 (245·5–588·3)
South Korea	1758·7 (1508·4–2044·4)	182·7 (–249·4–658·0)	1941·4 (1470·0–2514·9)	10·4 (–13·8–37·7)	356·7 (241·0–503·0)	36·9 (–49·4–140·9)	393·6 (244·5–592·5)
**High income North America**	**3509·0 (3132·7–3979·9)**	**1159·1 (914·4–1433·1)**	**4668·1 (4115·3–5340·2)**	**33·0 (27·2–39·5)**	**701·0 (490·2–965·6)**	**231·3 (151·7–336·7)**	**932·4 (653·3–1302·8)**
Canada	2392·4 (2040·0–2823·0)	560·2 (–56·2–1292·5)	2952·6 (2247·9–3871·9)	23·4 (–2·5–53·6)	484·5 (328·8–691·8)	113·3 (–12·5–285·2)	597·8 (383·0–883·6)
Greenland	5236·4 (4422·8–6200·8)	924·9 (–295·6–2357·0)	6161·3 (4696·8–8127·4)	17·7 (–5·5–43·5)	1063·2 (719·1–1501·3)	188·1 (–62·8–501·0)	1251·3 (795·4–1892·2)
USA	3633·1 (3243·2–4113·6)	1225·8 (971·4–1518·7)	4858·8 (4261·9–5537·4)	33·7 (27·4–40·6)	725·1 (508·2–999·3)	244·4 (156·8–351·9)	969·6 (680·6–1352·3)
**Southern Latin America**	**2361·0 (2068·1–2723·4)**	**981·4 (466·3–1580·1)**	**3342·4 (2700·0–4110·9)**	**41·6 (20·2–66·1)**	**478·2 (328·2–666·7)**	**198·4 (89·6–355·9)**	**676·5 (445·3–983·5)**
Argentina	1992·3 (1767·8–2259·4)	888·6 (305·2–1610·1)	2880·9 (2220·1–3718·5)	44·6 (15·3–80·6)	404·6 (282·6–563·7)	180·4 (57·5–361·0)	585·0 (365·8–894·1)
Chile	3246·4 (2738·4–3890·1)	1316·8 (298·5–2444·1)	4563·2 (3357·8–5987·3)	40·6 (10·2–76·0)	655·1 (438·5–921·9)	264·4 (62·9–511·8)	919·5 (579·9–1381·8)
Uruguay	2521·4 (2132·7–2981·6)	425·6 (–166·2–1152·2)	2947·0 (2218·5–3840·1)	16·9 (–6·7–44·2)	507·4 (346·2–715·9)	85·4 (–31·7–236·5)	592·8 (370·8–871·8)
**Western Europe**	**3401·8 (2958·5–3902·2)**	**856·6 (585·4–1180·8)**	**4258·4 (3685·5–4950·4)**	**25·2 (17·5–33·4)**	**678·2 (463·8–941·5)**	**170·6 (98·3–258·7)**	**848·8 (581·3–1203·3)**
Andorra	3198·1 (2660·8–3865·6)	1154·1 (236·1–2317·4)	4352·2 (3216·3–5765·3)	36·0 (7·8–70·2)	642·4 (426·8–913·5)	232·0 (44·8–497·8)	874·4 (532·6–1309·6)
Austria	2280·8 (1950·8–2649·9)	539·1 (–34·2–1245·6)	2820·0 (2124·2–3690·2)	23·6 (–1·5–52·2)	456·2 (312·4–638·9)	107·0 (–6·1–255·0)	563·2 (363·9–823·7)
Belgium	3028·8 (2566·1–3549·1)	885·4 (111·8–1873·3)	3914·2 (2904·7–5094·2)	29·2 (3·4–60·9)	605·3 (404·9–843·4)	177·5 (18·6–399·3)	782·7 (482·4–1157·3)
Cyprus	2421·4 (2007·0–2913·2)	500·1 (–124·7–1184·0)	2921·5 (2157·3–3813·6)	20·7 (–5·5–49·3)	488·3 (326·4–692·6)	100·7 (–23·1–263·2)	589·0 (359·0–879·7)
Denmark	2901·3 (2441·1–3408·9)	490·7 (–221·0–1306·3)	3392·0 (2547·6–4417·2)	17·0 (–7·4–44·4)	579·8 (391·3–816·3)	98·1 (–42·2–271·8)	677·9 (419·2–998·2)
Finland	3671·5 (3149·9–4254·8)	391·3 (–422·9–1412·0)	4062·8 (3045·7–5275·4)	10·6 (–12·0–36·5)	734·5 (499·6–1043·3)	79·1 (–80·4–291·4)	813·7 (497·0–1231·7)
France	3349·9 (2848·9–3908·3)	1117·3 (155·2–2194·0)	4467·2 (3357·0–5866·7)	33·3 (4·7–64·9)	667·9 (457·5–938·8)	223·1 (27·2–486·5)	890·9 (553·7–1347·5)
Germany	2907·1 (2476·8–3402·7)	485·3 (–199·1–1280·7)	3392·3 (2561·7–4442·0)	16·7 (–7·0–44·3)	576·0 (389·9–808·1)	95·1 (–42·5–263·4)	671·1 (438·1–978·4)
Greece	4917·1 (4108·9–5845·3)	851·0 (–341·6–2186·4)	5768·1 (4248·5–7440·6)	17·3 (–7·8–43·6)	978·4 (662·8–1386·1)	169·9 (–66·0–453·5)	1148·2 (724·6–1705·6)
Iceland	2196·0 (1855·1–2604·0)	410·7 (–127·7–1041·4)	2606·7 (1971·9–3383·3)	18·7 (–5·8–47·7)	442·7 (296·2–619·9)	82·3 (–25·8–225·7)	525·0 (324·2–787·9)

Ireland	3687·3 (3227·7–4256·9)	977·0 (39·0–2049·4)	4664·3 (3578·2–6019·7)	26·5 (1·0–55·0)	741·0 (514·3–1015·2)	197·2 (7·7–426·3)	938·2 (574·1–1379·7)
Israel	3319·5 (2782·9–3951·5)	925·9 (63·0–1910·3)	4245·4 (3179·0–5508·5)	27·9 (1·9–55·8)	671·7 (450·7–949·1)	186·6 (12·0–418·7)	858·2 (542·9–1290·7)
Italy	3358·2 (2892·3–3863·9)	769·3 (494·8–1056·7)	4127·4 (3535·1–4826·5)	22·9 (14·8–30·6)	666·1 (457·3–928·6)	152·3 (85·5–227·7)	818·4 (554·6–1141·1)
Luxembourg	2597·5 (2275·6–3002·6)	739·9 (51·6–1568·5)	3337·4 (2577·9–4244·1)	28·5 (1·8–59·5)	521·0 (358·3–719·4)	147·7 (9·7–322·0)	668·7 (429·8–991·6)
Malta	2478·6 (2063·2–2966·0)	480·7 (–131·2–1230·3)	2959·3 (2203·6–3889·8)	19·4 (–5·2–49·9)	496·0 (333·2–710·5)	96·0 (–26·6–263·0)	592·0 (372·4–889·9)
Monaco	3718·7 (2981·3–4578·9)	750·1 (–159·5–1831·5)	4468·8 (3290·8–5910·3)	20·2 (–4·3–49·9)	741·5 (487·7–1076·6)	149·0 (–30·4–365·2)	890·5 (548·5–1320·4)
Netherlands	2997·4 (2515·6–3530·9)	821·1 (17·5–1764·3)	3818·6 (2890·9–5008·1)	27·4 (0·5–56·5)	601·7 (403·6–841·4)	164·6 (3·7–372·6)	766·3 (478·6–1139·3)
Norway	3135·4 (2695·0–3660·6)	530·2 (221·7–904·6)	3665·5 (3079·1–4346·1)	16·9 (7·2–27·8)	631·8 (429·7–882·6)	106·6 (37·6–196·9)	738·4 (493·9–1043·6)
Portugal	4469·6 (3767·5–5246·8)	1329·0 (131·7–2652·5)	5798·6 (4449·6–7423·9)	29·8 (3·0–58·1)	884·3 (597·8–1239·3)	263·0 (27·7–544·4)	1147·4 (721·2–1700·4)
San Marino	3740·8 (2993·6–4646·4)	1227·5 (171·6–2452·6)	4968·2 (3475·8–6610·3)	32·8 (4·6–63·8)	747·3 (495·6–1083·8)	245·2 (32·9–515·4)	992·5 (610·5–1520·4)
Spain	4071·6 (3457·4–4667·6)	1199·6 (124·2–2394·6)	5271·2 (3984·7–6604·2)	29·5 (3·1–60·1)	814·0 (553·7–1121·1)	240·2 (21·5–508·2)	1054·2 (660·5–1544·8)
Sweden	3683·5 (3218·3–4213·2)	904·2 (149·7–1735·1)	4587·7 (3648·0–5680·2)	24·5 (4·0–47·0)	738·4 (507·0–1026·9)	180·7 (28·8–377·3)	919·1 (611·0–1315·7)
Switzerland	3160·8 (2780·5–3590·0)	549·1 (–192·3–1414·7)	3709·9 (2841·9–4688·2)	17·4 (–6·1–44·2)	630·2 (430·8–876·1)	109·5 (–33·8–299·0)	739·7 (469·7–1080·8)
UK	3641·1 (3157·3–4217·9)	1011·8 (827·3–1227·8)	4652·9 (4031·5–5393·2)	27·8 (24·0–31·7)	728·1 (495·7–1017·3)	202·1 (136·0–289·8)	930·2 (637·7–1290·8)
**Latin America and Caribbean**	**2626·8 (2291·4–3034·4)**	**914·2 (737·4–1127·5)**	**3541·0 (3063·3–4097·7)**	**34·8 (29·5–40·7)**	**527·5 (361·9–730·0)**	**183·5 (120·5–264·5)**	**711·0 (484·7–980·5)**
**Andean Latin America**	**1776·3 (1488·1–2126·8)**	**1010·8 (581·4–1542·6)**	**2787·1 (2205·5–3476·2)**	**56·9 (33·4–82·1)**	**360·3 (242·1–515·6)**	**204·5 (106·5–343·8)**	**564·8 (368·4–825·2)**
Bolivia	2360·1 (1951·9–2848·0)	1337·4 (429·7–2374·4)	3697·6 (2648·1–4979·0)	56·7 (19·4–99·0)	477·3 (316·6–679·8)	269·2 (88·6–515·4)	746·5 (465·5–1131·4)
Ecuador	2319·6 (1914·4–2778·8)	1240·5 (448·3–2194·9)	3560·0 (2604·9–4735·8)	53·5 (20·2–96·1)	471·7 (314·2–672·0)	251·4 (88·4–479·0)	723·1 (443·9–1074·2)
Peru	1292·8 (1080·3–1566·2)	780·3 (249·2–1517·5)	2073·1 (1459·7–2909·5)	60·3 (19·2–111·2)	262·0 (175·0–372·7)	158·1 (47·7–315·8)	420·2 (243·3–652·6)
**Caribbean**	**2856·7 (2419·5–3403·3)**	**623·1 (295·0–1000·8)**	**3479·8 (2874·5–4201·7)**	**21·8 (10·3–34·7)**	**573·4 (383·7–804·2)**	**125·0 (50·0–223·4)**	**698·5 (464·2–993·8)**
Antigua and Barbuda	2320·5 (1904·0–2823·0)	458·7 (–109·9–1162·8)	2779·2 (2071·7–3656·2)	19·8 (–4·4–48·8)	466·6 (308·3–667·7)	92·4 (–19·4–247·1)	559·0 (350·8–850·3)
Bahamas	2270·0 (1856·5–2767·4)	885·7 (230·7–1718·8)	3155·7 (2314·3–4251·4)	39·0 (10·5–72·2)	458·3 (303·3–657·8)	178·6 (43·1–360·1)	636·8 (398·5–967·0)
Barbados	2674·1 (2252·6–3177·9)	438·1 (–172·2–1239·0)	3112·3 (2302·7–4066·3)	16·4 (–6·8–45·6)	534·2 (353·7–763·2)	87·6 (–35·3–258·3)	621·8 (382·6–938·4)
Belize	2196·1 (1798·0–2643·6)	913·2 (233·4–1749·5)	3109·3 (2271·3–4206·7)	41·5 (10·7–78·2)	447·0 (300·4–647·0)	185·5 (43·9–375·9)	632·5 (390·9–980·5)
Bermuda	2875·0 (2417·8–3425·3)	533·5 (–167·6–1267·4)	3408·5 (2568·4–4365·4)	18·6 (–5·4–43·5)	573·5 (381·8–811·2)	105·9 (–32·7–272·0)	679·5 (439·7–1001·2)
Cuba	3663·2 (3095·8–4323·7)	530·8 (–294·8–1521·6)	4194·1 (3243·6–5457·0)	14·5 (–7·9–41·1)	728·2 (482·5–1015·2)	105·4 (–55·4–307·1)	833·6 (526·8–1210·7)
Dominica	2376·0 (1979·1–2858·9)	433·7 (–172·3–1127·0)	2809·7 (2107·8–3752·6)	18·3 (–7·0–48·1)	476·0 (320·2–667·7)	86·9 (–32·9–239·4)	562·9 (348·3–855·3)
Dominican Republic	2849·2 (2366·9–3435·0)	954·8 (146·8–1966·8)	3804·0 (2817·6–5057·2)	33·5 (5·1–67·1)	576·8 (385·7–819·8)	193·1 (30·1–438·5)	770·0 (482·7–1172·1)
Grenada	2490·6 (2087·3–3011·9)	456·5 (–117·1–1144·5)	2947·1 (2210·8–3914·1)	18·3 (–4·7–45·7)	502·0 (330·5–717·4)	92·2 (–25·1–239·8)	594·3 (366·7–873·8)
Guyana	3931·7 (3289·8–4679·7)	1215·1 (194·2–2485·5)	5146·8 (3855·5–6745·8)	30·9 (5·2–62·1)	791·5 (537·9–1123·9)	244·3 (36·6–531·3)	1035·7 (662·9–1537·6)
Haiti	2307·0 (1882·9–2848·9)	397·8 (–129·0–1012·1)	2704·8 (1950·0–3577·1)	17·3 (–6·1–42·9)	464·9 (306·6–671·3)	80·2 (–29·7–218·8)	545·1 (331·4–825·1)
Jamaica	2263·2 (1876·1–2730·4)	474·6 (–102·0–1165·6)	2737·8 (1991·9–3526·4)	21·1 (–4·6–51·0)	457·7 (307·1–645·7)	96·2 (–20·3–245·3)	553·9 (340·7–814·0)

Puerto Rico	2370·0 (2000·6–2820·7)	595·8 (–52·5–1313·6)	2965·8 (2195·2–3899·6)	25·2 (–2·5–54·0)	471·2 (315·1–669·6)	118·0 (–9·4–277·5)	589·1 (365·6–861·3)
Saint Kitts and Nevis	3487·2 (2757·9–4346·9)	614·1 (–219·0–1607·9)	4101·3 (2955·6–5466·8)	17·7 (–6·0–44·9)	701·0 (464·6–1035·9)	123·4 (–39·8–336·4)	824·4 (518·5–1258·3)
Saint Lucia	2702·9 (2273·1–3246·1)	508·0 (–117·9–1321·4)	3210·9 (2458·7–4241·2)	18·8 (–4·4–47·6)	541·6 (358·2–773·1)	102·0 (–24·1–271·1)	643·6 (404·1–964·6)
Saint Vincent and the Grenadines	2616·4 (2196·2–3124·7)	477·8 (–180·2–1201·6)	3094·2 (2309·0–4027·4)	18·3 (–7·0–46·2)	525·5 (348·4–741·5)	96·5 (–39·3–261·8)	622·0 (383·4–941·8)
Suriname	3900·1 (3300·5–4638·6)	1020·6 (66·1–2158·7)	4920·7 (3703·9–6391·2)	26·2 (1·8–54·8)	783·8 (520·6–1125·0)	203·9 (13·0–445·1)	987·7 (618·1–1423·7)
Trinidad and Tobago	3207·1 (2691·4–3778·2)	651·4 (–138·9–1657·3)	3858·5 (2905·5–5110·7)	20·3 (–4·3–52·2)	642·5 (434·3–910·3)	131·3 (–27·1–349·5)	773·8 (477·7–1166·7)
Virgin Islands	2896·0 (2420·6–3446·3)	359·0 (–315·2–1138·2)	3255·0 (2424·8–4296·4)	12·4 (–11·0–39·1)	574·2 (381·7–818·6)	70·8 (–65·3–240·6)	645·0 (408·7–964·7)
**Central Latin America**	**2468·8 (2114·9–2881·0)**	**982·4 (768·8–1257·4)**	**3451·2 (2935·0–4083·2)**	**39·8 (32·8–47·4)**	**498·0 (336·2–693·2)**	**198·1 (129·9–288·6)**	**696·1 (465·6–983·3)**
Colombia	1505·0 (1257·1–1814·5)	538·9 (85·0–1064·0)	2043·9 (1504·4–2763·1)	35·8 (5·6–69·2)	304·5 (203·3–444·1)	109·2 (16·4–229·7)	413·7 (255·4–619·2)
Costa Rica	2691·5 (2255·0–3214·9)	946·9 (178·8–1843·8)	3638·3 (2721·0–4779·4)	35·2 (6·7–68·7)	542·7 (361·0–766·3)	191·5 (34·4–420·6)	734·2 (449·4–1121·7)
El Salvador	2656·7 (2207·3–3216·6)	705·9 (46·3–1603·6)	3362·6 (2481·2–4503·7)	26·5 (1·8–59·3)	536·6 (358·7–772·5)	142·1 (9·2–330·3)	678·7 (418·3–1017·5)
Guatemala	2547·3 (2107·4–3086·3)	1061·3 (318·9–2026·5)	3608·6 (2677·3–4815·2)	41·7 (11·9–77·0)	512·5 (344·6–720·9)	213·3 (59·3–433·5)	725·8 (454·5–1096·2)
Honduras	2047·1 (1678·6–2503·8)	1039·9 (310·6–1922·1)	3087·0 (2245·9–4229·0)	50·7 (16·0–89·3)	414·1 (273·1–586·1)	210·3 (60·2–405·7)	624·4 (382·6–941·6)
Mexico	2829·9 (2456·4–3269·6)	1194·4 (934·8–1495·2)	4024·3 (3459·4–4685·7)	42·2 (34·8–50·6)	570·3 (387·2–788·3)	240·5 (152·2–350·0)	810·9 (543·6–1129·4)
Nicaragua	2352·0 (1946·9–2818·4)	692·0 (97·5–1427·4)	3044·0 (2260·3–4023·3)	29·4 (4·1–58·0)	477·6 (319·2–695·9)	140·9 (18·3–311·7)	618·5 (379·8–915·2)
Panama	2196·3 (1822·7–2673·6)	1235·7 (445·7–2221·8)	3432·0 (2463·8–4585·8)	56·3 (20·7–99·7)	443·6 (298·5–629·5)	250·1 (84·7–483·2)	693·7 (418·9–1059·6)
Venezuela	2571·3 (2145·1–3072·7)	816·2 (93·3–1645·3)	3387·6 (2508·9–4481·3)	31·8 (3·7–64·9)	518·6 (344·9–735·1)	164·7 (17·8–369·3)	683·3 (420·7–1039·9)
**Tropical Latin America**	**2996·6 (2659·6–3384·4)**	**872·1 (590·2–1157·6)**	**3868·7 (3341·8–4426·3)**	**29·1 (20·5–38·2)**	**597·8 (414·2–823·2)**	**174·1 (103·2–262·2)**	**771·9 (531·0–1065·3)**
Brazil	3022·1 (2683·2–3406·8)	875·1 (584·7–1167·1)	3897·2 (3376·5–4453·5)	29·0 (20·3–38·2)	602·7 (417·8–829·5)	174·6 (102·3–264·9)	777·3 (536·5–1069·8)
Paraguay	2230·3 (1856·3–2684·2)	783·8 (158·0–1543·2)	3014·1 (2224·5–4007·8)	35·1 (7·2–68·1)	451·0 (302·5–638·0)	158·9 (30·0–342·4)	609·9 (377·3–926·7)
**North Africa and Middle East**	**3321·4 (2752·3–4013·2)**	**1235·2 (896·1–1642·5)**	**4556·6 (3729·1–5578·3)**	**37·2 (29·5–46·0)**	**674·1 (449·3–956·3)**	**250·5 (155·5–375·5)**	**924·6 (617·5–1311·6)**
Afghanistan	3081·2 (2466·8–3836·6)	1091·5 (227·7–2101·7)	4172·8 (2941·5–5579·0)	35·4 (7·3–68·5)	625·9 (412·7–908·5)	222·5 (41·8–473·0)	848·3 (504·5–1310·4)
Algeria	3110·2 (2534·1–3806·1)	793·3 (19·9–1722·7)	3903·5 (2858·7–5181·0)	25·5 (0·6–54·1)	631·5 (417·0–905·5)	161·5 (3·4–384·7)	793·1 (488·0–1203·0)
Bahrain	4097·9 (3352·5–4993·4)	1169·4 (149·7–2492·1)	5267·3 (3856·2–7001·8)	28·5 (3·7–57·9)	833·8 (556·8–1190·0)	236·8 (27·5–536·0)	1070·6 (662·7–1595·2)
Egypt	2720·8 (2207·9–3348·5)	1164·3 (385·0–2204·3)	3885·1 (2845·0–5314·8)	42·8 (15·2–75·4)	554·9 (368·9–792·9)	237·4 (71·1–473·4)	792·3 (484·1–1202·0)
Iran	4031·8 (3315·5–4885·0)	1895·1 (1445·3–2446·1)	5927·0 (4847·6–7213·0)	47·0 (38·4–56·0)	812·4 (548·2–1154·6)	381·2 (238·9–564·1)	1193·6 (791·6–1677·3)
Iraq	2739·7 (2236·0–3362·0)	1144·0 (306·5–2149·0)	3883·7 (2793·9–5198·2)	41·7 (10·9–76·4)	557·3 (369·9–800·8)	231·2 (59·6–460·5)	788·6 (482·6–1174·9)
Jordan	2888·5 (2320·0–3579·3)	744·4 (7·4–1627·8)	3632·9 (2661·3–4918·0)	25·8 (0·3–53·2)	590·6 (386·1–841·9)	152·1 (1·3–341·4)	742·7 (454·1–1111·3)
Kuwait	3530·4 (2812·5–4370·3)	1293·1 (293·4–2491·5)	4823·5 (3541·0–6359·9)	36·7 (8·1–67·9)	718·5 (472·6–1039·8)	263·2 (57·3–540·7)	981·7 (602·2–1498·5)
Lebanon	3893·4 (3215·1–4760·7)	1087·9 (84·6–2207·3)	4981·4 (3596·1–6650·8)	27·9 (2·1–57·5)	783·0 (517·3–1126·4)	219·7 (15·0–492·9)	1002·7 (609·5–1531·0)
Libya	3767·8 (3012·6–4617·4)	1245·3 (192·0–2526·4)	5013·1 (3659·7–6666·7)	33·1 (4·9–64·8)	764·3 (508·8–1090·4)	251·7 (36·7–559·6)	1015·9 (622·6–1541·7)
Morocco	4279·2 (3544·7–5176·8)	1628·3 (389·6–3185·2)	5907·5 (4358·4–7809·9)	38·1 (9·2–72·3)	865·5 (578·8–1219·4)	329·5 (78·0–688·8)	1195·0 (759·9–1808·1)

Oman	3196·1 (2580·6–3991·5)	720·0 (–116·9–1656·0)	3916·1 (2790·5–5326·7)	22·5 (–3·5–52·2)	657·4 (429·6–945·6)	147·5 (–24·1–356·6)	804·9 (484·7–1198·0)
Palestine	4674·5 (3769·3–5739·2)	1883·3 (543·0–3513·4)	6557·9 (4782·7–8698·0)	40·3 (11·6–73·0)	954·8 (627·3–1367·4)	384·9 (102·8–766·2)	1339·7 (809·2–1998·0)
Qatar	3642·0 (2888·2–4595·6)	1346·0 (310·2–2631·0)	4988·1 (3571·5–6806·8)	37·0 (8·2–72·4)	750·4 (483·1–1075·6)	276·3 (57·8–584·3)	1026·7 (632·5–1560·5)
Saudi Arabia	3583·0 (2899·8–4397·5)	1354·8 (296·0–2578·6)	4937·8 (3459·8–6712·1)	37·8 (8·4–71·9)	729·5 (484·2–1033·2)	275·6 (56·6–555·3)	1005·0 (591·9–1533·6)
Sudan	2964·9 (2389·8–3655·8)	996·1 (214·3–1996·2)	3960·9 (2904·4–5470·8)	33·6 (7·2–64·7)	607·0 (396·6–874·3)	203·6 (41·0–451·5)	810·6 (494·0–1232·6)
Syria	3263·7 (2621·8–3999·8)	750·7 (–82·5–1756·4)	4014·4 (2949·3–5260·1)	23·0 (–2·8–52·2)	658·8 (433·5–931·4)	152·1 (–14·9–377·5)	810·9 (488·6–1239·3)
Tunisia	4750·6 (3921·1–5712·9)	1515·9 (204·2–3144·7)	6266·5 (4686·9–8297·2)	31·9 (4·4–65·1)	956·8 (643·4–1356·1)	305·1 (35·8–658·3)	1261·9 (800·0–1921·1)
Turkey	3195·9 (2637·7–3852·9)	1270·9 (308·5–2456·2)	4466·9 (3225·1–5965·4)	39·8 (10·3–74·6)	648·2 (434·5–928·0)	257·4 (61·9–526·3)	905·6 (573·8–1340·7)
United Arab Emirates	3382·0 (2630·5–4315·3)	867·3 (–15·4–1976·7)	4249·3 (3080·7–5805·9)	25·7 (–0·5–54·3)	691·0 (449·4–1012·1)	177·2 (–3·1–412·6)	868·2 (521·2–1352·2)
Yemen	3334·1 (2683·7–4168·4)	645·8 (–126·4–1563·7)	3980·0 (2943·8–5262·9)	19·4 (–3·8–47·1)	675·0 (446·3–980·3)	131·2 (–23·9–331·4)	806·1 (489·9–1205·1)
**South Asia**	**2664·2 (2313·9–3099·5)**	**962·6 (761·6–1187·1)**	**3626·8 (3122·5–4232·7)**	**36·1 (29·7–42·8)**	**534·5 (364·5–745·9)**	**192·8 (127·1–278·6)**	**727·3 (493·3–1012·5)**
Bangladesh	3592·0 (3002·3–4307·7)	1390·5 (313·2–2757·5)	4982·4 (3673·3–6670·0)	38·7 (8·8–74·7)	724·6 (486·0–1015·8)	280·6 (60·8–593·4)	1005·2 (626·9–1551·2)
Bhutan	2945·5 (2438·9–3573·7)	423·3 (–314·0–1312·3)	3368·8 (2474·2–4489·3)	14·4 (–10·5–44·1)	594·5 (393·6–844·0)	85·4 (–60·3–273·1)	679·9 (425·9–1017·3)
India	2577·1 (2247·5–2983·1)	901·0 (712·0–1118·5)	3478·1 (3006·3–4065·6)	35·0 (28·5–41·9)	516·0 (353·7–713·7)	180·0 (116·9–261·1)	696·0 (475·4–971·8)
Nepal	3638·2 (3112·0–4239·7)	1305·8 (292·2–2502·2)	4944·0 (3691·4–6494·2)	35·8 (7·9–66·8)	730·1 (490·4–1027·3)	262·7 (48·2–532·5)	992·8 (601·8–1475·1)
Pakistan	2413·2 (2022·1–2874·2)	992·3 (554·5–1533·8)	3405·5 (2743·1–4262·3)	41·1 (23·1–62·8)	487·1 (329·9–688·7)	200·4 (97·3–343·7)	687·5 (449·8–1007·2)
**Southeast Asia, east Asia, and Oceania**	**1707·8 (1492·4–1958·7)**	**195·8 (121·8–281·4)**	**1903·6 (1656·1–2194·3)**	**11·5 (7·2–16·0)**	**344·5 (236·4–481·1)**	**39·5 (21·3–62·7)**	**383·9 (261·4–538·1)**
**East Asia**	**1876·3 (1642·8–2156·0)**	**164·0 (63·8–271·8)**	**2040·3 (1765·5–2352·9)**	**8·7 (3·5–14·1)**	**376·3 (256·5–529·7)**	**32·8 (12·5–59·7)**	**409·2 (280·8–577·2)**
China	1889·0 (1655·0–2169·1)	167·3 (62·9–274·4)	2056·3 (1780·0–2364·6)	8·9 (3·3–14·5)	378·8 (258·5–533·0)	33·5 (12·7–60·9)	412·3 (283·3–581·6)
North Korea	1527·2 (1292·8–1820·7)	78·0 (–264·9–523·9)	1605·3 (1175·1–2151·9)	5·1 (–17·5–34·0)	308·9 (204·4–437·2)	15·4 (–55·7–107·1)	324·3 (202·7–482·0)
Taiwan (province of China)	1503·4 (1271·1–1782·5)	67·7 (–291·3–483·4)	1571·1 (1143·0–2094·9)	4·5 (–19·9–33·5)	301·4 (199·0–430·5)	13·9 (–57·8–99·9)	315·4 (191·1–479·1)
**Oceania**	**1570·6 (1288·1–1922·3)**	**25·6 (–276·6–392·6)**	**1596·2 (1219·7–2109·9)**	**1·7 (–17·6–24·0)**	**322·3 (214·5–468·2)**	**5·4 (–57·2–85·0)**	**327·7 (207·4–488·7)**
American Samoa	1289·4 (1074·4–1568·8)	141·0 (–151·9–509·6)	1430·4 (1061·0–1900·3)	10·9 (–12·3–38·6)	261·6 (175·3–373·1)	28·6 (–31·9–106·4)	290·2 (179·2–434·7)
Cook Islands	2018·1 (1628·3–2509·6)	200·7 (–274·8–790·1)	2218·8 (1589·9–3049·3)	9·9 (–13·4–37·6)	405·5 (272·7–587·8)	40·7 (–51·3–165·4)	446·1 (272·0–690·3)
Federated States of Micronesia	1515·8 (1250·4–1843·1)	44·2 (–317·3–486·2)	1560·0 (1133·5–2090·4)	3·0 (–20·8–31·8)	310·1 (207·0–443·2)	9·3 (–68·7–104·1)	319·5 (191·3–500·3)
Fiji	1540·4 (1276·8–1841·3)	124·3 (–256·8–521·2)	1664·7 (1219·4–2204·1)	8·1 (–16·1–32·9)	313·4 (211·7–447·1)	25·3 (–55·0–109·7)	338·7 (209·4–513·7)
Guam	1862·0 (1568·6–2238·3)	471·3 (–1·8–1059·1)	2333·3 (1734·3–3112·3)	25·3 (–0·1–55·5)	381·4 (256·3–544·4)	97·0 (–0·4–229·9)	478·3 (291·0–721·1)
Kiribati	1464·5 (1200·3–1810·6)	74·3 (–274·8–463·0)	1538·8 (1141·4–2077·9)	5·1 (–18·0–32·1)	300·0 (202·1–429·9)	15·4 (–55·5–97·6)	315·4 (192·0–484·8)
Marshall Islands	1458·4 (1194·3–1772·7)	54·3 (–284·7–489·7)	1512·7 (1091·1–2060·5)	3·7 (–19·0–31·3)	298·0 (199·8–429·6)	11·2 (–55·5–102·8)	309·3 (189·2–482·1)
Nauru	1638·5 (1264·3–2119·7)	199·2 (–207·4–649·7)	1837·7 (1268·7–2579·9)	12·1 (–12·7–38·6)	336·0 (215·4–500·2)	40·5 (–45·6–139·1)	376·5 (220·7–586·4)
Niue	1952·3 (1568·2–2427·9)	184·5 (–286·9–774·2)	2136·8 (1542·5–2876·2)	9·5 (–14·5–38·0)	393·0 (262·9–578·7)	37·4 (–54·2–157·6)	430·4 (266·1–672·5)
Northern Mariana Islands	1679·3 (1392·7–1999·9)	332·0 (–72·6–841·7)	2011·3 (1505·6–2655·6)	19·8 (–4·1–48·6)	341·6 (228·6–485·4)	67·5 (–13·4–178·9)	409·1 (255·0–619·4)

Palau	2034·6 (1605·9–2561·2)	163·1 (–298·3–763·5)	2197·7 (1561·6–2971·1)	8·1 (–15·1–36·4)	410·3 (270·2–603·6)	32·7 (–60·4–153·4)	443·0 (269·7–684·2)
Papua New Guinea	1593·0 (1299·0–1956·8)	3·2 (–389·8–450·4)	1596·3 (1157·5–2188·9)	0·3 (–24·7–28·6)	327·3 (218·9–474·6)	0·8 (–80·4–98·8)	328·1 (200·8–501·2)
Samoa	1302·9 (1081·3–1597·5)	25·9 (–289·1–379·9)	1328·8 (971·4–1808·9)	2·0 (–21·2–29·8)	266·3 (174·3–381·7)	5·4 (–59·0–85·5)	271·7 (166·5–418·1)
Solomon Islands	1435·3 (1162·8–1762·7)	33·4 (–307·4–417·2)	1468·7 (1042·6–2060·2)	2·3 (–20·9–30·0)	294·9 (195·8–429·9)	6·9 (–64·1–98·7)	301·8 (185·4–469·4)
Tokelau	1784·5 (1428·7–2235·7)	187·1 (–239·7–684·6)	1971·6 (1396·4–2677·3)	10·4 (–13·3–37·7)	362·2 (239·2–530·6)	38·2 (–49·3–147·5)	400·4 (238·7–623·0)
Tonga	1218·7 (1014·2–1486·4)	136·8 (–164·5–487·0)	1355·5 (976·1–1814·3)	11·2 (–14·4–38·7)	249·3 (166·1–357·6)	27·9 (–34·0–102·8)	277·1 (168·1–421·6)
Tuvalu	1750·6 (1364·5–2217·7)	177·3 (–220·2–633·7)	1927·9 (1360·7–2647·1)	10·1 (–13·4–35·0)	357·0 (236·3–530·8)	36·1 (–43·6–132·6)	393·0 (238·8–601·0)
Vanuatu	1426·1 (1177·7–1751·1)	24·5 (–316·8–430·9)	1450·6 (1032·1–2044·3)	1·6 (–22·6–28·8)	292·9 (197·2–426·5)	4·8 (–65·5–89·6)	297·7 (179·9–464·7)
**Southeast Asia**	**1351·7 (1152·3–1598·1)**	**267·1 (178·5–375·9)**	**1618·8 (1354·0–1939·6)**	**19·7 (13·5–26·1)**	**276·6 (187·4–389·1)**	**54·7 (31·0–85·2)**	**331·3 (222·4–468·2)**
Cambodia	1545·1 (1291·2–1861·3)	175·8 (–198·8–638·9)	1720·9 (1267·7–2309·8)	11·4 (–11·5–41·6)	316·8 (209·7–454·2)	36·4 (–38·6–138·7)	353·1 (215·4–541·7)
Indonesia	1164·7 (976·1–1373·3)	258·5 (159·6–378·3)	1423·2 (1180·6–1711·4)	22·2 (14·0–31·0)	239·6 (163·4–339·0)	53·1 (28·5–84·3)	292·7 (198·9–414·3)
Laos	1380·7 (1140·7–1672·7)	212·1 (–127·5–628·8)	1592·8 (1161·6–2135·1)	15·4 (–9·6–44·3)	285·4 (191·2–410·2)	43·9 (–26·4–137·8)	329·2 (200·8–483·6)
Malaysia	2081·4 (1733·9–2516·3)	281·8 (–191·9–873·9)	2363·2 (1711·8–3103·5)	13·5 (–8·8–42·6)	424·9 (285·3–606·8)	57·7 (–37·4–188·8)	482·6 (302·5–726·1)
Maldives	1735·2 (1433·8–2102·6)	466·0 (44·5–981·3)	2201·2 (1636·7–2936·5)	26·8 (2·6–55·0)	357·7 (238·7–500·2)	96·2 (8·1–228·2)	453·9 (285·7–704·7)
Mauritius	2960·8 (2487·0–3537·7)	405·1 (–309·7–1221·4)	3365·8 (2503·7–4391·6)	13·7 (–10·6–41·6)	596·0 (394·6–851·2)	81·7 (–60·9–256·0)	677·7 (428·2–1023·7)
Myanmar	918·7 (745·7–1126·2)	256·5 (12·0–549·7)	1175·2 (857·3–1573·2)	27·9 (1·4–56·8)	188·1 (124·9–268·5)	52·4 (2·3–121·4)	240·4 (146·6–360·7)
Philippines	1538·0 (1294·8–1817·4)	540·4 (419·2–681·4)	2078·5 (1740·5–2474·4)	35·1 (29·5–41·0)	315·5 (215·6–445·3)	110·8 (72·9–159·6)	426·3 (290·5–601·3)
Seychelles	1460·1 (1220·6–1754·3)	258·3 (–99·2–714·4)	1718·4 (1265·1–2311·6)	17·6 (–7·0–47·2)	297·7 (198·4–423·9)	52·7 (–20·1–153·6)	350·4 (216·8–540·1)
Sri Lanka	1622·5 (1382·7–1908·7)	321·9 (–62·5–794·7)	1944·4 (1490·5–2503·7)	19·9 (–4·1–47·2)	330·7 (222·5–467·6)	66·2 (–10·2–174·0)	396·9 (249·8–595·9)
Thailand	1867·6 (1577·0–2247·1)	132·4 (–294·9–685·5)	2000·0 (1459·6–2699·7)	7·1 (–16·4–36·5)	376·2 (254·7–538·2)	26·7 (–63·9–143·9)	402·9 (247·8–622·8)
Timor–Leste	1293·6 (1063·0–1591·8)	197·8 (–108·6–585·7)	1491·3 (1082·8–2034·1)	15·3 (–8·9–45·2)	266·2 (178·0–381·9)	40·7 (–22·9–130·8)	306·9 (193·9–468·0)
Vietnam	1159·0 (963·4–1391·4)	80·1 (–202·3–420·0)	1239·1 (888·6–1663·5)	6·9 (–17·8–35·3)	237·2 (157·1–338·6)	16·4 (–43·7–92·5)	253·7 (151·7–384·0)
**Sub–Saharan Africa**	**2429·0 (2048·0–2910·2)**	**559·0 (423·3–722·8)**	**2988·0 (2513·5–3583·4)**	**23·0 (18·3–27·9)**	**492·7 (333·6–695·6)**	**113·3 (71·2–168·7)**	**606·0 (408·5–857·1)**
**Central sub–Saharan Africa**	**3506·1 (2867·9–4267·0)**	**770·9 (171·3–1499·7)**	**4277·1 (3326·6–5497·1)**	**22·0 (4·9–41·9)**	**711·8 (474·7–1025·4)**	**156·7 (31·8–327·6)**	**868·5 (551·2–1262·3)**
Angola	3504·5 (2843·4–4270·5)	757·0 (–91·6–1714·1)	4261·5 (3156·9–5540·9)	21·7 (–2·7–47·8)	713·8 (474·7–1037·9)	153·6 (–18·9–362·5)	867·4 (528·8–1287·7)
Central African Republic	3921·2 (3214·0–4815·5)	694·3 (–260·4–1856·5)	4615·6 (3320·3–6217·7)	17·7 (–6·1–46·0)	793·2 (529·0–1143·5)	140·5 (–49·7–394·3)	933·7 (562·3–1467·6)
Congo	3952·9 (3233·8–4761·7)	913·4 (–27·5–2151·2)	4866·3 (3606·6–6545·3)	23·1 (–0·6–54·3)	801·9 (530·1–1155·4)	185·3 (–4·9–478·6)	987·2 (604·4–1501·4)
Democratic Republic of the Congo	3436·4 (2799·6–4180·4)	771·2 (–65·1–1774·5)	4207·6 (3069·6–5639·4)	22·4 (–2·2–50·9)	697·0 (464·2–1005·9)	156·8 (–8·6–388·2)	853·9 (520·4–1283·7)
Equatorial Guinea	3921·3 (3163·1–4832·3)	866·7 (–36·5–2093·1)	4788·0 (3523·3–6367·6)	22·1 (–1·0–51·1)	799·9 (530·7–1148·6)	177·3 (–5·9–443·7)	977·2 (600·1–1488·9)
Gabon	4036·9 (3319·4–4869·0)	731·5 (–209·6–1889·7)	4768·3 (3590·8–6316·4)	18·1 (–5·5–45·7)	817·1 (542·9–1162·1)	148·3 (–43·2–404·2)	965·4 (599·2–1437·7)
**Eastern sub–Saharan Africa**	**2541·3 (2137·0–3041·2)**	**521·0 (338·3–734·1)**	**3062·4 (2542·1–3688·6)**	**20·5 (13·5–27·4)**	**517·0 (348·6–724·7)**	**105·9 (60·4–168·1)**	**622·8 (419·5–876·8)**
Burundi	2375·5 (1940·3–2893·1)	233·9 (–302·8–921·3)	2609·5 (1935·1–3564·9)	9·8 (–12·5–36·7)	482·2 (317·5–686·7)	47·3 (–62·1–198·9)	529·5 (325·7–807·9)

Comoros	2635·9 (2168·8–3176·8)	579·6 (–49·5–1356·6)	3215·5 (2438·4–4295·4)	22·0 (–1·8–49·4)	535·1 (353·6–769·4)	117·3 (–11·3–281·7)	652·4 (418·8–975·7)
Djibouti	2636·4 (2142·3–3223·6)	475·1 (–125·1–1239·1)	3111·5 (2267·9–4144·1)	18·0 (–4·5–45·6)	538·8 (351·3–782·6)	97·1 (–24·1–258·4)	635·9 (395·2–964·2)
Eritrea	2698·3 (2194·4–3302·1)	268·6 (–329·9–948·4)	2966·9 (2222·0–3926·8)	10·1 (–11·8–34·8)	548·1 (359·1–786·8)	54·7 (–66·8–204·3)	602·9 (374·1–901·0)
Ethiopia	2414·0 (2030·5–2866·5)	496·6 (186·0–835·6)	2910·6 (2386·3–3553·7)	20·6 (7·7–33·9)	492·5 (335·4–699·2)	101·4 (36·4–186·7)	593·9 (394·2–844·0)
Kenya	2618·2 (2270·3–3036·1)	890·6 (703·8–1109·1)	3508·8 (3006·7–4110·1)	34·0 (28·7–39·6)	532·7 (363·4–736·9)	180·9 (119·9–264·2)	713·6 (494·7–1001·2)
Madagascar	2540·9 (2082·2–3101·2)	488·0 (–171·8–1206·5)	3028·9 (2201·4–4110·0)	19·2 (–6·7–46·2)	519·6 (346·7–744·1)	99·6 (–34·0–267·3)	619·2 (367·1–935·9)
Malawi	1982·9 (1626·2–2416·1)	394·4 (–120·1–1007·5)	2377·3 (1740·5–3167·2)	19·9 (–6·0–49·9)	404·4 (267·5–575·2)	80·5 (–21·3–211·2)	484·9 (292·1–728·1)
Mozambique	2458·3 (2028·9–2974·8)	435·5 (–155·1–1231·4)	2893·8 (2137·0–3852·1)	17·7 (–6·3–48·2)	493·2 (327·0–703·4)	87·2 (–29·3–246·4)	580·4 (353·7–851·8)
Rwanda	2928·0 (2402·8–3574·0)	485·4 (–187·5–1315·4)	3413·3 (2483·6–4539·7)	16·6 (–5·9–44·4)	593·2 (393·1–849·0)	98·1 (–37·5–282·8)	691·2 (433·2–1039·2)
Somalia	2304·8 (1875·6–2872·7)	408·6 (–134·6–1076·2)	2713·4 (2024·7–3619·8)	17·8 (–6·0–44·9)	469·6 (309·5–671·9)	82·7 (–25·6–222·5)	552·3 (338·2–830·8)
South Sudan	2350·3 (1944·0–2880·2)	342·8 (–199·4–1016·7)	2693·1 (1968·1–3583·4)	14·6 (–8·5–41·2)	474·4 (316·5–669·6)	68·9 (–37·7–206·2)	543·2 (343·3–794·6)
Uganda	3578·5 (2889·6–4394·0)	759·9 (–70·8–1864·7)	4338·4 (3178·7–5781·0)	21·3 (–2·2–49·2)	729·7 (483·6–1036·4)	154·3 (–15·6–398·3)	884·0 (555·9–1354·2)
Tanzania	2403·3 (1976·1–2929·0)	432·7 (–116·8–1102·8)	2836·1 (2124·9–3744·8)	18·0 (–4·9–45·5)	488·4 (321·7–691·9)	87·8 (–23·1–230·2)	576·1 (355·7–861·4)
Zambia	2031·4 (1661·5–2472·2)	254·1 (–223·3–845·6)	2285·5 (1685·1–3035·9)	12·5 (–11·2–40·8)	412·5 (271·3–594·7)	51·7 (–44·7–181·0)	464·2 (285·8–703·2)
**Southern sub–Saharan Africa**	**2807·3 (2428·2–3292·9)**	**1066·6 (742·0–1437·2)**	**3874·0 (3255·2–4609·8)**	**38·0 (27·1–48·6)**	**560·7 (387·3–774·8)**	**212·9 (128·1–330·1)**	**773·6 (523·5–1094·1)**
Botswana	2914·5 (2400·3–3561·3)	371·9 (–277·5–1174·5)	3286·4 (2418·1–4397·9)	12·7 (–9·7–40·4)	584·9 (390·1–840·7)	75·1 (–54·4–245·1)	660·0 (410·6–1003·9)
Eswatini	2786·4 (2282·6–3383·1)	1010·4 (241·5–2029·4)	3796·8 (2784·0–5138·3)	36·2 (8·5–68·6)	558·6 (376·1–802·3)	202·7 (41·8–435·0)	761·3 (470·1–1169·5)
Lesotho	4005·4 (3301·3–4866·8)	984·6 (–61·5–2123·5)	4990·0 (3700·5–6573·2)	24·6 (–1·5–54·0)	798·0 (538·8–1133·4)	195·9 (–11·1–455·1)	994·0 (628·1–1487·5)
Namibia	2121·0 (1753·8–2547·0)	620·8 (35·9–1302·0)	2741·8 (2005·5–3655·3)	29·2 (1·7–59·9)	429·0 (290·0–613·8)	125·6 (8·2–271·5)	554·6 (343·7–830·3)
South Africa	3129·4 (2725·7–3629·0)	1335·6 (896·3–1816·5)	4465·0 (3741·7–5286·2)	42·7 (30·0–55·6)	624·1 (431·2–865·3)	266·2 (155·1–415·0)	890·2 (603·4–1259·3)
Zimbabwe	1564·0 (1276·3–1915·5)	306·3 (–67·3–793·7)	1870·3 (1365·9–2450·9)	19·6 (–4·9–47·4)	315·3 (211·3–458·5)	61·7 (–14·3–160·4)	377·0 (232·1–572·2)
**Western sub–Saharan Africa**	**1967·1 (1652·6–2360·3)**	**448·9 (335·7–591·8)**	**2416·0 (2030·7–2892·0)**	**22·8 (17·8–27·9)**	**399·3 (270·3–561·0)**	**90·9 (56·9–134·0)**	**490·2 (331·6–700·0)**
Benin	2161·7 (1775·8–2630·8)	221·9 (–286·7–821·7)	2383·6 (1740·9–3203·4)	10·3 (–13·2–36·8)	439·4 (287·8–622·4)	44·7 (–60·8–176·2)	484·1 (297·9–724·9)
Burkina Faso	2107·2 (1736·0–2560·2)	246·1 (–232·8–802·3)	2353·3 (1747·3–3172·5)	11·7 (–11·5–37·9)	429·5 (285·4–616·0)	50·2 (–47·6–171·6)	479·8 (302·2–729·0)
Cabo Verde	3556·9 (2961·3–4272·4)	1652·0 (448·7–2968·1)	5209·0 (3822·5–6790·3)	46·5 (12·1–82·5)	721·7 (484·8–1008·4)	334·0 (90·1–620·6)	1055·8 (650·9–1584·1)
Cameroon	2405·4 (1966·5–2944·5)	285·7 (–274·9–980·1)	2691·1 (1938·3–3611·5)	11·9 (–11·1–38·6)	488·8 (326·0–697·3)	58·2 (–56·2–191·1)	546·9 (330·2–807·5)
Chad	2471·5 (2002·4–3040·4)	356·8 (–200·9–1050·2)	2828·3 (2086·3–3803·6)	14·5 (–8·7–41·4)	502·1 (333·6–713·2)	72·7 (–45·2–223·3)	574·9 (354·2–862·0)
Côte d’Ivoire	1949·9 (1589·8–2370·8)	240·4 (–221·3–795·2)	2190·3 (1613·4–2997·4)	12·3 (–11·6–40·7)	396·4 (262·5–567·8)	49·1 (–46·4–171·4)	445·5 (275·0–683·7)
The Gambia	3168·1 (2579·5–3874·0)	613·8 (–152·9–1550·4)	3781·9 (2762·3–5115·4)	19·4 (–4·8–46·4)	639·5 (422·8–912·8)	124·2 (–28·6–321·0)	763·7 (475·4–1158·9)
Ghana	2542·4 (2091·1–3107·9)	438·4 (–149·8–1167·9)	2980·8 (2231·1–3958·2)	17·2 (–6·0–45·1)	516·9 (341·1–747·0)	88·6 (–28·6–249·1)	605·4 (373·0–909·1)
Guinea	2205·6 (1820·0–2693·8)	375·9 (–166·9–1025·8)	2581·5 (1919·9–3391·4)	17·1 (–7·3–47·3)	447·8 (300·9–637·3)	74·7 (–38·3–209·4)	522·5 (335·8–778·6)
Guinea–Bissau	2260·8 (1845·6–2786·7)	393·5 (–150·8–1085·1)	2654·3 (1966·6–3557·2)	17·4 (–7·1–47·3)	459·0 (304·0–658·0)	79·7 (–32·8–230·2)	538·8 (340·5–809·6)

Liberia	2508·1 (2040·2–3078·3)	427·4 (–157·6–1238·9)	2935·6 (2176·2–3967·2)	17·1 (–6·4–46·6)	498·8 (329·8–705·2)	85·5 (–30·5–252·4)	584·3 (357·4–890·4)
Mali	1519·1 (1237·9–1869·3)	310·0 (–68·3–747·1)	1829·1 (1319·2–2441·0)	20·3 (–4·8–48·9)	309·3 (205·0–441·6)	63·0 (–13·7–167·8)	372·3 (228·3–563·1)
Mauritania	1845·6 (1523·8–2252·5)	426·5 (–35·2–945·4)	2272·1 (1700·3–3012·8)	23·1 (–1·8–51·6)	376·6 (248·9–539·4)	87·3 (–6·3–205·5)	463·9 (283·4–701·7)
Niger	1792·8 (1469·3–2213·8)	204·3 (–227·2–714·0)	1997·1 (1465·9–2659·3)	11·4 (–13·0–37·9)	366·3 (242·4–528·2)	41·4 (–47·0–151·7)	407·7 (250·4–615·1)
Nigeria	1735·1 (1467·2–2043·5)	578·6 (454·7–731·2)	2313·7 (1952·3–2757·4)	33·3 (27·8–39·0)	351·6 (240·8–495·1)	117·0 (76·4–168·8)	468·6 (317·6–650·6)
São Tomé and Príncipe	2093·4 (1707·9–2582·5)	433·2 (–93·9–1071·3)	2526·5 (1827·6–3390·7)	20·7 (–4·5–48·8)	427·6 (281·4–618·7)	88·5 (–18·0–231·1)	516·1 (319·3–794·8)
Senegal	2115·0 (1757·4–2585·7)	615·5 (50·2–1324·4)	2730·6 (2033·6–3651·3)	29·1 (2·6–59·8)	428·9 (285·1–611·7)	124·3 (10·0–267·6)	553·2 (342·4–817·4)
Sierra Leone	2373·9 (1947·2–2921·7)	374·1 (–206·6–1020·0)	2748·0 (2025·2–3618·9)	15·8 (–9·3–43·4)	481·3 (319·3–692·6)	75·5 (–43·7–227·7)	556·8 (349·5–832·4)
Togo	2489·1 (2041·6–3015·4)	336·0 (–228·6–1029·0)	2825·1 (2104·5–3767·3)	13·6 (–9·0–41·7)	505·9 (333·8–720·7)	67·9 (–47·5–213·2)	573·8 (362·8–860·9)

Table S7: Prevalence and DALYs of MDD in 1000s, with 95% uncertainty intervals, by location, for the year 2020

Location	Baseline prevalence	Additional prevalence	Final prevalence	% change	Baseline DALYs	Additional DALYs	Final DALYs
**Global**	**193000 (167000–224000)**	**53200 (44800–62900)**	**246000 (212000–285000)**	**27·6 (25·1–30·3)**	**38700 (26400–53900)**	**10700 (7210–14900)**	**49400 (33600–68700)**
**Central Europe, eastern Europe, and central Asia**	**10600 (9200–12300)**	**3120 (2440–3960)**	**13700 (11800–16000)**	**29·4 (23·9–35·8)**	**2110 (1440–2970)**	**619 (409–890)**	**2730 (1860–3820)**
**Central Asia**	**2040 (1720–2440)**	**708 (413–1050)**	**2740 (2240–3380)**	**34·7 (21·8–49·2)**	**413 (279–584)**	**143 (75·4–233)**	**557 (363–789)**
Armenia	73·1 (61·6–87·1)	26·6 (4·64–52)	99·7 (73·5–131)	36·4 (6·3–70·4)	14·6 (9·74–20·8)	5·31 (0·937–11)	19·9 (12·3–29·6)
Azerbaijan	192 (160–230)	66·8 (13·8–132)	259 (194–344)	34·8 (7·7–67·5)	39 (26·1–55·4)	13·5 (2·87–28·2)	52·5 (33·1–79·4)
Georgia	112 (94·5–133)	30·9 (0·999–67·2)	143 (107–188)	27·5 (0·9–59·1)	22·3 (14·9–31·4)	6·11 (0·161–13·9)	28·4 (17·5–41·9)
Kazakhstan	459 (390–542)	161 (28·8–310)	619 (459–812)	35·0 (6·4–67·9)	92·5 (62·9–131)	32·3 (5·82–65·2)	125 (79·3–181)
Kyrgyzstan	148 (125–177)	75·6 (23·4–142)	223 (164–301)	51·2 (16·2–91·9)	30·2 (20·2–43)	15·4 (4·69–31·1)	45·6 (27·7–70·1)
Mongolia	101 (85·2–121)	2·17 (–21·3–30·5)	103 (75·7–138)	2·2 (–20·1–30·1)	20·5 (13·6–29·1)	0·425 (–4·5–6·3)	21 (13·1–31·7)
Tajikistan	148 (122–181)	57·3 (14·6–111)	206 (152–272)	38·6 (9·9–74·0)	30·3 (20·2–43)	11·7 (2·96–23·4)	42 (25·8–63·5)
Turkmenistan	100 (84·1–121)	37·5 (7·99–73·5)	138 (101–185)	37·5 (8·1–73·1)	20·4 (13·4–29·4)	7·65 (1·55–16·7)	28·1 (17·3–42·3)
Uzbekistan	704 (583–855)	250 (48·3–497)	954 (700–1260)	35·5 (6·9–68·4)	144 (96·2–203)	51 (9·24–109)	195 (119–290)
**Central Europe**	**2370 (2040–2750)**	**590 (401–830)**	**2960 (2520–3470)**	**24·9 (17·1–34·6)**	**468 (318–650)**	**116 (67·9–179)**	**585 (396–807)**
Albania	45·6 (38·5–54·2)	17·6 (3·35–36·4)	63·2 (46·3–84·6)	38·7 (7·2–76·9)	9·14 (6·15–13)	3·55 (0·671–7·71)	12·7 (7·6–19·1)
Bosnia and Herzegovina	77 (64·4–92·6)	21·2 (1·42–43·8)	98·2 (74·4–127)	27·6 (1·9–56·8)	15·2 (10–21·4)	4·15 (0·354–9·42)	19·3 (12·3–28·8)
Bulgaria	165 (136–196)	38·6 (–3·37–90·2)	204 (154–267)	23·5 (–2·0–53·0)	32·3 (21·6–45·3)	7·57 (–0·629–18)	39·9 (25·4–59·2)
Croatia	117 (98·1–139)	23·3 (–3·65–57·3)	141 (104–184)	19·8 (–3·4–47·9)	23 (15·6–32·2)	4·58 (–0·811–12)	27·6 (17·4–42)
Czechia	265 (224–313)	62 (–7·02–138)	327 (246–422)	23·5 (–2·5–52·9)	51·9 (35·2–73)	12·1 (–1·21–28·3)	64·1 (40·9–91·4)
Hungary	248 (209–297)	35·6 (–22·3–98·2)	284 (215–370)	14·3 (–8·4–39·4)	48·6 (32·7–68·4)	6·98 (–4·28–20·9)	55·6 (34–81·7)
Montenegro	13·8 (11·7–16·7)	4·46 (0·357–8·96)	18·3 (13·6–23·9)	32·2 (2·5–63·4)	2·75 (1·84–3·82)	0·883 (0·0577–1·84)	3·63 (2·22–5·24)
North Macedonia	40·8 (34–48·8)	14·9 (3·71–29·5)	55·7 (41·4–73·2)	36·5 (9·3–70·1)	8·1 (5·41–11·3)	2·95 (0·676–6·01)	11·1 (6·9–16·4)
Poland	597 (509–697)	145 (96·3–204)	742 (625–875)	24·3 (16·6–32·8)	119 (81·7–167)	28·7 (17·2–44·2)	147 (102–206)
Romania	410 (344–484)	133 (19·3–275)	542 (407–715)	32·4 (4·7–67·2)	81·1 (54·1–113)	26·2 (3·83–59·7)	107 (67·2–158)
Serbia	211 (176–251)	52·5 (–0·0897–117)	263 (199–344)	24·8 (–0·0–55·3)	41·5 (27·9–57·9)	10·3 (–0·0191–24·4)	51·9 (32·6–77·1)
Slovakia	121 (100–143)	28·6 (–2·57–65·1)	149 (110–198)	23·8 (–2·3–52·9)	23·9 (15·6–33·6)	5·64 (–0·483–13·6)	29·5 (19–43·5)
Slovenia	61·6 (52–73·1)	14·1 (–0·923–32·2)	75·6 (57–99·1)	22·8 (–1·6–51·0)	12·1 (8·16–17)	2·74 (–0·164–6·51)	14·8 (9·22–21·9)
**Eastern Europe**	**6190 (5330–7150)**	**1820 (1340–2470)**	**8020 (6830–9430)**	**29·4 (22·1–38·3)**	**1230 (838–1710)**	**360 (229–547)**	**1590 (1090–2250)**
Belarus	346 (294–406)	85·5 (–1·52–189)	432 (322–557)	24·7 (–0·5–54·0)	68·7 (46·1–95·9)	16·9 (–0·245–39·5)	85·6 (54·1–126)
Estonia	48·9 (40·5–57·8)	7·09 (–4·07–20·2)	56 (41·5–72·8)	14·5 (–8·3–40·6)	9·61 (6·38–13·5)	1·39 (–0·757–4·24)	11 (6·82–16)
Latvia	72·3 (60·5–85·3)	13·2 (–3·87–34·9)	85·5 (63·5–114)	18·2 (–5·4–47·1)	14·2 (9·51–19·7)	2·59 (–0·655–6·87)	16·8 (10·4–25·1)

Lithuania	114 (96·8–134)	29·8 (–0·781–64·5)	144 (108–187)	26·1 (–0·7–54·7)	22·6 (14·9–31·6)	5·9 (–0·192–13·8)	28·5 (17·7–43·1)
Republic of Moldova	106 (89·2–127)	37·6 (7·82–74·6)	144 (106–189)	35·3 (7·1–69·4)	21·2 (14·1–30·4)	7·45 (1·44–15·2)	28·6 (18–43·2)
Russia	3780 (3220–4390)	1210 (983–1490)	4990 (4270–5840)	32·0 (27·2–37·1)	749 (510–1050)	239 (159–341)	988 (677–1390)
Ukraine	1730 (1480–2000)	442 (46·3–932)	2170 (1680–2800)	25·6 (2·6–52·4)	340 (233–475)	86·8 (8·84–195)	427 (284–644)
**High–income**	**33700 (29700–38300)**	**9130 (7300–11200)**	**42900 (37700–49100)**	**27·1 (22·6–31·5)**	**6740 (4650–9280)**	**1820 (1170–2600)**	**8570 (5920–11900)**
**Australasia**	**1060 (906–1240)**	**116 (–99·3–368)**	**1170 (886–1490)**	**10·9 (–9·5–35·2)**	**212 (146–301)**	**23·4 (–19·9–78·7)**	**236 (150–352)**
Australia	920 (784–1090)	105 (–105–358)	1020 (755–1330)	11·4 (–11·8–39·4)	185 (127–262)	21·2 (–21·2–75·9)	206 (128–315)
New Zealand	135 (117–158)	11·4 (–15–41·4)	146 (113–187)	8·5 (–11·7–30·7)	27·2 (19–38·2)	2·32 (–3·14–9·05)	29·5 (18·7–43·3)
**High income Asia Pacific**	**3380 (2970–3820)**	**371 (97·4–686)**	**3750 (3240–4340)**	**11·0 (2·9–20·0)**	**682 (474–946)**	**74·7 (19·2–149)**	**756 (526–1060)**
Brunei	4·76 (3·87–5·81)	0·581 (–0·591–2)	5·34 (3·88–7·28)	12·2 (–12·4–40·7)	0·982 (0·642–1·43)	0·119 (–0·119–0·413)	1·1 (0·672–1·68)
Japan	2340 (2080–2630)	257 (122–400)	2600 (2300–2950)	11·0 (5·2–16·8)	471 (329–651)	51·6 (22–86·1)	523 (365–731)
Singapore	92·4 (79·2–107)	15·9 (–6·29–43·2)	108 (83·2–143)	17·2 (–6·1–45·8)	18·9 (12·9–26·5)	3·27 (–1·23–9·17)	22·2 (14–33·5)
South Korea	940 (806–1090)	97·6 (–133–352)	1040 (785–1340)	10·4 (–13·8–37·7)	191 (129–269)	19·7 (–26·4–75·3)	210 (131–317)
**High income North America**	**12900 (11500–14600)**	**4250 (3350–5250)**	**17100 (15100–19600)**	**33·0 (27·2–39·5)**	**2570 (1800–3540)**	**848 (556–1230)**	**3420 (2390–4770)**
Canada	878 (749–1040)	206 (–20·6–474)	1080 (825–1420)	23·4 (–2·5–53·6)	178 (121–254)	41·6 (–4·59–105)	219 (141–324)
Greenland	2·94 (2·48–3·48)	0·52 (–0·166–1·32)	3·46 (2·64–4·57)	17·7 (–5·5–43·5)	0·597 (0·404–0·843)	0·106 (–0·0353–0·281)	0·703 (0·447–1·06)
USA	12000 (10700–13600)	4040 (3200–5010)	16000 (14000–18300)	33·7 (27·4–40·6)	2390 (1680–3290)	806 (517–1160)	3200 (2240–4460)
**Southern Latin America**	**1590 (1390–1830)**	**659 (313–1060)**	**2250 (1810–2760)**	**41·6 (20·2–66·1)**	**321 (220–448)**	**133 (60·2–239)**	**454 (299–661)**
Argentina	905 (803–1030)	404 (139–732)	1310 (1010–1690)	44·6 (15·3–80·6)	184 (128–256)	82 (26·1–164)	266 (166–406)
Chile	594 (501–712)	241 (54·6–447)	835 (614–1100)	40·6 (10·2–76·0)	120 (80·2–169)	48·4 (11·5–93·6)	168 (106–253)
Uruguay	86·8 (73·4–103)	14·6 (–5·72–39·6)	101 (76·3–132)	16·9 (–6·7–44·2)	17·5 (11·9–24·6)	2·94 (–1·09–8·14)	20·4 (12·8–30)
**Western Europe**	**14900 (12900–17000)**	**3740 (2560–5150)**	**18600 (16100–21600)**	**25·2 (17·5–33·4)**	**2960 (2020–4110)**	**745 (429–1130)**	**3710 (2540–5250)**
Andorra	2·7 (2·25–3·26)	0·974 (0·199–1·96)	3·67 (2·72–4·87)	36·0 (7·8–70·2)	0·542 (0·36–0·771)	0·196 (0·0378–0·42)	0·738 (0·45–1·11)
Austria	204 (175–237)	48·3 (–3·07–112)	253 (190–331)	23·6 (–1·5–52·2)	40·9 (28–57·2)	9·59 (–0·55–22·8)	50·5 (32·6–73·8)
Belgium	347 (294–407)	101 (12·8–215)	448 (333–584)	29·2 (3·4–60·9)	69·3 (46·4–96·6)	20·3 (2·13–45·7)	89·7 (55·3–133)
Cyprus	32·2 (26·7–38·8)	6·66 (–1·66–15·8)	38·9 (28·7–50·8)	20·7 (–5·5–49·3)	6·5 (4·34–9·22)	1·34 (–0·308–3·5)	7·84 (4·78–11·7)
Denmark	169 (142–199)	28·6 (–12·9–76·2)	198 (149–258)	17·0 (–7·4–44·4)	33·8 (22·8–47·6)	5·72 (–2·46–15·9)	39·5 (24·5–58·2)
Finland	203 (174–236)	21·7 (–23·4–78·2)	225 (169–292)	10·6 (–12·0–36·5)	40·7 (27·7–57·8)	4·38 (–4·45–16·1)	45·1 (27·5–68·2)
France	2220 (1890–2590)	741 (103–1450)	2960 (2230–3890)	33·3 (4·7–64·9)	443 (303–622)	148 (18–323)	591 (367–893)
Germany	2480 (2110–2900)	414 (–170–1090)	2890 (2180–3790)	16·7 (–7·0–44·3)	491 (333–689)	81·1 (–36·2–225)	572 (374–834)
Greece	504 (421–599)	87·3 (–35–224)	591 (436–763)	17·3 (–7·8–43·6)	100 (68–142)	17·4 (–6·77–46·5)	118 (74·3–175)

Iceland	7·62 (6·44–9·04)	1·43 (–0·443–3·61)	9·05 (6·84–11·7)	18·7 (–5·8–47·7)	1·54 (1·03–2·15)	0·286 (–0·0896–0·783)	1·82 (1·12–2·73)
Ireland	182 (159–210)	48·1 (1·92–101)	230 (176–297)	26·5 (1·0–55·0)	36·5 (25·3–50)	9·71 (0·38–21)	46·2 (28·3–68)
Israel	314 (263–374)	87·6 (5·96–181)	402 (301–521)	27·9 (1·9–55·8)	63·6 (42·6–89·8)	17·7 (1·13–39·6)	81·2 (51·4–122)
Italy	2020 (1740–2320)	462 (297–635)	2480 (2120–2900)	22·9 (14·8–30·6)	400 (275–558)	91·5 (51·4–137)	492 (333–686)
Luxembourg	16·4 (14·4–19)	4·67 (0·326–9·91)	21·1 (16·3–26·8)	28·5 (1·8–59·5)	3·29 (2·26–4·54)	0·933 (0·0614–2·03)	4·22 (2·71–6·26)
Malta	10·9 (9·09–13·1)	2·12 (–0·578–5·42)	13 (9·71–17·1)	19·4 (–5·2–49·9)	2·18 (1·47–3·13)	0·423 (–0·117–1·16)	2·61 (1·64–3·92)
Monaco	1·4 (1·12–1·72)	0·283 (–0·0601–0·69)	1·68 (1·24–2·23)	20·2 (–4·3–49·9)	0·279 (0·184–0·406)	0·0561 (–0·0114–0·138)	0·335 (0·207–0·497)
Netherlands	515 (432–607)	141 (3–303)	656 (497–861)	27·4 (0·5–56·5)	103 (69·4–145)	28·3 (0·641–64)	132 (82·3–196)
Norway	169 (145–197)	28·6 (11·9–48·7)	197 (166–234)	16·9 (7·2–27·8)	34 (23·1–47·5)	5·74 (2·03–10·6)	39·8 (26·6–56·2)
Portugal	476 (401–559)	142 (14–282)	617 (474–791)	29·8 (3·0–58·1)	94·2 (63·7–132)	28 (2·95–58)	122 (76·8–181)
San Marino	1·24 (0·989–1·53)	0·405 (0·0567–0·81)	1·64 (1·15–2·18)	32·8 (4·6–63·8)	0·247 (0·164–0·358)	0·081 (0·0109–0·17)	0·328 (0·202–0·502)
Spain	1860 (1580–2140)	549 (56·9–1100)	2410 (1820–3020)	29·5 (3·1–60·1)	373 (254–513)	110 (9·84–233)	483 (302–707)
Sweden	380 (332–434)	93·2 (15·4–179)	473 (376–586)	24·5 (4·0–47·0)	76·1 (52·3–106)	18·6 (2·97–38·9)	94·8 (63–136)
Switzerland	280 (246–318)	48·6 (–17–125)	329 (252–415)	17·4 (–6·1–44·2)	55·8 (38·2–77·6)	9·7 (–3–26·5)	65·5 (41·6–95·7)
UK	2440 (2110–2820)	677 (554–822)	3120 (2700–3610)	27·8 (24·0–31·7)	487 (332–681)	135 (91·1–194)	623 (427–864)
**Latin America and Caribbean**	**15400 (13400–17800)**	**5360 (4320–6610)**	**20800 (18000–24000)**	**34·8 (29·5–40·7)**	**3090 (2120–4280)**	**1080 (706–1550)**	**4170 (2840–5750)**
**Andean Latin America**	**1130 (950–1360)**	**645 (371–985)**	**1780 (1410–2220)**	**56·9 (33·4–82·1)**	**230 (155–329)**	**131 (68–219)**	**361 (235–527)**
Bolivia	274 (227–331)	155 (49·9–276)	429 (307–578)	56·7 (19·4–99·0)	55·4 (36·7–78·9)	31·2 (10·3–59·8)	86·6 (54–131)
Ecuador	417 (344–500)	223 (80·6–395)	640 (468–851)	53·5 (20·2–96·1)	84·8 (56·5–121)	45·2 (15·9–86·1)	130 (79·8–193)
Peru	443 (370–537)	267 (85·4–520)	710 (500–997)	60·3 (19·2–111·2)	89·8 (60–128)	54·2 (16·3–108)	144 (83·4–224)
**Caribbean**	**1350 (1150–1610)**	**295 (140–474)**	**1650 (1360–1990)**	**21·8 (10·3–34·7)**	**272 (182–381)**	**59·2 (23·7–106)**	**331 (220–471)**
Antigua and Barbuda	2·06 (1·69–2·5)	0·407 (–0·0974–1·03)	2·46 (1·84–3·24)	19·8 (–4·4–48·8)	0·414 (0·273–0·592)	0·0819 (–0·0172–0·219)	0·496 (0·311–0·754)
Bahamas	8·75 (7·15–10·7)	3·41 (0·889–6·62)	12·2 (8·92–16·4)	39·0 (10·5–72·2)	1·77 (1·17–2·53)	0·688 (0·166–1·39)	2·45 (1·54–3·73)
Barbados	7·94 (6·69–9·44)	1·3 (–0·511–3·68)	9·24 (6·84–12·1)	16·4 (–6·8–45·6)	1·59 (1·05–2·27)	0·26 (–0·105–0·767)	1·85 (1·14–2·79)
Belize	9·22 (7·55–11·1)	3·84 (0·98–7·35)	13·1 (9·54–17·7)	41·5 (10·7–78·2)	1·88 (1·26–2·72)	0·779 (0·185–1·58)	2·66 (1·64–4·12)
Bermuda	1·84 (1·54–2·19)	0·341 (–0·107–0·81)	2·18 (1·64–2·79)	18·6 (–5·4–43·5)	0·366 (0·244–0·518)	0·0677 (–0·0209–0·174)	0·434 (0·281–0·64)
Cuba	415 (351–490)	60·2 (–33·4–172)	475 (368–618)	14·5 (–7·9–41·1)	82·5 (54·7–115)	11·9 (–6·28–34·8)	94·5 (59·7–137)
Dominica	1·62 (1·35–1·95)	0·296 (–0·118–0·769)	1·92 (1·44–2·56)	18·3 (–7·0–48·1)	0·325 (0·218–0·456)	0·0593 (–0·0224–0·163)	0·384 (0·238–0·584)
Dominican Republic	311 (258–375)	104 (16–215)	415 (307–552)	33·5 (5·1–67·1)	62·9 (42·1–89·4)	21·1 (3·28–47·8)	84 (52·7–128)
Grenada	2·53 (2·12–3·06)	0·464 (–0·119–1·16)	3 (2·25–3·98)	18·3 (–4·7–45·7)	0·511 (0·336–0·73)	0·0938 (–0·0255–0·244)	0·605 (0·373–0·889)

Guyana	30·2 (25·3–36)	9·34 (1·49–19·1)	39·6 (29·6–51·9)	30·9 (5·2–62·1)	6·08 (4·13–8·64)	1·88 (0·281–4·08)	7·96 (5·1–11·8)
Haiti	291 (238–360)	50·2 (–16·3–128)	342 (246–452)	17·3 (–6·1–42·9)	58·7 (38·7–84·8)	10·1 (–3·75–27·6)	68·9 (41·9–104)
Jamaica	62·9 (52·2–75·9)	13·2 (–2·84–32·4)	76·1 (55·4–98·1)	21·1 (–4·6–51·0)	12·7 (8·54–18)	2·68 (–0·563–6·82)	15·4 (9·48–22·6)
Puerto Rico	82·3 (69·5–97·9)	20·7 (–1·82–45·6)	103 (76·2–135)	25·2 (–2·5–54·0)	16·4 (10·9–23·2)	4·1 (–0·326–9·64)	20·5 (12·7–29·9)
Saint Kitts and Nevis	2·04 (1·61–2·54)	0·359 (–0·128–0·94)	2·4 (1·73–3·2)	17·7 (–6·0–44·9)	0·41 (0·272–0·606)	0·0722 (–0·0233–0·197)	0·482 (0·303–0·736)
Saint Lucia	4·75 (4–5·71)	0·893 (–0·207–2·32)	5·64 (4·32–7·45)	18·8 (–4·4–47·6)	0·952 (0·63–1·36)	0·179 (–0·0424–0·477)	1·13 (0·71–1·7)
Saint Vincent and the Grenadines	2·99 (2·51–3·58)	0·547 (–0·206–1·38)	3·54 (2·64–4·61)	18·3 (–7·0–46·2)	0·601 (0·399–0·849)	0·11 (–0·0449–0·3)	0·712 (0·439–1·08)
Suriname	22·5 (19–26·7)	5·88 (0·381–12·4)	28·4 (21·3–36·8)	26·2 (1·8–54·8)	4·52 (3–6·48)	1·18 (0·0749–2·56)	5·69 (3·56–8·2)
Trinidad and Tobago	44·7 (37·5–52·6)	9·07 (–1·93–23·1)	53·7 (40·5–71·2)	20·3 (–4·3–52·2)	8·95 (6·05–12·7)	1·83 (–0·378–4·87)	10·8 (6·65–16·2)
Virgin Islands	2·92 (2·44–3·48)	0·362 (–0·318–1·15)	3·29 (2·45–4·34)	12·4 (–11·0–39·1)	0·58 (0·385–0·826)	0·0715 (–0·0659–0·243)	0·651 (0·413–0·974)
**Central Latin America**	**6190 (5300–7220)**	**2460 (1930–3150)**	**8650 (7360–10200)**	**39·8 (32·8–47·4)**	**1250 (843–1740)**	**497 (326–723)**	**1740 (1170–2460)**
Colombia	720 (601–868)	258 (40·7–509)	977 (719–1320)	35·8 (5·6–69·2)	146 (97·2–212)	52·2 (7·82–110)	198 (122–296)
Costa Rica	128 (107–152)	44·9 (8·47–87·4)	172 (129–226)	35·2 (6·7–68·7)	25·7 (17·1–36·3)	9·08 (1·63–19·9)	34·8 (21·3–53·2)
El Salvador	167 (138–202)	44·3 (2·91–101)	211 (156–282)	26·5 (1·8–59·3)	33·7 (22·5–48·5)	8·91 (0·577–20·7)	42·6 (26·2–63·8)
Guatemala	405 (335–490)	169 (50·7–322)	573 (425–765)	41·7 (11·9–77·0)	81·4 (54·7–115)	33·9 (9·43–68·9)	115 (72·2–174)
Honduras	202 (166–247)	103 (30·6–190)	305 (222–417)	50·7 (16·0–89·3)	40·9 (27–57·8)	20·7 (5·94–40)	61·6 (37·8–92·9)
Mexico	3590 (3110–4140)	1510 (1180–1890)	5100 (4380–5940)	42·2 (34·8–50·6)	723 (491–999)	305 (193–444)	1030 (689–1430)
Nicaragua	155 (128–186)	45·6 (6·43–94·1)	201 (149–265)	29·4 (4·1–58·0)	31·5 (21·1–45·9)	9·29 (1·21–20·6)	40·8 (25–60·4)
Panama	92·8 (77–113)	52·2 (18·8–93·9)	145 (104–194)	56·3 (20·7–99·7)	18·8 (12·6–26·6)	10·6 (3·58–20·4)	29·3 (17·7–44·8)
Venezuela	734 (612–877)	233 (26·6–470)	967 (716–1280)	31·8 (3·7–64·9)	148 (98·5–210)	47 (5·07–105)	195 (120–297)
**Tropical Latin America**	**6720 (5970–7590)**	**1960 (1320–2600)**	**8680 (7500–9930)**	**29·1 (20·5–38·2)**	**1340 (929–1850)**	**390 (232–588)**	**1730 (1190–2390)**
Brazil	6560 (5820–7400)	1900 (1270–2530)	8460 (7330–9670)	29·0 (20·3–38·2)	1310 (907–1800)	379 (222–575)	1690 (1160–2320)
Paraguay	161 (134–194)	56·7 (11·4–112)	218 (161–290)	35·1 (7·2–68·1)	32·6 (21·9–46·2)	11·5 (2·17–24·8)	44·1 (27·3–67)
**North Africa and Middle East**	**20700 (17200–25100)**	**7710 (5590–10300)**	**28400 (23300–34800)**	**37·2 (29·5–46·0)**	**4210 (2810–5970)**	**1560 (971–2340)**	**5770 (3860–8190)**
Afghanistan	1230 (983–1530)	435 (90·7–837)	1660 (1170–2220)	35·4 (7·3–68·5)	249 (164–362)	88·6 (16·7–188)	338 (201–522)
Algeria	1360 (1110–1660)	347 (8·69–754)	1710 (1250–2270)	25·5 (0·6–54·1)	276 (182–396)	70·7 (1·51–168)	347 (213–526)
Bahrain	61·6 (50·4–75)	17·6 (2·25–37·4)	79·1 (57·9–105)	28·5 (3·7–57·9)	12·5 (8·36–17·9)	3·56 (0·414–8·05)	16·1 (9·96–24)
Egypt	2780 (2250–3420)	1190 (393–2250)	3970 (2910–5430)	42·8 (15·2–75·4)	567 (377–810)	242 (72·6–483)	809 (494–1230)
Iran	3430 (2820–4160)	1610 (1230–2080)	5050 (4130–6140)	47·0 (38·4–56·0)	692 (467–983)	325 (203–480)	1020 (674–1430)
Iraq	1240 (1010–1520)	518 (139–973)	1760 (1260–2350)	41·7 (10·9–76·4)	252 (167–362)	105 (27–208)	357 (218–532)

Jordan	346 (278–428)	89·1 (0·884–195)	435 (318–588)	25·8 (0·3–53·2)	70·7 (46·2–101)	18·2 (0·152–40·9)	88·9 (54·3–133)
Kuwait	161 (128–199)	58·8 (13·4–113)	220 (161–289)	36·7 (8·1–67·9)	32·7 (21·5–47·3)	12 (2·61–24·6)	44·7 (27·4–68·2)
Lebanon	218 (180–266)	60·8 (4·73–123)	278 (201–372)	27·9 (2·1–57·5)	43·7 (28·9–62·9)	12·3 (0·838–27·5)	56 (34·1–85·5)
Libya	258 (207–317)	85·4 (13·2–173)	344 (251–457)	33·1 (4·9–64·8)	52·4 (34·9–74·8)	17·3 (2·52–38·4)	69·7 (42·7–106)
Morocco	1550 (1290–1880)	591 (141–1160)	2140 (1580–2830)	38·1 (9·2–72·3)	314 (210–442)	120 (28·3–250)	434 (276–656)
Oman	152 (123–190)	34·3 (–5·56–78·8)	186 (133–254)	22·5 (–3·5–52·2)	31·3 (20·5–45)	7·02 (–1·15–17)	38·3 (23·1–57)
Palestine	236 (190–290)	95·1 (27·4–177)	331 (242–439)	40·3 (11·6–73·0)	48·2 (31·7–69)	19·4 (5·19–38·7)	67·6 (40·9–101)
Qatar	108 (85·3–136)	39·7 (9·16–77·7)	147 (105–201)	37·0 (8·2–72·4)	22·2 (14·3–31·8)	8·16 (1·71–17·2)	30·3 (18·7–46·1)
Saudi Arabia	1300 (1050–1590)	491 (107–935)	1790 (1250–2430)	37·8 (8·4–71·9)	264 (176–375)	99·9 (20·5–201)	364 (215–556)
Sudan	1230 (990–1510)	413 (88·8–827)	1640 (1200–2270)	33·6 (7·2–64·7)	251 (164–362)	84·3 (17–187)	336 (205–510)
Syria	462 (371–567)	106 (–11·7–249)	569 (418–745)	23·0 (–2·8–52·2)	93·3 (61·4–132)	21·5 (–2·11–53·5)	115 (69·2–176)
Tunisia	542 (448–652)	173 (23·3–359)	715 (535–947)	31·9 (4·4–65·1)	109 (73·4–155)	34·8 (4·09–75·1)	144 (91·3–219)
Turkey	2660 (2190–3210)	1060 (257–2040)	3720 (2680–4960)	39·8 (10·3–74·6)	539 (362–772)	214 (51·5–438)	753 (477–1120)
United Arab Emirates	318 (247–406)	81·6 (–1·44–186)	400 (290–546)	25·7 (–0·5–54·3)	65 (42·3–95·2)	16·7 (–0·289–38·8)	81·7 (49–127)
Yemen	1070 (863–1340)	208 (–40·7–503)	1280 (947–1690)	19·4 (–3·8–47·1)	217 (144–315)	42·2 (–7·7–107)	259 (158–387)
**South Asia**	**48500 (42200–56500)**	**17500 (13900–21600)**	**66100 (56900–77100)**	**36·1 (29·7–42·8)**	**9740 (6640–13600)**	**3510 (2320–5080)**	**13300 (8990–18400)**
Bangladesh	5790 (4840–6940)	2240 (505–4440)	8030 (5920–10700)	38·7 (8·8–74·7)	1170 (783–1640)	452 (97·9–956)	1620 (1010–2500)
Bhutan	22·2 (18·4–26·9)	3·19 (–2·36–9·88)	25·4 (18·6–33·8)	14·4 (–10·5–44·1)	4·47 (2·96–6·35)	0·643 (–0·454–2·06)	5·12 (3·21–7·66)
India	36100 (31500–41800)	12600 (9970–15700)	48700 (42100–56900)	35·0 (28·5–41·9)	7220 (4950–9990)	2520 (1640–3650)	9740 (6650–13600)
Nepal	1130 (963–1310)	404 (90·4–774)	1530 (1140–2010)	35·8 (7·9–66·8)	226 (152–318)	81·3 (14·9–165)	307 (186–456)
Pakistan	5550 (4650–6610)	2280 (1270–3520)	7830 (6300–9800)	41·1 (23·1–62·8)	1120 (758–1580)	461 (224–790)	1580 (1030–2310)
**Southeast Asia, east Asia, and Oceania**	**36800 (32100–42200)**	**4220 (2620–6060)**	**41000 (35700–47300)**	**11·5 (7·2–16·0)**	**7420 (5090–10400)**	**850 (458–1350)**	**8270 (5630–11600)**
**East Asia**	**27300 (23900–31400)**	**2390 (928–3960)**	**29700 (25700–34300)**	**8·7 (3·5–14·1)**	**5480 (3730–7710)**	**478 (182–869)**	**5960 (4090–8400)**
China	26600 (23300–30500)	2350 (884–3860)	28900 (25000–33200)	8·9 (3·3–14·5)	5330 (3630–7490)	471 (179–856)	5800 (3980–8180)
North Korea	399 (338–476)	20·4 (–69·2–137)	420 (307–562)	5·1 (–17·5–34·0)	80·7 (53·4–114)	4·03 (–14·6–28)	84·7 (53–126)
Taiwan (province of China)	356 (301–422)	16 (–68·9–114)	372 (270–495)	4·5 (–19·9–33·5)	71·3 (47·1–102)	3·3 (–13·7–23·6)	74·6 (45·2–113)
**Oceania**	**209 (172–256)**	**3·41 (–36·9–52·3)**	**213 (163–281)**	**1·7 (–17·6–24·0)**	**42·9 (28·6–62·4)**	**0·715 (–7·62–11·3)**	**43·7 (27·6–65·1)**
American Samoa	0·705 (0·588–0·858)	0·0771 (–0·0831–0·279)	0·783 (0·58–1·04)	10·9 (–12·3–38·6)	0·143 (0·0959–0·204)	0·0156 (–0·0174–0·0582)	0·159 (0·0981–0·238)
Cook Islands	0·355 (0·286–0·441)	0·0353 (–0·0483–0·139)	0·39 (0·279–0·536)	9·9 (–13·4–37·6)	0·0712 (0·0479–0·103)	0·00714 (–0·00901–0·0291)	0·0784 (0·0478–0·121)
Federated States of Micronesia	1·55 (1·28–1·89)	0·0454 (–0·325–0·499)	1·6 (1·16–2·14)	3·0 (–20·8–31·8)	0·318 (0·212–0·455)	0·00958 (–0·0705–0·107)	0·328 (0·196–0·513)
Fiji	14·2 (11·8–17)	1·14 (–2·37–4·8)	15·3 (11·2–20·3)	8·1 (–16·1–32·9)	2·89 (1·95–4·12)	0·233 (–0·507–1·01)	3·12 (1·93–4·73)

Guam	3·15 (2·66–3·79)	0·799 (–0·00307–1·79)	3·95 (2·94–5·27)	25·3 (–0·1–55·5)	0·646 (0·434–0·922)	0·164 (–0·000748–0·389)	0·81 (0·493–1·22)
Kiribati	1·75 (1·43–2·16)	0·0887 (–0·328–0·552)	1·84 (1·36–2·48)	5·1 (–18·0–32·1)	0·358 (0·241–0·513)	0·0183 (–0·0662–0·116)	0·376 (0·229–0·578)
Marshall Islands	0·824 (0·675–1)	0·0307 (–0·161–0·277)	0·855 (0·616–1·16)	3·7 (–19·0–31·3)	0·168 (0·113–0·243)	0·00634 (–0·0314–0·0581)	0·175 (0·107–0·272)
Nauru	0·179 (0·138–0·231)	0·0217 (–0·0226–0·0708)	0·2 (0·138–0·281)	12·1 (–12·7–38·6)	0·0366 (0·0235–0·0545)	0·00441 (–0·00497–0·0152)	0·041 (0·0241–0·0639)
Niue	0·0324 (0·026–0·0403)	0·00306 (–0·00476–0·0129)	0·0355 (0·0256–0·0478)	9·5 (–14·5–38·0)	0·00652 (0·00436–0·00961)	0·00062 (–9e–04–0·00262)	0·00715 (0·00442–0·0112)
Northern Mariana Islands	0·7 (0·58–0·834)	0·138 (–0·0303–0·351)	0·838 (0·628–1·11)	19·8 (–4·1–48·6)	0·142 (0·0953–0·202)	0·0281 (–0·00557–0·0746)	0·17 (0·106–0·258)
Palau	0·369 (0·291–0·464)	0·0296 (–0·054–0·138)	0·398 (0·283–0·538)	8·1 (–15·1–36·4)	0·0743 (0·049–0·109)	0·00592 (–0·0109–0·0278)	0·0803 (0·0489–0·124)
Papua New Guinea	157 (128–193)	0·32 (–38·5–44·5)	158 (114–216)	0·3 (–24·7–28·6)	32·3 (21·6–46·9)	0·0796 (–7·95–9·76)	32·4 (19·8–49·5)
Samoa	2·75 (2·29–3·38)	0·0548 (–0·611–0·803)	2·81 (2·05–3·82)	2·0 (–21·2–29·8)	0·563 (0·369–0·807)	0·0114 (–0·125–0·181)	0·574 (0·352–0·884)
Solomon Islands	9·65 (7·82–11·9)	0·225 (–2·07–2·8)	9·87 (7·01–13·9)	2·3 (–20·9–30·0)	1·98 (1·32–2·89)	0·0464 (–0·431–0·663)	2·03 (1·25–3·16)
Tokelau	0·0247 (0·0198–0·031)	0·00259 (–0·00332–0·00948)	0·0273 (0·0193–0·0371)	10·4 (–13·3–37·7)	0·00502 (0·00331–0·00735)	0·000529 (–0·000683–0·00204)	0·00555 (0·00331–0·00863)
Tonga	1·26 (1·05–1·54)	0·141 (–0·17–0·503)	1·4 (1·01–1·87)	11·2 (–14·4–38·7)	0·257 (0·172–0·369)	0·0288 (–0·0351–0·106)	0·286 (0·174–0·435)
Tuvalu	0·216 (0·168–0·273)	0·0219 (–0·0272–0·0781)	0·238 (0·168–0·326)	10·1 (–13·4–35·0)	0·044 (0·0291–0·0655)	0·00445 (–0·00537–0·0164)	0·0485 (0·0295–0·0741)
Vanuatu	4·37 (3·61–5·36)	0·0749 (–0·97–1·32)	4·44 (3·16–6·26)	1·6 (–22·6–28·8)	0·897 (0·604–1·31)	0·0147 (–0·2–0·274)	0·912 (0·551–1·42)
**Southeast Asia**	**9250 (7880–10900)**	**1830 (1220–2570)**	**11100 (9270–13300)**	**19·7 (13·5–26·1)**	**1890 (1280–2660)**	**374 (212–583)**	**2270 (1520–3200)**
Cambodia	265 (222–319)	30·2 (–34·1–110)	295 (218–396)	11·4 (–11·5–41·6)	54·4 (36–77·9)	6·24 (–6·63–23·8)	60·6 (37–93)
Indonesia	3090 (2590–3640)	685 (423–1000)	3770 (3130–4540)	22·2 (14·0–31·0)	635 (433–899)	141 (75·6–224)	776 (527–1100)
Laos	99·7 (82·3–121)	15·3 (–9·2–45·4)	115 (83·9–154)	15·4 (–9·6–44·3)	20·6 (13·8–29·6)	3·17 (–1·91–9·95)	23·8 (14·5–34·9)
Malaysia	656 (547–793)	88·9 (–60·5–276)	745 (540–979)	13·5 (–8·8–42·6)	134 (90–191)	18·2 (–11·8–59·5)	152 (95·4–229)
Maldives	8·85 (7·31–10·7)	2·38 (0·227–5)	11·2 (8·35–15)	26·8 (2·6–55·0)	1·82 (1·22–2·55)	0·491 (0·0414–1·16)	2·32 (1·46–3·59)
Mauritius	37·7 (31·6–45)	5·15 (–3·94–15·5)	42·8 (31·9–55·9)	13·7 (–10·6–41·6)	7·58 (5·02–10·8)	1·04 (–0·775–3·26)	8·62 (5·45–13)
Myanmar	505 (410–620)	141 (6·61–302)	647 (472–866)	27·9 (1·4–56·8)	103 (68·7–148)	28·8 (1·24–66·8)	132 (80·7–198)
Philippines	1740 (1460–2050)	610 (473–769)	2350 (1960–2790)	35·1 (29·5–41·0)	356 (243–503)	125 (82·3–180)	481 (328–679)
Seychelles	1·52 (1·27–1·82)	0·268 (–0·103–0·742)	1·78 (1·31–2·4)	17·6 (–7·0–47·2)	0·309 (0·206–0·44)	0·0547 (–0·0209–0·16)	0·364 (0·225–0·561)
Sri Lanka	359 (306–422)	71·2 (–13·8–176)	430 (329–553)	19·9 (–4·1–47·2)	73·1 (49·2–103)	14·6 (–2·26–38·5)	87·7 (55·2–132)
Thailand	1330 (1120–1590)	94 (–209–486)	1420 (1040–1920)	7·1 (–16·4–36·5)	267 (181–382)	18·9 (–45·3–102)	286 (176–442)
Timor–Leste	17·5 (14·3–21·5)	2·67 (–1·47–7·9)	20·1 (14·6–27·5)	15·3 (–8·9–45·2)	3·59 (2·4–5·15)	0·549 (–0·309–1·77)	4·14 (2·62–6·32)
Vietnam	1140 (946–1370)	78·6 (–199–412)	1220 (872–1630)	6·9 (–17·8–35·3)	233 (154–332)	16·1 (–42·9–90·8)	249 (149–377)

**Sub–Saharan Africa**	**26800 (22600–32100)**	**6160 (4660–7960)**	**32900 (27700–39500)**	**23·0 (18·3–27·9)**	**5430 (3680–7660)**	**1250 (785–1860)**	**6680 (4500–9440)**
**Central sub–Saharan Africa**	**4620 (3780–5630)**	**1020 (226–1980)**	**5640 (4390–7250)**	**22·0 (4·9–41·9)**	**939 (626–1350)**	**207 (41·9–432)**	**1150 (727–1660)**
Angola	1080 (876–1320)	233 (–28·2–528)	1310 (973–1710)	21·7 (–2·7–47·8)	220 (146–320)	47·3 (–5·84–112)	267 (163–397)
Central African Republic	211 (173–259)	37·4 (–14–100)	249 (179–335)	17·7 (–6·1–46·0)	42·7 (28·5–61·6)	7·57 (–2·68–21·2)	50·3 (30·3–79·1)
Congo	208 (170–250)	48 (–1·45–113)	256 (190–344)	23·1 (–0·6–54·3)	42·2 (27·9–60·7)	9·74 (–0·257–25·2)	51·9 (31·8–78·9)
Democratic Republic of the Congo	3000 (2440–3650)	673 (–56·8–1550)	3670 (2680–4920)	22·4 (–2·2–50·9)	608 (405–877)	137 (–7·47–339)	745 (454–1120)
Equatorial Guinea	57 (46–70·3)	12·6 (–0·53–30·4)	69·6 (51·2–92·6)	22·1 (–1·0–51·1)	11·6 (7·72–16·7)	2·58 (–0·0862–6·45)	14·2 (8·73–21·6)
Gabon	72 (59·2–86·9)	13·1 (–3·74–33·7)	85·1 (64·1–113)	18·1 (–5·5–45·7)	14·6 (9·69–20·7)	2·65 (–0·771–7·21)	17·2 (10·7–25·7)
**Eastern sub–Saharan Africa**	**10600 (8920–12700)**	**2180 (1410–3060)**	**12800 (10600–15400)**	**20·5 (13·5–27·4)**	**2160 (1460–3030)**	**442 (252–702)**	**2600 (1750–3660)**
Burundi	288 (235–351)	28·4 (–36·7–112)	317 (235–432)	9·8 (–12·5–36·7)	58·5 (38·5–83·3)	5·74 (–7·53–24·1)	64·2 (39·5–98)
Comoros	19 (15·7–23)	4·19 (–0·358–9·8)	23·2 (17·6–31)	22·0 (–1·8–49·4)	3·87 (2·56–5·56)	0·848 (–0·0814–2·04)	4·71 (3·03–7·05)
Djibouti	33 (26·8–40·4)	5·95 (–1·57–15·5)	39 (28·4–51·9)	18·0 (–4·5–45·6)	6·75 (4·4–9·8)	1·22 (–0·302–3·23)	7·96 (4·95–12·1)
Eritrea	178 (145–218)	17·7 (–21·8–62·6)	196 (147–259)	10·1 (–11·8–34·8)	36·2 (23·7–51·9)	3·61 (–4·41–13·5)	39·8 (24·7–59·5)
Ethiopia	2600 (2190–3090)	534 (200–899)	3130 (2570–3820)	20·6 (7·7–33·9)	530 (361–753)	109 (39·1–201)	639 (424–908)
Kenya	1310 (1130–1520)	444 (351–553)	1750 (1500–2050)	34·0 (28·7–39·6)	266 (181–368)	90·3 (59·8–132)	356 (247–500)
Madagascar	710 (582–866)	136 (–48–337)	846 (615–1150)	19·2 (–6·7–46·2)	145 (96·9–208)	27·8 (–9·5–74·7)	173 (103–261)
Malawi	378 (310–461)	75·2 (–22·9–192)	453 (332–604)	19·9 (–6·0–49·9)	77·1 (51–110)	15·4 (–4·06–40·3)	92·5 (55·7–139)
Mozambique	737 (608–892)	131 (–46·5–369)	868 (641–1150)	17·7 (–6·3–48·2)	148 (98–211)	26·1 (–8·79–73·9)	174 (106–255)
Rwanda	374 (307–457)	62 (–24–168)	436 (317–580)	16·6 (–5·9–44·4)	75·8 (50·2–108)	12·5 (–4·79–36·1)	88·3 (55·3–133)
Somalia	482 (392–601)	85·5 (–28·2–225)	568 (423–757)	17·8 (–6·0–44·9)	98·2 (64·7–141)	17·3 (–5·35–46·5)	116 (70·7–174)
South Sudan	232 (192–284)	33·8 (–19·7–100)	265 (194–353)	14·6 (–8·5–41·2)	46·8 (31·2–66)	6·79 (–3·72–20·3)	53·5 (33·8–78·3)
Uganda	1490 (1210–1840)	317 (–29·6–779)	1810 (1330–2410)	21·3 (–2·2–49·2)	305 (202–433)	64·5 (–6·54–166)	369 (232–566)
Tanzania	1390 (1140–1690)	249 (–67·4–636)	1630 (1220–2160)	18·0 (–4·9–45·5)	282 (185–399)	50·6 (–13·3–133)	332 (205–497)
Zambia	385 (315–469)	48·2 (–42·4–160)	434 (320–576)	12·5 (–11·2–40·8)	78·3 (51·5–113)	9·81 (–8·49–34·3)	88·1 (54·2–133)
**Southern sub–Saharan Africa**	**2210 (1910–2590)**	**840 (585–1130)**	**3050 (2560–3630)**	**38·0 (27·1–48·6)**	**442 (305–610)**	**168 (101–260)**	**609 (412–862)**
Botswana	67·7 (55·8–82·8)	8·64 (–6·45–27·3)	76·4 (56·2–102)	12·7 (–9·7–40·4)	13·6 (9·07–19·5)	1·74 (–1·26–5·7)	15·3 (9·54–23·3)
Eswatini	32·1 (26·3–38·9)	11·6 (2·78–23·3)	43·7 (32–59·1)	36·2 (8·5–68·6)	6·43 (4·33–9·23)	2·33 (0·481–5)	8·76 (5·41–13·5)
Lesotho	74·3 (61·2–90·3)	18·3 (–1·14–39·4)	92·6 (68·6–122)	24·6 (–1·5–54·0)	14·8 (9·99–21)	3·63 (–0·206–8·44)	18·4 (11·7–27·6)
Namibia	51·5 (42·6–61·9)	15·1 (0·874–31·6)	66·6 (48·7–88·8)	29·2 (1·7–59·9)	10·4 (7·05–14·9)	3·05 (0·2–6·6)	13·5 (8·35–20·2)
South Africa	1740 (1520–2020)	743 (499–1010)	2490 (2080–2940)	42·7 (30·0–55·6)	347 (240–482)	148 (86·3–231)	496 (336–701)

Zimbabwe	240 (196–294)	47 (–10·3–122)	287 (210–376)	19·6 (–4·9–47·4)	48·4 (32·4–70·4)	9·47 (–2·19–24·6)	57·9 (35·6–87·9)
**Western sub–Saharan Africa**	**9320 (7830–11200)**	**2130 (1590–2800)**	**11400 (9620–13700)**	**22·8 (17·8–27·9)**	**1890 (1280–2660)**	**430 (269–635)**	**2320 (1570–3310)**
Benin	286 (235–348)	29·3 (–37·9–109)	315 (230–424)	10·3 (–13·2–36·8)	58·1 (38·1–82·3)	5·92 (–8·04–23·3)	64 (39·4–95·9)
Burkina Faso	504 (415–612)	58·8 (–55·7–192)	563 (418–759)	11·7 (–11·5–37·9)	103 (68·2–147)	12 (–11·4–41)	115 (72·2–174)
Cabo Verde	20·4 (17–24·5)	9·46 (2·57–17)	29·8 (21·9–38·9)	46·5 (12·1–82·5)	4·13 (2·78–5·77)	1·91 (0·516–3·55)	6·05 (3·73–9·07)
Cameroon	749 (612–916)	88·9 (–85·6–305)	837 (603–1120)	11·9 (–11·1–38·6)	152 (101–217)	18·1 (–17·5–59·5)	170 (103–251)
Chad	419 (340–516)	60·5 (–34·1–178)	480 (354–645)	14·5 (–8·7–41·4)	85·2 (56·6–121)	12·3 (–7·67–37·9)	97·5 (60·1–146)
Côte d’Ivoire	524 (427–637)	64·6 (–59·5–214)	589 (434–806)	12·3 (–11·6–40·7)	107 (70·6–153)	13·2 (–12·5–46·1)	120 (73·9–184)
The Gambia	74·4 (60·6–91)	14·4 (–3·59–36·4)	88·8 (64·9–120)	19·4 (–4·8–46·4)	15 (9·93–21·4)	2·92 (–0·671–7·54)	17·9 (11·2–27·2)
Ghana	829 (682–1010)	143 (–48·9–381)	972 (728–1290)	17·2 (–6·0–45·1)	169 (111–244)	28·9 (–9·34–81·3)	197 (122–297)
Guinea	285 (235–348)	48·6 (–21·6–133)	334 (248–438)	17·1 (–7·3–47·3)	57·9 (38·9–82·4)	9·66 (–4·95–27·1)	67·5 (43·4–101)
Guinea–Bissau	45·8 (37·4–56·4)	7·97 (–3·05–22)	53·8 (39·8–72·1)	17·4 (–7·1–47·3)	9·3 (6·16–13·3)	1·62 (–0·665–4·66)	10·9 (6·9–16·4)
Liberia	128 (104–157)	21·8 (–8·03–63·1)	150 (111–202)	17·1 (–6·4–46·6)	25·4 (16·8–35·9)	4·36 (–1·55–12·9)	29·8 (18·2–45·4)
Mali	351 (286–432)	71·7 (–15·8–173)	423 (305–565)	20·3 (–4·8–48·9)	71·5 (47·4–102)	14·6 (–3·18–38·8)	86·1 (52·8–130)
Mauritania	77·2 (63·7–94·2)	17·8 (–1·47–39·5)	95 (71·1–126)	23·1 (–1·8–51·6)	15·8 (10·4–22·6)	3·65 (–0·263–8·6)	19·4 (11·9–29·4)
Niger	435 (357–537)	49·6 (–55·1–173)	485 (356–645)	11·4 (–13·0–37·9)	88·9 (58·8–128)	10 (–11·4–36·8)	98·9 (60·8–149)
Nigeria	3850 (3260–4540)	1280 (1010–1620)	5140 (4330–6120)	33·3 (27·8–39·0)	781 (535–1100)	260 (170–375)	1040 (705–1440)
São Tomé and Príncipe	4·35 (3·55–5·37)	0·901 (–0·195–2·23)	5·25 (3·8–7·05)	20·7 (–4·5–48·8)	0·889 (0·585–1·29)	0·184 (–0·0374–0·481)	1·07 (0·664–1·65)
Senegal	325 (270–397)	94·6 (7·72–204)	420 (313–561)	29·1 (2·6–59·8)	65·9 (43·8–94)	19·1 (1·53–41·1)	85 (52·6–126)
Sierra Leone	203 (167–250)	32 (–17·7–87·3)	235 (173–310)	15·8 (–9·3–43·4)	41·2 (27·3–59·3)	6·46 (–3·74–19·5)	47·7 (29·9–71·2)
Togo	202 (166–245)	27·3 (–18·6–83·6)	229 (171–306)	13·6 (–9·0–41·7)	41·1 (27·1–58·5)	5·52 (–3·86–17·3)	46·6 (29·5–69·9)

Table S8: Prevalence and DALYs of Anxiety disorders per 100 000 persons, with 95% uncertainty intervals, by location, for the year 2020

Location	Baseline prevalence	Additional prevalence	Final prevalence	% change	Baseline DALYs	Additional DALYs	Final DALYs
**Global**	**3824·9 (3283·3–4468·1)**	**977·5 (824·8–1161·6)**	**4802·4 (4108·2–5588·6)**	**25·6 (23·2–28·0)**	**454·8 (307·0–642·5)**	**116·1 (79·3–163·8)**	**570·9 (387·3–802·2)**
**Central Europe, eastern Europe, and central Asia**	**3274·3 (2801·2–3821·9)**	**981·0 (774·1–1214·4)**	**4255·3 (3593·1–4970·8)**	**30·0 (24·9–35·0)**	**385·5 (259·6–543·8)**	**115·4 (76·5–166·5)**	**500·9 (338·5–703·0)**
**Central Asia**	**2087·1 (1688·0–2594·0)**	**696·3 (392·6–1037·9)**	**2783·4 (2158·9–3493·7)**	**33·4 (19·1–47·9)**	**250·9 (161·6–363·3)**	**83·5 (39·8–137·8)**	**334·4 (215·4–486·0)**
Armenia	2744·5 (2222·5–3389·6)	1046·9 (217·4–2091·9)	3791·5 (2736·8–5119·3)	38·2 (7·7–74·3)	326·7 (216·4–474·2)	124·4 (24·6–263·3)	451·1 (270·7–674·7)
Azerbaijan	2363·5 (1898·2–2923·0)	757·0 (25·4–1665·9)	3120·5 (2139·7–4248·1)	32·0 (1·1–69·0)	284·0 (186·9–411·6)	91·0 (2·9–214·9)	375·0 (223·6–583·1)
Georgia	2414·6 (1958·3–2945·8)	750·4 (56·0–1624·5)	3165·1 (2259·7–4297·0)	31·2 (2·4–65·8)	285·5 (188·2–413·6)	88·6 (7·5–204·7)	374·1 (225·8–562·9)
Kazakhstan	2059·4 (1665·9–2541·7)	698·0 (92·0–1467·0)	2757·4 (1978·3–3745·6)	34·0 (4·1–69·3)	246·1 (160·1–356·0)	83·3 (10·2–182·8)	329·4 (202·0–512·2)
Kyrgyzstan	1965·6 (1576·8–2433·6)	948·1 (260·4–1804·4)	2913·7 (2083·1–4076·7)	48·2 (14·3–90·0)	237·2 (151·2–346·4)	113·7 (29·1–224·7)	350·9 (210·2–534·8)
Mongolia	2020·6 (1610·0–2535·2)	35·9 (–444·6–585·7)	2056·5 (1446·5–2840·1)	1·8 (–22·3–28·8)	242·5 (156·1–343·6)	4·2 (–55·7–75·1)	246·8 (144·4–370·0)
Tajikistan	2114·8 (1689·6–2692·9)	698·7 (82·3–1463·5)	2813·5 (2008·2–3887·9)	33·0 (3·7–67·6)	256·0 (164·5–371·6)	84·5 (8·3–187·7)	340·5 (203·1–532·8)
Turkmenistan	2151·7 (1714·8–2672·0)	751·3 (75·5–1562·6)	2903·0 (2097·0–3987·6)	35·0 (3·4–70·8)	259·3 (169·9–376·9)	90·7 (8·2–201·9)	350·0 (212·5–546·8)
Uzbekistan	1948·8 (1549·1–2445·3)	652·3 (74·2–1375·0)	2601·1 (1809·5–3605·9)	33·5 (3·8–67·7)	235·1 (149·8–346·8)	78·4 (8·1–173·2)	313·5 (188·9–479·3)
**Central Europe**	**3681·1 (3084·0–4381·6)**	**989·9 (670·0–1379·8)**	**4671·0 (3863·3–5668·9)**	**26·9 (18·9–34·9)**	**432·0 (288·8–621·3)**	**116·1 (66·9–179·8)**	**548·1 (368·2–780·6)**
Albania	3933·8 (3176·0–4803·1)	1431·1 (250·0–2891·0)	5364·9 (3895·4–7147·6)	36·5 (6·1–71·5)	466·4 (308·9–674·1)	169·9 (26·3–361·7)	636·3 (392·7–979·5)
Bosnia and Herzegovina	3874·0 (3148·3–4797·4)	1119·7 (67·5–2543·9)	4993·7 (3575·3–6794·2)	28·9 (1·8–63·6)	453·6 (303·0–654·5)	131·1 (7·8–311·0)	584·7 (365·8–888·7)
Bulgaria	3911·0 (3169·7–4799·9)	1004·8 (–43·4–2293·2)	4915·8 (3569·2–6630·4)	25·7 (–1·2–57·6)	457·6 (304·6–663·7)	117·3 (–4·9–292·2)	575·0 (358·3–884·3)
Croatia	3929·4 (3191·0–4812·0)	862·4 (–209·0–2114·3)	4791·8 (3408·4–6564·0)	22·0 (–5·5–52·1)	460·0 (306·8–667·2)	100·6 (–24·7–258·3)	560·5 (345·0–854·8)
Czechia	3592·3 (2922·5–4404·2)	952·2 (–29·3–2270·0)	4544·5 (3304·3–6271·2)	26·5 (–0·8–61·6)	419·4 (274·3–600·9)	111·2 (–4·1–264·8)	530·6 (319·1–800·5)
Hungary	3815·7 (3098·3–4647·4)	623·9 (–351·5–1791·8)	4439·6 (3196·8–6068·3)	16·3 (–9·2–46·8)	445·4 (298·8–649·5)	73·1 (–39·8–223·4)	518·4 (315·2–810·0)
Montenegro	3839·6 (3111·9–4699·3)	1272·2 (118·0–2669·5)	5111·8 (3585·5–6821·1)	33·2 (3·2–69·0)	453·4 (298·9–651·9)	150·5 (13·3–347·6)	603·9 (358·7–913·9)
North Macedonia	3847·0 (3142·7–4708·6)	1406·8 (259·4–2913·2)	5253·8 (3758·5–7128·9)	36·6 (7·1–73·8)	454·3 (297·6–657·5)	166·4 (30·2–362·0)	620·7 (381·7–959·7)
Poland	3589·0 (3109·0–4127·9)	906·1 (608·8–1226·8)	4495·1 (3860·6–5206·8)	25·3 (17·5–33·8)	421·4 (286·6–591·5)	106·3 (63·0–165·5)	527·7 (361·1–729·0)
Romania	3581·9 (2902·6–4413·3)	1239·1 (95·6–2629·1)	4820·9 (3421·3–6580·1)	34·6 (3·2–69·1)	421·3 (278·4–612·3)	145·5 (9·7–332·1)	566·9 (339·8–868·3)
Serbia	3697·4 (3001·6–4508·8)	1031·0 (22·2–2474·8)	4728·4 (3462·3–6402·7)	27·9 (0·6–64·7)	435·1 (285·8–631·6)	121·3 (2·5–297·2)	556·4 (341·8–840·3)
Slovakia	3758·2 (3055·6–4589·0)	949·8 (–40·5–2026·5)	4708·1 (3507·6–6309·1)	25·3 (–1·1–54·1)	442·4 (291·6–639·5)	112·3 (–4·3–259·6)	554·6 (344·2–854·1)
Slovenia	3741·6 (3057·6–4562·5)	990·4 (–58·2–2345·3)	4732·0 (3373·6–6480·1)	26·4 (–1·6–61·4)	437·3 (292·1–630·4)	115·7 (–6·4–292·9)	553·0 (334·1–860·7)
**Eastern Europe**	**3603·2 (3128·7–4130·9)**	**1108·2 (835·7–1427·2)**	**4711·4 (4038·1–5478·9)**	**30·8 (23·9–38·5)**	**422·5 (286·5–589·9)**	**129·8 (83·3–190·4)**	**552·3 (374·5–773·5)**
Belarus	3814·5 (3096·6–4700·2)	979·8 (–116·1–2379·6)	4794·3 (3425·5–6610·1)	25·6 (–3·1–60·0)	449·0 (296·0–645·6)	114·7 (–12·3–282·4)	563·6 (347·2–859·5)
Estonia	3766·9 (3063·4–4568·0)	659·1 (–366·3–1784·7)	4426·0 (3167·4–5947·5)	17·6 (–9·8–49·6)	441·7 (294·9–640·4)	77·5 (–47·3–228·1)	519·2 (317·1–795·4)
Latvia	4009·8 (3265·3–4839·9)	830·2 (–181·4–2162·2)	4840·0 (3492·7–6551·8)	20·7 (–4·7–54·5)	469·4 (311·7–671·0)	97·5 (–20·0–272·1)	566·9 (346·3–870·1)

Lithuania	4659·4 (3772·5–5649·5)	1348·8 (65·5–2844·7)	6008·2 (4326·8–8128·7)	29·0 (1·4–62·0)	545·3 (361·8–785·5)	157·7 (7·0–369·3)	703·1 (433·3–1055·8)
Republic of Moldova	4227·1 (3423·8–5211·2)	1592·6 (283·0–2998·4)	5819·7 (4088·9–7716·8)	37·8 (7·0–71·3)	499·2 (331·0–720·9)	188·6 (29·9–396·7)	687·8 (404·6–1059·2)
Russia	3597·1 (3129·3–4121·4)	1149·6 (945·6–1412·0)	4746·8 (4130·2–5460·8)	32·0 (26·9–37·3)	421·8 (285·7–594·0)	134·6 (90·0–193·0)	556·4 (379·8–776·4)
Ukraine	3438·1 (2990·0–3963·1)	968·0 (27·1–2102·5)	4406·2 (3324·5–5765·8)	28·2 (0·8–60·9)	402·6 (275·6–562·6)	113·3 (3·1–257·8)	515·9 (332·1–772·4)
**High–income**	**5356·8 (4609·1–6233·3)**	**1349·0 (1044·1–1678·8)**	**6705·7 (5773·4–7829·4)**	**25·2 (20·3–30·7)**	**627·7 (428·1–877·7)**	**158·0 (103·6–234·7)**	**785·7 (536·8–1101·1)**
**Australasia**	**6225·5 (5158·6–7610·0)**	**680·9 (–531·5–2118·3)**	**6906·4 (5209·8–9073·8)**	**10·9 (–8·7–34·8)**	**735·0 (488·5–1053·6)**	**80·9 (–61·7–278·2)**	**815·9 (516·0–1222·3)**
Australia	5975·6 (4887·8–7410·4)	696·1 (–713·8–2400·9)	6671·7 (4845·9–9038·0)	11·6 (–12·3–40·8)	705·6 (465·9–1018·4)	82·9 (–79·8–312·3)	788·5 (489·2–1221·6)
New Zealand	7485·8 (6443·1–8730·3)	585·8 (–777·2–2218·1)	8071·6 (6331·5–10221·4)	7·9 (–10·7–30·4)	883·4 (592·4–1222·2)	68·7 (–91·8–273·3)	952·1 (630·3–1388·4)
**High income Asia Pacific**	**2765·9 (2370·2–3231·2)**	**341·6 (68·3–672·6)**	**3107·5 (2582·1–3708·1)**	**12·4 (2·4–23·9)**	**329·2 (222·5–467·1)**	**40·7 (8·1–86·5)**	**369·9 (244·0–529·3)**
Brunei	3081·8 (2465·7–3823·4)	310·7 (–459·1–1241·0)	3392·5 (2404·5–4775·8)	10·1 (–15·4–40·3)	373·5 (240·1–544·0)	37·4 (–58·6–159·1)	410·9 (241·5–631·9)
Japan	2455·2 (2151·2–2780·3)	311·1 (182·9–460·7)	2766·3 (2387·1–3185·1)	12·7 (7·6–18·5)	291·0 (198·3–406·5)	36·9 (17·8–62·3)	328·0 (220·0–463·3)
Singapore	2984·8 (2399·0–3701·7)	539·8 (–237·9–1495·8)	3524·6 (2550·8–4780·6)	18·2 (–8·2–50·8)	359·7 (236·9–517·9)	65·2 (–30·8–194·5)	424·9 (256·3–655·6)
South Korea	3480·3 (2818·2–4295·8)	393·6 (–515·6–1456·8)	3873·9 (2745·3–5250·6)	11·3 (–14·3–41·0)	416·6 (274·2–605·7)	47·2 (–61·8–178·7)	463·8 (282·0–705·6)
**High income North America**	**5918·3 (5114·4–6866·2)**	**1643·8 (1243·0–2076·2)**	**7562·1 (6465·9–8780·2)**	**27·8 (21·7–34·1)**	**687·4 (468·7–961·2)**	**190·8 (121·5–282·7)**	**878·2 (598·1–1230·3)**
Canada	4539·3 (3665·8–5629·5)	948·5 (–233·4–2486·9)	5487·7 (3968·0–7472·6)	20·9 (–5·1–52·5)	535·6 (351·7–776·5)	111·7 (–24·6–299·7)	647·3 (395·6–1001·0)
Greenland	5127·5 (4064·6–6395·5)	893·8 (–420·2–2393·7)	6021·3 (4339·3–8069·3)	17·5 (–7·3–47·9)	605·1 (395·9–876·9)	105·8 (–43·1–316·7)	710·9 (435·3–1068·8)
USA	6072·0 (5260·5–7004·0)	1721·3 (1287·4–2189·8)	7793·3 (6670·2–9037·6)	28·4 (22·2–35·2)	704·4 (483·2–981·2)	199·5 (127·5–294·7)	904·0 (619·1–1267·9)
**Southern Latin America**	**5356·3 (4722·0–6055·4)**	**1972·2 (743·1–3244·6)**	**7328·5 (5851·2–8922·7)**	**36·8 (13·9–60·0)**	**636·8 (437·4–898·1)**	**234·2 (83·4–429·8)**	**871·0 (565·0–1257·4)**
Argentina	5171·3 (4648·1–5684·2)	1976·7 (375·1–3673·5)	7148·0 (5430·1–9103·9)	38·2 (7·2–70·9)	616·6 (427·0–862·5)	235·5 (45·0–484·5)	852·1 (538·6–1281·0)
Chile	5803·8 (4700·6–7134·7)	2165·2 (410·5–4262·1)	7969·1 (5889·3–10831·1)	37·4 (7·2–74·3)	685·7 (449·2–984·7)	255·4 (44·1–531·0)	941·1 (568·9–1439·6)
Uruguay	5419·3 (4414·9–6621·4)	887·4 (–551·2–2401·1)	6306·7 (4544·5–8518·1)	16·4 (–10·5–43·4)	642·1 (420·9–918·2)	105·4 (–65·2–307·8)	747·5 (447·5–1147·6)
**Western Europe**	**5935·3 (5000·5–7016·8)**	**1482·6 (963·6–2090·0)**	**7417·9 (6216·2–8868·3)**	**25·0 (16·6–34·1)**	**697·0 (472·7–976·3)**	**174·0 (99·5–271·1)**	**870·9 (585·8–1239·2)**
Andorra	5852·2 (4790·8–7172·8)	1950·7 (242·6–3873·8)	7802·9 (5625·3–10375·6)	33·4 (4·4–66·4)	690·6 (453·5–984·8)	229·2 (24·4–492·8)	919·8 (581·6–1369·1)
Austria	6173·3 (5040·4–7547·2)	1387·0 (–209·7–3177·0)	7560·3 (5594·5–9886·4)	22·5 (–3·1–51·5)	728·4 (491·9–1045·5)	163·0 (–21·9–379·1)	891·4 (543·8–1326·4)
Belgium	5360·8 (4358·2–6505·2)	1575·2 (–36·5–3436·7)	6936·1 (4952·8–9206·1)	29·3 (–0·7–60·8)	630·7 (419·3–902·8)	185·0 (–4·5–421·1)	815·7 (512·2–1253·5)
Cyprus	6326·8 (5109·0–7766·7)	1199·2 (–313·1–3039·4)	7526·0 (5452·3–9988·6)	18·9 (–5·0–46·6)	751·0 (496·5–1083·9)	142·6 (–36·8–382·6)	893·6 (557·3–1368·6)
Denmark	5396·8 (4419·3–6555·8)	991·8 (–402·3–2752·0)	6388·5 (4606·7–8722·2)	18·3 (–7·5–51·0)	637·4 (421·6–916·1)	116·1 (–48·6–339·4)	753·5 (458·2–1140·0)
Finland	4187·8 (3428·5–5064·8)	470·6 (–535·1–1713·2)	4658·4 (3281·6–6271·4)	11·2 (–12·6–40·0)	492·4 (325·5–698·3)	55·5 (–64·8–220·1)	547·9 (342·3–837·6)
France	6613·0 (5415·9–8002·3)	2159·6 (327·3–4409·7)	8772·7 (6380·2–11735·5)	32·7 (5·0–68·9)	777·2 (519·5–1103·2)	253·2 (36·7–576·1)	1030·4 (648·1–1583·2)
Germany	6650·9 (5407·5–8046·3)	1093·6 (–516·6–3111·8)	7744·5 (5728·7–10285·8)	16·5 (–8·0–46·7)	775·9 (513·8–1106·3)	127·9 (–58·2–385·0)	903·8 (544·6–1345·9)
Greece	5992·9 (4888·1–7219·5)	1179·6 (–354·3–3033·5)	7172·5 (5234·4–9598·7)	19·7 (–6·0–50·1)	704·0 (469·6–1007·1)	139·0 (–40·7–376·7)	842·9 (529·9–1255·2)
Iceland	5495·9 (4467·3–6630·8)	1046·3 (–353·6–2587·8)	6542·2 (4796·0–8628·3)	19·1 (–6·7–47·5)	653·1 (432·1–929·8)	124·5 (–41·1–342·6)	777·6 (473·7–1179·3)

Ireland	6720·1 (5441·2–8163·4)	1794·1 (–44·8–3833·4)	8514·2 (6100·0–11271·1)	26·7 (–0·7–57·6)	795·8 (528·5–1139·1)	211·6 (–5·8–477·5)	1007·4 (631·3–1493·1)
Israel	3801·4 (3122·7–4613·5)	1052·8 (–5·9–2427·4)	4854·2 (3534·4–6614·9)	27·7 (–0·1–62·6)	454·4 (302·4–650·0)	125·6 (–0·9–295·4)	580·0 (357·9–877·8)
Italy	5808·1 (5065·5–6603·5)	1427·6 (917·9–1993·6)	7235·7 (6209·1–8312·0)	24·6 (16·0–33·8)	681·0 (463·3–955·8)	167·0 (100·8–267·5)	848·0 (574·6–1187·5)
Luxembourg	5656·8 (4588·5–6911·6)	1559·8 (–3·6–3440·5)	7216·6 (5120·2–9710·0)	27·5 (–0·1–57·8)	669·0 (440·1–955·0)	184·2 (–0·5–409·4)	853·2 (512·3–1292·2)
Malta	6152·6 (4984·6–7514·5)	1174·9 (–451·4–2993·1)	7327·5 (5367·8–9725·0)	19·2 (–6·9–48·1)	725·7 (484·5–1045·4)	138·4 (–51·5–382·2)	864·1 (534·3–1315·4)
Monaco	5734·7 (4687·0–6961·4)	1242·7 (–231·8–2966·0)	6977·4 (5054·9–9173·3)	21·7 (–4·7–53·2)	673·2 (443·3–972·3)	145·5 (–26·1–356·7)	818·7 (498·6–1219·9)
Netherlands	6786·8 (5533·5–8182·5)	1845·7 (75·0–4056·9)	8632·5 (6377·9–11410·4)	27·3 (1·0–58·4)	801·7 (535·1–1146·5)	217·1 (8·3–501·1)	1018·8 (643·9–1514·3)
Norway	7106·9 (6188·5–8121·7)	1199·6 (549·2–1880·3)	8306·5 (7135·6–9729·2)	16·9 (7·8–25·9)	841·2 (576·2–1179·6)	141·9 (59·8–249·0)	983·1 (666·6–1373·0)
Portugal	8239·7 (6710·7–10034·1)	2417·1 (233·6–5100·4)	10656·8 (7936·4–14028·2)	29·3 (3·1–62·0)	964·5 (640·6–1379·5)	283·3 (30·9–641·8)	1247·8 (772·4–1937·0)
San Marino	5826·7 (4743·4–7088·0)	1881·2 (104·9–3959·2)	7707·8 (5564·7–10262·4)	32·3 (1·7–67·7)	684·7 (458·1–969·5)	219·7 (11·0–478·6)	904·4 (563·9–1332·0)
Spain	5078·6 (4156·6–6136·7)	1579·3 (197·5–3359·3)	6657·9 (4863·8–8969·1)	31·1 (3·7–64·6)	597·5 (389·6–847·7)	186·0 (22·7–400·9)	783·5 (465·6–1151·2)
Sweden	5281·3 (4613·2–6007·3)	1228·5 (70·3–2641·4)	6509·8 (5170·6–8072·9)	23·3 (1·4–50·9)	624·2 (421·6–872·1)	145·0 (7·0–334·8)	769·2 (488·9–1126·6)
Switzerland	7138·1 (5851·4–8634·1)	1080·8 (–636·3–3282·0)	8218·9 (5947·7–11211·2)	15·1 (–9·2–44·9)	839·0 (560·9–1192·5)	127·7 (–74·4–414·0)	966·7 (579·3–1475·1)
UK	4803·4 (4160·3–5484·5)	1380·7 (1145·9–1664·1)	6184·1 (5391·0–7102·4)	28·8 (24·6–33·0)	564·7 (385·9–789·9)	162·2 (106·9–234·0)	726·9 (491·8–1015·4)
**Latin America and Caribbean**	**5705·9 (4865·4–6732·9)**	**1804·1 (1425·8–2225·1)**	**7510·0 (6397·9–8786·6)**	**31·7 (25·8–37·7)**	**677·7 (457·5–958·2)**	**214·1 (142·6–307·3)**	**891·8 (603·3–1250·3)**
**Andean Latin America**	**5337·8 (4287·4–6703·7)**	**2774·6 (1648·6–4159·3)**	**8112·4 (6338·1–10270·2)**	**52·2 (30·7–75·6)**	**640·7 (418·5–931·7)**	**332·7 (178·2–536·9)**	**973·4 (631·4–1448·6)**
Bolivia	5378·1 (4305·0–6715·8)	2722·5 (925·6–5011·9)	8100·5 (5851·2–10966·1)	50·8 (17·8–91·4)	644·5 (420·8–938·5)	326·1 (108·1–654·7)	970·5 (602·1–1471·0)
Ecuador	5135·2 (4109·2–6446·0)	2475·0 (886·4–4517·9)	7610·2 (5515·1–10223·2)	48·3 (18·7–86·6)	616·8 (402·3–893·2)	296·6 (105·2–567·0)	913·4 (564·1–1356·1)
Peru	5430·3 (4350·7–6812·7)	2949·6 (1179·5–5057·5)	8379·9 (6122·0–11173·5)	54·5 (22·3–92·8)	652·0 (422·4–954·2)	353·9 (133·0–672·6)	1005·8 (632·4–1508·9)
**Caribbean**	**4517·0 (3646·4–5539·4)**	**1036·0 (441·6–1722·2)**	**5553·0 (4445·6–7035·9)**	**23·0 (10·2–37·7)**	**537·1 (352·0–774·8)**	**123·3 (50·2–232·6)**	**660·4 (424·8–957·1)**
Antigua and Barbuda	4738·5 (3823·7–5849·7)	1018·5 (–244·0–2542·5)	5757·1 (4137·5–7786·2)	21·5 (–6·0–52·3)	564·1 (371·4–816·2)	121·9 (–27·2–319·8)	686·0 (405·1–1057·7)
Bahamas	4706·6 (3791·1–5840·8)	1789·3 (393·2–3564·2)	6495·9 (4720·3–8772·3)	38·1 (9·3–73·4)	561·4 (364·0–807·8)	212·6 (49·3–429·4)	774·1 (487·2–1173·9)
Barbados	4789·6 (3855·2–5883·5)	932·0 (–313·0–2343·9)	5721·6 (4167·0–7460·3)	19·6 (–6·5–52·7)	566·7 (372·8–819·8)	110·6 (–36·7–298·5)	677·3 (411·0–1055·4)
Belize	4288·9 (3452·5–5406·2)	1662·6 (344·7–3261·8)	5951·5 (4218·9–8114·8)	38·8 (8·5–73·6)	515·0 (336·9–749·8)	198·7 (40·7–406·6)	713·8 (436·7–1077·9)
Bermuda	4809·2 (3935·0–5915·0)	1023·7 (–241·5–2586·2)	5832·9 (4222·8–7918·2)	21·3 (–5·2–52·7)	568·9 (372·1–813·5)	120·9 (–27·3–323·2)	689·9 (413·8–1048·0)
Cuba	4693·5 (3799·0–5755·7)	911·6 (–336·5–2544·9)	5605·0 (4060·8–7732·3)	19·4 (–7·3–51·6)	554·5 (370·2–793·1)	108·2 (–37·0–314·1)	662·7 (406·0–1022·0)
Dominica	4578·7 (3723·6–5619·5)	939·2 (–284·4–2503·3)	5517·8 (3995·9–7537·4)	20·5 (–6·6–52·9)	543·5 (359·1–788·4)	110·7 (–35·9–303·1)	654·2 (398·6–998·9)
Dominican Republic	4460·1 (3590·0–5553·4)	1438·1 (165·9–2973·5)	5898·1 (4210·8–7945·8)	32·3 (3·5–66·7)	534·6 (347·8–778·8)	173·0 (19·4–381·3)	707·6 (418·6–1096·1)
Grenada	4603·1 (3706·9–5696·3)	881·1 (–397·7–2315·8)	5484·2 (3866·8–7444·0)	19·2 (–8·2–49·5)	548·4 (357·8–801·2)	105·3 (–48·0–294·6)	653·7 (393·8–996·8)
Guyana	4431·4 (3552·4–5504·4)	1295·0 (89·0–2859·2)	5726·4 (4108·2–7901·9)	29·3 (2·2–63·2)	525·9 (346·5–766·4)	153·4 (10·7–355·5)	679·3 (409·7–1040·7)
Haiti	4323·6 (3451·6–5409·5)	694·2 (–429·8–2073·7)	5017·8 (3556·9–6834·1)	16·2 (–10·1–47·8)	514·5 (336·3–750·9)	82·4 (–53·0–250·1)	596·9 (359·6–906·7)
Jamaica	4477·2 (3604·6–5553·8)	914·3 (–235·3–2262·0)	5391·5 (3901·1–7285·7)	20·4 (–4·9–49·4)	535·7 (348·7–776·6)	109·6 (–29·2–292·9)	645·3 (385·6–1002·7)

Puerto Rico	4896·5 (3968·8–5989·9)	1347·6 (–34·2–3042·3)	6244·1 (4517·3–8369·7)	27·6 (–0·8–60·5)	576·7 (378·3–832·4)	157·7 (–4·2–357·7)	734·4 (460·3–1110·0)
Saint Kitts and Nevis	4774·4 (3839·2–5913·9)	965·5 (–311·9–2438·9)	5739·9 (4146·7–7765·2)	20·2 (–5·9–49·5)	567·5 (369·9–820·0)	114·7 (–34·2–301·7)	682·2 (418·9–1057·0)
Saint Lucia	4731·7 (3851·8–5871·0)	956·2 (–274·2–2480·5)	5687·9 (4147·3–7619·5)	20·3 (–5·9–52·6)	560·3 (371·2–808·1)	113·0 (–32·2–306·5)	673·3 (420·8–1029·6)
Saint Vincent and the Grenadines	4521·9 (3675·4–5577·2)	840·5 (–341·5–2342·5)	5362·5 (3857·1–7314·7)	18·6 (–7·6–51·0)	537·4 (351·4–767·5)	99·9 (–39·1–292·4)	637·3 (384·7–981·9)
Suriname	4398·0 (3573·8–5427·3)	1210·4 (–54·7–2636·0)	5608·4 (4048·9–7562·2)	27·5 (–1·2–58·9)	521·5 (340·1–756·3)	143·0 (–6·1–351·5)	664·4 (401·9–1023·0)
Trinidad and Tobago	4376·6 (3520·6–5381·5)	951·4 (–241·4–2465·4)	5328·0 (3829·5–7400·5)	21·6 (–5·7–54·5)	517·5 (338·4–757·4)	112·5 (–27·1–308·3)	630·0 (378·0–966·7)
Virgin Islands	4741·7 (3843·8–5828·2)	625·7 (–579·8–2145·6)	5367·4 (3853·8–7373·4)	13·2 (–12·4–44·6)	557·2 (371·6–801·0)	73·3 (–68·5–263·4)	630·5 (391·5–970·6)
**Central Latin America**	**4037·2 (3400·5–4805·2)**	**1512·4 (1091·1–1966·1)**	**5549·6 (4579·0–6623·4)**	**37·5 (28·3–47·0)**	**482·8 (323·0–685·3)**	**180·8 (114·6–272·5)**	**663·6 (444·0–947·8)**
Colombia	4865·0 (3889·5–6011·4)	1718·6 (308·6–3349·0)	6583·5 (4801·4–8673·7)	35·4 (6·6–68·8)	582·8 (378·3–833·7)	206·2 (30·8–422·5)	789·0 (488·4–1202·2)
Costa Rica	4379·9 (3554·3–5457·7)	1554·5 (179·6–3123·6)	5934·3 (4256·6–8130·7)	35·6 (4·4–70·9)	522·9 (340·9–757·1)	184·8 (21·8–393·5)	707·7 (431·1–1078·1)
El Salvador	4357·9 (3509·6–5462·0)	1111·2 (–120·5–2614·0)	5469·1 (3829·8–7397·4)	25·6 (–2·6–57·7)	521·4 (343·0–753·1)	133·2 (–13·6–323·4)	654·5 (394·9–989·2)
Guatemala	4073·8 (3277·1–5166·1)	1608·4 (370·1–3214·6)	5682·2 (4004·0–7888·1)	39·4 (9·2–75·4)	487·1 (314·7–708·4)	192·0 (41·9–404·1)	679·1 (421·7–1048·4)
Honduras	4049·2 (3235·1–5061·8)	1879·6 (489·4–3531·9)	5928·8 (4184·9–8082·1)	46·4 (13·3–85·6)	487·3 (314·1–706·6)	227·2 (61·9–468·5)	714·5 (411·3–1116·3)
Mexico	3662·0 (3136·2–4233·4)	1455·6 (1135·9–1816·7)	5117·7 (4365·0–5954·1)	39·8 (32·8–48·0)	437·3 (295·4–616·9)	173·5 (113·6–250·4)	610·8 (414·7–858·4)
Nicaragua	4231·7 (3435·3–5314·3)	1084·5 (–131·6–2659·8)	5316·2 (3651·7–7478·0)	25·5 (–3·5–59·5)	508·4 (331·7–739·0)	130·4 (–13·7–330·6)	638·7 (382·3–1006·7)
Panama	3883·8 (3138·9–4791·2)	2179·1 (818·9–3955·0)	6062·9 (4398·7–8248·8)	56·2 (21·6–95·7)	465·1 (304·0–671·1)	259·9 (92·0–498·5)	725·1 (441·9–1086·8)
Venezuela	4142·1 (3334·7–5116·3)	1320·4 (138·5–2875·8)	5462·5 (3930·1–7312·8)	32·0 (3·3–66·0)	495·3 (324·9–720·4)	158·2 (15·7–354·7)	653·5 (404·7–995·6)
**Tropical Latin America**	**7925·4 (6842·8–9164·5)**	**2017·2 (1373·3–2702·5)**	**9942·6 (8533·9–11581·0)**	**25·5 (17·9–33·7)**	**934·8 (631·3–1310·4)**	**237·7 (141·4–366·1)**	**1172·5 (796·1–1634·3)**
Brazil	7964·9 (6892·1–9191·2)	2016·5 (1362·4–2704·4)	9981·3 (8585·7–11601·4)	25·4 (17·7–33·9)	939·2 (634·0–1316·0)	237·5 (141·1–363·1)	1176·6 (802·5–1642·2)
Paraguay	6741·0 (5411·1–8347·3)	2039·7 (105·5–4211·3)	8780·7 (6441·2–11785·6)	30·3 (1·6–62·1)	805·1 (523·9–1168·8)	243·8 (13·8–548·3)	1048·8 (667·1–1629·0)
**North Africa and Middle East**	**5148·9 (4210·4–6289·4)**	**1664·8 (1178·0–2251·6)**	**6813·6 (5557·9–8391·8)**	**32·4 (24·9–41·1)**	**616·6 (408·5–882·7)**	**198·8 (124·5–303·9)**	**815·4 (542·7–1166·8)**
Afghanistan	4565·5 (3556·9–5735·6)	1314·0 (2·9–2974·1)	5879·5 (4118·9–8276·5)	28·8 (0·1–61·4)	548·5 (348·1–814·8)	156·7 (0·2–366·6)	705·3 (431·9–1092·7)
Algeria	4890·2 (3935·6–6053·1)	1189·3 (–179·5–2786·5)	6079·5 (4380·7–8152·0)	24·4 (–3·6–58·3)	586·4 (382·2–853·4)	142·8 (–20·2–356·5)	729·2 (431·2–1092·8)
Bahrain	5110·6 (4048·3–6335·3)	1369·7 (–113·4–3113·1)	6480·3 (4465·5–8750·2)	26·8 (–2·2–58·3)	611·5 (397·6–881·5)	162·8 (–15·3–392·3)	774·3 (466·5–1176·9)
Egypt	4396·4 (3516·1–5510·3)	1632·1 (271·1–3445·4)	6028·6 (4264·0–8367·3)	37·2 (7·1–73·7)	530·2 (345·4–778·6)	196·6 (36·2–419·2)	726·7 (441·3–1120·4)
Iran	7147·5 (6142·3–8247·9)	2799·5 (2144·1–3523·8)	9947·0 (8535·3–11618·3)	39·2 (31·4–47·6)	849·1 (575·1–1189·9)	332·2 (214·1–494·8)	1181·3 (800·6–1672·0)
Iraq	5158·2 (4087·3–6444·5)	1749·6 (260·0–3621·2)	6907·9 (4931·8–9414·2)	34·0 (4·7–69·3)	619·7 (396·2–910·3)	209·1 (32·2–430·8)	828·8 (505·4–1259·5)
Jordan	5140·6 (4127·7–6416·7)	1136·9 (–338·6–2846·9)	6277·5 (4437·7–8777·6)	22·0 (–6·4–53·4)	621·5 (400·3–902·0)	137·5 (–33·8–366·5)	759·0 (456·2–1186·4)
Kuwait	5200·4 (4088·3–6552·4)	1631·7 (158·1–3458·2)	6832·1 (4851·8–9355·9)	31·4 (2·7–64·2)	622·4 (406·8–910·1)	195·6 (17·9–454·6)	817·9 (493·4–1227·3)
Lebanon	6284·8 (5061·4–7740·6)	1624·8 (–35·1–3723·1)	7909·6 (5697·3–10592·5)	25·9 (–0·7–57·8)	747·1 (482·8–1081·5)	192·4 (–4·6–446·7)	939·5 (580·3–1410·4)
Libya	5604·9 (4498·6–6910·8)	1508·0 (–9·5–3422·8)	7112·8 (5158·6–9674·7)	26·9 (–0·2–58·1)	670·2 (432·1–971·0)	180·5 (–2·7–425·7)	850·6 (502·7–1288·6)
Morocco	5099·8 (4106·5–6309·4)	1816·6 (262·8–3534·0)	6916·3 (4893·8–9379·5)	35·6 (5·4–67·4)	609·4 (392·7–882·9)	216·5 (28·8–453·6)	825·9 (501·9–1247·0)

Oman	4909·7 (3823·5–6195·0)	963·4 (–399·7–2490·5)	5873·1 (4124·7–8086·0)	19·7 (–7·3–48·6)	593·9 (378·6–868·9)	116·5 (–43·1–312·0)	710·4 (424·6–1114·0)
Palestine	5279·3 (4177·5–6647·3)	1881·2 (272·9–3784·7)	7160·5 (4992·2–9801·9)	35·6 (5·3–73·3)	636·1 (404·0–921·3)	227·0 (29·9–512·6)	863·0 (518·7–1332·2)
Qatar	4769·8 (3719·9–6064·0)	1486·3 (111·4–3124·6)	6256·2 (4279·1–8634·1)	31·1 (2·3–65·2)	575·9 (366·9–832·6)	178·9 (15·5–393·2)	754·9 (442·2–1139·6)
Saudi Arabia	5146·2 (4076·4–6497·1)	1771·7 (239·7–3726·2)	6917·9 (4849·0–9637·7)	34·3 (4·9–68·7)	616·7 (396·6–912·8)	212·5 (27·8–477·1)	829·2 (502·9–1300·2)
Sudan	4882·9 (3879·5–6189·9)	1410·8 (62·6–3185·1)	6293·8 (4492·2–8800·9)	28·9 (1·6–64·3)	589·3 (375·9–863·7)	168·8 (7·9–394·5)	758·1 (455·3–1173·3)
Syria	5723·9 (4618·5–7103·1)	1193·6 (–275·0–3065·9)	6917·4 (4887·1–9439·0)	20·8 (–4·7–52·1)	682·4 (439·0–983·7)	141·9 (–33·6–379·6)	824·3 (501·4–1296·3)
Tunisia	5389·4 (4363·9–6637·9)	1607·3 (14·1–3499·8)	6996·7 (4955·9–9490·8)	29·8 (0·3–64·7)	642·0 (417·8–930·2)	190·8 (1·8–432·9)	832·8 (511·7–1278·1)
Turkey	4565·7 (3712·0–5628·0)	1598·1 (254·4–3335·1)	6163·8 (4363·8–8309·0)	35·1 (5·5–71·2)	545·4 (358·5–780·6)	189·6 (29·6–406·9)	735·0 (461·4–1084·2)
United Arab Emirates	4645·4 (3621·5–5905·3)	1117·8 (–124·1–2746·7)	5763·2 (4025·7–8091·4)	24·0 (–2·4–56·3)	557·3 (358·2–814·8)	134·0 (–15·6–336·5)	691·4 (406·4–1070·0)
Yemen	4771·1 (3741·1–6028·6)	667·7 (–512·6–2097·7)	5438·8 (3881·6–7392·5)	14·1 (–10·8–43·8)	570·5 (369·2–830·6)	79·8 (–65·8–262·7)	650·3 (388·0–985·4)
**South Asia**	**3019·7 (2590·4–3531·6)**	**1058·3 (813·0–1318·7)**	**4077·9 (3459·3–4786·7)**	**35·1 (28·2–42·0)**	**358·2 (243·5–503·0)**	**125·3 (81·0–181·3)**	**483·5 (328·8–679·5)**
Bangladesh	3184·4 (2552·4–4012·8)	1154·1 (137·8–2406·7)	4338·5 (3030·2–5966·6)	36·2 (4·6–75·3)	379·5 (247·9–551·5)	137·2 (14·9–314·1)	516·7 (311·3–796·6)
Bhutan	3280·7 (2641·8–4124·8)	516·7 (–383·3–1531·7)	3797·4 (2624·2–5165·6)	15·8 (–11·4–46·8)	391·9 (255·6–577·1)	61·5 (–40·5–193·7)	453·4 (267·8–708·5)
India	3013·4 (2594·0–3492·9)	1049·3 (816·8–1309·5)	4062·7 (3451·5–4734·1)	34·8 (28·5–41·5)	356·9 (242·8–500·6)	124·1 (81·5–180·0)	480·9 (327·5–675·3)
Nepal	3121·5 (2507·2–3938·6)	1141·5 (159·4–2232·4)	4263·0 (3007·2–5758·7)	36·6 (4·8–71·5)	371·0 (242·1–542·9)	134·9 (18·3–276·6)	505·9 (314·0–768·5)
Pakistan	2927·8 (2505·0–3426·4)	1036·4 (521·2–1687·2)	3964·2 (3182·4–4845·8)	35·4 (18·0–55·7)	349·5 (236·8–492·9)	123·4 (54·1–218·7)	473·0 (314·1–684·6)
**Southeast Asia, east Asia, and Oceania**	**3367·2 (2903·3–3891·5)**	**466·0 (307·2–632·0)**	**3833·2 (3281·8–4478·2)**	**13·8 (9·3–18·3)**	**403·4 (271·6–571·6)**	**55·8 (31·8–85·0)**	**459·2 (307·8–651·2)**
**East Asia**	**3302·6 (2862·8–3776·4)**	**361·9 (172·0–555·3)**	**3664·6 (3158·1–4238·3)**	**11·0 (5·1–16·9)**	**394·8 (266·3–558·0)**	**43·3 (17·8–74·5)**	**438·0 (298·0–618·8)**
China	3284·4 (2851·3–3756·3)	366·9 (175·0–567·6)	3651·2 (3148·5–4220·1)	11·2 (5·3–17·3)	392·6 (264·9–554·6)	43·8 (18·4–74·8)	436·4 (296·0–617·4)
North Korea	3745·9 (3042·5–4543·5)	259·8 (–702·8–1403·6)	4005·7 (2881·8–5489·9)	6·9 (–17·9–36·7)	450·2 (295·2–649·2)	30·7 (–89·2–178·5)	480·9 (293·9–771·8)
Taiwan (province of China)	3899·4 (3184·6–4748·5)	181·2 (–798·9–1221·7)	4080·6 (2931·6–5448·7)	4·7 (–19·6–32·7)	463·8 (305·0–664·1)	21·9 (–99·7–154·6)	485·6 (285·5–745·9)
**Oceania**	**3542·6 (2827·0–4453·6)**	**42·7 (–578·6–838·4)**	**3585·3 (2658·1–4801·5)**	**1·2 (–16·7–22·9)**	**427·6 (272·6–624·4)**	**5·1 (–73·8–98·6)**	**432·7 (267·6–654·0)**
American Samoa	3816·3 (3101·4–4738·1)	332·8 (–604·4–1398·3)	4149·1 (3000·2–5679·1)	8·7 (–15·4–37·0)	456·2 (299·1–658·5)	39·9 (–74·1–172·3)	496·0 (302·9–759·6)
Cook Islands	4072·5 (3325·8–4949·2)	355·2 (–606·4–1555·6)	4427·8 (3215·6–5946·8)	8·8 (–15·1–37·1)	482·0 (316·7–699·5)	42·0 (–75·2–190·9)	524·0 (324·9–806·6)
Federated States of Micronesia	3748·7 (3031·4–4608·4)	116·9 (–708·5–1276·4)	3865·6 (2714·7–5273·7)	3·1 (–18·9–34·1)	451·7 (290·1–656·0)	13·8 (–88·6–151·3)	465·6 (284·8–716·8)
Fiji	3725·1 (3008·0–4562·6)	283·2 (–617·0–1359·5)	4008·3 (2873·7–5455·0)	7·7 (–16·7–36·5)	446·7 (290·0–641·4)	34·0 (–78·5–175·0)	480·7 (298·5–748·0)
Guam	3798·5 (3099·8–4633·8)	878·6 (–245·5–2207·9)	4677·2 (3258·7–6331·9)	23·2 (–6·5–56·1)	455·8 (299·2–660·3)	105·4 (–29·6–281·3)	561·2 (341·9–875·0)
Kiribati	3622·3 (2891·2–4498·7)	169·8 (–655·2–1171·4)	3792·2 (2697·7–5154·3)	4·7 (–18·0–30·9)	436·2 (278·6–629·6)	20·7 (–83·1–150·2)	456·9 (275·8–716·7)
Marshall Islands	3660·1 (2927·8–4583·0)	125·1 (–768·1–1189·2)	3785·2 (2681·4–5226·5)	3·4 (–20·7–30·6)	440·4 (284·2–637·6)	15·2 (–92·4–148·9)	455·6 (265·7–714·3)
Nauru	3565·9 (2855·1–4454·2)	347·9 (–527·2–1451·2)	3913·7 (2712·9–5531·4)	9·7 (–14·5–39·2)	430·5 (273·4–628·3)	41·7 (–65·6–181·8)	472·2 (289·1–735·9)
Niue	3956·6 (3224·6–4823·7)	338·4 (–625·6–1466·6)	4295·0 (3100·8–5893·3)	8·5 (–15·2–36·3)	469·1 (310·3–676·4)	39·7 (–78·6–177·3)	508·7 (322·6–779·0)

Northern Mariana Islands	4096·3 (3310·3–5014·0)	823·8 (–215·4–2233·3)	4920·1 (3581·0–6736·4)	20·1 (–5·9–53·9)	489·0 (322·0–711·2)	98·4 (–25·2–276·5)	587·4 (363·8–905·2)
Palau	3957·7 (3181·4–4839·9)	299·9 (–627·0–1429·5)	4257·5 (3079·9–5789·1)	7·6 (–15·7–35·6)	468·9 (310·9–677·8)	35·3 (–76·2–176·4)	504·3 (317·5–761·2)
Papua New Guinea	3518·8 (2782·9–4427·0)	–6·7 (–810·7–949·6)	3512·1 (2500·9–4877·2)	–0·3 (–23·1–27·2)	425·1 (269·0–619·8)	–0·9 (–104·2–119·0)	424·3 (257·8–652·5)
Samoa	3556·1 (2891·2–4408·8)	78·5 (–744·3–1132·5)	3634·6 (2572·1–5063·8)	2·2 (–20·9–30·7)	428·8 (279·9–620·2)	9·0 (–96·6–132·5)	437·8 (262·4–665·4)
Solomon Islands	3459·0 (2747·7–4330·3)	36·9 (–758·0–875·4)	3495·9 (2489·4–4788·0)	1·1 (–22·1–26·0)	418·4 (271·6–612·2)	4·6 (–91·7–112·9)	423·0 (261·7–658·3)
Tokelau	3751·4 (3061·2–4623·9)	382·9 (–549·9–1420·8)	4134·2 (2932·9–5482·1)	10·3 (–14·6–38·8)	448·7 (297·5–648·9)	45·8 (–65·9–179·8)	494·6 (302·4–753·0)
Tonga	3610·1 (2904·8–4440·8)	335·7 (–557·4–1409·4)	3945·8 (2784·1–5327·1)	9·3 (–15·4–40·0)	435·3 (281·7–637·0)	41·3 (–68·0–179·9)	476·5 (289·1–745·9)
Tuvalu	3676·9 (2960·9–4526·2)	338·7 (–530·6–1443·0)	4015·6 (2863·2–5492·2)	9·2 (–14·9–37·8)	441·4 (287·7–638·1)	41·0 (–69·8–188·2)	482·3 (291·4–750·8)
Vanuatu	3459·9 (2759·0–4304·4)	35·4 (–722·9–951·8)	3495·4 (2496·3–4789·9)	1·1 (–21·1–28·0)	418·5 (268·3–605·3)	4·6 (–88·9–117·5)	423·1 (257·2–649·3)
Southeast Asia	3501·1 (2966·5–4123·2)	695·7 (454·0–970·2)	4196·7 (3535·9–4982·4)	19·9 (13·5–26·7)	421·1 (282·5–597·3)	83·6 (46·9–129·2)	504·8 (333·9–720·4)
Cambodia	3781·5 (3064·0–4689·8)	453·1 (–461·3–1632·8)	4234·6 (3003·6–5782·6)	12·0 (–12·0–42·2)	454·7 (296·0–657·9)	55·0 (–55·3–204·0)	509·7 (319·8–796·4)
Indonesia	3531·8 (3037·7–4088·6)	717·2 (412·0–1083·8)	4249·0 (3605·3–5017·2)	20·3 (12·2–29·0)	426·3 (285·9–596·7)	86·5 (42·5–141·1)	512·8 (343·9–723·5)
Laos	4328·1 (3558·8–5299·4)	613·8 (–450·0–1889·7)	4941·8 (3645·6–6561·9)	14·2 (–10·5–44·3)	523·7 (343·1–764·5)	73·5 (–56·5–230·5)	597·2 (378·1–897·9)
Malaysia	4461·5 (3557·7–5569·4)	618·3 (–461·3–2008·8)	5079·8 (3634·5–7139·4)	13·9 (–11·0–43·9)	535·9 (348·3–781·1)	74·3 (–53·4–257·3)	610·2 (362·7–930·1)
Maldives	3481·3 (2759·4–4449·8)	841·6 (–105·8–2094·3)	4322·9 (3089·2–6014·3)	24·2 (–3·0–57·1)	420·7 (270·9–613·3)	101·4 (–14·2–256·0)	522·1 (319·5–803·9)
Mauritius	3959·2 (3233·1–4879·5)	596·5 (–407·3–1813·2)	4555·7 (3314·2–6244·1)	15·1 (–10·3–45·0)	467·5 (306·8–673·4)	70·2 (–43·8–219·8)	537·7 (330·6–839·5)
Myanmar	3713·8 (2977·2–4527·2)	997·4 (–58·3–2240·0)	4711·1 (3409·6–6438·6)	26·9 (–1·5–60·0)	445·6 (288·0–639·1)	120·0 (–7·8–285·3)	565·6 (331·9–870·0)
Philippines	3778·4 (3240·2–4367·2)	1270·9 (1010·7–1577·9)	5049·3 (4324·5–5878·0)	33·6 (27·8–40·0)	455·4 (307·3–641·3)	153·0 (100·2–224·4)	608·4 (411·3–857·3)
Seychelles	3683·2 (2971·5–4544·8)	653·9 (–301·1–1831·3)	4337·2 (3103·4–5883·8)	17·8 (–7·7–47·9)	440·6 (289·5–635·6)	78·3 (–33·1–230·1)	518·9 (310·0–797·4)
Sri Lanka	3884·6 (3192·1–4743·0)	744·2 (–275·3–2008·0)	4628·8 (3304·5–6287·6)	19·1 (–7·1–51·2)	462·4 (302·7–669·9)	88·2 (–34·3–252·2)	550·6 (329·9–847·6)
Thailand	3761·5 (3035·5–4647·1)	300·2 (–644·3–1457·0)	4061·7 (2844·9–5549·6)	7·9 (–17·0–37·5)	446·6 (293·6–644·5)	35·6 (–80·2–170·8)	482·2 (289·8–723·5)
Timor–Leste	3246·8 (2598·9–4034·8)	502·7 (–370·2–1496·6)	3749·4 (2675·1–5001·2)	15·6 (–11·8–44·9)	392·6 (253·4–575·1)	61·2 (–44·1–194·2)	453·8 (272·3–718·9)
Vietnam	2284·5 (1835·6–2826·1)	158·4 (–386·8–830·3)	2442·8 (1721·6–3331·2)	6·9 (–17·2–34·5)	275·3 (181·4–396·9)	19·2 (–46·3–102·6)	294·5 (178·3–456·8)
**Sub–Saharan Africa**	**3001·9 (2465·1–3671·3)**	**644·0 (479·0–829·9)**	**3645·9 (2985·7–4475·5)**	**21·5 (17·1–25·7)**	**361·8 (237·5–519·5)**	**77·5 (50·0–116·0)**	**439·3 (289·0–633·7)**
**Central sub–Saharan Africa**	**3187·4 (2538·4–4034·5)**	**622·8 (8·0–1331·7)**	**3810·1 (2857·6–5029·4)**	**19·6 (0·3–41·5)**	**382·6 (246·2–554·2)**	**74·7 (0·7–169·5)**	**457·2 (286·6–683·3)**
Angola	3125·0 (2465·2–3980·2)	619·9 (–170·0–1646·0)	3744·9 (2651·6–5113·1)	19·9 (–5·6–52·1)	376·8 (239·1–547·2)	74·5 (–19·4–203·4)	451·3 (268·2–680·3)
Central African Republic	3471·0 (2751·9–4401·7)	533·8 (–361·6–1618·5)	4004·8 (2882·3–5571·4)	15·5 (–11·0–44·0)	414·7 (265·7–603·5)	64·1 (–42·0–197·4)	478·8 (284·6–726·9)
Congo	3370·7 (2687·9–4216·7)	722·8 (–172·9–1819·8)	4093·4 (2915·8–5669·1)	21·4 (–4·9–52·6)	403·9 (259·0–591·7)	86·3 (–20·8–236·9)	490·2 (292·7–753·8)
Democratic Republic of the Congo	3171·5 (2512·9–4028·9)	623·4 (–221·8–1599·5)	3794·9 (2713·1–5117·0)	19·7 (–7·0–50·7)	380·2 (243·2–550·4)	74·7 (–24·7–202·7)	454·9 (277·8–711·0)
Equatorial Guinea	3369·6 (2644·6–4354·7)	641·5 (–261·7–1745·8)	4011·1 (2788·1–5600·2)	19·0 (–7·9–48·4)	405·8 (258·0–589·5)	77·1 (–33·7–215·3)	482·8 (287·6–738·1)
Gabon	3496·1 (2775·4–4411·5)	598·2 (–334·7–1690·0)	4094·3 (2894·7–5645·9)	17·1 (–9·4–47·0)	418·2 (267·6–602·2)	71·5 (–38·4–199·9)	489·7 (284·1–745·2)
**Eastern sub–Saharan Africa**	**3193·7 (2609·9–3956·1)**	**598·9 (365·7–870·5)**	**3792·6 (3084·1–4701·7)**	**18·8 (11·9–26·3)**	**385·4 (252·2–556·0)**	**72·2 (39·1–119·7)**	**457·6 (301·4–661·9)**

Burundi	3076·4 (2430·2–3915·4)	313·2 (–457·0–1242·5)	3389·6 (2432·1–4738·6)	10·2 (–15·3–38·8)	370·3 (236·8–540·1)	37·4 (–60·4–150·9)	407·7 (248·3–635·6)
Comoros	3450·2 (2735·1–4354·5)	693·6 (–217·1–1853·3)	4143·8 (2879·3–5706·9)	20·1 (–6·2–52·5)	416·4 (265·7–606·0)	83·6 (–25·0–234·0)	500·0 (299·0–778·7)
Djibouti	3194·8 (2513·0–4046·4)	552·1 (–311·6–1557·0)	3746·9 (2617·6–5140·6)	17·3 (–10·1–47·9)	386·5 (248·2–561·1)	66·6 (–37·6–199·9)	453·1 (271·5–725·4)
Eritrea	3282·8 (2589·3–4171·5)	358·0 (–440·3–1312·1)	3640·8 (2541·4–5130·7)	10·9 (–13·4–39·6)	395·7 (253·6–578·3)	43·1 (–49·9–168·7)	438·9 (265·7–687·0)
Ethiopia	3154·2 (2671·6–3718·0)	612·4 (199·5–1074·0)	3766·6 (3037·8–4608·3)	19·4 (6·2–32·9)	381·7 (255·6–545·1)	74·1 (22·3–142·4)	455·8 (305·2–649·1)
Kenya	3179·6 (2712·1–3741·8)	972·6 (754·5–1208·9)	4152·2 (3531·3–4862·3)	30·6 (25·0–36·5)	384·2 (258·3–544·2)	117·4 (75·0–170·4)	501·6 (335·8–709·5)
Madagascar	3380·9 (2662·6–4333·4)	611·7 (–224·7–1656·4)	3992·7 (2792·1–5507·8)	18·1 (–7·1–46·5)	409·1 (260·0–602·0)	73·8 (–27·5–209·6)	482·9 (293·9–755·3)
Malawi	3255·8 (2573·6–4162·7)	601·0 (–229·6–1660·8)	3856·8 (2690·3–5260·1)	18·5 (–7·1–49·8)	393·7 (251·2–574·4)	71·9 (–26·3–201·6)	465·6 (275·2–711·9)
Mozambique	3191·5 (2521·1–4072·8)	485·8 (–312·6–1492·0)	3677·3 (2530·1–5054·7)	15·2 (–10·1–46·8)	379·9 (242·7–551·9)	58·3 (–36·2–185·2)	438·2 (264·6–676·6)
Rwanda	3376·7 (2685·5–4306·0)	563·7 (–335·7–1740·4)	3940·5 (2731·1–5489·1)	16·6 (–10·0–47·5)	406·5 (260·9–593·3)	67·3 (–42·4–208·8)	473·8 (283·1–729·3)
Somalia	2819·8 (2218·2–3577·7)	456·6 (–258·3–1293·5)	3276·4 (2224·2–4545·3)	16·2 (–10·5–47·1)	340·5 (215·6–505·3)	55·2 (–34·3–167·1)	395·7 (234·6–614·4)
South Sudan	3516·9 (2777·7–4440·2)	502·2 (–382·0–1566·1)	4019·1 (2833·3–5532·9)	14·3 (–11·3–42·3)	422·2 (271·0–618·5)	59·7 (–46·9–194·5)	481·9 (289·7–735·1)
Uganda	3152·0 (2455·1–4050·8)	667·4 (–155·6–1743·4)	3819·5 (2627·3–5273·1)	21·2 (–4·9–51·7)	381·3 (240·7–565·7)	80·3 (–19·8–221·4)	461·6 (285·5–733·8)
Tanzania	3217·5 (2560·7–4108·7)	503·9 (–288·5–1517·5)	3721·5 (2588·1–5179·7)	15·6 (–8·9–45·4)	388·3 (248·5–564·5)	61·1 (–36·1–188·8)	449·3 (272·0–710·0)
Zambia	3293·9 (2591·9–4201·4)	331·2 (–463·5–1322·2)	3625·1 (2608·8–5124·2)	10·0 (–14·2–38·2)	396·5 (253·6–583·0)	39·2 (–53·5–161·6)	435·7 (267·0–672·0)
**Southern sub–Saharan Africa**	**3643·0 (3074·7–4332·9)**	**1272·8 (868·5–1755·5)**	**4915·9 (4094·3–5929·7)**	**35·0 (24·5–46·4)**	**432·0 (289·4–609·3)**	**150·6 (93·5–237·7)**	**582·6 (395·2–830·5)**
Botswana	3598·0 (2868·1–4543·8)	404·6 (–517·3–1463·4)	4002·6 (2839·9–5605·4)	11·3 (–14·0–39·6)	429·0 (275·7–621·5)	48·1 (–60·4–181·5)	477·1 (286·0–750·4)
Eswatini	3346·9 (2679·8–4227·0)	1058·4 (124·0–2282·1)	4405·3 (3127·7–6114·0)	31·7 (4·3–65·9)	398·7 (252·9–581·4)	126·2 (13·7–284·4)	524·9 (312·7–808·5)
Lesotho	3468·4 (2759·1–4357·0)	808·8 (–159·3–1939·6)	4277·2 (3003·6–5863·4)	23·4 (–4·1–57·1)	411·6 (263·5–595·2)	96·3 (–18·0–248·7)	507·9 (306·8–786·6)
Namibia	3374·2 (2679·0–4236·2)	971·8 (–3·0–2230·8)	4346·0 (3083·0–6012·8)	28·7 (–0·1–63·6)	405·1 (264·4–588·8)	116·2 (–0·4–274·4)	521·3 (320·8–816·1)
South Africa	3861·2 (3292·9–4474·9)	1537·7 (1038·1–2115·0)	5399·0 (4492·6–6408·8)	39·9 (26·9–53·8)	456·3 (307·9–638·6)	181·3 (110·9–287·6)	637·6 (438·2–914·3)
Zimbabwe	2947·9 (2324·1–3752·8)	560·6 (–195·9–1522·5)	3508·5 (2479·2–4829·2)	19·1 (–6·6–50·4)	354·9 (227·1–515·7)	67·5 (–22·8–194·1)	422·4 (252·6–665·1)
**Western sub–Saharan Africa**	**2673·9 (2196·1–3249·5)**	**585·8 (429·9–763·3)**	**3259·6 (2661·8–3969·7)**	**21·9 (17·0–27·5)**	**323·5 (211·0–467·3)**	**70·8 (43·0–110·4)**	**394·4 (259·4–575·9)**
Benin	2665·2 (2085·2–3392·0)	260·4 (–413·4–1003·3)	2925·6 (2042·4–4074·7)	9·8 (–15·4–37·7)	323·3 (204·1–476·8)	32·1 (–48·4–135·1)	355·4 (214·4–558·6)
Burkina Faso	2591·7 (2016·8–3291·3)	300·3 (–351·3–1189·8)	2892·0 (1944·3–4155·0)	11·4 (–14·4–44·4)	314·3 (197·2–461·7)	36·3 (–43·7–143·4)	350·6 (202·2–567·9)
Cabo Verde	2975·8 (2376·2–3726·2)	1415·3 (410·4–2791·0)	4391·1 (3154·3–6161·4)	47·7 (13·5–89·8)	358·9 (231·5–517·2)	170·0 (44·7–346·4)	528·8 (321·0–794·4)
Cameroon	2759·5 (2153·2–3525·9)	306·3 (–394·5–1138·2)	3065·8 (2135·6–4299·6)	11·1 (–13·9–42·3)	334·6 (209·6–501·0)	37·2 (–47·8–147·5)	371·9 (216·9–578·5)
Chad	2621·9 (2043·2–3356·7)	368·4 (–289·3–1228·6)	2990·3 (2077·2–4186·6)	14·0 (–11·7–48·8)	318·1 (203·1–467·2)	44·7 (–37·4–157·1)	362·8 (212·6–568·4)
Côte d'Ivoire	2783·3 (2195·1–3536·5)	340·3 (–355·2–1248·8)	3123·5 (2173·3–4356·6)	12·3 (–13·4–45·1)	337·1 (213·4–497·0)	41·6 (–41·8–158·9)	378·6 (232·8–590·2)
The Gambia	2831·1 (2228·7–3588·6)	504·4 (–280·6–1460·3)	3335·4 (2289·2–4668·9)	17·7 (–10·0–49·4)	342·4 (215·3–500·9)	60·9 (–32·3–191·5)	403·3 (228·7–641·0)
Ghana	2788·4 (2202·4–3528·5)	457·7 (–294·9–1416·6)	3246·1 (2297·0–4525·8)	16·5 (–10·8–50·1)	337·4 (216·0–491·2)	55·9 (–32·4–182·1)	393·3 (226·4–622·2)
Guinea	2768·3 (2188·1–3570·9)	463·4 (–257·8–1383·7)	3231·7 (2211·0–4524·2)	16·7 (–8·8–46·9)	335·6 (211·2–490·0)	56·0 (–30·6–171·6)	391·6 (228·6–620·9)

Guinea–Bissau	2686·5 (2083·7–3418·9)	436·5 (–235·6–1315·6)	3122·9 (2172·2–4344·0)	16·2 (–9·2–48·7)	325·3 (203·2–480·5)	53·2 (–27·2–160·4)	378·5 (220·6–599·9)
Liberia	2944·8 (2314·8–3716·4)	498·2 (–273·8–1530·9)	3443·1 (2389·1–4822·8)	16·8 (–9·8–49·0)	351·3 (224·6–513·2)	59·6 (–34·0–185·7)	410·9 (244·1–645·1)
Mali	2392·9 (1878·7–3025·3)	447·2 (–208·0–1229·4)	2840·1 (1998·5–3933·6)	18·7 (–9·0–50·4)	289·9 (183·6–426·1)	53·8 (–24·9–151·4)	343·6 (204·7–531·7)
Mauritania	2598·4 (2068·4–3271·6)	550·0 (–172·0–1348·5)	3148·3 (2222·5–4320·6)	21·2 (–6·9–52·2)	315·9 (199·6–461·0)	66·9 (–19·1–178·6)	382·9 (225·0–600·1)
Niger	2416·4 (1881·0–3076·5)	266·3 (–297·7–1007·7)	2682·6 (1874·8–3604·7)	11·2 (–12·5–41·4)	294·5 (185·2–430·9)	32·8 (–36·3–125·2)	327·4 (196·2–515·9)
Nigeria	2663·9 (2256·3–3115·9)	798·6 (607·7–1015·9)	3462·5 (2912·4–4095·4)	30·0 (24·3–36·0)	321·9 (212·9–458·4)	96·3 (62·2–140·1)	418·2 (281·5–597·2)
São Tomé and Príncipe	2912·7 (2309·7–3713·6)	540·2 (–235·2–1469·6)	3453·0 (2438·6–4811·3)	18·5 (–7·8–49·4)	353·8 (225·7–511·9)	65·4 (–27·4–188·6)	419·2 (253·1–654·0)
Senegal	2685·1 (2128·8–3379·5)	794·2 (20·3–1797·7)	3479·3 (2422·1–4859·4)	29·5 (0·9–66·4)	324·4 (205·6–479·4)	96·1 (2·6–230·0)	420·5 (249·3–663·9)
Sierra Leone	3013·6 (2376·7–3873·1)	431·8 (–348·3–1319·0)	3445·4 (2374·7–4741·8)	14·3 (–11·6–42·3)	364·5 (229·7–538·6)	52·2 (–39·7–170·3)	416·7 (246·0–649·1)
Togo	2992·8 (2383·6–3810·2)	380·1 (–385·2–1359·0)	3372·9 (2335·7–4707·6)	12·7 (–13·2–42·6)	361·8 (230·6–528·7)	45·8 (–47·7–167·9)	407·6 (239·7–628·7)

Table S9: Prevalence and DALYs of anxiety disorders in 1000s, with 95% uncertainty intervals, by location, for the year 2020

Location	Baseline prevalence	Additional prevalence	Final prevalence	% change	Baseline DALYs	Additional DALYs	Final DALYs
**Global**	**298000 (256000–348000)**	**76200 (64300–90600)**	**374000 (320000–436000)**	**25·6 (23·2–28·0)**	**35500 (23900–50100)**	**9050 (6180–12800)**	**44500 (30200–62500)**
**Central Europe, eastern Europe, and central Asia**	**13800 (11800–16100)**	**4130 (3260–5110)**	**17900 (15100–20900)**	**30·0 (24·9–35·0)**	**1620 (1090–2290)**	**486 (322–701)**	**2110 (1420–2960)**
**Central Asia**	**2030 (1640–2520)**	**677 (382–1010)**	**2700 (2100–3390)**	**33·4 (19·1–47·9)**	**244 (157–353)**	**81·1 (38·7–134)**	**325 (209–472)**
Armenia	82·9 (67·1–102)	31·6 (6·56–63·2)	114 (82·6–155)	38·2 (7·7–74·3)	9·86 (6·53–14·3)	3·76 (0·743–7·95)	13·6 (8·17–20·4)
Azerbaijan	244 (196–302)	78·2 (2·63–172)	323 (221–439)	32·0 (1·1–69·0)	29·4 (19·3–42·5)	9·4 (0·304–22·2)	38·8 (23·1–60·3)
Georgia	87·9 (71·3–107)	27·3 (2·04–59·1)	115 (82·2–156)	31·2 (2·4–65·8)	10·4 (6·85–15·1)	3·23 (0·272–7·45)	13·6 (8·22–20·5)
Kazakhstan	384 (310–474)	130 (17·1–273)	514 (369–698)	34·0 (4·1–69·3)	45·9 (29·8–66·4)	15·5 (1·89–34·1)	61·4 (37·6–95·5)
Kyrgyzstan	132 (106–163)	63·4 (17·4–121)	195 (139–273)	48·2 (14·3–90·0)	15·9 (10·1–23·2)	7·61 (1·95–15)	23·5 (14·1–35·8)
Mongolia	70·2 (56–88·1)	1·25 (–15·5–20·4)	71·5 (50·3–98·7)	1·8 (–22·3–28·8)	8·43 (5·43–11·9)	0·147 (–1·94–2·61)	8·58 (5·02–12·9)
Tajikistan	203 (162–258)	67 (7·9–140)	270 (193–373)	33·0 (3·7–67·6)	24·6 (15·8–35·7)	8·11 (0·796–18)	32·7 (19·5–51·1)
Turkmenistan	110 (87·9–137)	38·5 (3·87–80·1)	149 (108–204)	35·0 (3·4–70·8)	13·3 (8·71–19·3)	4·65 (0·421–10·4)	17·9 (10·9–28)
Uzbekistan	714 (568–896)	239 (27·2–504)	953 (663–1320)	33·5 (3·8–67·7)	86·2 (54·9–127)	28·7 (2·99–63·5)	115 (69·2–176)
**Central Europe**	**4200 (3520–5000)**	**1130 (764–1570)**	**5330 (4410–6470)**	**26·9 (18·9–34·9)**	**493 (329–709)**	**132 (76·2–205)**	**625 (420–890)**
Albania	105 (84·9–128)	38·3 (6·69–77·3)	143 (104–191)	36·5 (6·1–71·5)	12·5 (8·26–18)	4·54 (0·703–9·67)	17 (10·5–26·2)
Bosnia and Herzegovina	127 (103–158)	36·8 (2·22–83·5)	164 (117–223)	28·9 (1·8–63·6)	14·9 (9·95–21·5)	4·3 (0·256–10·2)	19·2 (12–29·2)
Bulgaria	269 (218–330)	69·1 (–2·99–158)	338 (245–456)	25·7 (–1·2–57·6)	31·5 (21–45·6)	8·07 (–0·339–20·1)	39·5 (24·6–60·8)
Croatia	166 (135–204)	36·5 (–8·85–89·5)	203 (144–278)	22·0 (–5·5–52·1)	19·5 (13–28·2)	4·26 (–1·04–10·9)	23·7 (14·6–36·2)
Czechia	382 (311–469)	101 (–3·12–242)	484 (352–667)	26·5 (–0·8–61·6)	44·6 (29·2–64)	11·8 (–0·436–28·2)	56·5 (34–85·2)
Hungary	368 (299–449)	60·2 (–33·9–173)	429 (309–586)	16·3 (–9·2–46·8)	43 (28·8–62·7)	7·05 (–3·84–21·6)	50·1 (30·4–78·2)
Montenegro	23·9 (19·4–29·2)	7·91 (0·734–16·6)	31·8 (22·3–42·4)	33·2 (3·2–69·0)	2·82 (1·86–4·05)	0·936 (0·0824–2·16)	3·76 (2·23–5·68)
North Macedonia	82 (67–100)	30 (5·53–62·1)	112 (80·2–152)	36·6 (7·1–73·8)	9·69 (6·35–14)	3·55 (0·644–7·72)	13·2 (8·14–20·5)
Poland	1380 (1190–1590)	348 (234–471)	1730 (1480–2000)	25·3 (17·5–33·8)	162 (110–227)	40·8 (24·2–63·6)	203 (139–280)
Romania	686 (556–845)	237 (18·3–504)	924 (655–1260)	34·6 (3·2–69·1)	80·7 (53·3–117)	27·9 (1·85–63·6)	109 (65·1–166)
Serbia	328 (266–400)	91·4 (1·97–219)	419 (307–567)	27·9 (0·6–64·7)	38·6 (25·3–56)	10·7 (0·224–26·3)	49·3 (30·3–74·5)
Slovakia	204 (166–249)	51·6 (–2·2–110)	256 (191–343)	25·3 (–1·1–54·1)	24 (15·8–34·7)	6·1 (–0·235–14·1)	30·1 (18·7–46·4)
Slovenia	77·6 (63·4–94·6)	20·5 (–1·21–48·7)	98·2 (70–134)	26·4 (–1·6–61·4)	9·07 (6·06–13·1)	2·4 (–0·132–6·08)	11·5 (6·93–17·9)
**Eastern Europe**	**7550 (6560–8660)**	**2320 (1750–2990)**	**9880 (8470–11500)**	**30·8 (23·9–38·5)**	**886 (601–1240)**	**272 (175–399)**	**1160 (785–1620)**
Belarus	359 (291–442)	92·1 (–10·9–224)	451 (322–621)	25·6 (–3·1–60·0)	42·2 (27·8–60·7)	10·8 (–1·15–26·5)	53 (32·6–80·8)
Estonia	49·5 (40·2–60)	8·66 (–4·81–23·4)	58·1 (41·6–78·1)	17·6 (–9·8–49·6)	5·8 (3·87–8·41)	1·02 (–0·621–3)	6·82 (4·16–10·4)

Latvia	76·1 (61·9–91·8)	15·7 (–3·44–41)	91·8 (66·3–124)	20·7 (–4·7–54·5)	8·9 (5·91–12·7)	1·85 (–0·379–5·16)	10·8 (6·57–16·5)
Lithuania	129 (104–156)	37·3 (1·81–78·6)	166 (120–225)	29·0 (1·4–62·0)	15·1 (10–21·7)	4·36 (0·194–10·2)	19·4 (12–29·2)
Republic of Moldova	154 (125–190)	58 (10·3–109)	212 (149–281)	37·8 (7·0–71·3)	18·2 (12·1–26·3)	6·87 (1·09–14·4)	25 (14·7–38·6)
Russia	5270 (4590–6040)	1680 (1390–2070)	6960 (6050–8000)	32·0 (26·9–37·3)	618 (419–870)	197 (132–283)	815 (557–1140)
Ukraine	1520 (1320–1750)	427 (12–927)	1940 (1470–2540)	28·2 (0·8–60·9)	177 (122–248)	50 (1·38–114)	227 (146–340)
**High–income**	**58200 (50100–67700)**	**14700 (11300–18200)**	**72900 (62800–85100)**	**25·2 (20·3–30·7)**	**6820 (4650–9540)**	**1720 (1130–2550)**	**8540 (5830–12000)**
**Australasia**	**1860 (1540–2270)**	**203 (–159–633)**	**2060 (1560–2710)**	**10·9 (–8·7–34·8)**	**219 (146–315)**	**24·2 (–18·4–83·1)**	**244 (154–365)**
Australia	1490 (1210–1840)	173 (–177–597)	1660 (1200–2250)	11·6 (–12·3–40·8)	175 (116–253)	20·6 (–19·8–77·6)	196 (122–304)
New Zealand	375 (322–437)	29·3 (–38·9–111)	404 (317–512)	7·9 (–10·7–30·4)	44·2 (29·6–61·2)	3·44 (–4·6–13·7)	47·7 (31·5–69·5)
**High income Asia Pacific**	**5170 (4430–6040)**	**639 (128–1260)**	**5810 (4830–6930)**	**12·4 (2·4–23·9)**	**615 (416–873)**	**76·1 (15·1–162)**	**691 (456–989)**
Brunei	13·7 (11–17·1)	1·39 (–2·05–5·54)	15·1 (10·7–21·3)	10·1 (–15·4–40·3)	1·67 (1·07–2·43)	0·167 (–0·261–0·71)	1·83 (1·08–2·82)
Japan	3130 (2740–3540)	396 (233–587)	3520 (3040–4060)	12·7 (7·6–18·5)	371 (253–518)	47 (22·6–79·3)	418 (280–590)
Singapore	170 (137–211)	30·7 (–13·5–85·2)	201 (145–272)	18·2 (–8·2–50·8)	20·5 (13·5–29·5)	3·71 (–1·76–11·1)	24·2 (14·6–37·3)
South Korea	1860 (1510–2300)	210 (–276–778)	2070 (1470–2810)	11·3 (–14·3–41·0)	223 (147–324)	25·2 (–33–95·5)	248 (151–377)
**High income North America**	**21700 (18700–25200)**	**6020 (4550–7610)**	**27700 (23700–32200)**	**27·8 (21·7–34·1)**	**2520 (1720–3520)**	**699 (445–1040)**	**3220 (2190–4510)**
Canada	1670 (1350–2070)	348 (–85·7–913)	2010 (1460–2740)	20·9 (–5·1–52·5)	197 (129–285)	41 (–9·02–110)	238 (145–367)
Greenland	2·88 (2·28–3·59)	0·502 (–0·236–1·34)	3·38 (2·44–4·53)	17·5 (–7·3–47·9)	0·34 (0·222–0·493)	0·0594 (–0·0242–0·178)	0·399 (0·245–0·6)
USA	20000 (17300–23100)	5670 (4240–7220)	25700 (22000–29800)	28·4 (22·2–35·2)	2320 (1590–3230)	658 (420–971)	2980 (2040–4180)
**Southern Latin America**	**3600 (3170–4070)**	**1320 (499–2180)**	**4920 (3930–5990)**	**36·8 (13·9–60·0)**	**428 (294–603)**	**157 (56–289)**	**585 (380–845)**
Argentina	2350 (2110–2580)	898 (170–1670)	3250 (2470–4140)	38·2 (7·2–70·9)	280 (194–392)	107 (20·5–220)	387 (245–582)
Chile	1060 (860–1310)	396 (75·1–780)	1460 (1080–1980)	37·4 (7·2–74·3)	125 (82·2–180)	46·7 (8·07–97·1)	172 (104–263)
Uruguay	186 (152–228)	30·5 (–19–82·6)	217 (156–293)	16·4 (–10·5–43·4)	22·1 (14·5–31·6)	3·63 (–2·24–10·6)	25·7 (15·4–39·5)
**Western Europe**	**25900 (21800–30600)**	**6470 (4210–9120)**	**32400 (27100–38700)**	**25·0 (16·6–34·1)**	**3040 (2060–4260)**	**759 (435–1180)**	**3800 (2560–5410)**
Andorra	4·94 (4·04–6·06)	1·65 (0·205–3·27)	6·59 (4·75–8·76)	33·4 (4·4–66·4)	0·583 (0·383–0·831)	0·193 (0·0206–0·416)	0·777 (0·491–1·16)
Austria	553 (452–676)	124 (–18·8–285)	677 (501–886)	22·5 (–3·1–51·5)	65·3 (44·1–93·7)	14·6 (–1·96–34)	79·9 (48·7–119)
Belgium	614 (499–745)	180 (–4·18–394)	794 (567–1050)	29·3 (–0·7–60·8)	72·2 (48–103)	21·2 (–0·512–48·2)	93·4 (58·7–144)
Cyprus	84·2 (68–103)	16 (–4·17–40·5)	100 (72·6–133)	18·9 (–5·0–46·6)	10 (6·61–14·4)	1·9 (–0·49–5·09)	11·9 (7·42–18·2)
Denmark	315 (258–382)	57·8 (–23·5–161)	373 (269–509)	18·3 (–7·5–51·0)	37·2 (24·6–53·4)	6·77 (–2·84–19·8)	43·9 (26·7–66·5)
Finland	232 (190–280)	26·1 (–29·6–94·9)	258 (182–347)	11·2 (–12·6–40·0)	27·3 (18–38·7)	3·07 (–3·59–12·2)	30·3 (19–46·4)
France	4380 (3590–5310)	1430 (217–2920)	5820 (4230–7780)	32·7 (5·0–68·9)	515 (344–731)	168 (24·4–382)	683 (430–1050)
Germany	5670 (4610–6860)	933 (–441–2650)	6600 (4890–8770)	16·5 (–8·0–46·7)	662 (438–943)	109 (–49·7–328)	771 (464–1150)
Greece	615 (501–740)	121 (–36·3–311)	735 (537–984)	19·7 (–6·0–50·1)	72·2 (48·1–103)	14·2 (–4·18–38·6)	86·4 (54·3–129)

Iceland	19·1 (15·5–23)	3·63 (–1·23–8·98)	22·7 (16·6–29·9)	19·1 (–6·7–47·5)	2·27 (1·5–3·23)	0·432 (–0·143–1·19)	2·7 (1·64–4·09)
Ireland	331 (268–402)	88·4 (–2·21–189)	419 (301–555)	26·7 (–0·7–57·6)	39·2 (26–56·1)	10·4 (–0·283–23·5)	49·6 (31·1–73·6)
Israel	360 (295–437)	99·6 (–0·556–230)	459 (334–626)	27·7 (–0·1–62·6)	43 (28·6–61·5)	11·9 (–0·0872–27·9)	54·9 (33·9–83·1)
Italy	3490 (3040–3970)	858 (552–1200)	4350 (3730–5000)	24·6 (16·0–33·8)	409 (278–575)	100 (60·6–161)	510 (345–714)
Luxembourg	35·7 (29–43·6)	9·85 (–0·0227–21·7)	45·6 (32·3–61·3)	27·5 (–0·1–57·8)	4·22 (2·78–6·03)	1·16 (–0·00292–2·59)	5·39 (3·24–8·16)
Malta	27·1 (22–33·1)	5·18 (–1·99–13·2)	32·3 (23·6–42·8)	19·2 (–6·9–48·1)	3·2 (2·13–4·6)	0·609 (–0·227–1·68)	3·81 (2·35–5·79)
Monaco	2·16 (1·77–2·62)	0·468 (–0·0873–1·12)	2·63 (1·9–3·46)	21·7 (–4·7–53·2)	0·254 (0·167–0·366)	0·0548 (–0·00984–0·134)	0·308 (0·188–0·46)
Netherlands	1170 (951–1410)	317 (12·9–697)	1480 (1100–1960)	27·3 (1·0–58·4)	138 (92–197)	37·3 (1·43–86·1)	175 (111–260)
Norway	383 (333–437)	64·6 (29·6–101)	447 (384–524)	16·9 (7·8–25·9)	45·3 (31–63·5)	7·64 (3·22–13·4)	53 (35·9–73·9)
Portugal	877 (715–1070)	257 (24·9–543)	1130 (845–1490)	29·3 (3·1–62·0)	103 (68·2–147)	30·2 (3·29–68·3)	133 (82·3–206)
San Marino	1·92 (1·57–2·34)	0·621 (0·0346–1·31)	2·55 (1·84–3·39)	32·3 (1·7–67·7)	0·226 (0·151–0·32)	0·0726 (0·00363–0·158)	0·299 (0·186–0·44)
Spain	2330 (1900–2810)	723 (90·5–1540)	3050 (2230–4110)	31·1 (3·7–64·6)	274 (178–388)	85·2 (10·4–184)	359 (213–527)
Sweden	544 (476–619)	127 (7·25–272)	671 (533–832)	23·3 (1·4–50·9)	64·4 (43·5–89·9)	14·9 (0·726–34·5)	79·3 (50·4–116)
Switzerland	632 (518–765)	95·7 (–56·4–291)	728 (527–993)	15·1 (–9·2–44·9)	74·3 (49·7–106)	11·3 (–6·59–36·7)	85·6 (51·3–131)
UK	3220 (2790–3670)	924 (767–1110)	4140 (3610–4760)	28·8 (24·6–33·0)	378 (258–529)	109 (71·6–157)	487 (329–680)
**Latin America and Caribbean**	**33400 (28500–39500)**	**10600 (8360–13000)**	**44000 (37500–51500)**	**31·7 (25·8–37·7)**	**3970 (2680–5620)**	**1260 (836–1800)**	**5230 (3540–7330)**
**Andean Latin America**	**3410 (2740–4280)**	**1770 (1050–2660)**	**5180 (4050–6560)**	**52·2 (30·7–75·6)**	**409 (267–595)**	**212 (114–343)**	**621 (403–925)**
Bolivia	624 (500–779)	316 (107–582)	940 (679–1270)	50·8 (17·8–91·4)	74·8 (48·8–109)	37·8 (12·6–76)	113 (69·9–171)
Ecuador	923 (739–1160)	445 (159–812)	1370 (991–1840)	48·3 (18·7–86·6)	111 (72·3–161)	53·3 (18·9–102)	164 (101–244)
Peru	1860 (1490–2330)	1010 (404–1730)	2870 (2100–3830)	54·5 (22·3–92·8)	223 (145–327)	121 (45·6–230)	345 (217–517)
Caribbean	2140 (1730–2620)	490 (209–815)	2630 (2100–3330)	23·0 (10·2–37·7)	254 (167–367)	58·4 (23·8–110)	313 (201–453)
Antigua and Barbuda	4·2 (3·39–5·19)	0·903 (–0·216–2·25)	5·1 (3·67–6·9)	21·5 (–6·0–52·3)	0·5 (0·329–0·724)	0·108 (–0·0242–0·284)	0·608 (0·359–0·938)
Bahamas	18·1 (14·6–22·5)	6·89 (1·51–13·7)	25 (18·2–33·8)	38·1 (9·3–73·4)	2·16 (1·4–3·11)	0·819 (0·19–1·65)	2·98 (1·88–4·52)
Barbados	14·2 (11·4–17·5)	2·77 (–0·929–6·96)	17 (12·4–22·2)	19·6 (–6·5–52·7)	1·68 (1·11–2·43)	0·328 (–0·109–0·886)	2·01 (1·22–3·13)
Belize	18 (14·5–22·7)	6·98 (1·45–13·7)	25 (17·7–34·1)	38·8 (8·5–73·6)	2·16 (1·41–3·15)	0·835 (0·171–1·71)	3 (1·83–4·53)
Bermuda	3·07 (2·51–3·78)	0·654 (–0·154–1·65)	3·73 (2·7–5·06)	21·3 (–5·2–52·7)	0·364 (0·238–0·52)	0·0773 (–0·0174–0·207)	0·441 (0·264–0·67)
Cuba	532 (431–652)	103 (–38·1–288)	635 (460–876)	19·4 (–7·3–51·6)	62·8 (42–89·9)	12·3 (–4·19–35·6)	75·1 (46–116)
Dominica	3·12 (2·54–3·83)	0·641 (–0·194–1·71)	3·76 (2·73–5·14)	20·5 (–6·6–52·9)	0·371 (0·245–0·538)	0·0755 (–0·0245–0·207)	0·446 (0·272–0·682)
Dominican Republic	487 (392–606)	157 (18·1–324)	643 (459–867)	32·3 (3·5–66·7)	58·3 (37·9–85)	18·9 (2·11–41·6)	77·2 (45·7–120)
Grenada	4·68 (3·77–5·79)	0·896 (–0·405–2·36)	5·58 (3·93–7·57)	19·2 (–8·2–49·5)	0·558 (0·364–0·815)	0·107 (–0·0488–0·3)	0·665 (0·401–1·01)
Guyana	34·1 (27·3–42·3)	9·96 (0·684–22)	44 (31·6–60·7)	29·3 (2·2–63·2)	4·04 (2·66–5·89)	1·18 (0·0819–2·73)	5·22 (3·15–8)
Haiti	546 (436–683)	87·7 (–54·3–262)	634 (449–863)	16·2 (–10·1–47·8)	65 (42·5–94·8)	10·4 (–6·69–31·6)	75·4 (45·4–115)

Jamaica	125 (100–154)	25·4 (–6·54–62·9)	150 (108–203)	20·4 (–4·9–49·4)	14·9 (9·7–21·6)	3·05 (–0·812–8·14)	17·9 (10·7–27·9)
Puerto Rico	170 (138–208)	46·8 (–1·19–106)	217 (157–291)	27·6 (–0·8–60·5)	20 (13·1–28·9)	5·48 (–0·145–12·4)	25·5 (16–38·5)
Saint Kitts and Nevis	2·79 (2·24–3·46)	0·564 (–0·182–1·43)	3·36 (2·42–4·54)	20·2 (–5·9–49·5)	0·332 (0·216–0·479)	0·0671 (–0·02–0·176)	0·399 (0·245–0·618)
Saint Lucia	8·32 (6·77–10·3)	1·68 (–0·482–4·36)	10 (7·29–13·4)	20·3 (–5·9–52·6)	0·985 (0·652–1·42)	0·199 (–0·0565–0·539)	1·18 (0·74–1·81)
Saint Vincent and the Grenadines	5·18 (4·21–6·38)	0·962 (–0·391–2·68)	6·14 (4·41–8·37)	18·6 (–7·6–51·0)	0·615 (0·402–0·878)	0·114 (–0·0448–0·335)	0·729 (0·44–1·12)
Suriname	25·3 (20·6–31·3)	6·97 (–0·315–15·2)	32·3 (23·3–43·6)	27·5 (–1·2–58·9)	3 (1·96–4·36)	0·824 (–0·0351–2·03)	3·83 (2·32–5·89)
Trinidad and Tobago	61 (49–74·9)	13·2 (–3·36–34·3)	74·2 (53·3–103)	21·6 (–5·7–54·5)	7·21 (4·71–10·5)	1·57 (–0·377–4·29)	8·77 (5·26–13·5)
Virgin Islands	4·79 (3·88–5·88)	0·632 (–0·585–2·17)	5·42 (3·89–7·44)	13·2 (–12·4–44·6)	0·563 (0·375–0·809)	0·074 (–0·0692–0·266)	0·637 (0·395–0·98)
**Central Latin America**	**10100 (8520–12000)**	**3790 (2740–4930)**	**13900 (11500–16600)**	**37·5 (28·3–47·0)**	**1210 (810–1720)**	**453 (287–683)**	**1660 (1110–2380)**
Colombia	2330 (1860–2870)	822 (148–1600)	3150 (2300–4150)	35·4 (6·6–68·8)	279 (181–399)	98·6 (14·7–202)	377 (234–575)
Costa Rica	208 (168–259)	73·7 (8·51–148)	281 (202–385)	35·6 (4·4–70·9)	24·8 (16·2–35·9)	8·76 (1·03–18·6)	33·5 (20·4–51·1)
El Salvador	273 (220–343)	69·7 (–7·56–164)	343 (240–464)	25·6 (–2·6–57·7)	32·7 (21·5–47·2)	8·35 (–0·85–20·3)	41·1 (24·8–62)
Guatemala	647 (521–821)	256 (58·8–511)	903 (636–1250)	39·4 (9·2–75·4)	77·4 (50–113)	30·5 (6·66–64·2)	108 (67–167)
Honduras	400 (319–499)	185 (48·3–349)	585 (413–798)	46·4 (13·3–85·6)	48·1 (31–69·7)	22·4 (6·11–46·2)	70·5 (40·6–110)
Mexico	4640 (3970–5370)	1840 (1440–2300)	6490 (5530–7550)	39·8 (32·8–48·0)	554 (374–782)	220 (144–317)	774 (526–1090)
Nicaragua	279 (227–351)	71·5 (–8·68–175)	351 (241–493)	25·5 (–3·5–59·5)	33·5 (21·9–48·7)	8·6 (–0·902–21·8)	42·1 (25·2–66·4)
Panama	164 (133–203)	92·1 (34·6–167)	256 (186–349)	56·2 (21·6–95·7)	19·7 (12·9–28·4)	11 (3·89–21·1)	30·7 (18·7–45·9)
Venezuela	1180 (952–1460)	377 (39·5–821)	1560 (1120–2090)	32·0 (3·3–66·0)	141 (92·7–206)	45·2 (4·49–101)	187 (116–284)
**Tropical Latin America**	**17800 (15300–20600)**	**4520 (3080–6060)**	**22300 (19100–26000)**	**25·5 (17·9–33·7)**	**2100 (1420–2940)**	**533 (317–821)**	**2630 (1790–3670)**
Brazil	17300 (15000–20000)	4380 (2960–5870)	21700 (18600–25200)	25·4 (17·7–33·9)	2040 (1380–2860)	516 (306–788)	2550 (1740–3560)
Paraguay	488 (392–604)	148 (7·63–305)	635 (466–853)	30·3 (1·6–62·1)	58·2 (37·9–84·6)	17·6 (1–39·7)	75·9 (48·3–118)
**North Africa and Middle East**	**32100 (26300–39300)**	**10400 (7350–14100)**	**42500 (34700–52400)**	**32·4 (24·9–41·1)**	**3850 (2550–5510)**	**1240 (777–1900)**	**5090 (3390–7280)**
Afghanistan	1820 (1420–2290)	524 (1·14–1180)	2340 (1640–3300)	28·8 (0·1–61·4)	219 (139–325)	62·4 (0·0693–146)	281 (172–435)
Algeria	2140 (1720–2650)	520 (–78·5–1220)	2660 (1920–3570)	24·4 (–3·6–58·3)	256 (167–373)	62·5 (–8·84–156)	319 (189–478)
Bahrain	76·8 (60·8–95·2)	20·6 (–1·7–46·8)	97·3 (67·1–131)	26·8 (–2·2–58·3)	9·19 (5·97–13·2)	2·45 (–0·229–5·89)	11·6 (7·01–17·7)
Egypt	4490 (3590–5630)	1670 (277–3520)	6160 (4350–8540)	37·2 (7·1–73·7)	541 (353–795)	201 (37–428)	742 (451–1140)
Iran	6090 (5230–7020)	2380 (1830–3000)	8470 (7270–9900)	39·2 (31·4–47·6)	723 (490–1010)	283 (182–421)	1010 (682–1420)
Iraq	2330 (1850–2920)	792 (118–1640)	3130 (2230–4260)	34·0 (4·7–69·3)	281 (179–412)	94·7 (14·6–195)	375 (229–570)
Jordan	615 (494–768)	136 (–40·5–341)	751 (531–1050)	22·0 (–6·4–53·4)	74·4 (47·9–108)	16·5 (–4·04–43·8)	90·8 (54·6–142)
Kuwait	237 (186–298)	74·3 (7·2–157)	311 (221–426)	31·4 (2·7–64·2)	28·3 (18·5–41·4)	8·9 (0·815–20·7)	37·2 (22·5–55·9)
Lebanon	351 (283–433)	90·8 (–1·96–208)	442 (318–592)	25·9 (–0·7–57·8)	41·7 (27–60·4)	10·7 (–0·256–25)	52·5 (32·4–78·8)

Libya	384 (309–474)	103 (–0·655–235)	488 (354–664)	26·9 (–0·2–58·1)	46 (29·6–66·6)	12·4 (–0·182–29·2)	58·3 (34·5–88·4)
Morocco	1850 (1490–2290)	659 (95·3–1280)	2510 (1780–3400)	35·6 (5·4–67·4)	221 (142–320)	78·5 (10·5–165)	300 (182–452)
Oman	234 (182–295)	45·9 (–19–119)	280 (196–385)	19·7 (–7·3–48·6)	28·3 (18–41·4)	5·55 (–2·05–14·9)	33·8 (20·2–53)
Palestine	267 (211–336)	95 (13·8–191)	362 (252–495)	35·6 (5·3–73·3)	32·1 (20·4–46·5)	11·5 (1·51–25·9)	43·6 (26·2–67·3)
Qatar	141 (110–179)	43·9 (3·29–92·2)	185 (126–255)	31·1 (2·3–65·2)	17 (10·8–24·6)	5·28 (0·458–11·6)	22·3 (13·1–33·6)
Saudi Arabia	1870 (1480–2360)	642 (86·9–1350)	2510 (1760–3490)	34·3 (4·9–68·7)	224 (144–331)	77·1 (10·1–173)	301 (182–471)
Sudan	2020 (1610–2560)	584 (25·9–1320)	2610 (1860–3640)	28·9 (1·6–64·3)	244 (156–358)	69·9 (3·29–163)	314 (189–486)
Syria	811 (654–1010)	169 (–39–434)	980 (692–1340)	20·8 (–4·7–52·1)	96·7 (62·2–139)	20·1 (–4·76–53·8)	117 (71–184)
Tunisia	615 (498–758)	183 (1·61–399)	799 (566–1080)	29·8 (0·3–64·7)	73·3 (47·7–106)	21·8 (0·206–49·4)	95·1 (58·4–146)
Turkey	3800 (3090–4680)	1330 (212–2770)	5130 (3630–6910)	35·1 (5·5–71·2)	454 (298–649)	158 (24·6–339)	612 (384–902)
United Arab Emirates	437 (341–556)	105 (–11·7–258)	542 (379–761)	24·0 (–2·4–56·3)	52·4 (33·7–76·7)	12·6 (–1·46–31·7)	65 (38·2–101)
Yemen	1530 (1200–1940)	215 (–165–674)	1750 (1250–2380)	14·1 (–10·8–43·8)	183 (119–267)	25·7 (–21·2–84·5)	209 (125–317)
**South Asia**	**55000 (47200–64400)**	**19300 (14800–24000)**	**74300 (63000–87200)**	**35·1 (28·2–42·0)**	**6530 (4440–9160)**	**2280 (1480–3300)**	**8810 (5990–12400)**
Bangladesh	5130 (4110–6470)	1860 (222–3880)	6990 (4880–9620)	36·2 (4·6–75·3)	612 (400–889)	221 (24–506)	833 (502–1280)
Bhutan	24·7 (19·9–31)	3·89 (–2·89–11·5)	28·6 (19·8–38·9)	15·8 (–11·4–46·8)	2·95 (1·92–4·34)	0·463 (–0·305–1·46)	3·41 (2·02–5·33)
India	42200 (36300–48900)	14700 (11400–18300)	56900 (48300–66300)	34·8 (28·5–41·5)	4990 (3400–7010)	1740 (1140–2520)	6730 (4580–9450)
Nepal	966 (776–1220)	353 (49·3–691)	1320 (930–1780)	36·6 (4·8–71·5)	115 (74·9–168)	41·7 (5·66–85·5)	156 (97·1–238)
Pakistan	6730 (5760–7870)	2380 (1200–3880)	9110 (7310–11100)	35·4 (18·0–55·7)	803 (544–1130)	284 (124–503)	1090 (722–1570)
**Southeast Asia, east Asia, and Oceania**	**72500 (62500–83800)**	**10000 (6610–13600)**	**82500 (70700–96400)**	**13·8 (9·3–18·3)**	**8690 (5850–12300)**	**1200 (685–1830)**	**9890 (6630–14000)**
**East Asia**	**48100 (41700–55000)**	**5270 (2500–8080)**	**53400 (46000–61700)**	**11·0 (5·1–16·9)**	**5750 (3880–8120)**	**630 (259–1080)**	**6380 (4340–9010)**
China	46200 (40100–52800)	5160 (2460–7980)	51300 (44300–59300)	11·2 (5·3–17·3)	5520 (3720–7800)	617 (259–1050)	6140 (4160–8680)
North Korea	979 (795–1190)	67·9 (–184–367)	1050 (753–1430)	6·9 (–17·9–36·7)	118 (77·1–170)	8·02 (–23·3–46·7)	126 (76·8–202)
Taiwan (province of China)	922 (753–1120)	42·9 (–189–289)	965 (693–1290)	4·7 (–19·6–32·7)	110 (72·1–157)	5·17 (–23·6–36·6)	115 (67·5–176)
**Oceania**	**472 (377–593)**	**5·69 (–77·1–112)**	**478 (354–640)**	**1·2 (–16·7–22·9)**	**57 (36·3–83·2)**	**0·684 (–9·83–13·1)**	**57·7 (35·7–87·1)**
American Samoa	2·09 (1·7–2·59)	0·182 (–0·331–0·765)	2·27 (1·64–3·11)	8·7 (–15·4–37·0)	0·25 (0·164–0·36)	0·0218 (–0·0405–0·0943)	0·271 (0·166–0·416)
Cook Islands	0·715 (0·584–0·869)	0·0624 (–0·107–0·273)	0·778 (0·565–1·04)	8·8 (–15·1–37·1)	0·0847 (0·0556–0·123)	0·00738 (–0·0132–0·0335)	0·0921 (0·0571–0·142)
Federated States of Micronesia	3·85 (3·11–4·73)	0·12 (–0·727–1·31)	3·97 (2·78–5·41)	3·1 (–18·9–34·1)	0·463 (0·298–0·673)	0·0142 (–0·0909–0·155)	0·478 (0·292–0·735)
Fiji	34·3 (27·7–42)	2·61 (–5·68–12·5)	36·9 (26·5–50·2)	7·7 (–16·7–36·5)	4·11 (2·67–5·91)	0·313 (–0·723–1·61)	4·43 (2·75–6·89)
Guam	6·44 (5·25–7·85)	1·49 (–0·416–3·74)	7·92 (5·52–10·7)	23·2 (–6·5–56·1)	0·772 (0·507–1·12)	0·179 (–0·0501–0·477)	0·951 (0·579–1·48)
Kiribati	4·32 (3·45–5·37)	0·203 (–0·782–1·4)	4·52 (3·22–6·15)	4·7 (–18·0–30·9)	0·52 (0·332–0·751)	0·0247 (–0·0991–0·179)	0·545 (0·329–0·855)
Marshall Islands	2·07 (1·65–2·59)	0·0707 (–0·434–0·672)	2·14 (1·51–2·95)	3·4 (–20·7–30·6)	0·249 (0·161–0·36)	0·00861 (–0·0522–0·0842)	0·257 (0·15–0·404)

Nauru	0·389 (0·311–0·486)	0·0379 (–0·0575–0·158)	0·427 (0·296–0·603)	9·7 (–14·5–39·2)	0·0469 (0·0298–0·0685)	0·00454 (–0·00715–0·0198)	0·0515 (0·0315–0·0802)
Niue	0·0657 (0·0535–0·0801)	0·00562 (–0·0104–0·0243)	0·0713 (0·0515–0·0978)	8·5 (–15·2–36·3)	0·00779 (0·00515–0·0112)	0·000659 (–0·00131–0·00294)	0·00845 (0·00536–0·0129)
Northern Mariana Islands	1·71 (1·38–2·09)	0·343 (–0·0898–0·931)	2·05 (1·49–2·81)	20·1 (–5·9–53·9)	0·204 (0·134–0·296)	0·041 (–0·0105–0·115)	0·245 (0·152–0·377)
Palau	0·717 (0·577–0·877)	0·0543 (–0·114–0·259)	0·772 (0·558–1·05)	7·6 (–15·7–35·6)	0·085 (0·0563–0·123)	0·0064 (–0·0138–0·032)	0·0914 (0·0575–0·138)
Papua New Guinea	348 (275–437)	–0·666 (–80·1–93·8)	347 (247–482)	–0·3 (–23·1–27·2)	42 (26·6–61·2)	–0·087 (–10·3–11·8)	41·9 (25·5–64·5)
Samoa	7·52 (6·11–9·32)	0·166 (–1·57–2·39)	7·68 (5·44–10·7)	2·2 (–20·9–30·7)	0·907 (0·592–1·31)	0·0191 (–0·204–0·28)	0·926 (0·555–1·41)
Solomon Islands	23·3 (18·5–29·1)	0·248 (–5·1–5·88)	23·5 (16·7–32·2)	1·1 (–22·1–26·0)	2·81 (1·83–4·12)	0·0308 (–0·616–0·759)	2·84 (1·76–4·43)
Tokelau	0·052 (0·0424–0·0641)	0·0053 (–0·00762–0·0197)	0·0573 (0·0406–0·076)	10·3 (–14·6–38·8)	0·00622 (0·00412–0·00899)	0·000635 (–0·000913–0·00249)	0·00685 (0·00419–0·0104)
Tonga	3·73 (3–4·59)	0·347 (–0·576–1·46)	4·08 (2·88–5·5)	9·3 (–15·4–40·0)	0·45 (0·291–0·658)	0·0426 (–0·0702–0·186)	0·492 (0·299–0·77)
Tuvalu	0·453 (0·365–0·558)	0·0418 (–0·0654–0·178)	0·495 (0·353–0·677)	9·2 (–14·9–37·8)	0·0544 (0·0355–0·0787)	0·00505 (–0·00861–0·0232)	0·0595 (0·0359–0·0926)
Vanuatu	10·6 (8·45–13·2)	0·108 (–2·21–2·92)	10·7 (7·65–14·7)	1·1 (–21·1–28·0)	1·28 (0·822–1·85)	0·014 (–0·272–0·36)	1·3 (0·788–1·99)
**Southeast Asia**	**24000 (20300–28200)**	**4760 (3110–6640)**	**28700 (24200–34100)**	**19·9 (13·5–26·7)**	**2880 (1930–4090)**	**572 (321–884)**	**3450 (2280–4930)**
Cambodia	649 (526–805)	77·7 (–79·2–280)	727 (515–992)	12·0 (–12·0–42·2)	78 (50·8–113)	9·43 (–9·5–35)	87·5 (54·9–137)
Indonesia	9360 (8050–10800)	1900 (1090–2870)	11300 (9560–13300)	20·3 (12·2–29·0)	1130 (758–1580)	229 (113–374)	1360 (911–1920)
Laos	312 (257–383)	44·3 (–32·5–136)	357 (263–474)	14·2 (–10·5–44·3)	37·8 (24·8–55·2)	5·31 (–4·08–16·6)	43·1 (27·3–64·8)
Malaysia	1410 (1120–1760)	195 (–145–633)	1600 (1150–2250)	13·9 (–11·0–43·9)	169 (110–246)	23·4 (–16·8–81·1)	192 (114–293)
Maldives	17·8 (14·1–22·7)	4·29 (–0·54–10·7)	22 (15·8–30·7)	24·2 (–3·0–57·1)	2·15 (1·38–3·13)	0·517 (–0·0724–1·31)	2·66 (1·63–4·1)
Mauritius	50·4 (41·1–62·1)	7·59 (–5·18–23·1)	58 (42·2–79·4)	15·1 (–10·3–45·0)	5·95 (3·9–8·57)	0·893 (–0·557–2·8)	6·84 (4·21–10·7)
Myanmar	2040 (1640–2490)	549 (–32·1–1230)	2590 (1880–3540)	26·9 (–1·5–60·0)	245 (158–352)	66 (–4·27–157)	311 (183–479)
Philippines	4270 (3660–4930)	1430 (1140–1780)	5700 (4880–6640)	33·6 (27·8–40·0)	514 (347–724)	173 (113–253)	687 (464–968)
Seychelles	3·83 (3·09–4·72)	0·679 (–0·313–1·9)	4·5 (3·22–6·11)	17·8 (–7·7–47·9)	0·458 (0·301–0·66)	0·0813 (–0·0344–0·239)	0·539 (0·322–0·828)
Sri Lanka	859 (706–1050)	165 (–60·8–444)	1020 (730–1390)	19·1 (–7·1–51·2)	102 (66·9–148)	19·5 (–7·59–55·8)	122 (72·9–187)
Thailand	2670 (2150–3300)	213 (–457–1030)	2880 (2020–3940)	7·9 (–17·0–37·5)	317 (208–457)	25·2 (–56·9–121)	342 (206–513)
Timor–Leste	43·8 (35·1–54·5)	6·78 (–5–20·2)	50·6 (36·1–67·5)	15·6 (–11·8–44·9)	5·3 (3·42–7·76)	0·826 (–0·595–2·62)	6·12 (3·68–9·7)
Vietnam	2240 (1800–2770)	155 (–380–815)	2400 (1690–3270)	6·9 (–17·2–34·5)	270 (178–390)	18·8 (–45·5–101)	289 (175–448)
**Sub–Saharan Africa**	**33100 (27200–40400)**	**7100 (5280–9140)**	**40200 (32900–49300)**	**21·5 (17·1–25·7)**	**3990 (2620–5720)**	**854 (551–1280)**	**4840 (3180–6980)**
**Central sub–Saharan Africa**	**4200 (3350–5320)**	**821 (10·6–1760)**	**5030 (3770–6630)**	**19·6 (0·3–41·5)**	**505 (325–731)**	**98·5 (0·921–224)**	**603 (378–901)**
Angola	963 (760–1230)	191 (–52·4–507)	1150 (817–1580)	19·9 (–5·6–52·1)	116 (73·7–169)	23 (–5·96–62·7)	139 (82·6–210)
Central African Republic	187 (148–237)	28·8 (–19·5–87·2)	216 (155–300)	15·5 (–11·0–44·0)	22·3 (14·3–32·5)	3·45 (–2·26–10·6)	25·8 (15·3–39·2)
Congo	177 (141–222)	38 (–9·09–95·7)	215 (153–298)	21·4 (–4·9–52·6)	21·2 (13·6–31·1)	4·54 (–1·09–12·5)	25·8 (15·4–39·6)
Democratic Republic of the	2770 (2190–3510)	544 (–193–1390)	3310 (2370–4460)	19·7 (–7·0–50·7)	332 (212–480)	65·1 (–21·6–177)	397 (242–620)

Congo							
Equatorial Guinea	49 (38·5–63·3)	9·33 (–3·8–25·4)	58·3 (40·5–81·4)	19·0 (–7·9–48·4)	5·9 (3·75–8·57)	1·12 (–0·49–3·13)	7·02 (4·18–10·7)
Gabon	62·4 (49·5–78·7)	10·7 (–5·97–30·2)	73·1 (51·6–101)	17·1 (–9·4–47·0)	7·46 (4·78–10·7)	1·28 (–0·685–3·57)	8·74 (5·07–13·3)
**Eastern sub–Saharan Africa**	**13300 (10900–16500)**	**2500 (1530–3630)**	**15800 (12900–19600)**	**18·8 (11·9–26·3)**	**1610 (1050–2320)**	**301 (163–500)**	**1910 (1260–2760)**
Burundi	373 (295–475)	38 (–55·4–151)	411 (295–575)	10·2 (–15·3–38·8)	44·9 (28·7–65·5)	4·53 (–7·32–18·3)	49·4 (30·1–77·1)
Comoros	24·9 (19·8–31·5)	5·01 (–1·57–13·4)	29·9 (20·8–41·2)	20·1 (–6·2–52·5)	3·01 (1·92–4·38)	0·604 (–0·181–1·69)	3·61 (2·16–5·63)
Djibouti	40 (31·5–50·7)	6·91 (–3·9–19·5)	46·9 (32·8–64·4)	17·3 (–10·1–47·9)	4·84 (3·11–7·02)	0·834 (–0·47–2·5)	5·67 (3·4–9·08)
Eritrea	217 (171–275)	23·6 (–29·1–86·6)	240 (168–339)	10·9 (–13·4–39·6)	26·1 (16·7–38·2)	2·85 (–3·3–11·1)	29 (17·5–45·3)
Ethiopia	3390 (2880–4000)	659 (215–1160)	4050 (3270–4960)	19·4 (6·2–32·9)	411 (275–587)	79·8 (24–153)	491 (328–699)
Kenya	1590 (1350–1870)	485 (377–603)	2070 (1760–2430)	30·6 (25·0–36·5)	192 (129–272)	58·6 (37·4–85·1)	250 (168–354)
Madagascar	945 (744–1210)	171 (–62·8–463)	1120 (780–1540)	18·1 (–7·1–46·5)	114 (72·6–168)	20·6 (–7·7–58·6)	135 (82·1–211)
Malawi	621 (491–794)	115 (–43·8–317)	736 (513–1000)	18·5 (–7·1–49·8)	75·1 (47·9–110)	13·7 (–5·02–38·5)	88·8 (52·5–136)
Mozambique	957 (756–1220)	146 (–93·7–447)	1100 (759–1520)	15·2 (–10·1–46·8)	114 (72·8–165)	17·5 (–10·8–55·5)	131 (79·3–203)
Rwanda	431 (343–550)	72 (–42·9–222)	503 (349–701)	16·6 (–10·0–47·5)	51·9 (33·3–75·8)	8·6 (–5·42–26·7)	60·5 (36·2–93·2)
Somalia	590 (464–748)	95·5 (–54–271)	685 (465–951)	16·2 (–10·5–47·1)	71·2 (45·1–106)	11·5 (–7·17–34·9)	82·8 (49·1–129)
South Sudan	347 (274–438)	49·5 (–37·6–154)	396 (279–545)	14·3 (–11·3–42·3)	41·6 (26·7–61)	5·88 (–4·62–19·2)	47·5 (28·5–72·4)
Uganda	1320 (1030–1690)	279 (–65–728)	1600 (1100–2200)	21·2 (–4·9–51·7)	159 (101–236)	33·5 (–8·27–92·5)	193 (119–307)
Tanzania	1850 (1480–2370)	291 (–166–875)	2150 (1490–2990)	15·6 (–8·9–45·4)	224 (143–325)	35·2 (–20·8–109)	259 (157–409)
Zambia	625 (492–797)	62·8 (–87·9–251)	688 (495–972)	10·0 (–14·2–38·2)	75·2 (48·1–111)	7·43 (–10·1–30·7)	82·7 (50·7–127)
**Southern sub–Saharan Africa**	**2870 (2420–3410)**	**1000 (684–1380)**	**3870 (3230–4670)**	**35·0 (24·5–46·4)**	**340 (228–480)**	**119 (73·7–187)**	**459 (311–654)**
Botswana	83·6 (66·7–106)	9·4 (–12–34)	93 (66–130)	11·3 (–14·0–39·6)	9·97 (6·41–14·4)	1·12 (–1·4–4·22)	11·1 (6·65–17·4)
Eswatini	38·5 (30·8–48·6)	12·2 (1·43–26·3)	50·7 (36–70·3)	31·7 (4·3–65·9)	4·59 (2·91–6·69)	1·45 (0·158–3·27)	6·04 (3·6–9·3)
Lesotho	64·3 (51·2–80·8)	15 (–2·95–36)	79·3 (55·7–109)	23·4 (–4·1–57·1)	7·64 (4·89–11)	1·79 (–0·334–4·61)	9·42 (5·69–14·6)
Namibia	82 (65·1–103)	23·6 (–0·0725–54·2)	106 (74·9–146)	28·7 (–0·1–63·6)	9·85 (6·43–14·3)	2·82 (–0·00936–6·67)	12·7 (7·8–19·8)
South Africa	2150 (1830–2490)	856 (578–1180)	3010 (2500–3570)	39·9 (26·9–53·8)	254 (171–355)	101 (61·7–160)	355 (244–509)
Zimbabwe	453 (357–576)	86·1 (–30·1–234)	539 (381–742)	19·1 (–6·6–50·4)	54·5 (34·9–79·2)	10·4 (–3·5–29·8)	64·9 (38·8–102)
**Western sub–Saharan Africa**	**12700 (10400–15400)**	**2770 (2040–3610)**	**15400 (12600–18800)**	**21·9 (17·0–27·5)**	**1530 (999–2210)**	**336 (204–523)**	**1870 (1230–2730)**
Benin	352 (276–449)	34·4 (–54·7–133)	387 (270–539)	9·8 (–15·4–37·7)	42·8 (27–63·1)	4·25 (–6·4–17·9)	47 (28·4–73·9)
Burkina Faso	620 (482–787)	71·8 (–84–284)	691 (465–993)	11·4 (–14·4–44·4)	75·1 (47·2–110)	8·68 (–10·5–34·3)	83·8 (48·3–136)
Cabo Verde	17 (13·6–21·3)	8·1 (2·35–16)	25·1 (18·1–35·3)	47·7 (13·5–89·8)	2·06 (1·33–2·96)	0·973 (0·256–1·98)	3·03 (1·84–4·55)
Cameroon	859 (670–1100)	95·3 (–123–354)	954 (665–1340)	11·1 (–13·9–42·3)	104 (65·2–156)	11·6 (–14·9–45·9)	116 (67·5–180)

Chad	445 (347–569)	62·5 (–49·1–208)	507 (352–710)	14·0 (–11·7–48·8)	54 (34·5–79·3)	7·59 (–6·34–26·6)	61·5 (36·1–96·4)
Côte d'Ivoire	748 (590–951)	91·5 (–95·5–336)	840 (584–1170)	12·3 (–13·4–45·1)	90·6 (57·4–134)	11·2 (–11·2–42·7)	102 (62·6–159)
The Gambia	66·5 (52·3–84·3)	11·8 (–6·59–34·3)	78·3 (53·8–110)	17·7 (–10·0–49·4)	8·04 (5·06–11·8)	1·43 (–0·759–4·5)	9·47 (5·37–15·1)
Ghana	910 (718–1150)	149 (–96·2–462)	1060 (749–1480)	16·5 (–10·8–50·1)	110 (70·5–160)	18·2 (–10·6–59·4)	128 (73·9–203)
Guinea	358 (283–462)	59·9 (–33·3–179)	418 (286–585)	16·7 (–8·8–46·9)	43·4 (27·3–63·3)	7·24 (–3·95–22·2)	50·6 (29·5–80·3)
Guinea–Bissau	54·4 (42·2–69·3)	8·84 (–4·77–26·6)	63·3 (44–88)	16·2 (–9·2–48·7)	6·59 (4·12–9·73)	1·08 (–0·55–3·25)	7·67 (4·47–12·2)
Liberia	150 (118–189)	25·4 (–14–78)	175 (122–246)	16·8 (–9·8–49·0)	17·9 (11·4–26·2)	3·04 (–1·73–9·46)	20·9 (12·4–32·9)
Mali	554 (435–700)	103 (–48·1–284)	657 (462–910)	18·7 (–9·0–50·4)	67·1 (42·5–98·6)	12·4 (–5·76–35)	79·5 (47·3–123)
Mauritania	109 (86·5–137)	23 (–7·2–56·4)	132 (93–181)	21·2 (–6·9–52·2)	13·2 (8·35–19·3)	2·8 (–0·8–7·47)	16 (9·41–25·1)
Niger	586 (456–747)	64·6 (–72·2–245)	651 (455–875)	11·2 (–12·5–41·4)	71·5 (45–105)	7·97 (–8·8–30·4)	79·4 (47·6–125)
Nigeria	5910 (5010–6920)	1770 (1350–2260)	7690 (6470–9090)	30·0 (24·3–36·0)	715 (473–1020)	214 (138–311)	928 (625–1330)
São Tomé and Príncipe	6·06 (4·8–7·72)	1·12 (–0·489–3·06)	7·18 (5·07–10)	18·5 (–7·8–49·4)	0·736 (0·469–1·06)	0·136 (–0·057–0·392)	0·872 (0·526–1·36)
Senegal	413 (327–519)	122 (3·12–276)	535 (372–747)	29·5 (0·9–66·4)	49·9 (31·6–73·7)	14·8 (0·403–35·4)	64·6 (38·3–102)
Sierra Leone	258 (203–331)	37 (–29·8–113)	295 (203–406)	14·3 (–11·6–42·3)	31·2 (19·7–46·1)	4·47 (–3·4–14·6)	35·7 (21·1–55·6)
Togo	243 (194–309)	30·9 (–31·3–110)	274 (190–382)	12·7 (–13·2–42·6)	29·4 (18·7–42·9)	3·72 (–3·88–13·6)	33·1 (19·5–51·1)

Table S10: Meta-regression coefficients from initial and final prevalence-adjustment models for MDD and anxiety disorders

Disorder	Covariate	Initial model			Final model		
		B	*p*	AIC	*B*	*p*	AIC
MDD	COVID-19 impact index	0·4	0·003	615·6	0·4	0·0044	609·9
	Mid age	> -0·1	< 0·001		> -0·1	< 0·001	
	Percent female	0·1	< 0·001		0·1	< 0·001	
	Combined depressive and anxiety disorder symptoms	< 0·1	0·857		-	-	
	Cross-sectional random sample	> -0·1	0·978		-	-	
	Longitudinal market research / quota sample	-0·2	0·590		-	-	
	Cross-sectional market research / quota sample	0·9	< 0·001		0·9	< 0·001	
Anxiety disorders	COVID-19 impact index	0·4	< 0·001	270·6	0·4	< 0·001	267·3
	Mid age	> -0·1	< 0·001		> -0·1	< 0·001	
	Percent female	0·1	< 0·001		0·1	< 0·001	
	Combined depressive and anxiety disorder symptoms	0·3	0·007		0·3	0·008	
	Cross-sectional random sample	-0·1	0·880		-	-	
	Longitudinal market research / quota sample	-0·4	0·426		-	-	
	Cross-sectional market research / quota sample	0·6	0·004		-0·6	0·002	

MDD = Major depressive disorder. AIC = Akaike information criterion.

Table S11: Leave-one-country-out cross-validation analysis results

Disorder	Location	Study	Mid age	% female	Observation (CI)	Prediction (PI)	Outside PI	LOCOCV RMSE	Benchmark RMSE
MDD	USA	Marroquin et al	46	50	-0·04 (-0·58 to 0·5)	-0·38 (-1·72 to 0·96)	No	0·66	0·65
		Wanberg et al	56	56	-0·2 (-0·45 to 0·06)	-0·35 (-1·7 to 1)	No		
		Katz et al	46	0	0·3 (-0·78 to 1·38)	-0·35 (-1·69 to 1)	No		
		Katz et al	46	0	0·27 (-0·76 to 1·3)	-0·35 (-1·69 to 1)	No		
		Katz et al	46	100	0·13 (-0·87 to 1·13)	-0·43 (-1·77 to 0·91)	No		
		Katz et al	46	100	0·22 (-0·7 to 1·14)	-0·43 (-1·77 to 0·91)	No		
		McGinty et al (a)	24	0	-2·24 (-2·92 to -1·55)	-1·3 (-2·58 to -0·02)	No		
		McGinty et al (a)	24	100	-2·04 (-2·7 to -1·37)	-1·38 (-2·66 to -0·1)	No		
		McGinty et al (a)	42	0	-1·5 (-2·05 to -0·94)	-1·21 (-2·49 to 0·07)	No		
		McGinty et al (a)	42	100	-1·3 (-1·83 to -0·77)	-1·29 (-2·56 to -0·01)	No		
		McGinty et al (a)	77	0	-0·82 (-1·45 to -0·19)	-1·04 (-2·32 to 0·24)	No		
		McGinty et al (a)	77	100	-0·62 (-1·23 to -0·01)	-1·12 (-2·39 to 0·16)	No		
		Twenge et al	24	0	-2·57 (-2·91 to -2·23)	-1·36 (-2·65 to -0·06)	No		
		Twenge et al	37	0	-2·46 (-2·77 to -2·16)	-1·28 (-2·57 to 0·01)	No		
		Twenge et al	52	0	-1·87 (-2·2 to -1·54)	-1·2 (-2·49 to 0·09)	No		
		Twenge et al	52	100	-2·49 (-2·82 to -2·16)	-1·29 (-2·57 to 0)	No		
		Twenge et al	80	0	-1·3 (-1·71 to -0·88)	-1·05 (-2·34 to 0·25)	No		
		Twenge et al	80	100	-1·92 (-2·34 to -1·5)	-1·13 (-2·42 to 0·16)	No		
		McGinty et al (b)	24	0	-2·48 (-3·33 to -1·63)	-1·22 (-2·49 to 0·06)	No		
		McGinty et al (b)	24	100	-2·07 (-2·9 to -1·24)	-1·28 (-2·55 to 0)	No		
		McGinty et al (b)	42	0	-1·39 (-2 to -0·77)	-1·14 (-2·42 to 0·13)	No		
		McGinty et al (b)	42	100	-0·98 (-1·56 to -0·39)	-1·21 (-2·48 to 0·07)	No		
		McGinty et al (b)	77	0	-0·87 (-1·59 to -0·15)	-1·01 (-2·28 to 0·27)	No		
		McGinty et al (b)	77	100	-0·46 (-1·15 to 0·23)	-1·07 (-2·34 to 0·21)	No		
		Bryan et al	58	51	-1·41 (-1·51 to -1·3)	-1·12 (-2·39 to 0·15)	No		
		Kantor et al	58	0	-1·19 (-1·46 to -0·92)	-1·14 (-2·42 to 0·14)	No		
		Kantor et al	58	100	-1·14 (-1·38 to -0·91)	-1·22 (-2·5 to 0·06)	No		
		Wilson et al	52	51	-0·9 (-1·1 to -0·71)	-1·24 (-2·52 to 0·04)	No		

		Ettman et al	28	0	-1·9 (-2·26 to -1·54)	-1·33 (-2·62 to -0·04)	No		
		Ettman et al	28	100	-2·06 (-2·4 to -1·72)	-1·42 (-2·71 to -0·13)	No		
		Ettman et al	50	0	-1·41 (-1·8 to -1·02)	-1·21 (-2·5 to 0·07)	No		
		Ettman et al	50	100	-1·57 (-1·95 to -1·2)	-1·3 (-2·59 to -0·02)	No		
		Ettman et al	80	0	-0·76 (-1·19 to -0·32)	-1·05 (-2·34 to 0·25)	No		
		Ettman et al	80	100	-0·92 (-1·34 to -0·5)	-1·14 (-2·43 to 0·16)	No		
		Killgore et al	51	56	-1·56 (-1·73 to -1·4)	-1·28 (-2·57 to 0·01)	No		
		Maxfield et al	50	0	-1·1 (-1·47 to -0·73)	-1·18 (-2·46 to 0·1)	No		
		Maxfield et al	50	100	-1·28 (-1·58 to -0·99)	-1·26 (-2·53 to 0·02)	No		
		Killgore et al	51	55	-1·64 (-1·8 to -1·48)	-1·17 (-2·44 to 0·1)	No		
		Killgore et al	51	54	-1·71 (-1·87 to -1·55)	-1·11 (-2·39 to 0·17)	No		
		Zhou et al	54	52	-0·46 (-0·66 to -0·26)	-1·22 (-2·5 to 0·06)	No		
		Vieira et al	46	0	-0·22 (-0·58 to 0·15)	-1·2 (-2·48 to 0·08)	No		
		Vieira et al	46	0	-0·12 (-0·45 to 0·21)	-1·2 (-2·48 to 0·08)	No		
		Vieira et al	46	100	-0·13 (-0·46 to 0·21)	-1·28 (-2·56 to 0)	No		
		Zhou et al	54	52	-0·17 (-0·4 to 0·06)	-1·16 (-2·44 to 0·11)	No		
		Zhou et al	54	52	-0·1 (-0·36 to 0·17)	-1·13 (-2·4 to 0·14)	No		
	China	Zhang et al	13	41	-0·38 (-0·57 to -0·19)	-0·02 (-1·46 to 1·42)	No	0·68	0·71
		Choi et al	58	55	-0·98 (-1·35 to -0·61)	-0·09 (-1·47 to 1·3)	No		
	Japan	Kikuchi et al	24	0	-0·12 (-0·67 to 0·43)	-0·1 (-1·66 to 1·46)	No	0·46	0·58
		Kikuchi et al	24	100	-0·21 (-0·75 to 0·34)	-0·12 (-1·68 to 1·44)	No		
		Kikuchi et al	34	0	0·06 (-0·46 to 0·59)	-0·09 (-1·64 to 1·47)	No		
		Kikuchi et al	34	100	-0·02 (-0·54 to 0·5)	-0·11 (-1·66 to 1·45)	No		
		Kikuchi et al	44	0	-0·32 (-0·89 to 0·25)	-0·07 (-1·63 to 1·49)	No		
		Kikuchi et al	44	100	-0·41 (-0·97 to 0·16)	-0·09 (-1·65 to 1·47)	No		
		Kikuchi et al	54	0	-0·16 (-0·81 to 0·5)	-0·06 (-1·61 to 1·5)	No		
		Kikuchi et al	54	100	-0·24 (-0·89 to 0·41)	-0·08 (-1·63 to 1·48)	No		
		Kikuchi et al	64	0	-0·56 (-1·34 to 0·22)	-0·04 (-1·6 to 1·51)	No		
		Kikuchi et al	64	100	-0·64 (-1·42 to 0·13)	-0·06 (-1·62 to 1·49)	No		
		Kikuchi et al	74	0	-0·49 (-1·4 to 0·42)	-0·03 (-1·58 to 1·53)	No		

		Kikuchi et al	74	100	-0·57 (-1·48 to 0·34)	-0·05 (-1·6 to 1·51)	No		
		Yamamoto et al	18	0	-1·95 (-2·36 to -1·53)	-1·17 (-2·65 to 0·32)	No		
		Yamamoto et al	18	100	-2·09 (-2·5 to -1·68)	-1·21 (-2·7 to 0·28)	No		
		Yamamoto et al	30	0	-0·95 (-1·1 to -0·8)	-1·13 (-2·62 to 0·35)	No		
		Yamamoto et al	30	100	-1·09 (-1·23 to -0·95)	-1·18 (-2·66 to 0·31)	No		
		Yamamoto et al	52	0	-0·89 (-1·03 to -0·74)	-1·06 (-2·54 to 0·42)	No		
		Yamamoto et al	52	100	-1·02 (-1·16 to -0·89)	-1·1 (-2·59 to 0·38)	No		
		Yamamoto et al	77	0	0·24 (-0·14 to 0·61)	-0·98 (-2·46 to 0·5)	No		
		Yamamoto et al	77	100	0·1 (-0·28 to 0·47)	-1·03 (-2·51 to 0·46)	No		
		Kiuchi et al	21	100	-1·06 (-1·57 to -0·55)	-1·18 (-2·68 to 0·31)	No		
		Kiuchi et al	24	0	-0·36 (-1·04 to 0·33)	-1·13 (-2·63 to 0·36)	No		
		Kiuchi et al	27	100	-0·58 (-1·02 to -0·15)	-1·16 (-2·66 to 0·33)	No		
		Kiuchi et al	32	0	-0·77 (-1·39 to -0·15)	-1·11 (-2·6 to 0·38)	No		
		Kiuchi et al	32	100	-1 (-1·37 to -0·64)	-1·15 (-2·64 to 0·34)	No		
		Kiuchi et al	37	0	-1·09 (-1·69 to -0·49)	-1·09 (-2·58 to 0·4)	No		
		Kiuchi et al	37	100	-1·01 (-1·45 to -0·57)	-1·13 (-2·63 to 0·36)	No		
		Kiuchi et al	42	100	-0·96 (-1·43 to -0·49)	-1·12 (-2·61 to 0·37)	No		
		Kiuchi et al	44	0	-1·02 (-1·54 to -0·49)	-1·07 (-2·56 to 0·42)	No		
		Kiuchi et al	47	100	-0·97 (-1·65 to -0·29)	-1·11 (-2·6 to 0·39)	No		
		Kiuchi et al	74	0	-0·81 (-1·65 to 0·02)	-0·99 (-2·47 to 0·5)	No		
		Kiuchi et al	74	100	-1·04 (-1·7 to -0·38)	-1·02 (-2·51 to 0·47)	No		
		Ueda et al	58	0	-1·23 (-1·67 to -0·79)	-1·03 (-2·52 to 0·45)	No		
		Ueda et al	58	100	-0·75 (-1·08 to -0·41)	-1·07 (-2·56 to 0·42)	No		
		Yamamoto et al	54	52	-0·99 (-1·24 to -0·75)	-1·09 (-2·57 to 0·39)	No		
		Fukase et al	24	0	-1·79 (-2·58 to -1·01)	-1·07 (-2·59 to 0·45)	No		
		Fukase et al	24	100	-0·98 (-1·72 to -0·24)	-1·09 (-2·61 to 0·43)	No		
		Fukase et al	34	0	-1·7 (-2·52 to -0·88)	-1·05 (-2·56 to 0·47)	No		
		Fukase et al	34	100	-0·89 (-1·67 to -0·12)	-1·07 (-2·59 to 0·45)	No		
		Fukase et al	44	0	-1·96 (-2·8 to -1·13)	-1·03 (-2·54 to 0·49)	No		
		Fukase et al	44	100	-1·15 (-1·95 to -0·36)	-1·05 (-2·57 to 0·47)	No		

		Fukase et al	54	0	-1·25 (-1·94 to -0·55)	-1·01 (-2·52 to 0·51)	No		
		Fukase et al	54	100	-0·44 (-1·08 to 0·21)	-1·03 (-2·55 to 0·49)	No		
		Fukase et al	64	0	-1·28 (-2·25 to -0·3)	-0·99 (-2·5 to 0·53)	No		
		Fukase et al	64	100	-0·47 (-1·4 to 0·47)	-1·01 (-2·53 to 0·51)	No		
	Czechia	Winkler et al	58	54	-1·17 (-1·39 to -0·96)	-1·02 (-2·36 to 0·33)	No	0·16	0·06
	UK	Shevlin et al	50	52	-0·97 (-1·22 to -0·72)	-1·21 (-2·51 to 0·09)	No	0·72	0·53
		O'Connor et al	58	51	-1·2 (-1·44 to -0·95)	-1·03 (-2·49 to 0·42)	No		
		Groarke et al	52	65	-1·52 (-1·77 to -1·26)	-1·23 (-2·61 to 0·14)	No		
		O'Connor et al	58	51	-1·1 (-1·35 to -0·86)	-1·03 (-2·48 to 0·42)	No		
		Pieh et al (c)	26	0	-2·66 (-3·46 to -1·86)	-2·08 (-3·5 to -0·65)	No		
		Pieh et al (c)	26	100	-2·63 (-3·37 to -1·9)	-2·18 (-3·6 to -0·76)	No		
		Pieh et al (c)	50	0	-2·25 (-2·89 to -1·61)	-1·27 (-2·67 to 0·13)	No		
		Pieh et al (c)	50	100	-2·23 (-2·79 to -1·66)	-1·38 (-2·77 to 0·02)	No		
		Pieh et al (c)	82	0	-0·91 (-1·77 to -0·06)	-0·16 (-1·6 to 1·28)	No		
		Pieh et al (c)	82	100	-0·89 (-1·69 to -0·09)	-0·27 (-1·69 to 1·16)	No		
		O'Connor et al	58	51	-1·07 (-1·31 to -0·82)	-1·01 (-2·35 to 0·33)	No		
		Widnall et al	14	0	0·13 (-0·21 to 0·46)	-1·56 (-3·18 to 0·07)	Yes		
		Widnall et al	14	100	-0·14 (-0·37 to 0·1)	-1·66 (-3·28 to -0·04)	No		
		Vizard et al	28	0	-1·29 (-2·1 to -0·48)	-0·7 (-2·13 to 0·72)	No		
		Vizard et al	28	100	-1·32 (-2·04 to -0·6)	-0·77 (-2·19 to 0·65)	No		
		Vizard et al	54	0	-0·24 (-0·93 to 0·44)	-0·13 (-1·55 to 1·29)	No		
		Vizard et al	54	100	-0·27 (-0·84 to 0·3)	-0·19 (-1·61 to 1·22)	No		
		Vizard et al	84	0	-0·77 (-1·6 to 0·06)	0·52 (-0·92 to 1·95)	No		
		Vizard et al	84	100	-0·8 (-1·54 to -0·05)	0·45 (-0·98 to 1·88)	No		
		Katz et al	48	0	-0·32 (-1·43 to 0·79)	-0·45 (-2·09 to 1·2)	No		
		Katz et al	48	0	-0·46 (-1·55 to 0·63)	-0·45 (-2·09 to 1·2)	No		
		Katz et al	48	100	-0·18 (-1·03 to 0·66)	-0·56 (-2·19 to 1·08)	No		
		Katz et al	48	100	-0·18 (-1 to 0·65)	-0·56 (-2·19 to 1·08)	No		
		Kwong et al	28	73	0·34 (0·19 to 0·49)	-1·23 (-2·87 to 0·41)	No		
		Daly et al (a)	26	0	-0·71 (-0·85 to -0·57)	-1·22 (-2·88 to 0·43)	No		

		Daly et al (a)	26	100	-0·88 (-1·02 to -0·75)	-1·33 (-2·98 to 0·31)	No		
		Daly et al (a)	42	0	-0·55 (-0·69 to -0·42)	-0·66 (-2·29 to 0·98)	No		
		Daly et al (a)	42	100	-0·73 (-0·86 to -0·6)	-0·77 (-2·4 to 0·86)	No		
		Daly et al (a)	57	0	-0·34 (-0·48 to -0·21)	-0·12 (-1·76 to 1·52)	No		
		Daly et al (a)	57	100	-0·52 (-0·65 to -0·39)	-0·23 (-1·86 to 1·39)	No		
		Daly et al (a)	82	0	-0·66 (-0·81 to -0·51)	0·76 (-0·91 to 2·44)	No		
		Daly et al (a)	82	100	-0·83 (-0·98 to -0·69)	0·65 (-1·01 to 2·32)	No		
		Daly et al (a)	26	0	-0·54 (-0·68 to -0·4)	-1 (-2·53 to 0·53)	No		
		Daly et al (a)	26	100	-0·62 (-0·75 to -0·49)	-1·09 (-2·62 to 0·43)	No		
		Daly et al (a)	42	0	-0·48 (-0·62 to -0·34)	-0·54 (-2·05 to 0·98)	No		
		Daly et al (a)	42	100	-0·56 (-0·69 to -0·43)	-0·63 (-2·14 to 0·88)	No		
		Daly et al (a)	57	0	-0·32 (-0·46 to -0·19)	-0·1 (-1·62 to 1·42)	No		
		Daly et al (a)	57	100	-0·4 (-0·53 to -0·28)	-0·19 (-1·7 to 1·32)	No		
		Daly et al (a)	82	0	-0·53 (-0·69 to -0·38)	0·63 (-0·92 to 2·17)	No		
		Daly et al (a)	82	100	-0·61 (-0·76 to -0·47)	0·54 (-1 to 2·07)	No		
		Daly et al (a)	26	0	-0·41 (-0·55 to -0·27)	-0·72 (-2·15 to 0·7)	No		
		Daly et al (a)	26	100	-0·43 (-0·56 to -0·29)	-0·79 (-2·21 to 0·63)	No		
		Daly et al (a)	42	0	-0·39 (-0·53 to -0·25)	-0·39 (-1·8 to 1·03)	No		
		Daly et al (a)	42	100	-0·4 (-0·54 to -0·27)	-0·45 (-1·87 to 0·96)	No		
		Daly et al (a)	57	0	-0·3 (-0·43 to -0·16)	-0·07 (-1·49 to 1·35)	No		
		Daly et al (a)	57	100	-0·31 (-0·44 to -0·18)	-0·14 (-1·55 to 1·28)	No		
		Daly et al (a)	82	0	-0·43 (-0·58 to -0·28)	0·46 (-0·98 to 1·89)	No		
		Daly et al (a)	82	100	-0·44 (-0·59 to -0·3)	0·39 (-1·04 to 1·82)	No		
	Norway	Knudsen et al	42	62	0·47 (-0·35 to 1·3)	-0·2 (-1·59 to 1·2)	No	0·68	0·78
		Knudsen et al	42	62	0·75 (-0·25 to 1·74)	-0·11 (-1·53 to 1·32)	No		
		Knudsen et al	42	62	-0·55 (-1·3 to 0·2)	-0·1 (-1·53 to 1·32)	No		
	Australia	ABS (2020)	26	0	-1·24 (-1·73 to -0·75)	-0·1 (-1·56 to 1·36)	No	0·51	0·51
		ABS (2020)	50	0	-0·16 (-0·51 to 0·19)	-0·06 (-1·52 to 1·4)	No		
		ABS (2020)	50	100	-0·48 (-0·77 to -0·19)	-0·08 (-1·54 to 1·38)	No		
		ABS (2020)	82	0	0·29 (-0·15 to 0·72)	-0·02 (-1·47 to 1·44)	No		

		ABS (2020)	82	100	-0·04 (-0·43 to 0·36)	-0·03 (-1·49 to 1·42)	No		
		Biddle et al	58	50	-0·26 (-0·43 to -0·09)	-0·11 (-1·55 to 1·32)	No		
	New Zealand	Sibley et al	58	65	0·14 (-0·23 to 0·5)	-0·11 (-1·52 to 1·31)	No	0·57	0·56
		Every-Palmer et al	21	51	-1·97 (-2·34 to -1·61)	-1·23 (-2·54 to 0·07)	No		
		Every-Palmer et al	30	51	-2·09 (-2·39 to -1·79)	-1·2 (-2·5 to 0·11)	No		
		Every-Palmer et al	40	51	-2·31 (-2·65 to -1·98)	-1·15 (-2·45 to 0·15)	No		
		Every-Palmer et al	50	51	-1·47 (-1·81 to -1·13)	-1·11 (-2·41 to 0·19)	No		
		Every-Palmer et al	60	51	-0·93 (-1·34 to -0·51)	-1·06 (-2·36 to 0·24)	No		
		Every-Palmer et al	70	51	-1 (-1·56 to -0·45)	-1·02 (-2·32 to 0·28)	No		
		Every-Palmer et al	82	51	-0·83 (-1·7 to 0·04)	-0·96 (-2·26 to 0·34)	No		
		Bulbulia et al	58	65	0·04 (-0·34 to 0·42)	-0·11 (-1·53 to 1·31)	No		
	Austria	Pieh et al (b)	54	0	-1·52 (-2·1 to -0·95)	-1·07 (-2·37 to 0·24)	No	0·31	0·21
		Pieh et al (b)	54	100	-1·5 (-2·04 to -0·97)	-1·13 (-2·43 to 0·17)	No		
		Pieh et al (b)	82	0	-1·11 (-1·99 to -0·23)	-0·94 (-2·24 to 0·37)	No		
		Pieh et al (b)	82	100	-1·09 (-1·94 to -0·23)	-1 (-2·3 to 0·31)	No		
	Denmark	Sønderskov et al (b)	58	0	-0·11 (-0·29 to 0·07)	-1·16 (-2·39 to 0·08)	No	1·16	1·21
		Sønderskov et al (b)	58	100	-0·21 (-0·38 to -0·05)	-1·24 (-2·47 to 0)	No		
		Sønderskov et al (a)	58	0	0·16 (-0·04 to 0·36)	-1·12 (-2·35 to 0·11)	Yes		
		Sønderskov et al (a)	58	100	0·09 (-0·09 to 0·27)	-1·17 (-2·4 to 0·06)	Yes		
	France	Peretti-Watel et al	58	52	-1·37 (-1·49 to -1·25)	-1·18 (-2·52 to 0·17)	No	0·2	0·34
	Germany	Peters et al	47	52	-0·34 (-0·38 to -0·31)	-0·17 (-1·54 to 1·21)	No	0·18	0·24
	Ireland	Daly et al (b)	26	0	-1·72 (-2·1 to -1·34)	-1·38 (-2·71 to -0·06)	No	0·65	0·69
		Daly et al (b)	26	100	-1·9 (-2·26 to -1·53)	-1·47 (-2·79 to -0·15)	No		
		Daly et al (b)	44	0	-1·05 (-1·45 to -0·65)	-1·27 (-2·59 to 0·05)	No		
		Daly et al (b)	44	100	-1·22 (-1·6 to -0·84)	-1·36 (-2·68 to -0·05)	No		
		Daly et al (b)	77	0	-0·08 (-0·69 to 0·54)	-1·08 (-2·41 to 0·25)	No		
		Daly et al (b)	77	100	-0·25 (-0·85 to 0·35)	-1·17 (-2·5 to 0·15)	No		
		Daly et al (b)	26	0	-1·28 (-1·69 to -0·87)	-1·35 (-2·67 to -0·03)	No		
		Daly et al (b)	26	100	-1·5 (-1·88 to -1·13)	-1·44 (-2·75 to -0·13)	No		
		Daly et al (b)	44	0	-0·94 (-1·37 to -0·51)	-1·25 (-2·56 to 0·07)	No		

		Daly et al (b)	44	100	-1·16 (-1·56 to -0·77)	-1·34 (-2·65 to -0·03)	No		
		Daly et al (b)	77	0	0·21 (-0·32 to 0·75)	-1·07 (-2·39 to 0·25)	No		
		Daly et al (b)	77	100	-0·01 (-0·52 to 0·5)	-1·16 (-2·47 to 0·16)	No		
		Daly et al (b)	26	0	-1·72 (-2·1 to -1·35)	-1·18 (-2·49 to 0·13)	No		
		Daly et al (b)	26	100	-1·76 (-2·12 to -1·4)	-1·24 (-2·54 to 0·07)	No		
		Daly et al (b)	44	0	-1·2 (-1·59 to -0·81)	-1·11 (-2·42 to 0·19)	No		
		Daly et al (b)	44	100	-1·24 (-1·62 to -0·87)	-1·17 (-2·48 to 0·14)	No		
		Daly et al (b)	77	0	-0·1 (-0·58 to 0·38)	-1 (-2·31 to 0·31)	No		
		Daly et al (b)	77	100	-0·14 (-0·61 to 0·33)	-1·05 (-2·36 to 0·26)	No		
	Netherlands	Van der Velden et al	26	0	0·14 (-0·16 to 0·44)	-0·24 (-1·67 to 1·19)	No	0·25	0·32
		Van der Velden et al	26	100	0·26 (-0·03 to 0·54)	-0·29 (-1·72 to 1·14)	No		
		Van der Velden et al	42	0	-0·24 (-0·55 to 0·07)	-0·18 (-1·61 to 1·25)	No		
		Van der Velden et al	42	100	-0·12 (-0·42 to 0·18)	-0·23 (-1·66 to 1·2)	No		
		Van der Velden et al	57	0	-0·29 (-0·61 to 0·04)	-0·12 (-1·56 to 1·31)	No		
		Van der Velden et al	57	100	-0·17 (-0·48 to 0·14)	-0·17 (-1·61 to 1·26)	No		
		Van der Velden et al	82	0	-0·03 (-0·39 to 0·32)	-0·03 (-1·47 to 1·41)	No		
		Van der Velden et al	82	100	0·08 (-0·26 to 0·43)	-0·08 (-1·52 to 1·35)	No		
	Spain	Ayuso-Mateos et al	34	0	-1·47 (-3·45 to 0·52)	-0·24 (-1·62 to 1·13)	No	0·75	0·86
		Ayuso-Mateos et al	34	100	-1·26 (-3·21 to 0·69)	-0·31 (-1·68 to 1·07)	No		
		Ayuso-Mateos et al	74	0	-0·55 (-2·5 to 1·41)	-0·09 (-1·47 to 1·28)	No		
		Ayuso-Mateos et al	74	100	-0·35 (-2·27 to 1·57)	-0·16 (-1·54 to 1·22)	No		
		Valiente et al	30	0	-2·49 (-2·82 to -2·16)	-1·42 (-2·83 to -0·01)	No		
		Valiente et al	30	100	-2·49 (-2·8 to -2·17)	-1·55 (-2·97 to -0·14)	No		
		Valiente et al	40	0	-2·15 (-2·45 to -1·85)	-1·35 (-2·76 to 0·05)	No		
		Valiente et al	40	100	-2·15 (-2·44 to -1·87)	-1·48 (-2·89 to -0·07)	No		
		Valiente et al	50	0	-1·07 (-1·37 to -0·76)	-1·28 (-2·68 to 0·13)	No		
		Valiente et al	50	100	-1·07 (-1·36 to -0·78)	-1·41 (-2·82 to 0)	No		
		Valiente et al	60	0	-0·73 (-1·05 to -0·42)	-1·21 (-2·61 to 0·2)	No		
		Valiente et al	60	100	-0·74 (-1·03 to -0·44)	-1·34 (-2·75 to 0·07)	No		
		Valiente et al	70	0	-0·38 (-0·96 to 0·2)	-1·13 (-2·54 to 0·28)	No		

		Valiente et al	70	100	-0·38 (-0·95 to 0·19)	-1·26 (-2·68 to 0·15)	No		
Anxiety disorders	USA	Marroquin et al	46	50	-0·21 (-0·85 to 0·43)	-0·27 (-1·45 to 0·9)	No	0·78	0·76
		Kantor et al	58	0	-1·86 (-2·1 to -1·63)	-0·85 (-2·39 to 0·69)	No		
		Kantor et al	58	100	-1·6 (-1·8 to -1·4)	-0·94 (-2·48 to 0·6)	No		
		Katz et al	46	0	0·21 (-1·05 to 1·47)	-0·29 (-1·48 to 0·9)	No		
		Katz et al	46	0	0·27 (-0·76 to 1·3)	-0·5 (-1·72 to 0·72)	No		
		Katz et al	46	100	0·45 (-0·88 to 1·78)	-0·39 (-1·57 to 0·79)	No		
		Katz et al	46	100	0·22 (-0·7 to 1·14)	-0·6 (-1·81 to 0·61)	No		
		McGinty et al (a)	24	0	-2·24 (-2·92 to -1·55)	-1·09 (-2·66 to 0·49)	No		
		McGinty et al (a)	24	100	-2·04 (-2·7 to -1·37)	-1·18 (-2·75 to 0·4)	No		
		McGinty et al (a)	42	0	-1·5 (-2·05 to -0·94)	-1·06 (-2·62 to 0·51)	No		
		McGinty et al (a)	42	100	-1·3 (-1·83 to -0·77)	-1·14 (-2·71 to 0·42)	No		
		McGinty et al (a)	77	0	-0·82 (-1·45 to -0·19)	-1 (-2·56 to 0·57)	No		
		McGinty et al (a)	77	100	-0·62 (-1·23 to -0·01)	-1·08 (-2·65 to 0·48)	No		
		Twenge et al	52	0	-1·87 (-2·2 to -1·54)	-1·11 (-2·69 to 0·48)	No		
		Twenge et al	52	100	-2·49 (-2·82 to -2·16)	-1·21 (-2·79 to 0·38)	No		
		Twenge et al	80	0	-1·3 (-1·71 to -0·88)	-1·05 (-2·63 to 0·53)	No		
		Twenge et al	80	100	-1·92 (-2·34 to -1·5)	-1·15 (-2·74 to 0·43)	No		
		McGinty et al (b)	24	0	-2·48 (-3·33 to -1·63)	-0·98 (-2·53 to 0·57)	No		
		McGinty et al (b)	24	100	-2·07 (-2·9 to -1·24)	-1·05 (-2·6 to 0·5)	No		
		McGinty et al (b)	42	0	-1·39 (-2 to -0·77)	-0·96 (-2·5 to 0·59)	No		
		McGinty et al (b)	42	100	-0·98 (-1·56 to -0·39)	-1·03 (-2·57 to 0·52)	No		
		McGinty et al (b)	77	0	-0·87 (-1·59 to -0·15)	-0·91 (-2·45 to 0·63)	No		
		McGinty et al (b)	77	100	-0·46 (-1·15 to 0·23)	-0·98 (-2·52 to 0·57)	No		
		Zhou et al	54	52	-0·19 (-0·38 to -0·01)	-0·93 (-2·47 to 0·61)	No		
		Vieira et al	46	0	-0·12 (-0·45 to 0·21)	-1·07 (-2·64 to 0·5)	No		
		Zhou et al	54	52	0·06 (-0·16 to 0·28)	-0·87 (-2·41 to 0·66)	No		
		Zhou et al	54	52	0·25 (0 to 0·5)	-0·84 (-2·37 to 0·69)	No		
	China	Zhang et al	13	41	-0·19 (-0·41 to 0·03)	-0·07 (-1·61 to 1·47)	No	0·71	1·06
		Choi et al	58	55	-1·13 (-1·68 to -0·58)	-0·13 (-1·67 to 1·41)	No		

	Japan	Kikuchi et al	24	0	-0·12 (-0·67 to 0·43)	-0·13 (-1·84 to 1·58)	No	0·44	0·65
		Kikuchi et al	24	100	-0·21 (-0·75 to 0·34)	-0·15 (-1·86 to 1·55)	No		
		Kikuchi et al	34	0	0·06 (-0·46 to 0·59)	-0·13 (-1·83 to 1·58)	No		
		Kikuchi et al	34	100	-0·02 (-0·54 to 0·5)	-0·15 (-1·86 to 1·56)	No		
		Kikuchi et al	44	0	-0·32 (-0·89 to 0·25)	-0·12 (-1·83 to 1·59)	No		
		Kikuchi et al	44	100	-0·41 (-0·97 to 0·16)	-0·14 (-1·85 to 1·56)	No		
		Kikuchi et al	54	0	-0·16 (-0·81 to 0·5)	-0·12 (-1·83 to 1·59)	No		
		Kikuchi et al	54	100	-0·24 (-0·89 to 0·41)	-0·14 (-1·85 to 1·57)	No		
		Kikuchi et al	64	0	-0·56 (-1·34 to 0·22)	-0·11 (-1·82 to 1·6)	No		
		Kikuchi et al	64	100	-0·64 (-1·42 to 0·13)	-0·14 (-1·84 to 1·57)	No		
		Kikuchi et al	74	0	-0·49 (-1·4 to 0·42)	-0·11 (-1·82 to 1·61)	No		
		Kikuchi et al	74	100	-0·57 (-1·48 to 0·34)	-0·13 (-1·84 to 1·58)	No		
		Yamamoto et al	18	0	-1·95 (-2·36 to -1·53)	-0·94 (-2·91 to 1·04)	No		
		Yamamoto et al	18	100	-2·09 (-2·5 to -1·68)	-0·99 (-2·97 to 0·99)	No		
		Yamamoto et al	30	0	-0·95 (-1·1 to -0·8)	-0·92 (-2·9 to 1·05)	No		
		Yamamoto et al	30	100	-1·09 (-1·23 to -0·95)	-0·98 (-2·95 to 1)	No		
		Yamamoto et al	52	0	-0·89 (-1·03 to -0·74)	-0·9 (-2·87 to 1·07)	No		
		Yamamoto et al	52	100	-1·02 (-1·16 to -0·89)	-0·95 (-2·93 to 1·02)	No		
		Yamamoto et al	77	0	0·24 (-0·14 to 0·61)	-0·88 (-2·85 to 1·09)	No		
		Yamamoto et al	77	100	0·1 (-0·28 to 0·47)	-0·93 (-2·9 to 1·04)	No		
		Kiuchi et al	21	100	-1·06 (-1·57 to -0·55)	-0·96 (-2·94 to 1·01)	No		
		Kiuchi et al	24	0	-0·36 (-1·04 to 0·33)	-0·91 (-2·88 to 1·06)	No		
		Kiuchi et al	27	100	-0·58 (-1·02 to -0·15)	-0·96 (-2·93 to 1·02)	No		
		Kiuchi et al	32	0	-0·77 (-1·39 to -0·15)	-0·9 (-2·87 to 1·07)	No		
		Kiuchi et al	32	100	-1 (-1·37 to -0·64)	-0·95 (-2·92 to 1·02)	No		
		Kiuchi et al	37	0	-1·09 (-1·69 to -0·49)	-0·9 (-2·87 to 1·07)	No		
		Kiuchi et al	37	100	-1·01 (-1·45 to -0·57)	-0·95 (-2·92 to 1·03)	No		
		Kiuchi et al	42	100	-0·96 (-1·43 to -0·49)	-0·94 (-2·91 to 1·03)	No		
		Kiuchi et al	44	0	-1·02 (-1·54 to -0·49)	-0·89 (-2·86 to 1·08)	No		
		Kiuchi et al	47	100	-0·97 (-1·65 to -0·29)	-0·94 (-2·91 to 1·03)	No		

		Kiuchi et al	74	0	-0·81 (-1·65 to 0·02)	-0·86 (-2·83 to 1·1)	No		
		Kiuchi et al	74	100	-1·04 (-1·7 to -0·38)	-0·91 (-2·88 to 1·06)	No		
	Czechia	Winkler et al	58	54	-0·56 (-0·72 to -0·39)	-0·81 (-2·61 to 0·99)	No	0·25	0·45
	UK	Widnall et al	14	0	0·47 (0·14 to 0·8)	-1·66 (-4·16 to 0·84)	No	0·85	0·37
		Widnall et al	14	100	0 (-0·22 to 0·22)	-1·77 (-4·29 to 0·75)	No		
		Katz et al	48	0	0 (-2·01 to 2·01)	-0·81 (-3·39 to 1·78)	No		
		Katz et al	48	0	-0·46 (-1·55 to 0·63)	-1·2 (-3·6 to 1·19)	No		
		Katz et al	48	100	0·29 (-0·77 to 1·35)	-0·92 (-3·52 to 1·68)	No		
		Katz et al	48	100	-0·18 (-1 to 0·65)	-1·32 (-3·72 to 1·08)	No		
		Daly et al (a)	26	0	-0·71 (-0·85 to -0·57)	-1·82 (-4·24 to 0·61)	No		
		Daly et al (a)	26	100	-0·88 (-1·02 to -0·75)	-1·93 (-4·36 to 0·5)	No		
		Daly et al (a)	42	0	-0·55 (-0·69 to -0·42)	-1·36 (-3·74 to 1·02)	No		
		Daly et al (a)	42	100	-0·73 (-0·86 to -0·6)	-1·48 (-3·86 to 0·91)	No		
		Daly et al (a)	57	0	-0·34 (-0·48 to -0·21)	-0·94 (-3·31 to 1·44)	No		
		Daly et al (a)	57	100	-0·52 (-0·65 to -0·39)	-1·05 (-3·43 to 1·33)	No		
		Daly et al (a)	82	0	-0·66 (-0·81 to -0·51)	-0·23 (-2·68 to 2·23)	No		
		Daly et al (a)	82	100	-0·83 (-0·98 to -0·69)	-0·34 (-2·79 to 2·1)	No		
		Daly et al (a)	26	0	-0·54 (-0·68 to -0·4)	-1·52 (-3·62 to 0·58)	No		
		Daly et al (a)	26	100	-0·62 (-0·75 to -0·49)	-1·61 (-3·72 to 0·49)	No		
		Daly et al (a)	42	0	-0·48 (-0·62 to -0·34)	-1·14 (-3·2 to 0·93)	No		
		Daly et al (a)	42	100	-0·56 (-0·69 to -0·43)	-1·23 (-3·3 to 0·84)	No		
		Daly et al (a)	57	0	-0·32 (-0·46 to -0·19)	-0·78 (-2·85 to 1·28)	No		
		Daly et al (a)	57	100	-0·4 (-0·53 to -0·28)	-0·88 (-2·94 to 1·19)	No		
		Daly et al (a)	82	0	-0·53 (-0·69 to -0·38)	-0·19 (-2·32 to 1·94)	No		
		Daly et al (a)	82	100	-0·61 (-0·76 to -0·47)	-0·29 (-2·41 to 1·84)	No		
		Daly et al (a)	26	0	-0·41 (-0·55 to -0·27)	-1·12 (-2·84 to 0·6)	No		
		Daly et al (a)	26	100	-0·43 (-0·56 to -0·29)	-1·19 (-2·92 to 0·54)	No		
		Daly et al (a)	42	0	-0·39 (-0·53 to -0·25)	-0·84 (-2·54 to 0·86)	No		
		Daly et al (a)	42	100	-0·4 (-0·54 to -0·27)	-0·91 (-2·61 to 0·79)	No		
		Daly et al (a)	57	0	-0·3 (-0·43 to -0·16)	-0·58 (-2·28 to 1·12)	No		

		Daly et al (a)	57	100	-0·31 (-0·44 to -0·18)	-0·65 (-2·35 to 1·05)	No		
		Daly et al (a)	82	0	-0·43 (-0·58 to -0·28)	-0·14 (-1·89 to 1·6)	No		
		Daly et al (a)	82	100	-0·44 (-0·59 to -0·3)	-0·21 (-1·96 to 1·53)	No		
		Kwong et al	28	73	-0·71 (-0·89 to -0·53)	-1·45 (-4·03 to 1·14)	No		
	Norway	Knudsen et al	42	62	0·58 (0·15 to 1·01)	-0·2 (-1·75 to 1·35)	No	0·56	0·63
		Knudsen et al	42	62	0·26 (-2·06 to 2·59)	-0·11 (-1·66 to 1·44)	No		
		Knudsen et al	42	62	0·32 (-0·17 to 0·8)	-0·11 (-1·66 to 1·44)	No		
	Australia	ABS (2020)	26	0	-1·24 (-1·73 to -0·75)	-0·14 (-1·74 to 1·47)	No	0·5	0·48
		ABS (2020)	50	0	-0·16 (-0·51 to 0·19)	-0·13 (-1·73 to 1·48)	No		
		ABS (2020)	50	100	-0·48 (-0·77 to -0·19)	-0·15 (-1·76 to 1·46)	No		
		ABS (2020)	82	0	0·29 (-0·15 to 0·72)	-0·11 (-1·71 to 1·5)	No		
		ABS (2020)	82	100	-0·04 (-0·43 to 0·36)	-0·13 (-1·74 to 1·48)	No		
		Biddle et al	58	50	-0·26 (-0·43 to -0·09)	-0·25 (-1·86 to 1·36)	No		
	New Zealand	Sibley et al	58	65	0·14 (-0·23 to 0·5)	-0·22 (-1·64 to 1·21)	No	0·7	0·6
		Every-Palmer et al	21	51	-1·97 (-2·34 to -1·61)	-1 (-2·62 to 0·61)	No		
		Every-Palmer et al	30	51	-2·09 (-2·39 to -1·79)	-0·99 (-2·6 to 0·62)	No		
		Every-Palmer et al	40	51	-2·31 (-2·65 to -1·98)	-0·97 (-2·59 to 0·64)	No		
		Every-Palmer et al	50	51	-1·47 (-1·81 to -1·13)	-0·96 (-2·57 to 0·66)	No		
		Every-Palmer et al	60	51	-0·93 (-1·34 to -0·51)	-0·94 (-2·56 to 0·68)	No		
		Every-Palmer et al	70	51	-1 (-1·56 to -0·45)	-0·92 (-2·55 to 0·7)	No		
		Every-Palmer et al	82	51	-0·83 (-1·7 to 0·04)	-0·9 (-2·53 to 0·73)	No		
		Bulbulia et al	58	65	0·04 (-0·34 to 0·42)	-0·22 (-1·64 to 1·21)	No		
	France	Sante Publique France	58	50	-0·85 (-0·95 to -0·74)	-1·13 (-3·11 to 0·85)	No	0·6	0·62
		Sante Publique France	58	50	-0·56 (-0·67 to -0·45)	-1·16 (-3·21 to 0·89)	No		
		Sante Publique France	58	50	-0·35 (-0·47 to -0·23)	-1·09 (-2·96 to 0·78)	No		
		Sante Publique France	58	50	-0·4 (-0·52 to -0·28)	-1·06 (-2·87 to 0·74)	No		
		Sante Publique France	58	50	-0·35 (-0·47 to -0·23)	-1·04 (-2·78 to 0·7)	No		
		Sante Publique France	58	50	-0·37 (-0·49 to -0·25)	-1·02 (-2·72 to 0·68)	No		
		Sante Publique France	58	50	-0·31 (-0·43 to -0·19)	-0·96 (-2·54 to 0·62)	No		
		Sante Publique France	58	50	-0·26 (-0·39 to -0·14)	-0·92 (-2·44 to 0·59)	No		

		Sante Publique France	58	50	-0·24 (-0·36 to -0·11)	-0·89 (-2·36 to 0·58)	No		
		Sante Publique France	58	50	-0·19 (-0·32 to -0·07)	-0·85 (-2·27 to 0·58)	No		
		Sante Publique France	58	50	-0·13 (-0·26 to 0)	-0·82 (-2·22 to 0·58)	No		
		Sante Publique France	58	50	-0·27 (-0·39 to -0·15)	-0·79 (-2·18 to 0·6)	No		
		Sante Publique France	58	50	-0·31 (-0·43 to -0·19)	-0·81 (-2·21 to 0·59)	No		
		Sante Publique France	58	50	-0·31 (-0·43 to -0·19)	-0·87 (-2·32 to 0·58)	No		
		Sante Publique France	58	50	-0·34 (-0·46 to -0·22)	-0·87 (-2·32 to 0·58)	No		
		Sante Publique France	58	50	-0·41 (-0·53 to -0·3)	-0·97 (-2·57 to 0·63)	No		
		Sante Publique France	58	50	-0·52 (-0·63 to -0·41)	-1·05 (-2·81 to 0·72)	No		
		Sante Publique France	58	50	-0·37 (-0·49 to -0·26)	-1 (-2·65 to 0·66)	No		
		Sante Publique France	58	50	-0·48 (-0·6 to -0·37)	-0·94 (-2·49 to 0·61)	No		
		Sante Publique France	58	50	-0·42 (-0·54 to -0·3)	-0·98 (-2·6 to 0·64)	No		
	Germany	Ravens-Sieberer et al	14	50	-0·6 (-0·8 to -0·39)	-0·23 (-1·84 to 1·37)	No	0·27	0·64
		Peters et al	47	52	-0·3 (-0·33 to -0·26)	-0·18 (-1·79 to 1·42)	No		
	Netherlands	Van der Velden et al	26	0	0·14 (-0·16 to 0·44)	-0·29 (-1·89 to 1·31)	No	0·31	0·47
		Van der Velden et al	26	100	0·26 (-0·03 to 0·54)	-0·35 (-1·94 to 1·25)	No		
		Van der Velden et al	42	0	-0·24 (-0·55 to 0·07)	-0·27 (-1·87 to 1·33)	No		
		Van der Velden et al	42	100	-0·12 (-0·42 to 0·18)	-0·33 (-1·92 to 1·27)	No		
		Van der Velden et al	57	0	-0·29 (-0·61 to 0·04)	-0·25 (-1·85 to 1·35)	No		
		Van der Velden et al	57	100	-0·17 (-0·48 to 0·14)	-0·31 (-1·9 to 1·29)	No		
		Van der Velden et al	82	0	-0·03 (-0·39 to 0·32)	-0·22 (-1·82 to 1·39)	No		
		Van der Velden et al	82	100	0·08 (-0·26 to 0·43)	-0·27 (-1·87 to 1·33)	No		

MDD = Major depressive disorder, USA = United States of America, UK = United Kingdom, CI = Confidence interval, PI = Prediction interval, LOCOCV = Leave-one-country-out cross-validation, RMSE = Root-mean-square error

Section 9. Authors' contributions

Damian F Santomauro was responsible for the design of the study, development of the methods, and statistical analyses. Ana M Mantilla Herrera and Jamileh Shadid searched for data sources and did the title and abstract and full text screening of identified studies. Damian F Santomauro crosschecked all inclusions, reasons for exclusion, and extracted the data. Damian F Santomauro, Alize J Ferrari, Ana M Mantilla Herrera, and Jamileh Shadid wrote the first draft of the manuscript. Damian F Santomauro, Alize J Ferrari, and Harvey A Whiteford critically revised the manuscript for important intellectual content. All authors contributed to writing the final manuscript, critically reviewed the methods and results, and approved the final version of the manuscript. More information about individual author contributions to the research are below.

Section 9.1. Managing the estimation or publications process
Damian F Santomauro, Charlie Ashbaugh, Joanne Amlag, Sabina Bloom, William J Dangel, Amanda Deen, Maja Pasovic, Simon I Hay, and Alize J Ferrari.

Section 9.2. Writing the first draft of the manuscript
Damian F Santomauro, Ana M Mantilla Herrera, Jamileh Shadid, Harvey A Whiteford, and Alize J Ferrari.

Section 9.3. Primary responsibility for applying analytical methods to produce estimates
Damian F Santomauro.

Section 9.4. Primary responsibility for seeking, cataloguing, extracting, or cleaning data; designing or coding figures and tables
Damian F Santomauro, Ana M Mantilla Herrera, and Jamileh Shadid (for data inputs related to mental health indicators).

Damian F Santomauro, Ana M Mantilla Herrera, Jhilik Chattopadhyay, Rebecca M Cogen, Samuel B Ewald, Gaorui Guo, Monika Helak, Erin N Hulland, Bulat Idrisov, Akiaja Lindstrom, Emily Linebarger, Ali H Mokdad, Paulami Naik, Shuhei Nomura, James Kevin O'Halloran, Louise Penberthy, Grace Reinke, Anh Truc Vo, Stefanie Watson, and Bethany Zigler (for data inputs related to COVID-19 indicators).

Section 9.5. Providing data or critical feedback on data sources
Damian F Santomauro, Ana M Mantilla Herrera, Jamileh Shadid, Charlie Ashbaugh, Alize Ferrari, Harvey A Whiteford (for data inputs related to mental health indicators).

Damian F Santomauro, Ana M Mantilla Herrera, David Pigott, Cristiana Abbafati, Christopher Adolph, Bree L Bang-Jensen, Gregory J Bertolacci, Rachel Castellano, Suman Chakrabarti, Carolyn Dapper, Megan Erickson, Abraham D Flaxman, Nancy Fullman, Ababi Zergaw Giref, Gaorui Guo, Erin N Hulland, Bulat Idrisov, Emily Linebarger, Paulo A Lotufo, Beatrice Magistro, Deborah Carvalho Malta, Johan Månsson, Fatima Marinho, Ali H Mokdad, Lorenzo Monasta, Shuhei Nomura, Maja Pasovic, Robert C Reiner Jr, Antonio Luiz P Ribeiro, Elena Varavikova, Anh Truc Vo, Rebecca Walcott, Stefanie Watson, Charles Shey Wiysonge, Simon I Hay, Theo Vos, Christopher J L Murray, and Alize J Ferrari (for data inputs related to COVID-19 indicators).

Section 9.5. Developing methods or computational machinery
Damian F Santomauro, Ana M Mantilla Herrera, Jamileh Shadid, Charlie Ashbaugh, David Pigott, Peng Zheng, Joanne O Amlag, Emma Castro, James K Collins, Xiaochen Dai, Abraham D Flaxman, Joseph Jon Frostad, John R Giles, Jiawei He, Ali H Mokdad, Robert C Reiner Jr, Aleksei Sholokhov, Reed J D Sorensen, Theo Vos, Christopher J L Murray, Harvey A Whiteford, and Alize J Ferrari.

Section 9.6. Providing critical feedback on methods or results
Damian F Santomauro, Ana M Mantilla Herrera, Jamileh Shadid, Charlie Ashbaugh, David Pigott, Peng Zheng, Ali H Mokdad, Simon I Hay, Theo Vos, Christopher J L Murray, Harvey A Whiteford, and Alize J Ferrari.

Section 9.7. Drafting the work or revising is critically for important intellectual content
Damian F Santomauro, Ana M Mantilla Herrera, Jamileh Shadid, Charlie Ashbaugh, David Pigott, Peng Zheng, Ali H Mokdad, Simon I Hay, Theo Vos, Christopher J L Murray, Harvey A Whiteford, and Alize J Ferrari.

Section 9.7. Managing the overall research enterprise
Damian F Santomauro, David Pigott, Rafael Lozano, Ali H Mokdad, Simon I Hay, Theo Vos, Christopher J L Murray, Harvey A Whiteford, and Alize J Ferrari.
